# Increased epigenetic alterations at the promoters of transcriptional regulators following inadequate maternal gestational weight gain

**DOI:** 10.1038/srep14224

**Published:** 2015-09-29

**Authors:** Tomoko Kawai, Takahiro Yamada, Kosei Abe, Kohji Okamura, Hiromi Kamura, Rina Akaishi, Hisanori Minakami, Kazuhiko Nakabayashi, Kenichiro Hata

**Affiliations:** 1 Department of Maternal-Fetal Biology, National Research Institute for Child Health and Development, Tokyo 157-8535, Japan; 2 Department of Systems BioMedicine, National Research Institute for Child Health and Development, Tokyo 157-8535, Japan; 3 Division of Developmental Genomics, National Research Institute for Child Health and Development, Tokyo 157-8535, Japan; 4 Department of Obstetrics and Gynecology, Hokkaido University Graduate School of Medicine, Sapporo 060-8638, Japan

## Abstract

Epigenetic modifications are thought to serve as a memory of exposure to *in utero* environments. However, few human studies have investigated the associations between maternal nutritional conditions during pregnancy and epigenetic alterations in offspring. In this study, we report genome-wide methylation profiles for 33 postpartum placentas from pregnancies of normal and foetal growth restriction with various extents of maternal gestational weight gain. Epigenetic alterations accumulate in the placenta under adverse *in utero* environments, as shown by application of Smirnov-Grubbs’ outlier test. Moreover, hypermethylation occurs frequently at the promoter regions of transcriptional regulator genes, including polycomb targets and zinc-finger genes, as shown by annotations of the genomic and functional features of loci with altered DNA methylation. Aberrant epigenetic modifications at such developmental regulator loci, if occurring in foetuses as well, will elevate the risk of developing various diseases, including metabolic and mental disorders, later in life.

Foetal environmental factors, including maternal nutrition, hormonal disturbance, and chemical exposure, affect foetal growth and can cause birth defects. Recent studies have linked poor foetal growth to increased risks of coronary heart disease^1^, type II diabetes^2^,^3^, kidney disease^1^,^4^, and brain disorders^5^ in adulthood. Barker first proposed that nutritional conditions *in utero* may be responsible, at least in part, for the developmental programming of the foetus and placenta, potentially predisposing the individual to adult metabolic disease^6^. The concept that the foetal environment affects health later in life has been defined as the developmental origins of health and disease (DOHaD)^7^, in which epigenetic modifications are thought to serve as a memory of exposure to *in utero* environments^8^,^9^. Epigenetic modifications, such as DNA methylation and histone modifications, are involved in tissue- and developmental stage-specific gene expression and silencing, and they can be transmitted in a stable manner through mitotic cell division, thereby inducing long-term changes in gene regulation. Developmental programming during the foetal period, therefore, could affect adult health through epigenetic mechanisms. In fact, many studies using animal models have demonstrated that nutrient manipulation during pregnancy induces epigenetic alterations at specific loci or globally in the offspring^10^. On the other hand, human studies showing associations of *in utero* conditions and epigenetic alterations have been relatively limited^11^.

The predominant form of DNA methylation is methylation of cytosine in the context of CpG. The diploid human genome contains more than 10^8^ cytosines, of which more than 10^7^ are present in the context of CpGs^12^. The genome-wide DNA methylation pattern in various types of cells in the body is bimodal, with the majority of CpG sites being highly methylated (>85%), while CpG islands (CGIs) are largely unmethylated (<10%). After fertilisation of the oocyte by sperm, the paternal genome becomes actively demethylated in the zygote, and the maternal genome undergoes passive demethylation until the early blastocyst stage^13^. DNA methylation patterns are re-established in a lineage-specific manner^14^,^15^. The genome in cells of the placenta remains relatively hypomethylated compared to that in somatic tissues. A genome-wide DNA methylation analysis of promoter regions in human placental tissues collected during the first, second, and third trimesters revealed that there is a significant increase in the average methylation level in autosomes from the second-trimester placenta to the full-term placenta^16^. Interindividual variations in DNA methylation levels have also been shown to increase during gestation^16^. Foetal environmental factors, such as maternal weight, maternal alcohol intake, maternal smoking, and maternal psychological stress, have recently been shown to affect DNA methylation in the human placenta^11^,^17^,^18^. However, compared to the number of studies that have examined the DNA methylation levels at repetitive sequences, such as long interspersed nuclear elements (LINEs) and short interspersed nuclear elements (SINEs), and at certain imprinted loci^19^,^20^,^21^, studies evaluating the alterations in DNA methylation on the genome-wide scale in relation to the foetal environment have been limited^18^,^21^,^22^,^23^,^24^.

Maternal gestational weight gain (GWG) influences the foetal nutritional environment during gestation. The Japan Ministry of Health, Labour, and Welfare recommends that women with a prepregnancy body mass index (BMI) between 18.5 and 25 should gain about 7–12 kg body weight during the gestational period. Excessive GWG has been reported to be associated with increased neonatal obesity during infancy and adulthood^25^,^26^,^27^. On the other hand, insufficient GWG is related to increased risk of low birth weight^28^,^29^, which is known to be associated with metabolic syndrome, including impaired glucose tolerance, insulin resistance, and coronary heart disease, during adulthood^1^,^2^,^3^,^4^. Foetal growth restriction (FGR), which may be caused by foetal, placental, and/or maternal factors^30^, is defined as a foetus that has not reached its growth potential (below the 10th percentile for gestational age).

In this study, we elucidate the effects of *in utero* environments on the human placental epigenome. To this end, we examine a collection of postpartum placentas using array-based genome-wide DNA methylation analysis and evaluate DNA methylation levels in placental tissues in relation to GWG and birth weight. We demonstrate that inadequate GWG perturbs the placental epigenome variably among subjects, and that such epigenetic alterations occur preferentially at the CGI promoters of genes encoding transcriptional factors. Therefore, our results demonstrate that epigenetic alterations accumulate in the placenta under adverse *in utero* environments, supporting the importance of appropriate *in utero* conditions and maternal health in foetal development.

## Results

### Alterations in placental DNA methylation were associated with FGR and GWG

We subjected placentas from 14 births exhibiting FGR and 19 births within the normal range of birth weight (Table 1) to genome-wide DNA methylation analysis, and assessed whether the FGR placentas contained CpG sites that were differentially methylated compared with the placentas with a birth weight within normal range. Wilcoxon rank-sum tests^31^ did not detect any CpG sites as significantly differentially methylated between two groups (significance level = Benjamini–Hochberg [BH] adjusted *p*-value of 0.05). Comparisons of FGR and normal placentas within subgroups depending on maternal GWG (insufficient, adequate, and excessive) also did not detect any differentially methylated CpG sites in the FGR placentas (Supplementary Fig. 1). Next, we assessed whether placentas from subjects with excessive or insufficient maternal GWG contained CpG sites that were differentially methylated compared with those in placentas from subjects with adequate GWG. Four comparisons (i.e., insufficient versus adequate and excessive versus adequate within FGR and normal categories) did not detect any significantly differentially methylated CpG sites between two subgroups (Supplementary Fig. 1). These results suggested that no specific CpG sites showed consistent changes in DNA methylation associated with the FGR phenotype or inadequate maternal GWG in this study.

**Figure 1:**
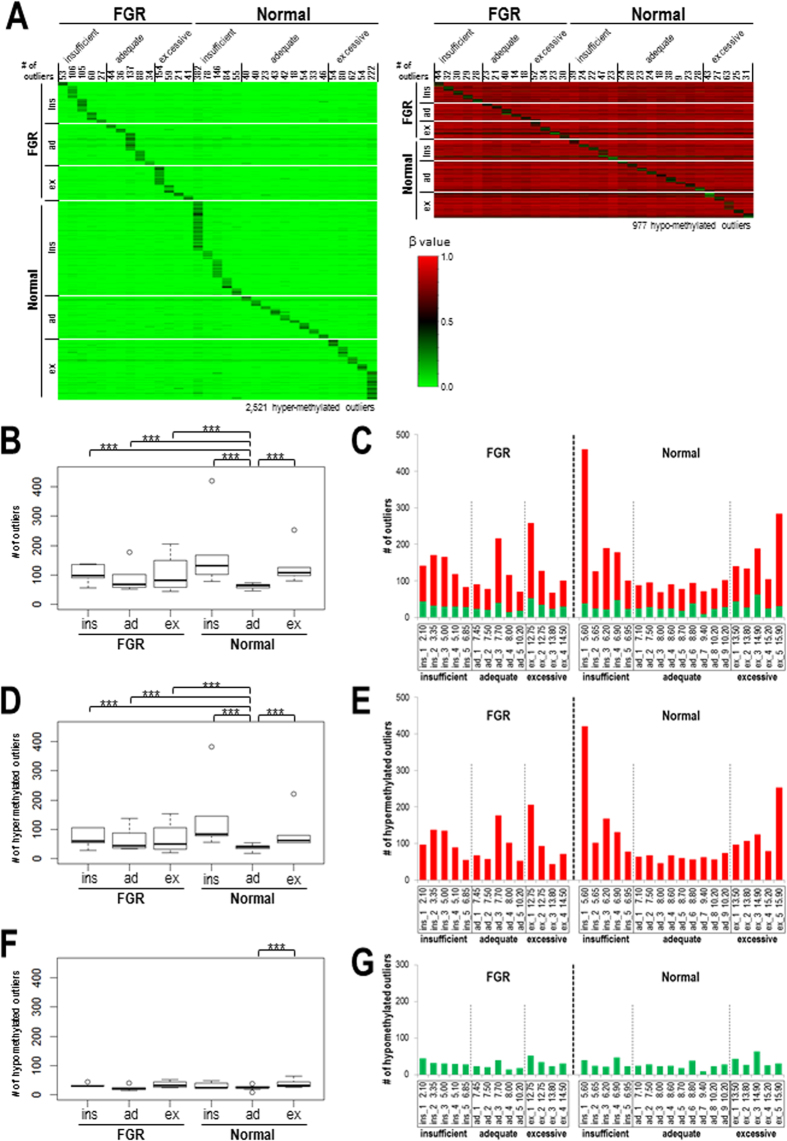
(**A**) Heatmap visualisation of the β value of methylation outliers detected by Smirnov-Grubbs’ outlier tests. The numbers of outliers detected in each placenta are indicated above the heatmap. The colour scale represents the β value from 0 to 1. The left and right panels represent hypermethylated and hypomethylated outliers, respectively (**B,D,F**). Box plots showing the distribution of the numbers of outliers in each of six placental categories (***, Tukey’s multiple comparison test *P*-value < 0.001). ins, insufficient; ad, adequate; ex, excessive (**C,E,F**). Bar plots for the numbers of outliers in FGR and normal subjects sorted according to weight gained during pregnancy. Plots for all outliers (**B,C**), hypermethylated outliers only (**D,E**), and hypomethylated outliers only (**F,G**) are shown. Red and green bars represent the numbers of hyper- and hypomethylated outliers, respectively (**C,E,F**).

Next, we considered the possibility that the FGR phenotype and/or inadequate GWG may affect the placental epigenome in different ways among individual subjects rather than showing similar effects for all individuals within a group. To evaluate this possibility, we searched for CpG sites whose methylation level differed significantly in one placenta (as compared with all of the other placenta samples) by performing Smirnov-Grubbs’ outlier test with Bonferroni multiple test corrections (significant level = 0.1) for each placenta. We detected 2,983 and 1,416 CpG sites as hyper- and hypomethylated outliers, respectively, among the 33 subjects. To reduce the numbers of outliers that could have been detected spuriously due to SNPs at/near the target CpG sites, we excluded the CpG sites whose corresponding probes are annotated to contain known SNPs as described in the Methods. When 89,678 probes were regarded as potentially SNP-containing based on the Illumina probe annotation, 2,521 (85%) and 977 (69%) CpG sites remained as hyper- and hypomethylated outliers, respectively (Fig. 1 and Supplementary Tables 1 and 2).

We subjected these remaining outliers to further data analyses. Hypomethylated outliers coincided with SNP-containing probes more often than hypermethylated outliers (439/1,416 (31%) versus 462/2,983 (15%)). The mean (standard deviation [SD]) β values of the 2,521 hyper- and the 977 hypomethylated outliers were 0.24 (0.13) and 0.56 (0.19), respectively. The mean (SD) Δβ values (Δβ = the β value of the outlier–the mean β value of the other samples) of hyper- and hypomethylated outliers were 0.18 (0.11) and –0.27 (0.12), respectively.

While the numbers of outliers in the “normal_adequate” category were low and relatively consistent among subjects (ranging from 46 to 74), those in the other five categories were higher, exhibiting statistical significance (Tukey’s multiple comparison test *p*-value < 0.001; Fig. 1B) and diverse among the subjects (ranging from 44 to 421). In normal subjects, the greater the insufficiency or excessiveness of maternal GWG, the higher the number of methylation outliers, as represented by the U-shaped appearance of the bar plots for the number of outliers in normal subjects sorted according to weight gained during pregnancy (Fig. 1C). In FGR subjects, all three subcategories (FGR_insufficient, FGR_adequate, and FGR_excessive) contained significantly higher numbers of outliers than the “normal_adequate” category (Fig. 1B). The numbers of outliers in FGR_insufficient and FGR_excessive categories were also significantly higher than those in FGR_adequate (Fig. 1B). The numbers of outliers were neither associated with C-section nor correlated with gestational weeks (Supplementary Table 4). These results suggested that both FGR and inadequate GWG conditions affected the placental epigenome independently and additively.

Next, we examined the numbers of hyper- and hypomethylated outliers in each subject (Fig. 1E,G). While the numbers of hypomethylated outliers were not much different among subjects (Fig. 1F), the numbers of hypermethylated outliers were significantly higher in subjects in the other five categories compared to subjects in the “normal_adequate” category (*P* < 0.001; Fig. 1D). Therefore, only hypermethylated outliers occurred with FGR pregnancies and normal pregnancies with inadequate GWG. Because of the nature of the Smirnov-Grubbs’ outlier test, the identified methylation outliers were all specific to individuals (deviated only in one sample among the cohort). Our results demonstrate that the adverse pregnancy conditions, FGR and inadequate GWG, affected the placental epigenome variably among individuals.

### Contrasting genomic features of hyper- and hypomethylated outliers

**Figure 2:**
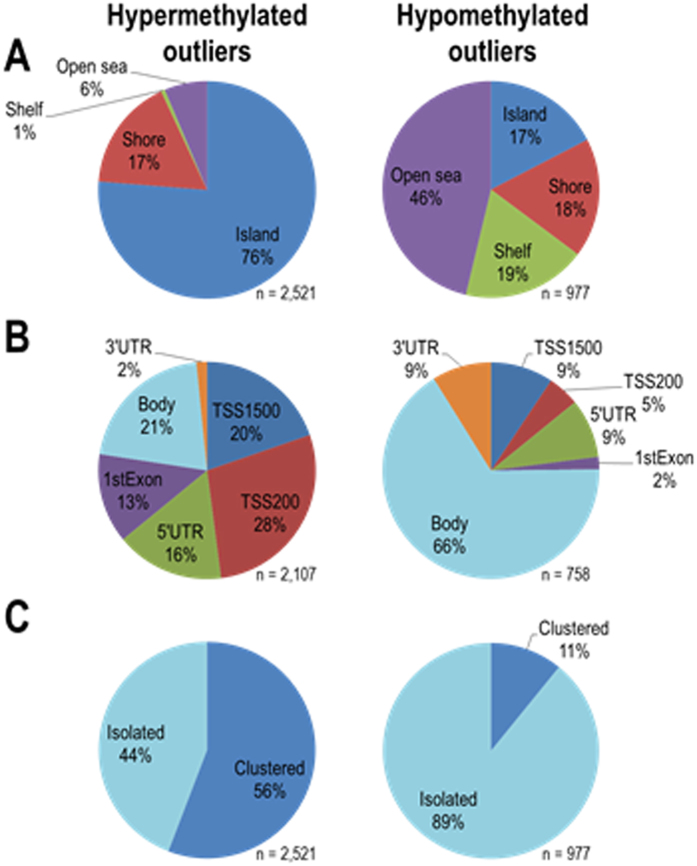
Genomic features of 2,521 hyper- and 977 hypomethylated outliers. Distribution of outliers in relation to CGIs and their shores and shelves (**A**) and to gene feature groups (**B**). Six gene feature categories, i.e., TSS1500, TSS200, the 5′UTR, the first exon, the gene body, and the 3′UTR, are regarded as genic regions, in which 2,107 (84%) and 758 (78%) CpG sites were located. The ratio of clustered and isolated outliers is shown (**C**).

We subsequently annotated the genomic features of 2,521 hyper- and 977 hypomethylated outliers (Fig. 2). Among these outliers, 2,107 (84%) and 758 (78%) CpG sites were located in genic regions (in 1,001 and 606 genes, respectively). Hypermethylated outliers were found to be predominantly located in CGIs or their shores/shelves (94% in total) and proximal to the transcriptional start sites (defined as “pTSS” hereafter; i.e., TSS1500, TSS200, the 5′ untranslated region [UTR], and the first exon categories; 77%). In contrast, hypomethylated outliers were most frequently located outside of CGIs, shores, and shelves (open sea, 46%) and in gene bodies (66%) (Fig. 2A,B). Hypermethylated outliers tended to be detected consecutively at two or more adjacent probes (“clustered”; 56%), while hypomethylated outliers did not (11%; Fig. 2C). These results implied that hypermethylated outliers tended to be clustered within CGI promoters.

**Figure 3:**
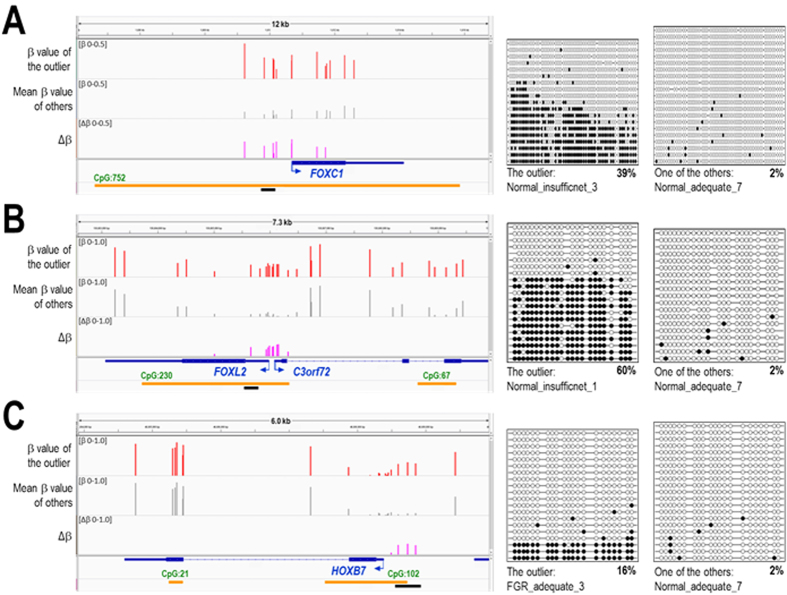
Examples of promoter-wide hypermethylation at *FOXC1* (**A**), *FOXL2* (**B**), and *HOXB7* (**C**) loci. The β value of the outlier, the mean of the β values of samples other than the outlier, and the Δβ are shown together with Refseq gene and UCSC-defined CGIs using IGV at the left side in each panel. The data range of 0 to 0.5 (or 0 to 1.0) is shown for β and Δβ values. The outlier samples for the three loci are Normal_insufficient_3 (**A**), Normal_insufficient_1 (**B**), and FGR_adequate_3 (**C**). DNA methylation status of these promoter regions were validated by targeted bisulfite sequencing (BS). The black horizontal bar at the bottom in each panel shows the interval of the bisulfite-PCR amplicon. The BS results for the outlier sample and a control (Normal_adequate_7) are shown at the right side in each panel. Open and closed circles represent unmethylated and methylated CpG sites, respectively. Each row of circles corresponds to an individual clone sequenced. The overall methylation rate (%) is shown underneath each panel of the BS results.

We therefore scrutinised the extent of hypermethylation and the positional distribution relative to the TSS of hypermethylated outliers by visualising β and Δβ values on the Integrative Genomics Viewer (IGV, www.broadinstitute.org/igv/home). Indeed, we found that hypermethylated outliers were often distributed in a promoter-wide manner (i.e., located consecutively and clustered around the TSS) with relatively large methylation differences, as exemplified by *FOXC1, FOXL2*, and *HOXB7* loci (Fig. 3). The methylation statuses in the outlier sample and a control (Normal_adequate_7) at these promoter regions were validated to be hypermethylated and unmethylated, respectively, by targeted bisulfite sequencing analyses (Fig. 3). The appearance of both of heavily methylated and unmethylated clones in individual outlier samples may indicate the mosaic composition of normal and epimutated cells in these placentas.

The observation that hypermethylated outliers were often clustered at CGI promoters suggested that placental hypermethylation events do not occur in a purely random manner in terms of genomic location, but instead occur due to dysfunction of certain intrinsic mechanisms regulating the epigenetic status of CGI promoters under adverse *in utero* environments.

### Hypermethylated outliers were frequently associated with genes encoding transcriptional regulators

In order to search for functional characteristics of genes containing hypermethylated outliers, we performed gene ontology (GO) analysis; 1,001 genes hosting hypermethylated outliers (as well as 606 genes hosting hypomethylated outliers for comparison) were analysed using the Database for Annotation, Visualization, and Integrated Discovery (DAVID) v6.7. The 606 genes hosting hypomethylated outliers were found to be weakly enriched with only one term, “cytoskeletal protein binding”, in the Molecular Function (MF) category (Benjamini’s corrected *Pc* = 0.0025). However, the 1,001 genes hosting hypermethylated outliers were highly enriched with terms related to transcriptional regulators and neuronal differentiation in the Biological Process (BP) and MF categories (e.g., BP terms “regulation of transcription, DNA-dependent” [*Pc* = 1.96 × 10^−8^] and “neuronal differentiation” [*Pc* = 3.16 × 10^−8^]; Table 2 and Supplementary Table 3). We subsequently performed GO analysis for subgroups of genes: 409 genes hosting highly deviated (Δβ > 0.2) hypermethylated outliers, 709 genes hosting hypermethylated outliers in the pTSS, and 317 genes hosting two or more clustered hypermethylated outliers. These subgroups of genes were also found to be significantly enriched with terms related to transcriptional regulators (Supplementary Table 3). These results supported our observation that hypermethylated outliers are often distributed in a promoter-wide manner and that the genes hosting such outliers are significantly enriched with genes encoding transcriptional regulators. We further performed GO analysis for the 163 genes hosting highly deviated (Δβ > 0.2) and clustered hypermethylated outliers in the pTSS. Among those, 36 genes were assigned to the category “GO:0006355~regulation of transcription, DNA-dependent” with a statistical significance [*Pc* = 0.0038] and showed a higher fold enrichment value to the term than that of the entire (1,001) genes (2.25 versus 1.70, Supplementary Table 3). Importantly, in 35 out of the 36 genes encoding transcriptional regulators (97%), promoter hypermethylation was detected in the placentas from cases of inadequate GWG or FGR (Table 3).

## Discussion

In this study, we demonstrated the possibility that inadequate maternal GWG enhances aberrant DNA methylation in the placenta. We initially failed to identify specific loci whose methylation was commonly altered across all subjects in each of the GWG categories. We subsequently used Smirnov-Grubbs’ outlier tests, which detect the most significantly deviated outlier among subjects, for each of the CpG probes and found that hypermethylated loci accumulated in normal pregnancies with inadequate GWG and in FGR pregnancies. The results suggested that the epigenetically affected loci due to adverse *in utero* environments were variable among the subjects examined in this study. It should be noted that the relatively small number of the enrolled subjects (partly due to exclusion of the subjects with certain types of pregnancy complications) with various layers of heterogeneities (e.g., genetic, phenotypic, and environmental) may account for a primary cause of the absence of commonly epigenetically affected loci and the variation of affected loci among the individuals studied. While many animal studies have clearly demonstrated direct associations between *in utero* nutritional conditions during foetal development and epigenetic alterations (at certain loci or globally)^10^, evidence from studies in human populations has been limited. Unlike the homogeneous genetic backgrounds of animal models and the well-controlled environmental and experimental conditions that can be easily achieved in animal studies, individuals in human studies are genetically heterogeneous and have not been exposed to identical environments throughout their lives. These unavoidable genetic and environmental heterogeneities in human subjects very likely give rise to individual variations in epigenetically affected loci, even when the subjects were exposed to similar nutritional environments for a certain period. Provided that epimutations could occur not only at common loci but at variable loci among subjects, Smirnov-Grubbs’ outlier test is effective in evaluating the extent of the accumulation of the latter type of epimutations under certain disease and/or malnutrition conditions and may be applicable to a wide range of epigenetic studies in human populations.

FGR is idiopathic in most cases and is generally thought to be caused by foetal, placental, maternal, and/or environmental factors^30^. Therefore, the hypermethylation events observed with significantly high frequencies in placentas from FGR births in this study may also be explained by various factors. Unidentified genetic factors, such as foetal and/or placental chromosomal abnormalities and mutations at certain genes, if they exist, could affect the epigenomes of both the foetus and placenta, regardless of *in utero* conditions. Maternal and environmental factors deteriorating *in utero* conditions and contributing to the FGR phenotype may not have been identified in some subjects enrolled in this study. On the other hand, in normal pregnancy cases with inadequate GWG, since the body weights of the babies were within the normal range, the foetuses (and the placentas) were considered to be genetically normal. Under this assumption, promoter hypermethylation observed with higher frequencies in placentas with inadequate GWG than in those with adequate GWG can be regarded as environmentally induced epigenetic alterations.

Multiple independent studies have shown that genetic variants can cause variations in DNA methylation levels, defined as sequence-dependent allele-specific DNA methylation (ASM)^32^. A recent methylC-Seq study of the mouse genome revealed that sequence-dependent ASMs typically exist as isolated CpG sites in intergenic and intronic regions, but are relatively depleted from proximal promoters^33^. Moreover, sequence-dependent ASMs are influenced by defined sequences nearby and they appear to have little effect on gene expression. The genomic features of hypomethylated outliers in our study were similar to those of sequence-dependent ASMs. On the other hand, the characteristics of the hypermethylated outliers, being clustered (56%) in the pTSS (77%), were distinct from those of sequence-dependent ASMs. It is generally challenging to distinguish whether differentially methylated regions among genetically heterogeneous human populations are epimutations or sequence-dependent ASMs. However, considering the above-mentioned genomic features of the hypermethylated outliers as well as their enrichment in the promoter regions of transcriptional regulator genes (which will be discussed in detail in the next paragraph), at least a portion of these outliers likely represent genuine epigenetic alterations rather than sequence-dependent changes in DNA methylation.

We initially considered that placental epimutations may have occurred randomly under aberrant *in utero* environments; our data subsequently revealed that hypermethylated outliers were not found completely randomly in terms of genomic location, but tend to be frequent at the promoters of genes encoding transcription factors. Considering that the promoter regions of genes encoding developmental regulators, such as homeobox proteins and other developmental transcription factors, have been reported to be mostly devoid of sequence-dependent ASMs^33^, the hypermethylated outliers located at the promoter regions of such genes identified in this study (Table 3) most likely represent epigenetic alterations due to aberrant *in utero* environments. In a recent genome-wide DNA methylation study using reduced representation bisulphite sequencing (RRBS) in a murine model of FGR, genes hosting differentially methylated regions in the placenta upon maternal calorie restriction are significantly enriched (*P* < 0.05) with GO terms such as homeobox and transcription factor activity, among others^34^. Notably, our own annotations for the 131 genes hosting hypermethylated regions in the placenta upon maternal gestational calorie restriction^34^ using DAVID revealed that these genes were moderately enriched with genes assigned with the GO Molecular Function term “DNA binding” (17 out of the 131 genes were assigned this term). Therefore, although the statistical method used for detecting differentially methylated regions is different from that in our study, some aspects of this murine study were consistent with our findings demonstrating the enrichment of placental epimutations in transcriptional regulator genes.

Our findings also suggested the possibility that certain epigenetic regulatory systems are susceptible to the disruptive effects of aberrant *in utero* environments. In fact, a careful analysis of the 36 genes assigned with GO terms related to transcriptional regulation (Table 3) revealed that polycomb group repressive complexes (PRCs)^35^ represent a primary candidate of such regulatory mechanisms. We found that seven out of the 36 genes (i.e., *HOXB7, GBX2, HMX2, SOX7, F2R, FOXL2*, and *FOXC1*) were included in the 653 PRC2 targets in mouse embryonic stem cells, as identified by a ChIP-on-chip analysis^36^. Further annotations of the 36 genes using the ChIP-seq data for EZH2 and SUZ12, which are components of PRC2^35^, from a human ES cell line (H1-hESC) produced by the Encyclopedia of DNA Elements (ENCODE) Consortium (http://genome.ucsc.edu/ENCODE/) identified additional eight PRC2 targets (Table 3). Consistent with our observations, epigenetic variation between twin-twin transfusion syndrome children, wherein twin foetuses occasionally exhibit striking growth differences, is most prominent at the CpG sites within the target regions of PRCs^37^. Furthermore, Wilhelm-Benartzi *et al.* reported significant associations of placental LINE-1 and AluYb8 methylation levels with birth weight percentile and significant differences in the methylation levels of these repetitive elements upon maternal alcohol or tobacco use during pregnancy^21^. Interestingly, the authors also revealed the positive association of increased placental AluYb8 methylation with the average methylation levels of CpG sites in polycomb group target genes. Therefore, evidence from these previous reports and our current findings suggest the possibility that PRCs occasionally fail to recognise their targets with a stochastic nature in the placenta under improper *in utero* environments, leading to epigenetic switching from PRC marks (H3K27me3) to DNA methylation. Another striking feature of these 36 genes was that 15 (42%) were zinc-finger genes (Table 3). Zinc-finger genes are often silenced through H3K9me3-mediated gene silencing coupled with promoter DNA methylation in toxicant-induced carcinogenesis, suggesting the existence of an unknown epigenetic mechanism through which many zinc-finger genes are coregulated^38^. This hypothetical regulatory mechanism may also be susceptible to the effects of adverse *in utero* environments.

In addition to the enrichment of GO terms related to transcriptional regulation, the genes hosting hypermethylated outliers were also found to be enriched with the GO term “neuron differentiation” (Supplementary Table 3).This seemingly unexpected observation is consistent with those of previous studies. In an array-based expression study that identified 7,519 genes exhibiting differential expression between human placentas sampled during the first and third trimesters, both up- and downregulated genes in the third trimester were found to be enriched with genes involved in human neurogenesis^39^. The authors of the study have suggested that the brain and placenta possibly share common developmental routes. In the above-mentioned RRBS study of the murine model of intrauterine malnutrition^34^, GO terms found to be enriched in genes hosting altered placental DNA methylation upon maternal caloric restriction were shown to contain neuron-related terms^34^. Additionally, several neural factors, such as BDNF^40^, NGF^41^, and serotonin^42^, have been shown to be secreted from the placenta. Among these factors, BDNF has also been shown to potentiate placental development and play an important role in cytotrophoblast differentiation^43^,^44^. Furthermore, placental BDNF expression has been reported to be significantly correlated with neonatal birth weight^40^ and to be decreased upon maternal malnutrition in rats^45^. Because of the functional significance of a subset of genes in both the placenta and brain, it is tempting to speculate that the foetuses may have gained epigenetic alteration patterns that are similar to those observed in the placenta in pregnancies with inadequate GWG. Hypermethylation at the promoter regions of genes encoding developmental regulators (PRC2 targets) and neuronal regulators at early embryonic stages would reduce their expression levels when these genes are expressed in a spatio-temporal manner, and such aberrant expression of critical developmental regulators may elevate the risk of developing various diseases, including metabolic and mental disorders, later in life.

In this study, we demonstrated that loci with alterations in the placental DNA methylation under inadequate GWG were not common among subjects but were instead distributed in an individual-specific manner. Furthermore, such epigenetic alterations under the adverse pregnancy condition were found to occur preferentially at the CGI promoters of genes encoding transcriptional factors. Our novel findings support the necessity of large-scale epigenomic studies of placental tissues and samples (e.g., cord blood) from newborns for pregnancies under normal and malnutrition conditions, together with follow-up studies when the newborns reach adulthood in order to elucidate the epigenetic mechanisms underlying developmental programming in humans and their roles in health and disease in later life.

## Materials and Methods

### Study design

The present study was approved by the Ethics Committee of the National Center of Child Health and Development (NCCHD), Japan and by the Human Study Committee of the Hokkaido University Hospital, Japan. Informed consent was obtained from all subjects. Pregnant Japanese women who did not have pregnancy complications of gestational diabetes, pre-eclampsia, or pregnancy-induced hypertension were enrolled. All enrolled subjects did not smoke or drink alcohol, and did not exhibit hypertension or proteinuria during pregnancy. Subjects (n = 33) were categorised into six categories according to GWG and newborn birth weight: FGR_adequate, FGR_insufficient, FGR_excessive, normal_adequate, normal_insufficient, and normal_excessive, consisting of 5, 5, 4, 9, 5, and 5 placentas, respectively. Prepregnancy BMIs were similar among all groups. The characteristics of each group are shown in Table 1. BMI, body weight, GWG, and additional clinical information (maternal complication, gestational week, delivery method, and newborn’s gender) for each of the subjects are provided as Supplementary Table 4. Although the Institute of Medicine of the United States recommends that pregnant women whose prepregnancy BMI is in the normal range (18.5–24.9) should gain 11.3–15.9 kg during pregnancy, we defined adequate GWG as gaining 7–12 kg in this study in accordance with the recommendations of the Japan Ministry of Health, Labour, and Welfare^46^. This difference is also consistent with the different average BMIs of Japanese and US women (21.14 ± 3.28^47^ versus 27.05 ± 0.35^48^, respectively).

### Genomic DNA extraction and DNA methylation profiling

Full-term placental samples were obtained from normal caesarean sections or vaginal deliveries. Chorionic villous tissue was obtained from the foetal side of the placenta. Genomic DNA was purified from the tissue using a QIAamp DNA Mini kit (Qiagen, Valencia, CA, USA). Genomic DNA (1.5 μg) was bisulphite converted using an EpiTect Plus DNA Bisulfite Kit (Qiagen). After determining the concentration of bisulphited DNA, 300 ng of bisulphite DNA from each sample was subjected to Illumina Infinium HumanMethylation450 BeadChip analysis using the manufacturer’s standard protocol.

### Data processing

To calculate the DNA methylation levels of more than 480,000 CpG sites assayed on the HumanMethylation450 BeadChip (Illumina), the signal intensity data (.idat files), produced by the Illumina iSCAN system, were processed using Illumina GenomeStudio Methylation Analysis Module v1.9.0 with background subtraction and control normalisation options. The methylation levels were calculated as β values ranging from 0 (completely unmethylated) to 1 (completely methylated; β value = intensity of the methylated allele/[intensity of the unmethylated allele + intensity of the methylated allele + 100]). The obtained data have been deposited in NCBI’s Gene Expression Omnibus and are accessible through GEO accession number GSE62733. From 485,577 probes on the BeadChip array, the following probes were excluded: the probes on sex chromosomes, the probes for 65 random SNPs (which assay highly-polymorphic SNPs rather than DNA methylation), and the probes whose detection *p*-value was higher than 0.01 or whose β value was missing in one or more samples. The β values (methylation levels) of the remaining 449,848 probes were corrected by an Empirical Bayes method, ComBat^49^, to remove the array-batch effect, and subjected to statistical tests.

To detect differentially methylated CpG sites between groups, the Illumina Methylation Analyzer (IMA)^31^ was run using the Wilcoxon rank-sum test for inference of differences between categorical groups. The BH procedure was used for multiple testing corrections, and the cut-off for the adjusted *p*-values was set to 0.05. Smirnov-Grubbs’ outlier test with Bonferroni multiple test corrections was performed using the R Package ‘outliers’ (http://cran.r-project.org/web/packages/outliers/outliers.pdf) and custom R scripts to detect outlying CpG sites, and the cut-off for the corrected *p*-values was set to 0.1.

The Illumina-provided probe annotation, HumanMethylation450_15017482_v.1.1.csv, was used to sort out the outlying CpG sites whose β value could possibly have been affected by sequence variation within the corresponding probe sequence. This table lists 89,678 probes as SNP-containing in its “probe_SNPs” and “probe_SNPs_10” columns based on the information of NCBI dbSNP Build 131. The refSNP information registered in dbSNP Build 142 was also tested for the same purpose of SNP filtering (Supplementary Fig. 2).

When a single CpG site was assigned to multiple gene symbols or gene features in the Illumina probe annotation, only the lead-off gene symbol or feature was used for gene ontology and genome feature annotations.

### Targeted bisulfite sequencing

Bisulfite sequencing analysis was performed as described previously^50^ using bisulfite-PCR primers designed by the MethPrimer website^51^. The forward and reverse primer sequences, and the genomic interval (hg19) of the amplicon are: 5′-GAGAGGTTGGGGTAATTTTAG-3′, 5′-AAAAACTTCTAAACTTTTAAACATCC-3′ and chr6:1609671-1610171 (501bp) for the *FOXC1* locus; 5′-GGGGTAGTTGGTTATTATGATAAAGT-3′, 5′-ACTCCCCATAACCAAAAACTAAACT-3′, and chr3:138665547-138665794 (248 bp) for the *FOXL2* locus; 5′-AGTTTTGTGGATTGGGGTTG-3′, 5′-ACACCTAAAAAAACTTACTCCATCTC-3′, and chr17:46688533-46688920 (388 bp) for the *HOXB7* locus. The obtained sequence data were analysed using the QUMA website^52^.

## Additional Information

**How to cite this article**: Kawai, T. *et al.* Increased epigenetic alterations at the promoters of transcriptional regulators following inadequate maternal gestational weight gain. *Sci. Rep.* **5**, 14224; doi: 10.1038/srep14224 (2015).

## Supplementary Material

Supplementary Information

## Figures and Tables

**Table 1:** Characteritics of mothers, newborns, and placentas enrolled in this study.

	FGR			Normal		
	adequate	insufficient	excessive	adequate	insufficient	excessive
	(n => 5)	(n = 5)	(n = 4)	(n = 9)	(n = 5)	(n = 5)
BMI of pre-pregnancy (kg/m^2^)	20.3 ± 2.0	18.7 ± 1.1	19.6 ± 1.0	20.1 ± 1.9	19.7 ± 2.2	19.8 ± 1.1
BMI at delivery (kg/m^2^)	23.7 ± 2.5	20.4 ± 1.4^a^	25.2 ± 1.6	23.5 ± 1.7	22.3 ± 1.9	25.5 ± 1.2^d^
B.W. of pre-pregnancy (kg)	49.0 ± 5.3	49.4 ± 5.9	46.5 ± 1.7	51.0 ± 5.4	48.0 ± 7.0	50.6 ± 3.6
B.W. at delivery (kg)	57.2 ± 5.7	53.9 ±± 6.5	60.0 ± 2.2	59.7 ± 4.8	54.3 ± 6.5	65.3 ± 3.9^d^
Gestational weight gain (kg)	8.2 ± 1.2	4.5 ± 1.8^a^	13.5 ± 0.9^b^	8.7 ± 1.1	6.3 ± 0.7^c^	14.7 ± 1.0^d^
B.W. of newborn (g)	1984 ± 296^e^	1702 ± 282^f^	1860 ± 535^g^	2937 ± 297	3010 ± 483	3452 ± 200
B.W./B.H. of newborn (cm/g)	45.4 ± 5.2^e^	40.9 ± 5.5^f^	43.8 ± 7.7^g^	61.3 ± 4.0	62.5 ± 7.9	68.3 ± 3.6^d^
Placental weight (P.W.) (g)	417.0 ± 47.9^e^	351.0 ± 83.2^f^	487.5 ± 24.0^b^,^g^	631.7 ± 189.2	639.0 ± 132.9	652.0 ± 97.8
B.W. of newborn/P.W.	4.8 ± 0.5	5.0 ± 1.1	3.8 ± 1.1^g^	4.9 ± 1.2	4.8 ± 1.0	5.4 ± 0.7
Gestational weeks	37.0 ± 1.4	35.8 ± 1.3^f^	36.3 ± 3.0	38.0 ± 1.7	39.6 ± 1.7	39.6 ± 1.1
Ratio of C. section to vaginal delivery	3 to 2	2 to 3	2 to 2	2 to 7	1 to 4	1 to 4
Ratio of male to female newborns	3 to 2	2 to 3	0 to 4	6 to 3	1 to 4	3 to 2
Age at delivery	31.4 ± 3.6	28.6 ± 2.4	26.8 ± 4.0	32.0 ± 7.1	31.4 ± 7.6	32.6 ± 5.9

BMI, body mass index; B. W., body weight; B. H., body height.

^a^*p* < 0.05 in the t-test between FGR_insufficient and FGR_adequate.

^b^*p* < 0.05 in the t-test between FGR_excess and FGR_adequate.

^c^*p* < 0.05 in the t-test between Normal_insufficient and Normal_adequate.

^d^*p* < 0.05 in the t-test between Normal_excess and Normal_adequate.

^e^*p* < 0.05 in the t-test between FGR_adequate and Normal_adequate.

^f^*p* < 0.05 in the t-test between FGR_insufficient and Normal_insufficient.

^g^*p* < 0.05 in the t-test between FGR_excess and Normal_excess.

**Table 2:** Top 5 gene ontology (GO) Biological Process terms significantly enriched among the 1,001 genes hosting 2,521 hypermethylated outliers.

Gene ontology term(Biological Process)	Genecount	Foldenrichment	*p*-value	Benjamini’sadjusted *p*-value
GO:0006355 regulation of transcription, DNA-dependent	157	1.70	6.73E-12	1.96E-08
GO:0030182 neuron differentiation	60	2.63	1.29E-11	1.88E-08
GO:0051252 regulation of RNA metabolic process	157	1.66	3.85E-11	3.74E-08
GO:0045449 regulation of transcription	199	1.47	2.39E-09	1.74E-06
GO:0007409 axonogenesis	31	3.08	6.61E-08	3.85E-05

**Table 3:** The list of 36 genes assigned with transcription factor-related gene ontology terms among the 163 genes hosting highly-deviated and clustered hypermethylated outliers in pTSS.

Subject	GeneSymbol	Gene Name	# ofhyper-methylatedoutliers in pTSS	averageΔβ ofoutliers
N_adequate_5	ZNF649**	zinc finger protein 649	6	0.19
N_excessive_1	GBX2*	gastrulation brain homeobox 2	3	0.25
N_excessive_1	ZNF350**	zinc finger protein 350	8	0.30
N_excessive_2	ZFP37**	zinc finger protein 37 homolog	4	0.44
N_excessive_5	ZHX2*	zinc fingers and homeoboxes 2	8	0.15
N_excessive_5	CDKN1C*	cyclin-dependent kinase inhibitor 1C	4#	0.28
N_excessive_5	PAX6*	paired box 6	2	0.23
N_insufficient_1	KCNH8*	potassium voltage-gated channel, subfamily H, member 8	4	0.24
N_insufficient_1	FOXL2*	forkhead box L2	10	0.21
N_insufficient_1	F2R*	coagulation factor II (thrombin) receptor	3	0.20
N_insufficient_1	SOX7*	SRY (sex determining region Y)-box 7	3	0.18
N_insufficient_1	NFIB*	nuclear factor I/B	2	0.37
N_insufficient_1	HMX2*	H6 family homeobox 2	2	0.23
N_insufficient_2	TFCP2*	transcription factor CP2	5	0.19
N_insufficient_3	PROX1*	prospero homeobox 1	3	0.15
N_insufficient_3	FOXC1*	forkhead box C1	8	0.19
N_insufficient_3	FOXB1*	forkhead box B1	2	0.17
N_insufficient_5	*ETV1*	ets variant 1	6	0.11
N_insufficient_5	ZNF426**	zinc finger protein 426	2	0.17
FGR_adequate_1	*PER1*	period homolog 1	6	0.16
FGR_adequate_3	ZNF619**	zinc finger protein 619	4	0.27
FGR_adequate_3	ZKSCAN4**	zinc finger with KRAB and SCAN domains 4	3	0.25
FGR_adequate_3	*PGBD1*	piggyBac transposable element derived 1	7	0.21
FGR_adequate_3	HOXB7*	homeobox B7	4	0.23
FGR_adequate_4	ESR1*	estrogen receptor 1	3#	0.17
FGR_adequate_4	*MGA*	MAX gene associated	3	0.17
FGR_excessive_1	RFX8*	hypothetical protein LOC731220	3	0.33
FGR_excessive_1	ZNF483**	zinc finger protein 483	2	0.20
FGR_excessive_1	ZNF254**	zinc finger protein 254	3#	0.21
FGR_excessive_2	ZNF577**	zinc finger protein 577	6#	0.43
FGR_excessive_3	ZNF655**	zinc finger protein 655	2	0.35
FGR_insufficient_1	ZNF562**	zinc finger protein 562	8	0.36
FGR_insufficient_1	ZNF805**	zinc finger protein 805	2	0.30
FGR_insufficient_2	ZNF583**	zinc finger protein 583	6	0.27
FGR_insufficient_3	ZNF354C**	zinc finger protein 354C	7	0.27
FGR_insufficient_3	*ETV1*	ets variant 1	6	0.16
FGR_insufficient_4	ZIK1**	zinc finger protein interacting with K protein 1	2	0.42

^*^Polycomb repressive complex 2 (PRC2) target genes in mouse or human embryonic stem cell lines (16/36, 44%). The 653 mouse PRC2-targets identified in Ref.36 and the ChIP-seq dataset of the PRC2 components (EZH2 and SUZ12) for a human ES cell line (H1-hESC) produced by the Encyclopedia of DNA Elements (ENCODE) Consortium were refered to search for PRC2-targets among the 36 genes.

^**^Zinc-finger genes (15/36, 42%).

^#^indicates gene body probes nearby (<1 kb) the pTSS region.
