# Supplementary material for: Increased epigenetic alterations at the promoters of transcriptional regulators following inadequate maternal gestational weight gain
Source: Sci Rep. 2015 Sep 29;5:14224. doi: 10.1038/srep14224 (PMC4586460; doi:10.1038/srep14224)
Supplement: Supplementary Information [file srep14224-s1.pdf]

List of Supplementary Figures and Tables

Increased epigenetic alterations at the promoters of transcriptional regulators following inadequate maternal gestational weight gain

Tomoko Kawai, Takahiro Yamada, Kosei Abe, Kohji Okamura, Hiromi Kamura, Rina Akaishi, Hisanori Minakami, Kazuhiko Nakabayashi, Kenichiro Hata

Supplementary Fig.1:
Schematic representation of the group comparisons performed using Wilcoxon rank-sum test to detect differentially methylated CpG sites

Supplementary Fig.2:
Evaluation of the numbers of SNP-containing probes among the probes corresponding to the 4,399 methylation outliers among 33 placentas detected by the Smirnov-Grubbs' outlier test

Supplementary Table 1:
List of 2,521 hypermethylated outliers

Supplementary Table 2:
List of 977 hypomethylated outliers

Supplementary Table 3:
Full results of Gene ontlogy term analysis for the full and partial lists of hyper- and hypo-methylated outliers using DAVID

Supplementary Table 4
Additional clinical information of the subjects

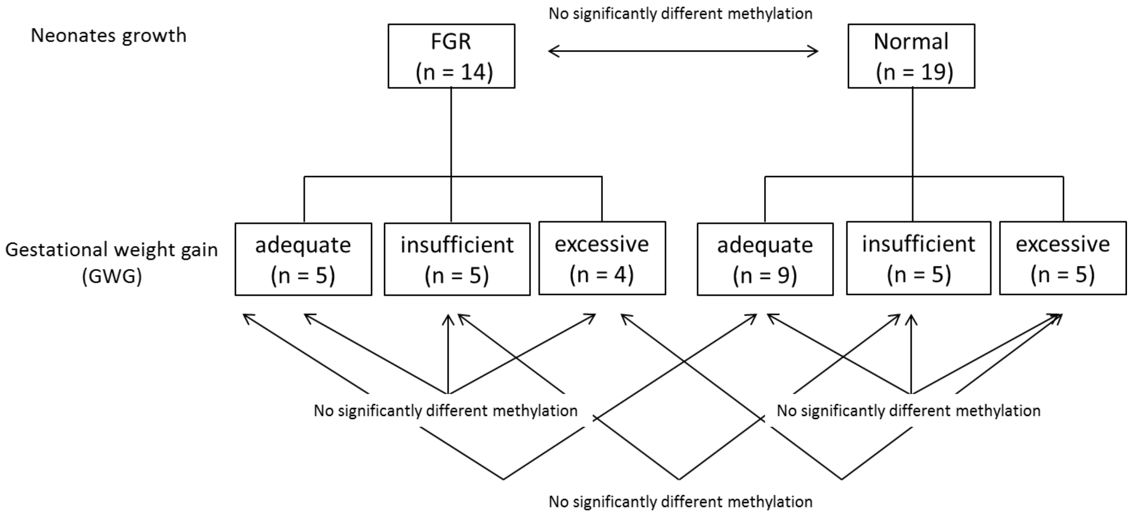

Supplementary Figure 1:

Schematic representation of the group comparisons performed using Wilcoxon rank-sum test to detect differentially methylated CpG sites.

FGR (n=14) and Normal (n=19) birth groups were further categorized according to the extent of maternal gestational weight gain (GWG) (adequate, insufficient, or excessive). Among 449,848 CpG sites subjected to the statistical analysis, no sites showed statistically significant differential methylation in the comparison between FGR and Normal groups and in the seven comparisons between two subgroups (significance level = BH adjusted *p*-value 0.05).

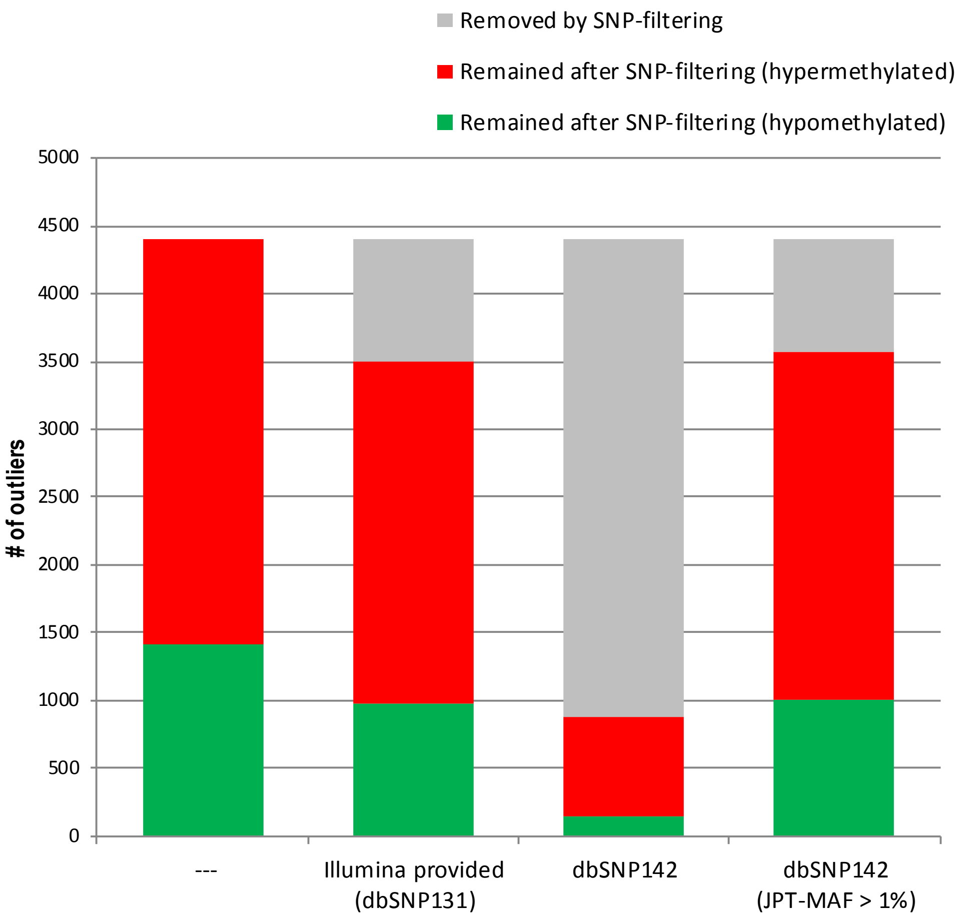

	---	Illumina provided (dbSNP131)	dbSNP142	dbSNP142 (JPT-MAF > 1%)
Removed by SNP-filtering	---	901 / 4399 (20.5%)	3523 / 4399 (80.0%)	832 / 4399 (18.9%)
Remained after SNP-filtering	---	3498 / 4399 (79.5%)	876 / 4399 (20.0%)	3567 / 4399 (81.1%)
hypermethylated	2983 / 4399 (67.8%)	2521 / 3498 (72.1%)	729 / 876 (83.2%)	2569 / 3567 (72.0%)
hypomethylated	1416 / 4399 (32.2%)	977 / 3498 (27.9%)	147 / 876 (16.8%)	998 / 3567 (28.0%)

Supplementary Figure 2:

Evaluation of the numbers of SNP-containing probes among the probes corresponding to the 4,399 methylation outliers detected by the Smirnov-Grubbs' outlier test.

Illumina-provided list for SNP-containing probes were initially used to count the number of the outlying CpG sites whose beta value could possibly have been affected by sequence variation within the corresponding probe sequence. The Illumina probe annotation, HumanMethylation450_15017482_v.1.1.csv, lists 89,678 probes as SNP-containing in its "probe_SNPs" and "probe_SNPs_10" columns based on the information of NCBI dbSNP Build 131. Among the probes corresponding to the 4,399 outliers, 901 (20.5%) probes were found

to be annotated as SNP-containing. The numbers of remaining hyper- and hypomethylated outliers after SNP filtering were 2,521 and 977, respectively. Hypomethylated outliers tended to coincide with SNP-containing probes more often than hypermethylated outliers.

We also conducted SNP-filtering using the dbSNP142 dataset (the current second latest version). This SNP filtering removed an extremely high fraction of probes (80.0% removed). However, many of the SNPs located in the intervals of the probes removed were expected to be monomorphic (or with a very low minor allele frequency) in the Japanese population. Therefore, to keep the number of remaining probes after SNP-filtering appropriate for subsequent feature annotations, we also tested a filtering using only SNPs whose minor allele frequency is >1% in the Japanese population. In order to identify SNPs and short indels that are common (MAF > 1%) in the Japanese population, we used datasets from dbSNP build 142 (ftp://ftp.wip.ncbi.nlm.nih.gov/snp/organisms/human_9606_b142_GRCh37p13/VCF/All_20150217.vcf.gz) and the 1000 Genomes Project (ftp://ftp.ncbi.nih.gov/1000genomes/ftp/release/20130502/). Allele frequency among the Japanese individuals (JPT samples) was calculated for each of refSNP entries when it was included in the variant list of the latter project. Genotypes of 48 female and 56 male JPT samples (from NA18939 to NA19091 with some missing data) were available in the release. The compiled list of refSNPs and their allele frequencies among the JPT samples is available upon request. Among 82,009,761 refSNPs registered in dbSNP142, 9,061,456 SNPs showing a MAF > 1% in the JPT population were used for SNP-filtering. As a result, 832 (18.9%) among 4,399 outlier probes were removed as SNP-containing probes.

We confirmed that approximately 90% of the probes were common between the two remaining outlier probe-sets after the SNP filtering using Illumina provided list (dbSNP131-based) and after the dbSNP142 filtering with the consideration of MAF (> 1%), and that genomic and functional features (such as those shown in Fig.2, Table 2 and Table 3) were similar between them (data not shown).

Supplementary Table 1: list of 2,521 hypermethylated outliers

#	Sample_ID	Outlier-test P-value (adjusted)	Outlier's beta-value	Mean of the beta-values of others	delta-beta	CHR	MAPINFO_hg19	TargetID	UCSC_REFGENE_NAME	UCSC_REFGENE_GROUP	UCSC_CPG_ISLANDS_NAME	RELATION_TO_ UCSC_CPG_ISLAND
hyper1	FGR_insufficient_1	2.9E-02	0.83	0.51	0.32	2	10152012	cg06819088				
hyper2	FGR_insufficient_1	1.9E-03	0.82	0.56	0.26	2	10152901	cg14811551				
hyper3	FGR_insufficient_1	1.5E-03	0.88	0.56	0.32	2	10155062	cg07951151				
hyper4	FGR_insufficient_1	5.4E-03	0.18	0.07	0.11	2	145277741	cg19123296	ZEB2;ZEB2;ZEB2;ZEB2;ZEB2	Body;5'UTR;1stExon;1stExon;5'U	chr2:145281736-145282269	N_Shelf
hyper5	FGR_insufficient_1	2.8E-03	0.51	0.26	0.25	2	241519705	cg13857354				
hyper6	FGR_insufficient_1	3.5E-04	0.29	0.05	0.24	3	145878177	cg00506629	PLOD2;PLOD2	Body;Body	chr3:145878430-145879287	N_Shore
hyper7	FGR_insufficient_1	1.9E-10	0.13	0.01	0.12	4	106630029	cg17197981	GSTCD;INTS12;INTS12;GSTCD	1stExon;TSS200;TSS200;5'UTR	chr4:106629575-106630214	Island
hyper8	FGR_insufficient_1	2.4E-05	0.26	0.09	0.17	4	113970660	cg01268541	ANK2;ANK2;ANK2	TSS200;Body;TSS200		
hyper9	FGR_insufficient_1	5.9E-03	0.33	0.15	0.19	5	168729558	cg21545840	SLIT3	TSS1500	chr5:168727429-168728275	S_Shore
hyper10	FGR_insufficient_1	1.5E-03	0.54	0.24	0.30	6	27661349	cg16615900				
hyper11	FGR_insufficient_1	1.4E-04	0.23	0.10	0.13	6	27661566	cg23078194				
hyper12	FGR_insufficient_1	5.7E-06	0.28	0.04	0.24	6	30071226	cg11002033	TRIM31	3'UTR	chr6:30071225-30071428	Island
hyper13	FGR_insufficient_1	5.1E-02	0.31	0.04	0.27	6	41605343	cg17094014	MDFI	TSS1500	chr6:41604583-41606663	Island
hyper14	FGR_insufficient_1	1.9E-10	0.49	0.09	0.40	7	1272325	cg07765161	UNCX	TSS1500	chr7:1269165-1282864	Island
hyper15	FGR_insufficient_1	1.6E-03	0.50	0.08	0.41	8	11665843	cg00262122	FDFT1	Body	chr8:11665773-11666045	Island
hyper16	FGR_insufficient_1	5.1E-02	0.52	0.12	0.40	8	116660586	cg03277455	TRPS1	5'UTR	chr8:116660432-116660747	Island
hyper17	FGR_insufficient_1	1.3E-07	0.49	0.12	0.37	9	16829168	cg07941680	BNC2	Body	chr9:16828935-16829169	Island
hyper18	FGR_insufficient_1	5.9E-07	0.26	0.08	0.18	14	65007512	cg16319578	HSPA2;HSPA2	1stExon;5'UTR	chr14:65006874-65009197	Island
hyper19	FGR_insufficient_1	1.1E-02	0.38	0.18	0.20	14	65008641	cg10856875	HSPA2	1stExon	chr14:65006874-65009197	Island
hyper20	FGR_insufficient_1	1.1E-03	0.16	0.08	0.08	14	65009101	cg15802091	HSPA2	1stExon	chr14:65006874-65009197	Island
hyper21	FGR_insufficient_1	8.9E-03	0.30	0.07	0.23	15	88800510	cg04920689	NTRK3;NTRK3;NTRK3	TSS1500;TSS1500;TSS1500	chr15:88799522-88801017	Island
hyper22	FGR_insufficient_1	3.1E-05	0.15	0.04	0.11	16	699218	cg02734472	WDR90	TSS200	chr16:698826-700244	Island
hyper23	FGR_insufficient_1	1.1E-04	0.14	0.03	0.11	16	699293	cg08806263	WDR90	TSS200	chr16:698826-700244	Island
hyper24	FGR_insufficient_1	1.0E-07	0.40	0.13	0.28	17	26645325	cg10342447	TMEM97	TSS1500	chr17:26645290-26645614	Island
hyper25	FGR_insufficient_1	4.0E-06	0.47	0.05	0.42	17	26645466	cg19026207	TMEM97	TSS1500	chr17:26645290-26645614	Island
hyper26	FGR_insufficient_1	2.3E-08	0.45	0.07	0.38	17	26645861	cg01273840	TMEM97	TSS1500	chr17:26645860-26646605	Island
hyper27	FGR_insufficient_1	8.0E-08	0.48	0.06	0.42	17	26645865	cg03772856	TMEM97	TSS1500	chr17:26645860-26646605	Island
hyper28	FGR_insufficient_1	0.0E+00	0.32	0.01	0.31	17	26645920	cg07078622	TMEM97	TSS200	chr17:26645860-26646605	Island
hyper29	FGR_insufficient_1	7.5E-09	0.37	0.00	0.37	17	26645922	cg21784498	TMEM97	TSS200	chr17:26645860-26646605	Island
hyper30	FGR_insufficient_1	0.0E+00	0.34	0.02	0.31	17	26646013	cg12694510	TMEM97	TSS200	chr17:26645860-26646605	Island
hyper31	FGR_insufficient_1	2.9E-03	0.49	0.01	0.47	17	26646107	cg05898602	TMEM97	TSS200	chr17:26645860-26646605	Island
hyper32	FGR_insufficient_1	1.1E-04	0.48	0.03	0.45	17	26646373	cg07097636	TMEM97	1stExon	chr17:26645860-26646605	Island
hyper33	FGR_insufficient_1	2.8E-08	0.41	0.08	0.33	17	26646651	cg01438829	TMEM97	Body	chr17:26645290-26645614	S_Shore
hyper34	FGR_insufficient_1	3.7E-05	0.07	0.01	0.06	17	37213281	cg04029055				
hyper35	FGR_insufficient_1	9.0E-03	0.15	0.02	0.13	17	62658371	cg25704638	SMURF2;SMURF2	5'UTR;1stExon	chr17:62657409-62658858	Island
hyper36	FGR_insufficient_1	1.1E-02	0.30	0.08	0.22	17	62658750	cg04808672	SMURF2	TSS1500	chr17:62657409-62658858	Island
hyper37	FGR_insufficient_1	3.9E-02	0.24	0.10	0.14	17	62658827	cg22459320	SMURF2	TSS1500	chr17:62657409-62658858	Island
hyper38	FGR_insufficient_1	1.8E-08	0.30	0.05	0.25	19	9785295	cg09668689	ZNF562;ZNF562;ZNF562	5'UTR;5'UTR;5'UTR	chr19:9785480-9785806	N_Shore
hyper39	FGR_insufficient_1	1.1E-08	0.46	0.03	0.43	19	9785511	cg21610904	ZNF562;ZNF562;ZNF562	5'UTR;5'UTR;5'UTR	chr19:9785480-9785806	Island
hyper40	FGR_insufficient_1	0.0E+00	0.48	0.07	0.42	19	9785727	cg24874111	ZNF562;ZNF562;ZNF562;ZNF562	1stExon;5'UTR;5'UTR;5'UTR;1stE	chr19:9785480-9785806	Island
hyper41	FGR_insufficient_1	0.0E+00	0.48	0.09	0.39	19	9785779	cg18587364	ZNF562;ZNF562;ZNF562	TSS200;TSS200;TSS200	chr19:9785480-9785806	Island
hyper42	FGR_insufficient_1	1.9E-10	0.46	0.08	0.38	19	9785805	cg20631204	ZNF562;ZNF562;ZNF562	TSS200;TSS200;TSS200	chr19:9785480-9785806	Island
hyper43	FGR_insufficient_1	0.0E+00	0.39	0.04	0.35	19	9785906	cg14073063	ZNF562;ZNF562;ZNF562	TSS200;TSS200;TSS200	chr19:9785480-9785806	S_Shore
hyper44	FGR_insufficient_1	0.0E+00	0.42	0.03	0.40	19	9785919	cg14308647	ZNF562;ZNF562;ZNF562	TSS200;TSS200;TSS200	chr19:9785480-9785806	S_Shore
hyper45	FGR_insufficient_1	0.0E+00	0.39	0.05	0.34	19	9786027	cg17704570	ZNF562;ZNF562;ZNF562	TSS1500;TSS1500;TSS1500	chr19:9785480-9785806	S_Shore
hyper46	FGR_insufficient_1	8.0E-09	0.27	0.07	0.20	19	9786115	cg17691880	ZNF562;ZNF562;ZNF562	TSS1500;TSS1500;TSS1500	chr19:9785480-9785806	S_Shore
hyper47	FGR_insufficient_1	4.4E-06	0.36	0.03	0.33	19	53758521	cg04154138	ZNF677	TSS1500	chr19:53757819-53758148	S_Shore
hyper48	FGR_insufficient_1	6.4E-03	0.48	0.12	0.36	19	57751591	cg03252986	ZNF805;ZNF805	TSS1500;TSS1500	chr19:57751888-57752286	N_Shore
hyper49	FGR_insufficient_1	3.4E-02	0.29	0.05	0.24	19	57751691	cg02446106	ZNF805;ZNF805	TSS1500;TSS1500	chr19:57751888-57752286	N_Shore
hyper50	FGR_insufficient_1	0.0E+00	0.29	0.06	0.23	20	14318152	cg23127998	FLRT3;MACROD2;FLRT3;FLRT3;	5'UTR;Body;5'UTR;1stExon;1stExon		
hyper51	FGR_insufficient_1	1.1E-07	0.17	0.04	0.12	20	14318398	cg06722503	FLRT3;MACROD2;FLRT3	TSS200;Body;TSS200		
hyper52	FGR_insufficient_1	1.3E-03	0.17	0.07	0.11	20	14318410	cg26082838	FLRT3;MACROD2;FLRT3	TSS200;Body;TSS200		
hyper53	FGR_insufficient_1	4.1E-05	0.12	0.02	0.10	22	38221095	cg01961447	GALR3	Body	chr22:38220589-38221619	Island
hyper54	FGR_insufficient_2	7.9E-02	0.09	0.06	0.04	1	35496883	cg17434472	ZMYM6	5'UTR	chr1:35497121-35497587	N_Shore
hyper55	FGR_insufficient_2	6.4E-06	0.23	0.06	0.17	1	91172982	cg19731044			chr1:91172102-91172771	S_Shore
hyper56	FGR_insufficient_2	5.6E-04	0.14	0.04	0.10	1	111217406	cg26013553	KCNA3	1stExon	chr1:111216244-111217937	Island
hyper57	FGR_insufficient_2	0.0E+00	0.20	0.02	0.19	1	111217497	cg11595545	KCNA3;KCNA3	1stExon;5'UTR	chr1:111216244-111217937	Island
hyper58	FGR_insufficient_2	3.1E-02	0.17	0.09	0.08	1	111217715	cg09410234	KCNA3	TSS200	chr1:111216244-111217937	Island
hyper59	FGR_insufficient_2	4.2E-02	0.25	0.06	0.19	1	207818449	cg26750487	CR1L	TSS200	chr1:207818389-207818952	Island
hyper60	FGR_insufficient_2	8.0E-02	0.22	0.07	0.15	2	290317	cg03468249			chr2:287353-290099	S_Shore
hyper61	FGR_insufficient_2	2.2E-04	0.26	0.11	0.15	2	290382	cg11432790			chr2:287353-290099	S_Shore
hyper62	FGR_insufficient_2	3.5E-07	0.09	0.01	0.08	2	75874389	cg23889112	MRPL19	Body	chr2:75873981-75874420	Island
hyper63	FGR_insufficient_2	1.0E-03	0.17	0.07	0.11	2	145275197	cg17751438	ZEB2;ZEB2;ZEB2	Body;5'UTR;5'UTR	chr2:145273456-145275378	Island
hyper64	FGR_insufficient_2	9.2E-02	0.14	0.08	0.06	2	145275199	cg21701531	ZEB2;ZEB2;ZEB2	Body;5'UTR;5'UTR	chr2:145273456-145275378	Island
hyper65	FGR_insufficient_2	2.8E-07	0.10	0.01	0.09	2	176957842	cg04415176	HOXD13	1stExon	chr2:176957054-176958279	Island
hyper66	FGR_insufficient_2	4.0E-05	0.37	0.03	0.33	3	36805814	cg24144083			chr3:36805949-36806214	N_Shore
hyper67	FGR_insufficient_2	0.0E+00	0.38	0.05	0.32	3	42725643	cg13010797	KBTBD5	TSS1500	chr3:42727084-42727916	N_Shore
hyper68	FGR_insufficient_2	3.5E-04	0.08	0.02	0.06	3	194408845	cg09652652	FAM43A;FAM43A	3'UTR;1stExon	chr3:194406440-194409045	Island
hyper69	FGR_insufficient_2	1.5E-04	0.18	0.02	0.15	4	157997554	cg11163620	GLRB;GLRB;GLRB;GLRB	5'UTR;5'UTR;5'UTR;1stExon	chr4:157997166-157997686	Island
hyper70	FGR_insufficient_2	8.3E-02	0.12	0.07	0.05	5	691229	cg22736718	TPPP	5'UTR	chr5:691080-691376	Island
hyper71	FGR_insufficient_2	4.0E-08	0.27	0.02	0.25	5	71404443	cg21952901	MAP1B	Body	chr5:71402877-71404868	Island
hyper72	FGR_insufficient_2	1.6E-02	0.12	0.02	0.10	5	132948217	cg04504441	FSTL4;FSTL4	1stExon;5'UTR	chr5:132946744-132948467	Island
hyper73	FGR_insufficient_2	2.0E-07	0.18	0.07	0.11	5	146257653	cg03225002	PPP2R2B;PPP2R2B;PPP2R2B;P	5'UTR;5'UTR;1stExon;Body;5'UTR	chr5:146257346-146258575	Island
hyper74	FGR_insufficient_2	1.6E-04	0.17	0.07	0.09	5	146258034	cg03693911	PPP2R2B;PPP2R2B;PPP2R2B;P	5'UTR;5'UTR;1stExon;Body;5'UTR	chr5:146257346-146258575	Island
hyper75	FGR_insufficient_2	3.1E-08	0.20	0.08	0.12	5	146258159	cg14693112	PPP2R2B;PPP2R2B;PPP2R2B;P	TSS200;5'UTR;Body;5'UTR;5'UTR	chr5:146257346-146258575	Island
hyper76	FGR_insufficient_2	3.1E-06	0.15	0.08	0.07	5	146258181	cg08774231	PPP2R2B;PPP2R2B;PPP2R2B;P	TSS200;5'UTR;Body;5'UTR;5'UTR	chr5:146257346-146258575	Island
hyper77	FGR_insufficient_2	1.9E-10	0.19	0.05	0.14	5	146258189	cg04583285	PPP2R2B;PPP2R2B;PPP2R2B;P	TSS200;5'UTR;Body;5'UTR;5'UTR	chr5:146257346-146258575	Island
hyper78	FGR_insufficient_2	3.9E-08	0.18	0.03	0.15	5	146258195	cg09528265	PPP2R2B;PPP2R2B;PPP2R2B;P	TSS200;5'UTR;Body;5'UTR;5'UTR	chr5:146257346-146258575	Island
hyper79	FGR_insufficient_2	1.3E-09	0.21	0.05	0.16	5	146258354	cg03851984	PPP2R2B;PPP2R2B;PPP2R2B;P	TSS200;5'UTR;Body;5'UTR;5'UTR	chr5:146257346-146258575	Island
hyper80	FGR_insufficient_2	3.6E-08	0.17	0.03	0.14	5	146258427	cg25149751	PPP2R2B;PPP2R2B;PPP2R2B;P	5'UTR;Body;5'UTR;5'UTR;Body;T	chr5:146257346-146258575	Island
hyper81	FGR_insufficient_2	5.3E-09	0.22	0.07	0.15	5	146258461	cg03659005	PPP2R2B;PPP2R2B;PPP2R2B;P	TSS200;5'UTR;Body;5'UTR;TSS2	chr5:146257346-146258575	Island
hyper82	FGR_insufficient_2	3.1E-09	0.24	0.03	0.21	5	146258463	cg25363885	PPP2R2B;PPP2R2B;PPP2R2B;P	TSS200;5'UTR;Body;5'UTR;TSS2	chr5:146257346-146258575	Island
hyper83	FGR_insufficient_2	9.5E-04	0.20	0.06	0.14	5	146258512	cg13971892	PPP2R2B;PPP2R2B;PPP2R2B;P	TSS200;5'UTR;Body;5'UTR;TSS2	chr5:146257346-146258575	Island
hyper84	FGR_insufficient_2	1.4E-03	0.22	0.08	0.14	5	146258522	cg15927927	PPP2R2B;PPP2R2B;PPP2R2B;P	TSS200;5'UTR;Body;5'UTR;TSS2	chr5:146257346-146258575	Island
hyper85	FGR_insufficient_2	7.7E-06	0.26	0.08	0.18	5	146258546	cg25021259	PPP2R2B;PPP2R2B;PPP2R2B;P	5'UTR;Body;5'UTR;TSS1500;TSS	chr5:146257346-146258575	Island
hyper86	FGR_insufficient_2	6.3E-03	0.21	0.09	0.12	5	146258615	cg13983063	PPP2R2B;PPP2R2B;PPP2R2B;P	5'UTR;Body;5'UTR;TSS1500;TSS	chr5:146257346-146258575	S_Shore
hyper87	FGR_insufficient_2	2.6E-03	0.20	0.10	0.11	6	24495989	cg26943671	ALDH5A1;ALDH5A1	Body;Body	chr6:24494664-24495670	S_Shore
hyper88	FGR_insufficient_2	1.4E-02	0.13	0.05	0.08	6	26458326	cg01542644	BTN2A1;BTN2A1;BTN2A1;BTN2A	5'UTR;1stExon;1stExon;5'UTR	chr6:26458310-26458534	Island
hyper89	FGR_insufficient_2	1.1E-02	0.30	0.08	0.22	6	32821090	cg19776032	PSMB9;PSMB9;TAP1	TSS1500;TSS1500;1stExon	chr6:32820849-32822370	Island
hyper90	FGR_insufficient_2	4.2E-02	0.32	0.11	0.21	6	32821111	cg01897517	PSMB9;PSMB9;TAP1	TSS1500;TSS1500;1stExon	chr6:32820849-32822370	Island
hyper91	FGR_insufficient_2	1.6E-04	0.14	0.03	0.11	6	32821289	cg11012835	PSMB9;PSMB9;TAP1	TSS1500;TSS1500;1stExon	chr6:32820849-32822370	Island
hyper92	FGR_insufficient_2	9.7E-11	0.19	0.01	0.18	6	32821299	cg23922920	PSMB9;PSMB9;TAP1	TSS1500;TSS1500;1stExon	chr6:32820849-32822370	Island
hyper93	FGR_insufficient_2	7.8E-02	0.22	0.09	0.14	6	33589418	cg16400825	ITPR3	1stExon	chr6:33588617-33589909	Island
hyper94	FGR_insufficient_2	2.9E-02	0.34	0.08	0.26	6	75794995	cg11353250	COL12A1;COL12A1	3'UTR;3'UTR	chr6:75794880-75795088	Island
hyper95	FGR_insufficient_2	2.9E-10	0.25	0.06	0.19	7	66309627	cg10663655	LOC729156	Body	chr7:66309503-66310040	Island
hyper96	FGR_insufficient_2	0.0E+00	0.24	0.06	0.18	7	66309823	cg25610234	LOC729156	TSS200	chr7:66309503-66310040	Island
hyper97	FGR_insufficient_2	0.0E+00	0.25	0.02	0.23	7	66309851	cg06828852	LOC729156	TSS200	chr7:66309503-66310040	Island
hyper98	FGR_insufficient_2	0.0E+00	0.26	0.04	0.21	7	66309966	cg06061069	LOC729156	TSS200	chr7:66309503-66310040	Island
hyper99	FGR_insufficient_2	0.0E+00	0.42	0.06	0.36	7	66309972	cg07880104	LOC729156	TSS200	chr7:66309503-66310040	Island
hyper100	FGR_insufficient_2	5.6E-02	0.23	0.05	0.18	7	93519473	cg12973591	TFPI2	Body	chr7:93519366-93520184	Island
hyper101	FGR_insufficient_2	1.3E-03	0.14	0.05	0.09	8	38243683	cg14336578	LETM2	TSS1500	chr8:38243741-38244695	N_Shore
hyper102	FGR_insufficient_2	8.7E-02	0.16	0.03	0.13	8	41755140	cg01616178	ANK1	TSS1500	chr8:41753340-41755250	Island
hyper103	FGR_insufficient_2	5.2E-02	0.15	0.04	0.11	8	144242106	cg24971209	LY6H;LY6H;LY6H	TSS1500;TSS200;TSS1500	chr8:144240761-144242113	Island
hyper104	FGR_insufficient_2	4.0E-03	0.10	0.05	0.06	9	123837845	cg13822008			chr9:123836702-123837585	S_Shore
hyper105	FGR_insufficient_2	3.4E-02	0.16	0.09	0.07	9	124991047	cg21237939	LHX6;LHX6	TSS200;TSS200	chr9:124987743-124991086	Island
hyper106	FGR_insufficient_2	1.9E-10	0.36	0.06	0.29	9	131038152	cg13942909	C9orf119;GOLGA2	TSS1500;Body	chr9:131037591-131038695	Island
hyper107	FGR_insufficient_2	9.9E-03	0.17	0.07	0.11	10	102495141	cg22964842			chr10:102495116-102495613	Island
hyper108	FGR_insufficient_2	0.0E+00	0.41	0.04	0.37	10	103990984	cg05762694	PITX3	Body	chr10:103990213-103991829	Island
hyper109	FGR_insufficient_2	1.6E-08	0.49	0.07	0.42	10	103991057	cg07754999	PITX3	Body	chr10:103990213-103991829	Island
hyper110	FGR_insufficient_2	9.6E-05	0.21	0.03	0.18	10	103991299	cg00630724	PITX3	Body	chr10:103990213-103991829	Island
hyper111	FGR_insufficient_2	0.0E+00	0.32	0.05	0.27	10	103991465	cg20641465	PITX3	Body	chr10:103990213-103991829	Island
hyper112	FGR_insufficient_2	2.2E-04	0.19	0.08	0.11	10	103991777	cg08129312	PITX3	Body	chr10:103990213-103991829	Island
hyper113	FGR_insufficient_2	1.1E-04	0.15	0.06	0.09	11	112151121	cg10001219				
hyper114	FGR_insufficient_2	4.8E-03	0.15	0.08	0.07	11	118122801	cg23873021	MPZL3	Body		
hyper115	FGR_insufficient_2	3.9E-07	0.30	0.05	0.25	12	52401214	cg00817367	GRASP	Body	chr12:52400467-52401696	Island
hyper116	FGR_insufficient_2	1.1E-02	0.19	0.05	0.14	12	106977781	cg26732691	RFX4	Body	chr12:106979429-106981086	N_Shore
hyper117	FGR_insufficient_2	4.4E-02	0.05	0.01	0.04	12	107712120	cg09652427	BTBD11	TSS200	chr12:107711603-107714107	Island
hyper118	FGR_insufficient_2	4.7E-02	0.18	0.08	0.10	13	114898440	cg11320418	RASA3	TSS1500	chr13:114897358-114898516	Island
hyper119	FGR_insufficient_2	1.5E-03	0.67	0.15	0.52	15	27018037	cg18026588	GABRB3;GABRB3	1stExon;Body	chr15:27016892-27018943	Island
hyper120	FGR_insufficient_2	8.4E-05	0.12	0.04	0.07	15	56757108	cg24200809	MNS1	Body	chr15:56757179-56757383	N_Shore
hyper121	FGR_insufficient_2	6.8E-08	0.17	0.07	0.10	15	56757382	cg18864479	MNS1	TSS200	chr15:56757179-56757383	Island
hyper122	FGR_insufficient_2	1.1E-03	0.14	0.04	0.10	15	56757499	cg22674884	MNS1	TSS200	chr15:56757179-56757383	S_Shore
hyper123	FGR_insufficient_2	3.2E-03	0.12	0.04	0.08	15	56757517	cg07581460	MNS1	TSS200	chr15:56757179-56757383	S_Shore
hyper124	FGR_insufficient_2	3.0E-04	0.17	0.08	0.09	16	2198777	cg08813207	RAB26;RAB26	1stExon;5'UTR	chr16:2198455-2199129	Island

hyper125	FGR_insufficient_2	1.5E-02	0.22	0.12	0.09	16	2198781	cg26112564	RAB26;RAB26	1stExon;5'UTR	chr16:2198455-2199129	Island
hyper126	FGR_insufficient_2	1.9E-10	0.15	0.01	0.14	16	28074384	cg03394150	GSG1L	Body	chr16:28073817-28075296	Island
hyper127	FGR_insufficient_2	5.8E-02	0.06	0.02	0.04	16	28074462	cg10471437	GSG1L	1stExon	chr16:28073817-28075296	Island
hyper128	FGR_insufficient_2	5.1E-02	0.09	0.03	0.07	16	52580987	cg26817217	TOX3;TOX3	5'UTR;TSS200	chr16:52581293-52581610	N_Shore
hyper129	FGR_insufficient_2	5.0E-04	0.17	0.01	0.16	16	56623390	cg08270206	MT3;MT3	5'UTR;1stExon	chr16:56623068-56623914	Island
hyper130	FGR_insufficient_2	3.2E-04	0.09	0.02	0.06	17	19648414	cg19435033	ALDH3A1;ALDH3A1;ALDH3A1	Body;Body;1stExon	chr17:19648139-19648492	Island
hyper131	FGR_insufficient_2	2.0E-02	0.24	0.11	0.13	17	79859932	cg22700854	NPB	TSS200	chr17:79859808-79860963	Island
hyper132	FGR_insufficient_2	8.2E-03	0.31	0.19	0.12	17	79859945	cg20081244	NPB	TSS200	chr17:79859808-79860963	Island
hyper133	FGR_insufficient_2	2.9E-05	0.25	0.07	0.18	17	79859947	cg08983961	NPB	TSS200	chr17:79859808-79860963	Island
hyper134	FGR_insufficient_2	1.4E-02	0.16	0.04	0.11	19	4247036	cg11395893	CCDC94	TSS200	chr19:4246828-4247222	Island
hyper135	FGR_insufficient_2	2.3E-02	0.22	0.10	0.12	19	31839734	cg06196755	TSHZ3	Body	chr19:31839635-31843049	Island
hyper136	FGR_insufficient_2	1.3E-04	0.13	0.04	0.09	19	31841391	cg00545804	TSHZ3	TSS1500	chr19:31839635-31843049	Island
hyper137	FGR_insufficient_2	4.1E-05	0.26	0.07	0.19	19	44302906	cg20110633	LYPD5;LYPD5	Body;Body	chr19:44302665-44303176	Island
hyper138	FGR_insufficient_2	0.0E+00	0.22	0.02	0.20	19	44303058	cg16900980	LYPD5;LYPD5	Body;Body	chr19:44302665-44303176	Island
hyper139	FGR_insufficient_2	3.6E-09	0.18	0.07	0.11	19	44405818	cg13933427			chr19:44405817-44406053	Island
hyper140	FGR_insufficient_2	4.7E-04	0.17	0.07	0.09	19	44529414	cg20254725	ZNF222;ZNF222	TSS200;TSS200	chr19:44529393-44529778	Island
hyper141	FGR_insufficient_2	1.6E-03	0.14	0.01	0.13	19	54599221	cg11196851	OSCAR;OSCAR;OSCAR;OSCAR	Body;Body;Body;Body	chr19:54599160-54599456	Island
hyper142	FGR_insufficient_2	8.1E-02	0.19	0.02	0.18	19	54599285	cg05606351	OSCAR;OSCAR;OSCAR;OSCAR	Body;Body;Body;Body	chr19:54599160-54599456	Island
hyper143	FGR_insufficient_2	3.4E-02	0.05	0.01	0.04	19	55953996	cg12137286	SHISA7	1stExon	chr19:55953371-55954641	Island
hyper144	FGR_insufficient_2	0.0E+00	0.34	0.03	0.31	19	56915522	cg02776314	ZNF583;ZNF583;ZNF583	5'UTR;TSS200;TSS200	chr19:56915356-56915856	Island
hyper145	FGR_insufficient_2	1.0E-07	0.28	0.08	0.20	19	56915650	cg00796360	ZNF583;ZNF583;ZNF583	5'UTR;TSS200;TSS200	chr19:56915356-56915856	Island
hyper146	FGR_insufficient_2	1.4E-09	0.27	0.08	0.18	19	56915653	cg24119079	ZNF583;ZNF583;ZNF583	5'UTR;TSS200;TSS200	chr19:56915356-56915856	Island
hyper147	FGR_insufficient_2	0.0E+00	0.28	0.01	0.27	19	56915655	cg03280624	ZNF583;ZNF583;ZNF583	5'UTR;TSS200;TSS200	chr19:56915356-56915856	Island
hyper148	FGR_insufficient_2	0.0E+00	0.27	0.03	0.24	19	56915658	cg27009208	ZNF583;ZNF583;ZNF583	5'UTR;TSS200;TSS200	chr19:56915356-56915856	Island
hyper149	FGR_insufficient_2	9.7E-11	0.45	0.06	0.39	19	56915732	cg01873234	ZNF583;ZNF583;ZNF583;ZNF583	1stExon;1stExon;5'UTR;5'UTR;5'U	chr19:56915356-56915856	Island
hyper150	FGR_insufficient_2	1.9E-03	0.21	0.02	0.19	20	13200931	cg04432319	ISM1	TSS1500	chr20:13200670-13202616	Island
hyper151	FGR_insufficient_2	1.2E-09	0.16	0.01	0.15	20	13200954	cg12664209	ISM1	TSS1500	chr20:13200670-13202616	Island
hyper152	FGR_insufficient_2	3.3E-05	0.18	0.05	0.13	20	13200973	cg06262436	ISM1	TSS1500	chr20:13200670-13202616	Island
hyper153	FGR_insufficient_2	4.3E-09	0.15	0.01	0.14	20	13200980	cg09491991	ISM1	TSS1500	chr20:13200670-13202616	Island
hyper154	FGR_insufficient_2	5.8E-09	0.09	0.01	0.08	20	13200982	cg26322248	ISM1	TSS1500	chr20:13200670-13202616	Island
hyper155	FGR_insufficient_2	6.9E-05	0.19	0.04	0.15	21	34442360	cg17016394	OLIG1	TSS200	chr21:34442206-34444543	Island
hyper156	FGR_insufficient_2	9.4E-09	0.11	0.01	0.10	21	34442511	cg05720454	OLIG1;OLIG1	1stExon;5'UTR	chr21:34442206-34444543	Island
hyper157	FGR_insufficient_2	3.9E-04	0.18	0.09	0.09	21	34442674	cg02736969	OLIG1	1stExon	chr21:34442206-34444543	Island
hyper158	FGR_insufficient_2	2.1E-07	0.21	0.06	0.15	21	34443010	cg03732762	OLIG1	1stExon	chr21:34442206-34444543	Island
hyper159	FGR_insufficient_2	4.0E-02	0.16	0.07	0.08	22	36236395	cg12797594	RBM9;RBM9;RBM9;RBM9;RBM9;	5'UTR;Body;Body;5'UTR;5'UTR;1stExon;1stExon;1stExon;5'UTR;1stExon		
hyper160	FGR_insufficient_3	2.5E-02	0.28	0.10	0.18	1	1950616	cg09107007	GABRD	TSS200	chr1:1950411-1951479	Island
hyper161	FGR_insufficient_3	2.7E-04	0.21	0.07	0.14	1	15417078	cg00866953	KIAA1026	Body		
hyper162	FGR_insufficient_3	3.3E-03	0.17	0.07	0.10	1	36772064	cg03506609	C1orf113;C1orf113	TSS1500;Body	chr1:36771830-36773009	Island
hyper163	FGR_insufficient_3	1.5E-06	0.17	0.02	0.16	1	36772095	cg13446200	C1orf113;C1orf113	TSS1500;Body	chr1:36771830-36773009	Island
hyper164	FGR_insufficient_3	1.5E-04	0.21	0.06	0.15	1	36772417	cg05063806	C1orf113;C1orf113	TSS1500;Body	chr1:36771830-36773009	Island
hyper165	FGR_insufficient_3	1.2E-02	0.19	0.03	0.16	1	115632576	cg17131030	TSPAN2	TSS1500	chr1:115631850-115632584	Island
hyper166	FGR_insufficient_3	1.7E-02	0.19	0.03	0.16	1	166134282	cg21868774	FAM78B	Body	chr1:166134258-166136448	Island
hyper167	FGR_insufficient_3	3.9E-02	0.17	0.07	0.09	1	182360697	cg12535596	GLUL;GLUL;GLUL	5'UTR;TSS200;5'UTR	chr1:182359697-182361563	Island
hyper168	FGR_insufficient_3	1.1E-07	0.20	0.08	0.12	1	209921370	cg06541795			chr1:209920953-209921345	S_Shore
hyper169	FGR_insufficient_3	2.6E-02	0.05	0.01	0.04	1	225965400	cg08090133	SRP9;SRP9	TSS200;TSS200	chr1:225965330-225965734	Island
hyper170	FGR_insufficient_3	3.6E-04	0.17	0.02	0.14	2	263656	cg13354988	ACP1;ACP1;SH3YL1;ACP1;SH3Y	TSS1500;TSS1500;Body;TSS150	chr2:263400-265238	Island
hyper171	FGR_insufficient_3	1.1E-03	0.13	0.07	0.06	2	14775132	cg11533098	FAM84A	3'UTR	chr2:14772377-14775809	Island
hyper172	FGR_insufficient_3	0.0E+00	0.36	0.06	0.29	3	112931390	cg13765303	BOC;BOC	1stExon;5'UTR	chr3:112930437-112931506	Island
hyper173	FGR_insufficient_3	3.9E-02	0.57	0.33	0.25	3	165636307	cg15486124				
hyper174	FGR_insufficient_3	6.1E-07	0.23	0.07	0.16	3	197183416	cg26048523			chr3:197183404-197183724	Island
hyper175	FGR_insufficient_3	0.0E+00	0.30	0.02	0.27	3	197183582	cg25221442			chr3:197183404-197183724	Island
hyper176	FGR_insufficient_3	3.9E-10	0.31	0.03	0.27	3	197183662	cg11302533			chr3:197183404-197183724	Island
hyper177	FGR_insufficient_3	1.4E-05	0.28	0.09	0.19	4	4874548	cg17684202			chr4:4873262-4873613	S_Shore
hyper178	FGR_insufficient_3	4.1E-02	0.19	0.09	0.10	4	48271107	cg17566936	TEC	5'UTR	chr4:48271362-48271846	N_Shore
hyper179	FGR_insufficient_3	1.6E-02	0.05	0.01	0.03	5	68485104	cg05567409	CENPH	TSS1500	chr5:68485215-68485577	N_Shore
hyper180	FGR_insufficient_3	2.2E-06	0.21	0.05	0.17	5	167000712	cg22554488	ODZ2	Body		
hyper181	FGR_insufficient_3	1.4E-04	0.31	0.03	0.28	5	178487123	cg08522107	ZNF354C	TSS1500	chr5:178487146-178487921	N_Shore
hyper182	FGR_insufficient_3	6.2E-02	0.39	0.02	0.37	5	178487310	cg19761115	ZNF354C	TSS1500	chr5:178487146-178487921	Island
hyper183	FGR_insufficient_3	4.7E-04	0.40	0.02	0.38	5	178487384	cg12623648	ZNF354C	TSS1500	chr5:178487146-178487921	Island
hyper184	FGR_insufficient_3	4.6E-05	0.25	0.05	0.20	5	178487465	cg07470489	ZNF354C	TSS200	chr5:178487146-178487921	Island
hyper185	FGR_insufficient_3	1.6E-04	0.28	0.04	0.24	5	178487481	cg11538128	ZNF354C	TSS200	chr5:178487146-178487921	Island
hyper186	FGR_insufficient_3	2.1E-05	0.30	0.05	0.24	5	178487485	cg08278489	ZNF354C	TSS200	chr5:178487146-178487921	Island
hyper187	FGR_insufficient_3	4.7E-03	0.35	0.07	0.28	5	178487500	cg10802132	ZNF354C	TSS200	chr5:178487146-178487921	Island
hyper188	FGR_insufficient_3	2.5E-02	0.37	0.06	0.31	5	178487576	cg07515940	ZNF354C	TSS200	chr5:178487146-178487921	Island
hyper189	FGR_insufficient_3	1.3E-05	0.06	0.01	0.05	6	13814565	cg18831738	CCDC90A	1stExon	chr6:13813943-13814946	Island
hyper190	FGR_insufficient_3	0.0E+00	0.31	0.03	0.28	7	12250161	cg06286300	TMEM106B;TMEM106B	TSS1500;TSS1500	chr7:12250720-12251214	N_Shore
hyper191	FGR_insufficient_3	0.0E+00	0.34	0.02	0.32	7	12250517	cg24949795	TMEM106B;TMEM106B	TSS1500;TSS1500	chr7:12250720-12251214	N_Shore
hyper192	FGR_insufficient_3	0.0E+00	0.24	0.01	0.23	7	12251200	cg27479937	TMEM106B;TMEM106B	5'UTR;5'UTR	chr7:12250720-12251214	Island
hyper193	FGR_insufficient_3	0.0E+00	0.38	0.05	0.33	7	12251342	cg21597649	TMEM106B;TMEM106B	5'UTR;5'UTR	chr7:12250720-12251214	S_Shore
hyper194	FGR_insufficient_3	1.8E-06	0.17	0.06	0.11	7	14028845	cg16805994	ETV1;ETV1;ETV1;ETV1	5'UTR;5'UTR;5'UTR;5'UTR	chr7:14029378-14029593	N_Shore
hyper195	FGR_insufficient_3	7.2E-05	0.11	0.04	0.07	7	14028954	cg21249126	ETV1;ETV1;ETV1;ETV1	5'UTR;5'UTR;5'UTR;5'UTR	chr7:14029378-14029593	N_Shore
hyper196	FGR_insufficient_3	4.4E-08	0.38	0.08	0.30	7	14029015	cg03932308	ETV1;ETV1;ETV1;ETV1	5'UTR;5'UTR;5'UTR;5'UTR	chr7:14029378-14029593	N_Shore
hyper197	FGR_insufficient_3	3.1E-05	0.22	0.07	0.15	7	14029057	cg07323919	ETV1;ETV1;ETV1;ETV1	5'UTR;5'UTR;5'UTR;5'UTR	chr7:14029378-14029593	N_Shore
hyper198	FGR_insufficient_3	0.0E+00	0.29	0.07	0.22	7	14029085	cg06154860	ETV1;ETV1;ETV1;ETV1	5'UTR;5'UTR;5'UTR;5'UTR	chr7:14029378-14029593	N_Shore
hyper199	FGR_insufficient_3	2.2E-08	0.18	0.05	0.14	7	14029285	cg03166779	ETV1;ETV1;ETV1;ETV1;ETV1;ET	1stExon;5'UTR;5'UTR;5'UTR;1stE	chr7:14029378-14029593	N_Shore
hyper200	FGR_insufficient_3	8.3E-04	0.34	0.08	0.25	7	14029514	cg11104510	ETV1;ETV1;ETV1;ETV1;ETV1;ET	1stExon;5'UTR;5'UTR;5'UTR;1stE	chr7:14029378-14029593	Island
hyper201	FGR_insufficient_3	1.6E-05	0.24	0.07	0.17	7	42927968	cg00587042				
hyper202	FGR_insufficient_3	1.3E-05	0.42	0.05	0.37	7	42928245	cg16168014				
hyper203	FGR_insufficient_3	5.5E-02	0.08	0.01	0.06	7	44365536	cg16073349	CAMK2B;CAMK2B;CAMK2B;CAM	TSS1500;TSS1500;TSS1500;TSS	chr7:44364432-44365328	S_Shore
hyper204	FGR_insufficient_3	4.4E-02	0.27	0.11	0.16	7	101398152	cg14823389				
hyper205	FGR_insufficient_3	3.0E-04	0.27	0.09	0.18	7	101398177	cg22114489				
hyper206	FGR_insufficient_3	7.4E-08	0.43	0.11	0.32	7	142960387	cg17524624	GSTK1;GSTK1;GSTK1;GSTK1	TSS200;TSS200;TSS200;TSS200		
hyper207	FGR_insufficient_3	1.9E-10	0.32	0.06	0.26	8	67686767	cg15821835	SGK3;SGK3;SGK3	TSS1500;TSS1500;5'UTR	chr8:67687305-67687787	N_Shore
hyper208	FGR_insufficient_3	0.0E+00	0.36	0.04	0.33	8	67686852	cg21180685	SGK3;SGK3;SGK3	TSS1500;TSS1500;5'UTR	chr8:67687305-67687787	N_Shore
hyper209	FGR_insufficient_3	0.0E+00	0.37	0.07	0.30	8	67686871	cg07554251	SGK3;SGK3;SGK3	TSS1500;TSS1500;5'UTR	chr8:67687305-67687787	N_Shore
hyper210	FGR_insufficient_3	0.0E+00	0.30	0.04	0.26	8	67686942	cg10360928	SGK3;SGK3;SGK3	TSS1500;TSS1500;5'UTR	chr8:67687305-67687787	N_Shore
hyper211	FGR_insufficient_3	0.0E+00	0.32	0.08	0.24	8	67687060	cg24483552	SGK3;SGK3;SGK3	TSS1500;TSS1500;5'UTR	chr8:67687305-67687787	N_Shore
hyper212	FGR_insufficient_3	0.0E+00	0.28	0.05	0.23	8	67687318	cg02490907	SGK3;SGK3;SGK3	TSS200;5'UTR;TSS200	chr8:67687305-67687787	Island
hyper213	FGR_insufficient_3	4.7E-04	0.19	0.06	0.13	8	67687371	cg10626718	SGK3;SGK3;SGK3	TSS200;5'UTR;TSS200	chr8:67687305-67687787	Island
hyper214	FGR_insufficient_3	0.0E+00	0.19	0.02	0.17	8	67687406	cg07138092	SGK3;SGK3;SGK3	TSS200;5'UTR;TSS200	chr8:67687305-67687787	Island
hyper215	FGR_insufficient_3	0.0E+00	0.31	0.04	0.27	8	67687437	cg14940705	SGK3;SGK3;SGK3	TSS200;5'UTR;TSS200	chr8:67687305-67687787	Island
hyper216	FGR_insufficient_3	0.0E+00	0.45	0.05	0.41	8	67687455	cg25482853	SGK3;SGK3;SGK3	TSS200;5'UTR;TSS200	chr8:67687305-67687787	Island
hyper217	FGR_insufficient_3	0.0E+00	0.49	0.02	0.47	8	67687474	cg10891996	SGK3;SGK3;SGK3;SGK3;SGK3	1stExon;5'UTR;5'UTR;1stExon;5'U	chr8:67687305-67687787	Island
hyper218	FGR_insufficient_3	1.2E-06	0.43	0.04	0.40	8	79674645	cg11931596	IL7	Body		
hyper219	FGR_insufficient_3	6.9E-02	0.20	0.08	0.12	8	124408541	cg18604888	ATAD2	1stExon	chr8:124408092-124409038	Island
hyper220	FGR_insufficient_3	1.1E-07	0.30	0.08	0.22	8	145047469	cg00575153	PLEC1;PLEC1	Body;Body	chr8:145047293-145052305	Island
hyper221	FGR_insufficient_3	1.7E-03	0.18	0.09	0.09	8	145047566	cg15183991	PLEC1;PLEC1	Body;Body	chr8:145047293-145052305	Island
hyper222	FGR_insufficient_3	4.6E-02	0.13	0.05	0.08	10	89621789	cg04616691	PTEN;KILLIN	TSS1500;1stExon	chr10:89621772-89624128	Island
hyper223	FGR_insufficient_3	4.4E-02	0.12	0.07	0.06	10	89621822	cg08995089	PTEN;KILLIN	TSS1500;1stExon	chr10:89621772-89624128	Island
hyper224	FGR_insufficient_3	1.8E-08	0.20	0.05	0.15	10	97055150	cg22665099			chr10:97055128-97055448	Island
hyper225	FGR_insufficient_3	1.9E-08	0.18	0.08	0.11	10	97055282	cg06823512			chr10:97055128-97055448	Island
hyper226	FGR_insufficient_3	1.9E-05	0.25	0.06	0.18	10	97055439	cg20276630			chr10:97055128-97055448	Island
hyper227	FGR_insufficient_3	3.3E-03	0.24	0.10	0.14	10	106034472	cg00899160	GSTO2	5'UTR	chr10:106034574-106034803	N_Shore
hyper228	FGR_insufficient_3	2.9E-05	0.27	0.09	0.17	10	106034655	cg19917656	GSTO2	5'UTR	chr10:106034574-106034803	Island
hyper229	FGR_insufficient_3	1.6E-06	0.23	0.11	0.13	10	106034687	cg13766030	GSTO2	Body	chr10:106034574-106034803	Island
hyper230	FGR_insufficient_3	4.8E-02	0.23	0.04	0.19	10	131988874	cg27491759			chr10:131988211-131989332	Island
hyper231	FGR_insufficient_3	6.2E-04	0.09	0.02	0.07	10	134901297	cg15825786	GPR123	TSS200	chr10:134901198-134902619	Island
hyper232	FGR_insufficient_3	2.6E-02	0.11	0.05	0.06	10	134901301	cg18029761	GPR123	TSS200	chr10:134901198-134902619	Island
hyper233	FGR_insufficient_3	9.0E-02	0.04	0.01	0.03	11	3818855	cg05755092	PGAP2;NUP98;PGAP2;PGAP2;N	TSS200;5'UTR;TSS200;TSS1500;	chr11:3818447-3819307	Island
hyper234	FGR_insufficient_3	1.4E-02	0.24	0.02	0.22	11	61062910	cg06282270	VWCE	TSS200	chr11:61061751-61063063	Island
hyper235	FGR_insufficient_3	8.2E-02	0.37	0.20	0.17	11	61115736	cg20242427	DAK	3'UTR		
hyper236	FGR_insufficient_3	3.1E-04	0.11	0.03	0.08	11	75379624	cg11694752	MAP6;MAP6	TSS200;TSS200	chr11:75378416-75379882	Island
hyper237	FGR_insufficient_3	6.2E-04	0.22	0.08	0.14	12	48513167	cg16224902	PFKM;PFKM;PFKM	Body;1stExon;5'UTR		
hyper238	FGR_insufficient_3	6.9E-06	0.10	0.02	0.08	12	53085125	cg16207330	KRT77	Body		
hyper239	FGR_insufficient_3	1.5E-04	0.37	0.09	0.28	14	75180524	cg21190982	FCF1;KIAA0317	Body;TSS1500	chr14:75179629-75179875	S_Shore
hyper240	FGR_insufficient_3	1.5E-02	0.40	0.13	0.27	14	75180534	cg06642626	FCF1;KIAA0317	Body;TSS1500	chr14:75179629-75179875	S_Shore
hyper241	FGR_insufficient_3	7.1E-02	0.25	0.08	0.17	14	91224592	cg07545587	TTC7B	Body	chr14:91224829-91225109	N_Shore
hyper242	FGR_insufficient_3	3.9E-04	0.14	0.05	0.09	14	91224978	cg12055302	TTC7B	Body	chr14:91224829-91225109	Island
hyper243	FGR_insufficient_3	2.4E-03	0.09	0.01	0.07	15	52043708	cg07307653	TMOD2;TMOD2;LYSMD2	TSS200;TSS200;TSS200	chr15:52043422-52044447	Island
hyper244	FGR_insufficient_3	2.9E-10	0.26	0.06	0.20	15	65360366	cg27522060	RASL12;RASL12	5'UTR;1stExon	chr15:65360024-65360542	Island
hyper245	FGR_insufficient_3	1.4E-05	0.42	0.16	0.25	15	65360370	cg10604168	RASL12;RASL12	5'UTR;1stExon	chr15:65360024-65360542	Island
hyper246	FGR_insufficient_3	3.0E-02	0.18	0.07	0.10	15	96868935	cg03053826	NR2F2	TSS1500	chr15:96864881-96866787	S_Shelf
hyper247	FGR_insufficient_3	4.5E-03	0.11	0.03	0.07	16	11836374	cg08460793	TXNDC11	1stExon	chr16:11836073-11837092	Island
hyper248	FGR_insufficient_3	1.4E-02	0.09	0.03	0.06	16	67189629	cg01961069	TRADD	Body	chr16:67188613-67189652	Island
hyper249	FGR_insufficient_3	9.5E-03	0.40	0.08	0.32	17	2757523	cg02146462	RAP1GAP2;RAP1GAP2	Body;Body	chr17:2756101-2757938	Island
hyper250	FGR_insufficient_3	2.1E-07	0.23	0.02	0.22	17	34077244	cg14527050	GAS2L2	Body		
hyper251	FGR_insufficient_3	3.2E-04	0.06	0.01	0.05	17	72733168	cg19445996	RAB37;RAB37;RAB37;RAB37	TSS200;1stExon;Body;TSS200	chr17:72732848-72733545	Island
hyper252	FGR_insufficient_3	4.0E-02	0.05	0.02	0.03	17	72733172	cg04028224	RAB37;RAB37;RAB37;RAB37	TSS200;1stExon;Body;TSS200	chr17:72732848-72733545	Island
hyper253	FGR_insufficient_3	1.6E-03	0.46	0.18	0.28	18	8367314	cg23295886	LOC100192426;PTPRM;PTPRM	TSS1500;Body;Body	chr18:8367273-8367599	Island

hyper254	FGR_insufficient_3	0.0E+00	0.38	0.03	0.34	18	8367375	cg02996413	LOC100192426;PTPRM;PTPRM	TSS1500;Body;Body	chr18:8367273-8367599	Island
hyper255	FGR_insufficient_3	1.9E-10	0.45	0.05	0.40	18	8367425	cg25705486	LOC100192426;PTPRM;PTPRM	TSS1500;Body;Body	chr18:8367273-8367599	Island
hyper256	FGR_insufficient_3	4.8E-02	0.21	0.12	0.09	19	11533199	cg24185443	CCDC151	Body	chr19:11533198-11533619	Island
hyper257	FGR_insufficient_3	6.0E-05	0.09	0.02	0.07	19	38924182	cg02026160	RYR1;RYR1	TSS200;TSS200	chr19:38924239-38924519	N_Shore
hyper258	FGR_insufficient_3	5.5E-08	0.19	0.08	0.12	19	41882232	cg17831002	TMEM91;TMEM91;TMEM91;TMEI	TSS1500;TSS1500;TSS1500;TSS	chr19:41882507-41882773	N_Shore
hyper259	FGR_insufficient_3	1.6E-09	0.15	0.05	0.10	19	41882234	cg12946214	TMEM91;TMEM91;TMEM91;TMEI	TSS1500;TSS1500;TSS1500;TSS	chr19:41882507-41882773	N_Shore
hyper260	FGR_insufficient_3	1.4E-03	0.15	0.01	0.14	19	55973320	cg27153400	ISOC2;ISOC2;ISOC2	TSS1500;TSS1500;TSS1500	chr19:55972697-55973442	Island
hyper261	FGR_insufficient_3	4.0E-02	0.17	0.03	0.15	19	55973338	cg22563390	ISOC2;ISOC2;ISOC2	TSS1500;TSS1500;TSS1500	chr19:55972697-55973442	Island
hyper262	FGR_insufficient_3	6.4E-02	0.06	0.02	0.04	19	57049777	cg25963041	ZFP28	TSS1500	chr19:57049675-57050646	Island
hyper263	FGR_insufficient_3	2.5E-04	0.07	0.02	0.05	22	19132003	cg00280761	DGCR14	Body	chr22:19131992-19132297	Island
hyper264	FGR_insufficient_3	8.6E-03	0.07	0.02	0.05	22	50247415	cg20911168	ZBED4	TSS200	chr22:50246590-50248143	Island
hyper265	FGR_insufficient_4	7.2E-05	0.23	0.07	0.16	1	50489319	cg16260421	AGBL4	Body	chr1:50489417-50489846	N_Shore
hyper266	FGR_insufficient_4	3.9E-02	0.30	0.04	0.25	1	50513661	cg19098763	ELAVL4	TSS200	chr1:50513644-50514320	Island
hyper267	FGR_insufficient_4	3.6E-04	0.70	0.28	0.42	1	55504971	cg14993491	PCSK9	TSS1500	chr1:55505060-55506015	N_Shore
hyper268	FGR_insufficient_4	2.8E-06	0.13	0.03	0.10	1	76540574	cg21526205	ST6GALNAC3;ST6GALNAC3	Body;Body	chr1:76540147-76540653	Island
hyper269	FGR_insufficient_4	4.0E-05	0.20	0.05	0.15	1	76540641	cg27538686	ST6GALNAC3;ST6GALNAC3	Body;Body	chr1:76540147-76540653	Island
hyper270	FGR_insufficient_4	7.0E-06	0.23	0.09	0.14	2	11294738	cg09450367	PQLC3	TSS1500	chr2:11295559-11296006	N_Shore
hyper271	FGR_insufficient_4	2.7E-02	0.23	0.04	0.18	2	17721803	cg08031368	VSNL1	TSS200	chr2:17721537-17722021	Island
hyper272	FGR_insufficient_4	1.4E-06	0.09	0.01	0.07	2	51259493	cg08008931	NRXN1;NRXN1;NRXN1;NRXN1	1stExon;5'UTR;1stExon;5'UTR	chr2:51254522-51255498	S_Shelf
hyper273	FGR_insufficient_4	1.3E-02	0.16	0.08	0.08	2	85197811	cg21474384	KCMF1	TSS1500	chr2:85197445-85199321	Island
hyper274	FGR_insufficient_4	0.0E+00	0.42	0.06	0.35	2	88469792	cg18490616	THNSL2	TSS200	chr2:88469691-88470386	Island
hyper275	FGR_insufficient_4	0.0E+00	0.31	0.05	0.26	2	88470173	cg07952391	THNSL2	5'UTR	chr2:88469691-88470386	Island
hyper276	FGR_insufficient_4	0.0E+00	0.27	0.10	0.17	2	220083306	cg23547073	ABCB6	1stExon	chr2:220082991-220084012	Island
hyper277	FGR_insufficient_4	0.0E+00	0.39	0.00	0.39	2	220083502	cg27486695	ABCB6;ABCB6	5'UTR;1stExon	chr2:220082991-220084012	Island
hyper278	FGR_insufficient_4	0.0E+00	0.23	0.04	0.19	2	220083865	cg26544277	ABCB6	TSS200	chr2:220082991-220084012	Island
hyper279	FGR_insufficient_4	5.8E-10	0.35	0.07	0.27	2	220083916	cg23153680	ABCB6	TSS1500	chr2:220082991-220084012	Island
hyper280	FGR_insufficient_4	0.0E+00	0.25	0.07	0.17	2	220083951	cg06716437	ABCB6	TSS1500	chr2:220082991-220084012	Island
hyper281	FGR_insufficient_4	3.5E-02	0.38	0.06	0.32	3	35680890	cg24632247			chr3:35680509-35681628	Island
hyper282	FGR_insufficient_4	8.2E-02	0.38	0.11	0.27	3	38538168	cg16788250	EXOG;EXOG	Body;Body	chr3:38537682-38538071	S_Shore
hyper283	FGR_insufficient_4	7.4E-05	0.27	0.09	0.19	3	131246020	cg01966816			chr3:131245721-131246021	Island
hyper284	FGR_insufficient_4	6.6E-03	0.10	0.03	0.07	3	184971699	cg03414569	EHHADH;EHHADH;EHHADH	5'UTR;Body;1stExon	chr3:184971641-184972002	Island
hyper285	FGR_insufficient_4	7.8E-10	0.10	0.01	0.09	3	184971844	cg02738081	EHHADH;EHHADH;EHHADH;EHI	5'UTR;1stExon;1stExon;5'UTR	chr3:184971641-184972002	Island
hyper286	FGR_insufficient_4	1.1E-02	0.14	0.02	0.12	4	41883608	cg16146177			chr4:41883492-41884570	Island
hyper287	FGR_insufficient_4	4.8E-06	0.40	0.04	0.36	4	140952109	cg27341708	MAML3	Body		
hyper288	FGR_insufficient_4	2.2E-03	0.09	0.02	0.08	6	29600583	cg14128584	GABBR1;GABBR1	5'UTR;5'UTR	chr6:29600192-29600661	Island
hyper289	FGR_insufficient_4	6.5E-02	0.17	0.05	0.12	7	8473457	cg00852549	NXPH1	TSS200	chr7:8473139-8475199	Island
hyper290	FGR_insufficient_4	3.0E-05	0.21	0.04	0.17	7	64712472	cg05841659			chr7:64712213-64712786	Island
hyper291	FGR_insufficient_4	4.0E-02	0.04	0.01	0.03	7	140714747	cg08522707	MRPS33;MRPS33;MRPS33	5'UTR;1stExon;TSS1500	chr7:140714478-140714943	Island
hyper292	FGR_insufficient_4	2.6E-06	0.16	0.02	0.14	8	12051240	cg21196034	FAM86B1;FAM86B1	Body;Body	chr8:12051096-12052607	Island
hyper293	FGR_insufficient_4	1.9E-04	0.21	0.08	0.13	8	30770121	cg00719143			chr8:30769264-30770122	Island
hyper294	FGR_insufficient_4	8.9E-02	0.30	0.09	0.21	9	977109	cg12420104	DMRT3	1stExon	chr9:976162-977828	Island
hyper295	FGR_insufficient_4	4.1E-03	0.07	0.02	0.05	10	74020976	cg12799885			chr10:74020773-74021023	Island
hyper296	FGR_insufficient_4	4.8E-02	0.25	0.09	0.16	10	129705132	cg14325851	PTPRE	TSS200	chr10:129705217-129706237	N_Shore
hyper297	FGR_insufficient_4	6.7E-04	0.26	0.11	0.14	10	129705158	cg00335169	PTPRE	TSS200	chr10:129705217-129706237	N_Shore
hyper298	FGR_insufficient_4	4.7E-09	0.25	0.09	0.16	10	129705196	cg20299263	PTPRE	TSS200	chr10:129705217-129706237	N_Shore
hyper299	FGR_insufficient_4	1.9E-10	0.31	0.11	0.21	10	129705218	cg16442044	PTPRE	TSS200	chr10:129705217-129706237	Island
hyper300	FGR_insufficient_4	1.3E-08	0.15	0.03	0.12	12	29936839	cg08729318	TMTC1	5'UTR	chr12:29935995-29937433	Island
hyper301	FGR_insufficient_4	0.0E+00	0.23	0.07	0.15	12	29936845	cg20140110	TMTC1	5'UTR	chr12:29935995-29937433	Island
hyper302	FGR_insufficient_4	4.1E-02	0.12	0.06	0.06	12	48690546	cg00201175				
hyper303	FGR_insufficient_4	5.8E-04	0.16	0.06	0.10	12	121533833	cg01556017			chr12:121533571-121533923	Island
hyper304	FGR_insufficient_4	1.9E-06	0.16	0.06	0.10	14	39639031	cg20553470	TRAPPC6B;TRAPPC6B	Body;Body	chr14:39639364-39639635	N_Shore
hyper305	FGR_insufficient_4	4.0E-04	0.26	0.10	0.16	14	62279702	cg10981009			chr14:62279476-62280019	Island
hyper306	FGR_insufficient_4	9.9E-03	0.08	0.03	0.05	16	3313947	cg23754665			chr16:3313946-3314789	Island
hyper307	FGR_insufficient_4	2.3E-02	0.20	0.10	0.10	16	4421654	cg02048412	VASN;CORO7	TSS200;Body	chr16:4421642-4422240	Island
hyper308	FGR_insufficient_4	1.9E-10	0.24	0.05	0.18	16	4422216	cg10102242	VASN;CORO7	5'UTR;Body	chr16:4421642-4422240	Island
hyper309	FGR_insufficient_4	1.1E-08	0.30	0.11	0.20	16	4422228	cg12071544	VASN;CORO7	5'UTR;Body	chr16:4421642-4422240	Island
hyper310	FGR_insufficient_4	7.9E-05	0.09	0.02	0.07	16	54962963	cg09293786			chr16:54962422-54967805	Island
hyper311	FGR_insufficient_4	3.9E-10	0.31	0.05	0.26	17	27139003	cg17900697	C17orf63;C17orf63	5'UTR;5'UTR	chr17:27139185-27139671	N_Shore
hyper312	FGR_insufficient_4	0.0E+00	0.40	0.03	0.37	17	27139292	cg09688486	C17orf63;C17orf63	5'UTR;5'UTR	chr17:27139185-27139671	Island
hyper313	FGR_insufficient_4	0.0E+00	0.43	0.07	0.35	17	27139569	cg17613452	C17orf63;C17orf63	5'UTR;5'UTR	chr17:27139185-27139671	Island
hyper314	FGR_insufficient_4	0.0E+00	0.39	0.04	0.35	17	27139688	cg00977744	C17orf63;C17orf63	5'UTR;5'UTR	chr17:27139185-27139671	S_Shore
hyper315	FGR_insufficient_4	0.0E+00	0.40	0.05	0.35	19	30019369	cg05984554	VSTM2B	Body	chr19:30015781-30021367	Island
hyper316	FGR_insufficient_4	0.0E+00	0.24	0.03	0.21	19	41770160	cg10177748	HNRNPUL1;HNRNPUL1;HNRNPI	5'UTR;1stExon;5'UTR	chr19:41770013-41771113	Island
hyper317	FGR_insufficient_4	1.4E-03	0.13	0.05	0.08	19	44439215	cg09466880			chr19:44439200-44439612	Island
hyper318	FGR_insufficient_4	4.7E-02	0.26	0.03	0.23	19	44529683	cg09922628	ZNF222;ZNF222	5'UTR;Body	chr19:44529393-44529778	Island
hyper319	FGR_insufficient_4	5.7E-04	0.06	0.02	0.04	19	46405944	cg20152630	MYPOP	TSS200	chr19:46404537-46406545	Island
hyper320	FGR_insufficient_4	2.2E-03	0.43	0.04	0.39	19	56904945	cg02763101	ZNF582	TSS200	chr19:56904636-56905355	Island
hyper321	FGR_insufficient_4	3.1E-03	0.35	0.08	0.27	19	58095424	cg02653557	ZIK1	TSS1500	chr19:58094739-58095764	Island
hyper322	FGR_insufficient_4	2.1E-05	0.26	0.02	0.25	19	58095468	cg04342092	ZIK1	TSS200	chr19:58094739-58095764	Island
hyper323	FGR_insufficient_4	4.1E-04	0.56	0.16	0.40	19	58095581	cg12060744	ZIK1	TSS200	chr19:58094739-58095764	Island
hyper324	FGR_insufficient_4	2.7E-04	0.54	0.11	0.43	19	58095588	cg01046104	ZIK1	TSS200	chr19:58094739-58095764	Island
hyper325	FGR_insufficient_5	5.3E-03	0.35	0.04	0.31	2	43903582	cg09132607	PLEKHH2;LOC728819	Body;TSS200		
hyper326	FGR_insufficient_5	3.1E-05	0.49	0.05	0.44	2	43903650	cg02111748	PLEKHH2;LOC728819	Body;TSS200		
hyper327	FGR_insufficient_5	1.9E-10	0.16	0.01	0.15	3	46734995	cg11816841	ALS2CL	5'UTR	chr3:46734731-46735717	Island
hyper328	FGR_insufficient_5	8.4E-03	0.12	0.04	0.08	3	46735152	cg23444101	ALS2CL;ALS2CL	1stExon;5'UTR	chr3:46734731-46735717	Island
hyper329	FGR_insufficient_5	0.0E+00	0.27	0.02	0.25	3	46735315	cg03270929	ALS2CL	TSS200	chr3:46734731-46735717	Island
hyper330	FGR_insufficient_5	5.1E-06	0.09	0.02	0.07	3	46735319	cg07279281	ALS2CL	TSS200	chr3:46734731-46735717	Island
hyper331	FGR_insufficient_5	1.9E-10	0.23	0.04	0.20	3	46735335	cg19455322	ALS2CL	TSS200	chr3:46734731-46735717	Island
hyper332	FGR_insufficient_5	1.5E-05	0.26	0.10	0.16	3	46735338	cg21379004	ALS2CL	TSS200	chr3:46734731-46735717	Island
hyper333	FGR_insufficient_5	5.4E-04	0.16	0.04	0.12	3	46735454	cg25104512	ALS2CL	TSS1500	chr3:46734731-46735717	Island
hyper334	FGR_insufficient_5	9.4E-04	0.12	0.01	0.11	3	114866927	cg20784525	ZBTB20	TSS1500	chr3:114865436-114867124	Island
hyper335	FGR_insufficient_5	3.0E-03	0.24	0.05	0.18	5	134879818	cg14410395			chr5:134879443-134879819	Island
hyper336	FGR_insufficient_5	5.3E-03	0.36	0.03	0.32	5	134879961	cg18193764			chr5:134879443-134879819	S_Shore
hyper337	FGR_insufficient_5	2.2E-02	0.36	0.10	0.25	5	134880580	cg25916993			chr5:134879443-134879819	S_Shore
hyper338	FGR_insufficient_5	4.6E-04	0.52	0.16	0.36	5	167474670	cg23072711	ODZ2	Body		
hyper339	FGR_insufficient_5	3.1E-04	0.09	0.03	0.07	9	8857502	cg13723825	PTPRD;PTPRD	5'UTR;5'UTR	chr9:8857485-8858708	Island
hyper340	FGR_insufficient_5	3.9E-02	0.05	0.01	0.04	12	64784252	cg19998368	C12orf56;C12orf56	1stExon;1stExon	chr12:64784010-64784664	Island
hyper341	FGR_insufficient_5	1.9E-10	0.23	0.01	0.21	15	52587913	cg18363538	MYO5C;MYO5C	1stExon;5'UTR	chr15:52587353-52588172	Island
hyper342	FGR_insufficient_5	0.0E+00	0.17	0.01	0.16	15	52588057	cg20793089	MYO5C	TSS200	chr15:52587353-52588172	Island
hyper343	FGR_insufficient_5	1.5E-04	0.14	0.04	0.09	15	52588127	cg24321039	MYO5C	TSS200	chr15:52587353-52588172	Island
hyper344	FGR_insufficient_5	1.4E-02	0.09	0.02	0.07	15	54051880	cg25644243	WDR72	TSS200	chr15:54051655-54052004	Island
hyper345	FGR_insufficient_5	0.0E+00	0.34	0.05	0.30	17	5323432	cg21018494	RPAIN;RPAIN;RPAIN;RPAIN;RP/	Body;1stExon;5'UTR;Body;5'UTR;	chr17:5322507-5323716	Island
hyper346	FGR_insufficient_5	5.8E-10	0.39	0.05	0.34	17	5323521	cg11829380	RPAIN;RPAIN;RPAIN;RPAIN;RP/	Body;1stExon;5'UTR;Body;5'UTR;	chr17:5322507-5323716	Island
hyper347	FGR_insufficient_5	0.0E+00	0.35	0.09	0.26	17	5323715	cg26894552	RPAIN;RPAIN;RPAIN;RPAIN;RP/	Body;Body;Body;Body;Body;Body;	chr17:5322507-5323716	Island
hyper348	FGR_insufficient_5	0.0E+00	0.28	0.06	0.22	17	5323805	cg15229153	RPAIN;RPAIN;RPAIN;RPAIN;RP/	Body;Body;Body;Body;Body;Body;	chr17:5322507-5323716	S_Shore
hyper349	FGR_insufficient_5	9.7E-10	0.47	0.08	0.39	19	22816896	cg08271804	ZNF492	TSS1500	chr19:22817274-22817546	N_Shore
hyper350	FGR_insufficient_5	8.8E-05	0.14	0.04	0.10	19	23578063	cg20103107	ZNF91	Body		
hyper351	FGR_insufficient_5	5.0E-02	0.13	0.05	0.08	19	23578260	cg17853707	ZNF91;ZNF91	1stExon;5'UTR		
hyper352	FGR_adequate_1	1.7E-02	0.09	0.02	0.07	2	114341680	cg03532167	WASH2P	Body	chr2:114341301-114342298	Island
hyper353	FGR_adequate_1	0.0E+00	0.38	0.09	0.30	2	200320455	cg03139053	SATB2	Body	chr2:200320502-200329681	N_Shore
hyper354	FGR_adequate_1	1.5E-02	0.22	0.08	0.14	4	6675611	cg19255853	LOC93622	TSS1500	chr4:6675691-6676407	N_Shore
hyper355	FGR_adequate_1	3.7E-04	0.21	0.06	0.15	4	6675619	cg21954109	LOC93622	TSS1500	chr4:6675691-6676407	N_Shore
hyper356	FGR_adequate_1	2.3E-07	0.10	0.01	0.09	4	6675674	cg20699200	LOC93622	TSS200	chr4:6675691-6676407	N_Shore
hyper357	FGR_adequate_1	2.7E-07	0.07	0.01	0.07	4	6675756	cg12283822	LOC93622	TSS200	chr4:6675691-6676407	Island
hyper358	FGR_adequate_1	1.0E-04	0.14	0.06	0.08	4	6675881	cg15397506	LOC93622	Body	chr4:6675691-6676407	Island
hyper359	FGR_adequate_1	7.1E-08	0.21	0.07	0.15	4	88343936	cg21298523	NUDT9;NUDT9;NUDT9	5'UTR;5'UTR;1stExon	chr4:88343677-88344159	Island
hyper360	FGR_adequate_1	0.0E+00	0.34	0.07	0.26	5	157158882	cg08953461	THG1L	Body	chr5:157158378-157158856	S_Shore
hyper361	FGR_adequate_1	6.8E-05	0.07	0.02	0.05	6	42928277	cg07941301	GNMT	TSS1500	chr6:42928218-42928810	Island
hyper362	FGR_adequate_1	3.2E-02	0.12	0.03	0.08	6	42928303	cg04013093	GNMT	TSS200	chr6:42928218-42928810	Island
hyper363	FGR_adequate_1	5.5E-04	0.13	0.05	0.08	6	42928475	cg13397359	GNMT	TSS200	chr6:42928218-42928810	Island
hyper364	FGR_adequate_1	1.3E-02	0.06	0.02	0.05	6	42928485	cg02353165	GNMT	TSS200	chr6:42928218-42928810	Island
hyper365	FGR_adequate_1	1.1E-06	0.17	0.07	0.11	6	42928495	cg24101359	GNMT	TSS200	chr6:42928218-42928810	Island
hyper366	FGR_adequate_1	2.9E-06	0.12	0.02	0.10	6	42928500	cg14338887	GNMT;GNMT	1stExon;5'UTR	chr6:42928218-42928810	Island
hyper367	FGR_adequate_1	8.3E-02	0.13	0.04	0.09	6	42928546	cg26578923	GNMT	1stExon	chr6:42928218-42928810	Island
hyper368	FGR_adequate_1	1.9E-10	0.31	0.02	0.29	7	102158209	cg11171224			chr7:102157823-102158510	Island
hyper369	FGR_adequate_1	1.4E-03	0.15	0.05	0.09	10	17685563	cg20444320	STAM	TSS1500	chr10:17686097-17686335	N_Shore
hyper370	FGR_adequate_1	0.0E+00	0.24	0.06	0.18	10	17685617	cg02997119	STAM	TSS1500	chr10:17686097-17686335	N_Shore
hyper371	FGR_adequate_1	2.3E-03	0.14	0.05	0.08	10	17685929	cg10053162	STAM	TSS200	chr10:17686097-17686335	N_Shore
hyper372	FGR_adequate_1	0.0E+00	0.18	0.04	0.14	10	17685950	cg23138461	STAM	TSS200	chr10:17686097-17686335	N_Shore
hyper373	FGR_adequate_1	0.0E+00	0.24	0.06	0.18	10	17686075	cg15660681	STAM	TSS200	chr10:17686097-17686335	N_Shore
hyper374	FGR_adequate_1	0.0E+00	0.29	0.04	0.25	10	17686107	cg13612989	STAM	TSS200	chr10:17686097-17686335	Island
hyper375	FGR_adequate_1	0.0E+00	0.37	0.07	0.30	10	17686119	cg13680864	STAM	TSS200	chr10:17686097-17686335	Island
hyper376	FGR_adequate_1	0.0E+00	0.25	0.07	0.18	10	17686274	cg18418503	STAM;STAM	5'UTR;1stExon	chr10:17686097-17686335	Island
hyper377	FGR_adequate_1	0.0E+00	0.28	0.06	0.22	10	17686414	cg19950823	STAM	Body	chr10:17686097-17686335	S_Shore
hyper378	FGR_adequate_1	1.9E-10	0.22	0.04	0.17	12	49211867	cg04152629	CACNB3	TSS1500	chr12:49212162-49212642	N_Shore
hyper379	FGR_adequate_1	7.1E-04	0.14	0.02	0.11	12	49212305	cg13285458	CACNB3	TSS1500	chr12:49212162-49212642	Island
hyper380	FGR_adequate_1	2.3E-07	0.19	0.08	0.11	12	49212475	cg00065408	CACNB3	TSS200	chr12:49212162-49212642	Island
hyper381	FGR_adequate_1	1.5E-07	0.27	0.03	0.24	14	102026953	cg01693026	DIO3;MIR1247	TSS1500;TSS200	chr14:102025989-102031567	Island
hyper382	FGR_adequate_1	0.0E+00	0.25	0.02	0.23	17	8055733	cg07592976	PER1;PER1	5'UTR;1stExon	chr17:8054550-8055835	Island

hyper383	FGR_adequate_1	0.0E+00	0.22	0.01	0.20	17	8055756	cg23363911	PER1	TSS200	chr17:8054550-8055835	Island
hyper384	FGR_adequate_1	0.0E+00	0.23	0.08	0.15	17	8055831	cg14158601	PER1	TSS200	chr17:8054550-8055835	Island
hyper385	FGR_adequate_1	1.9E-10	0.25	0.08	0.17	17	8055834	cg21093368	PER1	TSS200	chr17:8054550-8055835	Island
hyper386	FGR_adequate_1	1.2E-02	0.17	0.09	0.07	17	8055884	cg20503576	PER1	TSS200	chr17:8054550-8055835	S_Shore
hyper387	FGR_adequate_1	1.7E-06	0.26	0.12	0.14	17	8055888	cg16545079	PER1	TSS200	chr17:8054550-8055835	S_Shore
hyper388	FGR_adequate_1	2.8E-03	0.21	0.08	0.13	17	41116355	cg20848987	AARSD1;AARSD1	Body;Body	chr17:41116349-41116551	Island
hyper389	FGR_adequate_1	3.2E-02	0.28	0.04	0.24	17	41116479	cg25104488	AARSD1;AARSD1	Body;Body	chr17:41116349-41116551	Island
hyper390	FGR_adequate_1	0.0E+00	0.25	0.02	0.23	17	41116483	cg18658096	AARSD1;AARSD1	Body;Body	chr17:41116349-41116551	Island
hyper391	FGR_adequate_1	1.8E-02	0.23	0.08	0.15	17	41116517	cg16143763	AARSD1;AARSD1	Body;Body	chr17:41116349-41116551	Island
hyper392	FGR_adequate_1	1.3E-03	0.14	0.06	0.08	18	28621490	cg11814235	DSC3;DSC3	Body;Body	chr18:28621489-28623117	Island
hyper393	FGR_adequate_1	3.9E-05	0.19	0.04	0.15	19	17448797	cg09793057	GTPBP3;GTPBP3;GTPBP3	Body;Body;Body	chr19:17448735-17449043	Island
hyper394	FGR_adequate_1	3.8E-04	0.08	0.01	0.06	22	19754125	cg01697719	TBX1;TBX1;TBX1	Body;Body;Body	chr22:19753312-19755013	Island
hyper395	FGR_adequate_1	2.4E-02	0.15	0.08	0.07	22	19754448	cg21507095	TBX1;TBX1;TBX1	Body;3'UTR;Body	chr22:19753312-19755013	Island
hyper396	FGR_adequate_2	1.9E-04	0.15	0.05	0.10	1	151119109	cg00427454	SEMA6C	TSS200	chr1:151118049-151119373	Island
hyper397	FGR_adequate_2	6.3E-05	0.30	0.12	0.17	1	162792246	cg10588211			chr1:162792176-162792574	Island
hyper398	FGR_adequate_2	1.2E-07	0.16	0.04	0.11	2	24307071	cg08714407	TP53I3;TP53I3	Body;1stExon	chr2:24307026-24307678	Island
hyper399	FGR_adequate_2	6.0E-05	0.21	0.07	0.14	2	24307515	cg00605408	TP53I3;TP53I3;TP53I3	1stExon;5'UTR;5'UTR	chr2:24307026-24307678	Island
hyper400	FGR_adequate_2	4.8E-02	0.19	0.10	0.10	2	24307741	cg20763553	TP53I3;TP53I3	TSS200;TSS200	chr2:24307026-24307678	S_Shore
hyper401	FGR_adequate_2	7.5E-04	0.26	0.07	0.19	4	118006405	cg08145625	TRAM1L1	1stExon	chr4:118006538-118006859	N_Shore
hyper402	FGR_adequate_2	3.4E-03	0.24	0.07	0.18	4	118006429	cg07038423	TRAM1L1	1stExon	chr4:118006538-118006859	N_Shore
hyper403	FGR_adequate_2	9.4E-03	0.34	0.02	0.32	4	118006619	cg21599230	TRAM1L1;TRAM1L1	1stExon;5'UTR	chr4:118006538-118006859	Island
hyper404	FGR_adequate_2	2.1E-03	0.33	0.03	0.30	4	118006747	cg24183909	TRAM1L1	TSS200	chr4:118006538-118006859	Island
hyper405	FGR_adequate_2	5.4E-03	0.42	0.06	0.36	4	118006750	cg07837556	TRAM1L1	TSS200	chr4:118006538-118006859	Island
hyper406	FGR_adequate_2	1.1E-07	0.35	0.03	0.32	4	118006808	cg05807991	TRAM1L1	TSS200	chr4:118006538-118006859	Island
hyper407	FGR_adequate_2	2.6E-08	0.26	0.02	0.24	4	118006812	cg11163975	TRAM1L1	TSS200	chr4:118006538-118006859	Island
hyper408	FGR_adequate_2	4.8E-06	0.31	0.08	0.23	4	118006825	cg10277927	TRAM1L1	TSS200	chr4:118006538-118006859	Island
hyper409	FGR_adequate_2	8.5E-02	0.19	0.07	0.12	4	118006832	cg01904183	TRAM1L1	TSS200	chr4:118006538-118006859	Island
hyper410	FGR_adequate_2	8.6E-02	0.04	0.01	0.03	4	129209112	cg13841734	PGRMC2	TSS200	chr4:129208297-129209780	Island
hyper411	FGR_adequate_2	6.0E-04	0.19	0.07	0.12	4	163085081	cg22161476	FSTL5;FSTL5;FSTL5;FSTL5;FSTI	1stExon;1stExon;5'UTR;5'UTR;1stExon;5'UTR		
hyper412	FGR_adequate_2	1.8E-03	0.15	0.08	0.07	4	163085311	cg18424393	FSTL5;FSTL5;FSTL5	TSS200;TSS200;TSS200		
hyper413	FGR_adequate_2	1.4E-09	0.30	0.12	0.18	4	163085318	cg14650167	FSTL5;FSTL5;FSTL5	TSS200;TSS200;TSS200		
hyper414	FGR_adequate_2	2.9E-06	0.19	0.07	0.12	4	163085342	cg18695917	FSTL5;FSTL5;FSTL5	TSS200;TSS200;TSS200		
hyper415	FGR_adequate_2	9.7E-10	0.27	0.06	0.21	4	163085348	cg03767310	FSTL5;FSTL5;FSTL5	TSS200;TSS200;TSS200		
hyper416	FGR_adequate_2	2.9E-10	0.24	0.06	0.18	4	163085403	cg26105150	FSTL5;FSTL5;FSTL5	TSS1500;TSS1500;TSS1500		
hyper417	FGR_adequate_2	9.7E-11	0.23	0.06	0.18	4	163085411	cg26883282	FSTL5;FSTL5;FSTL5	TSS1500;TSS1500;TSS1500		
hyper418	FGR_adequate_2	0.0E+00	0.23	0.06	0.17	4	163085663	cg01734786	FSTL5;FSTL5;FSTL5	TSS1500;TSS1500;TSS1500		
hyper419	FGR_adequate_2	0.0E+00	0.22	0.07	0.15	4	163085697	cg13808714	FSTL5;FSTL5;FSTL5	TSS1500;TSS1500;TSS1500		
hyper420	FGR_adequate_2	9.9E-05	0.12	0.03	0.09	5	1877135	cg18349911			chr5:1874907-1879032	Island
hyper421	FGR_adequate_2	1.0E-04	0.34	0.04	0.31	5	175793049	cg07352345	ARL10	Body	chr5:175792355-175793505	Island
hyper422	FGR_adequate_2	1.1E-02	0.09	0.01	0.08	8	1771561	cg04926329	ARHGEF10	TSS1500	chr8:1771361-1772760	Island
hyper423	FGR_adequate_2	1.0E-05	0.12	0.02	0.09	11	66824445	cg04420309	RHOD	1stExon	chr11:66824118-66824621	Island
hyper424	FGR_adequate_2	4.6E-02	0.43	0.04	0.39	12	11653600	cg06598091			chr12:11653232-11653775	Island
hyper425	FGR_adequate_2	1.7E-04	0.17	0.04	0.13	13	42846195	cg12578287	AKAP11	TSS200	chr13:42845853-42846807	Island
hyper426	FGR_adequate_2	8.1E-02	0.36	0.07	0.29	13	42846648	cg08791849	AKAP11	5'UTR	chr13:42845853-42846807	Island
hyper427	FGR_adequate_2	5.5E-07	0.19	0.05	0.14	15	41805530	cg27496526	LTK;LTK;LTK	Body;Body;Body	chr15:41803443-41805531	Island
hyper428	FGR_adequate_2	1.9E-03	0.38	0.17	0.21	17	27279763	cg08657247	PHF12;PHF12	TSS1500;TSS1500	chr17:27276219-27278877	S_Shore
hyper429	FGR_adequate_2	1.2E-06	0.17	0.05	0.12	21	34638868	cg17943175	IL10RB	Body	chr21:34638515-34639075	Island
hyper430	FGR_adequate_2	6.0E-05	0.14	0.06	0.08	21	34638971	cg17506742	IL10RB	Body	chr21:34638515-34639075	Island
hyper431	FGR_adequate_2	5.8E-02	0.07	0.03	0.05	21	34639113	cg14280424	IL10RB	Body	chr21:34638515-34639075	S_Shore
hyper432	FGR_adequate_3	0.0E+00	0.23	0.02	0.21	1	714526	cg04231937			chr1:713984-714547	Island
hyper433	FGR_adequate_3	6.6E-07	0.22	0.08	0.14	1	714565	cg04496485			chr1:713984-714547	S_Shore
hyper434	FGR_adequate_3	0.0E+00	0.17	0.01	0.15	1	22140859	cg17456100	LDLRAD2	Body	chr1:22140891-22141407	N_Shore
hyper435	FGR_adequate_3	2.8E-05	0.11	0.02	0.09	1	22141014	cg15467646	LDLRAD2	Body	chr1:22140891-22141407	Island
hyper436	FGR_adequate_3	0.0E+00	0.41	0.05	0.36	1	23894874	cg07422195			chr1:23894640-23895064	Island
hyper437	FGR_adequate_3	0.0E+00	0.29	0.02	0.27	1	23894902	cg04567466			chr1:23894640-23895064	Island
hyper438	FGR_adequate_3	4.3E-03	0.10	0.04	0.06	1	25943438	cg26890920	MAN1C1	TSS1500	chr1:25944099-25944772	N_Shore
hyper439	FGR_adequate_3	1.2E-09	0.22	0.08	0.15	1	35450601	cg14517250			chr1:35450649-35451037	N_Shore
hyper440	FGR_adequate_3	1.9E-10	0.38	0.10	0.28	1	35450650	cg26151000			chr1:35450649-35451037	Island
hyper441	FGR_adequate_3	0.0E+00	0.38	0.01	0.37	1	35450787	cg26792589			chr1:35450649-35451037	Island
hyper442	FGR_adequate_3	4.9E-08	0.27	0.09	0.17	1	35451172	cg10379872			chr1:35450649-35451037	S_Shore
hyper443	FGR_adequate_3	1.4E-03	0.40	0.07	0.32	1	52195547	cg22812770	OSBPL9;OSBPL9;OSBPL9;OSBF	5'UTR;1stExon;Body;5'UTR;Body;	chr1:52195028-52195821	Island
hyper444	FGR_adequate_3	6.5E-07	0.31	0.07	0.24	1	52195686	cg06327150	OSBPL9;OSBPL9;OSBPL9;OSBF	5'UTR;Body;Body;5'UTR;Body;Bo	chr1:52195028-52195821	Island
hyper445	FGR_adequate_3	8.6E-02	0.14	0.02	0.12	1	228196227	cg26332740	WNT3A	Body	chr1:228194096-228196512	Island
hyper446	FGR_adequate_3	0.0E+00	0.75	0.03	0.72	2	28614638	cg11133114	FOSL2	TSS1500	chr2:28612964-28616748	Island
hyper447	FGR_adequate_3	0.0E+00	0.33	0.04	0.28	2	227656248	cg19045733	IRS1	3'UTR	chr2:227655903-227656647	Island
hyper448	FGR_adequate_3	0.0E+00	0.44	0.06	0.38	2	227656261	cg07514207	IRS1	3'UTR	chr2:227655903-227656647	Island
hyper449	FGR_adequate_3	0.0E+00	0.34	0.07	0.27	2	227656408	cg02049847	IRS1	3'UTR	chr2:227655903-227656647	Island
hyper450	FGR_adequate_3	3.9E-10	0.34	0.05	0.29	2	227656417	cg08626939	IRS1	3'UTR	chr2:227655903-227656647	Island
hyper451	FGR_adequate_3	4.2E-02	0.07	0.03	0.04	2	237477186	cg12463851	CXCR7	TSS1500	chr2:237476311-237477263	Island
hyper452	FGR_adequate_3	5.8E-03	0.10	0.03	0.06	2	242673979	cg24430517	D2HGDH	TSS200	chr2:242673653-242675106	Island
hyper453	FGR_adequate_3	1.1E-05	0.33	0.12	0.20	3	40518595	cg22218536	ZNF619;ZNF619;ZNF619;ZNF619	TSS200;TSS200;TSS200;TSS200	chr3:40518517-40518848	Island
hyper454	FGR_adequate_3	2.3E-08	0.43	0.15	0.28	3	40518669	cg26132612	ZNF619;ZNF619;ZNF619;ZNF619	1stExon;Body;1stExon;5'UTR;5'U1	chr3:40518517-40518848	Island
hyper455	FGR_adequate_3	1.3E-09	0.33	0.05	0.27	3	40518742	cg00417297	ZNF619;ZNF619;ZNF619;ZNF619	1stExon;Body;1stExon;5'UTR;5'U1	chr3:40518517-40518848	Island
hyper456	FGR_adequate_3	4.3E-08	0.39	0.06	0.33	3	40518745	cg21379321	ZNF619;ZNF619;ZNF619;ZNF619	1stExon;Body;1stExon;5'UTR;5'U1	chr3:40518517-40518848	Island
hyper457	FGR_adequate_3	1.8E-03	0.18	0.08	0.10	3	43731973	cg14121103	ABHD5	TSS1500	chr3:43731998-43733108	N_Shore
hyper458	FGR_adequate_3	1.9E-03	0.16	0.06	0.10	3	43732102	cg19150852	ABHD5	TSS1500	chr3:43731998-43733108	Island
hyper459	FGR_adequate_3	2.5E-04	0.21	0.10	0.11	3	122745685	cg10084513	SEMA5B	5'UTR	chr3:122745183-122746986	Island
hyper460	FGR_adequate_3	1.9E-05	0.36	0.04	0.32	3	134369939	cg22889573	KY	TSS200	chr3:134369464-134370242	Island
hyper461	FGR_adequate_3	7.6E-04	0.19	0.04	0.14	3	134369974	cg00060320	KY	TSS200	chr3:134369464-134370242	Island
hyper462	FGR_adequate_3	1.9E-03	0.30	0.07	0.23	3	134369987	cg12307306	KY	TSS200	chr3:134369464-134370242	Island
hyper463	FGR_adequate_3	8.7E-03	0.21	0.09	0.13	3	134370046	cg24370755	KY	TSS200	chr3:134369464-134370242	Island
hyper464	FGR_adequate_3	2.7E-07	0.19	0.06	0.13	4	4544010	cg16107224	STX18	TSS1500	chr4:4543437-4544104	Island
hyper465	FGR_adequate_3	1.2E-04	0.14	0.04	0.10	4	4544016	cg14530801	STX18	TSS1500	chr4:4543437-4544104	Island
hyper466	FGR_adequate_3	9.7E-11	0.22	0.03	0.19	4	4544025	cg21433820	STX18	TSS1500	chr4:4543437-4544104	Island
hyper467	FGR_adequate_3	2.1E-04	0.27	0.05	0.22	4	11428822	cg07260927	HS3ST1	5'UTR	chr4:11429414-11429633	N_Shore
hyper468	FGR_adequate_3	8.0E-03	0.61	0.18	0.43	4	44451526	cg17790605	KCTD8	TSS1500	chr4:44449386-44451133	S_Shore
hyper469	FGR_adequate_3	7.0E-02	0.28	0.09	0.18	4	83957095	cg24137332	COPS4	Body		
hyper470	FGR_adequate_3	3.3E-03	0.20	0.09	0.12	4	111553161	cg19602081	PITX2;PITX2	Body;Body	chr4:111553079-111554210	Island
hyper471	FGR_adequate_3	3.1E-02	0.40	0.04	0.36	4	140656096	cg23847701	MAML3	Body	chr4:140655962-140657135	Island
hyper472	FGR_adequate_3	1.0E-03	0.28	0.08	0.21	4	140656407	cg01410531	MAML3	Body	chr4:140655962-140657135	Island
hyper473	FGR_adequate_3	2.8E-04	0.42	0.13	0.30	4	151500931	cg07904475	LRBA	Body	chr4:151500763-151501299	Island
hyper474	FGR_adequate_3	8.5E-06	0.50	0.10	0.40	4	151501035	cg03729251	LRBA	Body	chr4:151500763-151501299	Island
hyper475	FGR_adequate_3	1.7E-05	0.42	0.12	0.30	4	151501298	cg14266527	LRBA	Body	chr4:151500763-151501299	Island
hyper476	FGR_adequate_3	1.2E-02	0.51	0.04	0.48	5	1876337	cg14564616			chr5:1874907-1879032	Island
hyper477	FGR_adequate_3	8.7E-06	0.42	0.15	0.27	5	131991765	cg16097655			chr5:131991554-131992304	Island
hyper478	FGR_adequate_3	7.3E-09	0.25	0.02	0.23	5	131991993	cg23378989			chr5:131991554-131992304	Island
hyper479	FGR_adequate_3	4.3E-02	0.26	0.14	0.13	5	139017667	cg10495047			chr5:139017133-139017668	Island
hyper480	FGR_adequate_3	1.7E-02	0.13	0.07	0.06	5	172386259	cg22664253	LOC100268168;RPL26L1;LOC10(	Body;TSS200;TSS1500	chr5:172386298-172386542	N_Shore
hyper481	FGR_adequate_3	3.2E-09	0.82	0.45	0.37	5	178652557	cg05703112	ADAMTS2;ADAMTS2	Body;Body	chr5:178651051-178651328	S_Shore
hyper482	FGR_adequate_3	5.6E-05	0.34	0.11	0.24	6	28219524	cg10481571	ZKSCAN4	1stExon	chr6:28219523-28219745	Island
hyper483	FGR_adequate_3	1.1E-06	0.29	0.03	0.27	6	28219722	cg10464061	ZKSCAN4	1stExon	chr6:28219523-28219745	Island
hyper484	FGR_adequate_3	1.1E-06	0.29	0.05	0.24	6	28219744	cg22191873	ZKSCAN4	1stExon	chr6:28219523-28219745	Island
hyper485	FGR_adequate_3	8.4E-06	0.24	0.06	0.18	6	28220091	cg12561748	ZKSCAN4	TSS200	chr6:28219523-28219745	S_Shore
hyper486	FGR_adequate_3	2.6E-02	0.45	0.11	0.35	6	28220258	cg08014158	ZKSCAN4	TSS1500	chr6:28219523-28219745	S_Shore
hyper487	FGR_adequate_3	1.8E-05	0.27	0.10	0.18	6	28249142	cg10680411	PGBD1	TSS1500	chr6:28249166-28249568	N_Shore
hyper488	FGR_adequate_3	7.6E-04	0.32	0.13	0.20	6	28249205	cg09002836	PGBD1	TSS200	chr6:28249166-28249568	Island
hyper489	FGR_adequate_3	0.0E+00	0.24	0.02	0.22	6	28249221	cg11259833	PGBD1	TSS200	chr6:28249166-28249568	Island
hyper490	FGR_adequate_3	6.2E-06	0.23	0.06	0.16	6	28249251	cg00017002	PGBD1	TSS200	chr6:28249166-28249568	Island
hyper491	FGR_adequate_3	0.0E+00	0.30	0.04	0.26	6	28249271	cg09804108	PGBD1	TSS200	chr6:28249166-28249568	Island
hyper492	FGR_adequate_3	0.0E+00	0.15	0.01	0.13	6	28249428	cg06314658	PGBD1;PGBD1	1stExon;5'UTR	chr6:28249166-28249568	Island
hyper493	FGR_adequate_3	0.0E+00	0.30	0.06	0.24	6	28249545	cg20029652	PGBD1;PGBD1	1stExon;5'UTR	chr6:28249166-28249568	Island
hyper494	FGR_adequate_3	0.0E+00	0.35	0.07	0.28	6	28249704	cg11664467	PGBD1	5'UTR	chr6:28249166-28249568	S_Shore
hyper495	FGR_adequate_3	1.6E-03	0.05	0.01	0.04	6	30042985	cg03219282	RNF39;RNF39	Body;Body	chr6:30042918-30043500	Island
hyper496	FGR_adequate_3	1.0E-07	0.08	0.02	0.07	6	30658796	cg18650878	NRM	TSS200	chr6:30654392-30654934	S_Shelf
hyper497	FGR_adequate_3	6.2E-04	0.19	0.10	0.09	6	82461423	cg04399083	FAM46A	Body	chr6:82461133-82463805	Island
hyper498	FGR_adequate_3	2.0E-02	0.39	0.12	0.27	6	100441581	cg01189783	MCHR2;MCHR2	5'UTR;5'UTR	chr6:100441406-100442152	Island
hyper499	FGR_adequate_3	1.3E-02	0.08	0.02	0.05	7	94537870	cg09724492	PPP1R9A;PPP1R9A;PPP1R9A;PI	TSS1500;5'UTR;TSS1500;TSS15(	chr7:94536849-94537477	S_Shore
hyper500	FGR_adequate_3	5.3E-02	0.12	0.05	0.08	8	145911260	cg02610507			chr8:145909676-145912846	Island
hyper501	FGR_adequate_3	1.5E-05	0.11	0.05	0.07	9	34620091	cg22579849	DCTN3;DCTN3	Body;Body	chr9:34623512-34624081	N_Shelf
hyper502	FGR_adequate_3	3.0E-02	0.14	0.08	0.06	9	34620270	cg21159858	DCTN3;DCTN3	Body;Body	chr9:34623512-34624081	N_Shelf
hyper503	FGR_adequate_3	7.3E-03	0.14	0.07	0.07	9	124857534	cg14561360			chr9:124854824-124855819	S_Shore
hyper504	FGR_adequate_3	4.0E-03	0.21	0.08	0.14	9	130965902	cg21240523	CIZ1;DNM1;DNM1;CIZ1	5'UTR;1stExon;1stExon;5'UTR	chr9:130965464-130966516	Island
hyper505	FGR_adequate_3	1.2E-06	0.23	0.08	0.15	9	131940009	cg17885719	IER5L	1stExon	chr9:131936269-131941021	Island
hyper506	FGR_adequate_3	3.1E-08	0.28	0.06	0.22	10	18948698	cg08692130	ARL5B	Body	chr10:18948113-18948711	Island
hyper507	FGR_adequate_3	1.7E-07	0.19	0.06	0.12	10	31892190	cg02900332			chr10:31892189-31892752	Island
hyper508	FGR_adequate_3	0.0E+00	0.40	0.09	0.32	10	38691306	cg20942625	CDC10L	Body	chr10:38691424-38692162	N_Shore
hyper509	FGR_adequate_3	0.0E+00	0.32	0.08	0.24	10	38691403	cg22767914	CDC10L	Body	chr10:38691424-38692162	N_Shore
hyper510	FGR_adequate_3	6.1E-03	0.33	0.03	0.30	10	47008185	cg14673387			chr10:47008084-47008410	Island
hyper511	FGR_adequate_3	5.5E-03	0.38	0.03	0.35	10	47008202	cg12043818			chr10:47008084-47008410	Island

hyper512	FGR_adequate_3	1.3E-03	0.25	0.12	0.14	10	49813142	cg25290938	ARHGAP22	TSS200	chr10:49812852-49813352	Island
hyper513	FGR_adequate_3	1.3E-05	0.26	0.05	0.21	10	49813155	cg10242724	ARHGAP22	TSS200	chr10:49812852-49813352	Island
hyper514	FGR_adequate_3	3.7E-02	0.14	0.07	0.07	10	54073642	cg27411220	DKK1	TSS1500	chr10:54073989-54074827	N_Shore
hyper515	FGR_adequate_3	1.5E-02	0.05	0.01	0.04	10	111683345	cg05697697	XPNPEP1;XPNPEP1;XPNPEP1	TSS200;TSS200;TSS200	chr10:111682775-111683483	Island
hyper516	FGR_adequate_3	1.5E-02	0.14	0.04	0.10	11	790177	cg14183693	CEND1	TSS200	chr11:791953-792400	N_Shore
hyper517	FGR_adequate_3	2.1E-05	0.30	0.10	0.20	11	11643760	cg19755007	GALNTL4	TSS200	chr11:11642121-11643819	Island
hyper518	FGR_adequate_3	5.4E-02	0.54	0.14	0.40	11	69258203	cg06921552			chr11:69258149-69258544	Island
hyper519	FGR_adequate_3	1.4E-02	0.45	0.17	0.28	11	69258477	cg04729730			chr11:69258149-69258544	Island
hyper520	FGR_adequate_3	7.2E-06	0.28	0.08	0.20	12	49297569	cg22827806	CCDC65	TSS1500		
hyper521	FGR_adequate_3	2.4E-03	0.20	0.08	0.12	12	49297974	cg08869573	CCDC65;CCDC65	1stExon;5'UTR		
hyper522	FGR_adequate_3	2.9E-09	0.52	0.13	0.39	12	54567248	cg11426224				
hyper523	FGR_adequate_3	3.0E-03	0.28	0.09	0.19	12	54567476	cg15344914				
hyper524	FGR_adequate_3	7.4E-02	0.07	0.03	0.04	12	58145986	cg16716518	CDK4;CDK4	5'UTR;1stExon	chr12:58145853-58146360	Island
hyper525	FGR_adequate_3	1.4E-04	0.45	0.05	0.40	12	122688708	cg27049761	B3GNT4	5'UTR	chr12:122687800-122689106	Island
hyper526	FGR_adequate_3	6.6E-09	0.45	0.05	0.40	12	122688787	cg23445859	B3GNT4	5'UTR	chr12:122687800-122689106	Island
hyper527	FGR_adequate_3	7.5E-04	0.46	0.12	0.34	12	122688969	cg11970289	B3GNT4	5'UTR	chr12:122687800-122689106	Island
hyper528	FGR_adequate_3	1.3E-08	0.21	0.07	0.14	12	125411780	cg09234599			chr12:125412891-125413165	N_Shore
hyper529	FGR_adequate_3	8.4E-02	0.12	0.06	0.06	14	55658290	cg25465634	DLGAP5;DLGAP5;DLGAP5;DLGA	5'UTR;1stExon;5'UTR;1stExon	chr14:55658232-55658550	Island
hyper530	FGR_adequate_3	7.2E-05	0.38	0.05	0.33	14	70038884	cg18477204	C14orf162	TSS1500	chr14:70038108-70040302	Island
hyper531	FGR_adequate_3	4.0E-04	0.35	0.07	0.27	14	70038925	cg02380334	C14orf162	TSS1500	chr14:70038108-70040302	Island
hyper532	FGR_adequate_3	0.0E+00	0.71	0.07	0.65	14	77494451	cg02583546	C14orf4;C14orf4	5'UTR;1stExon	chr14:77491938-77495276	Island
hyper533	FGR_adequate_3	2.2E-02	0.80	0.50	0.30	15	62516405	cg03455316			chr15:62516324-62516805	Island
hyper534	FGR_adequate_3	1.9E-02	0.39	0.15	0.24	16	2848409	cg25618731	TESSP1	TSS200	chr16:2848426-2849145	N_Shore
hyper535	FGR_adequate_3	3.3E-05	0.31	0.03	0.28	16	55690443	cg09321400	SLC6A2	TSS200	chr16:55689598-55690895	Island
hyper536	FGR_adequate_3	1.3E-03	0.31	0.03	0.28	16	89641296	cg02449821	CPNE7;CPNE7	TSS1500;TSS1500	chr16:89641053-89643415	Island
hyper537	FGR_adequate_3	2.1E-06	0.14	0.01	0.13	17	17109640	cg03292213	PLD6;PLD6	1stExon;5'UTR	chr17:17108771-17109701	Island
hyper538	FGR_adequate_3	5.3E-08	0.11	0.01	0.10	17	17109651	cg11392858	PLD6	TSS200	chr17:17108771-17109701	Island
hyper539	FGR_adequate_3	3.6E-05	0.09	0.02	0.07	17	17109678	cg23661344	PLD6	TSS200	chr17:17108771-17109701	Island
hyper540	FGR_adequate_3	3.0E-04	0.23	0.06	0.17	17	17109740	cg00572586	PLD6	TSS200	chr17:17109893-17110296	N_Shore
hyper541	FGR_adequate_3	3.4E-03	0.18	0.08	0.11	17	33760419	cg18587557	SLFN12	TSS1500		
hyper542	FGR_adequate_3	3.5E-02	0.16	0.09	0.07	17	46688490	cg09357097	HOXB7	TSS200	chr17:46687527-46688730	Island
hyper543	FGR_adequate_3	2.1E-04	0.31	0.04	0.28	17	46688593	cg24712277	HOXB7	TSS1500	chr17:46687527-46688730	Island
hyper544	FGR_adequate_3	2.7E-02	0.37	0.06	0.31	17	46688729	cg22291922	HOXB7	TSS1500	chr17:46687527-46688730	Island
hyper545	FGR_adequate_3	7.1E-02	0.33	0.06	0.27	17	46688845	cg23730436	HOXB7	TSS1500	chr17:46690390-46691055	N_Shore
hyper546	FGR_adequate_3	3.9E-02	0.04	0.01	0.03	17	61905144	cg22530822	FTSJ3;PSMC5	TSS200;Body	chr17:61904726-61905184	Island
hyper547	FGR_adequate_3	8.9E-06	0.25	0.10	0.15	17	66192411	cg25077430			chr17:66192363-66195873	Island
hyper548	FGR_adequate_3	1.3E-07	0.34	0.09	0.25	17	66192886	cg13537781			chr17:66192363-66195873	Island
hyper549	FGR_adequate_3	4.1E-03	0.38	0.09	0.30	17	79370518	cg06873452			chr17:79366806-79374742	Island
hyper550	FGR_adequate_3	3.7E-03	0.43	0.09	0.34	17	79371014	cg18549292			chr17:79366806-79374742	Island
hyper551	FGR_adequate_3	1.1E-03	0.10	0.03	0.07	18	11908803	cg16860267	MPPE1	TSS200	chr18:11908222-11909082	Island
hyper552	FGR_adequate_3	2.2E-02	0.20	0.09	0.11	19	17799325	cg26496291	UNC13A	TSS1500	chr19:17798679-17799329	Island
hyper553	FGR_adequate_3	9.9E-03	0.15	0.04	0.10	19	36822770	cg22456785			chr19:36822395-36822690	S_Shore
hyper554	FGR_adequate_3	4.5E-04	0.24	0.06	0.18	19	47742829	cg17384056			chr19:47742725-47743151	Island
hyper555	FGR_adequate_3	1.3E-02	0.10	0.03	0.07	19	55851052	cg18844304	SUV420H2	TSS200	chr19:55850495-55851493	Island
hyper556	FGR_adequate_3	4.1E-03	0.10	0.05	0.05	19	56136136	cg00766729	ZNF784	TSS200	chr19:56135773-56136007	S_Shore
hyper557	FGR_adequate_3	1.5E-02	0.11	0.04	0.08	20	36793705	cg08545268	TGM2;TGM2	TSS200;TSS200	chr20:36793549-36793867	Island
hyper558	FGR_adequate_3	2.3E-04	0.07	0.02	0.05	20	36793715	cg12947626	TGM2;TGM2	TSS200;TSS200	chr20:36793549-36793867	Island
hyper559	FGR_adequate_3	5.6E-04	0.05	0.01	0.04	20	36793726	cg11059239	TGM2;TGM2	TSS200;TSS200	chr20:36793549-36793867	Island
hyper560	FGR_adequate_3	2.2E-03	0.12	0.07	0.05	20	44440947	cg03843031	UBE2C;UBE2C;UBE2C;UBE2C;U	TSS1500;TSS1500;TSS1500;TSS	chr20:44440946-44441655	Island
hyper561	FGR_adequate_3	3.0E-04	0.27	0.09	0.18	20	45338122	cg08038974	SLC2A10	TSS200	chr20:45338071-45338663	Island
hyper562	FGR_adequate_3	0.0E+00	0.42	0.01	0.41	20	45338329	cg13690178	SLC2A10;SLC2A10	5'UTR;1stExon	chr20:45338071-45338663	Island
hyper563	FGR_adequate_3	0.0E+00	0.45	0.08	0.37	20	45338396	cg16117472	SLC2A10	Body	chr20:45338071-45338663	Island
hyper564	FGR_adequate_3	0.0E+00	0.34	0.06	0.28	20	45338652	cg17550582	SLC2A10	Body	chr20:45338071-45338663	Island
hyper565	FGR_adequate_3	7.3E-02	0.29	0.08	0.21	22	31002942	cg00788739	TCN2	TSS1500		
hyper566	FGR_adequate_3	0.0E+00	0.35	0.11	0.25	22	39378235	cg14194956	APOBEC3B	TSS200		
hyper567	FGR_adequate_3	1.9E-10	0.26	0.06	0.20	22	39378382	cg25787886	APOBEC3B	TSS200		
hyper568	FGR_adequate_3	6.5E-03	0.14	0.05	0.09	22	42095368	cg10296410	MEI1	TSS200	chr22:42095414-42096189	N_Shore
hyper569	FGR_adequate_4	7.8E-10	0.14	0.01	0.13	1	6052487	cg05933935	NPHP4;NPHP4	1stExon;5'UTR	chr1:6051595-6052955	Island
hyper570	FGR_adequate_4	0.0E+00	0.11	0.01	0.10	1	6052495	cg00931619	NPHP4;NPHP4	1stExon;5'UTR	chr1:6051595-6052955	Island
hyper571	FGR_adequate_4	0.0E+00	0.12	0.01	0.12	1	6052500	cg20797142	NPHP4;NPHP4	1stExon;5'UTR	chr1:6051595-6052955	Island
hyper572	FGR_adequate_4	4.9E-10	0.22	0.08	0.14	1	8483390	cg15889173	RERE;RERE;RERE	5'UTR;Body;Body	chr1:8483389-8484339	Island
hyper573	FGR_adequate_4	0.0E+00	0.20	0.03	0.17	1	8483475	cg22736444	RERE;RERE;RERE	5'UTR;Body;Body	chr1:8483389-8484339	Island
hyper574	FGR_adequate_4	0.0E+00	0.20	0.02	0.18	1	8484169	cg15492466	RERE;RERE;RERE	Body;TSS1500;Body	chr1:8483389-8484339	Island
hyper575	FGR_adequate_4	0.0E+00	0.21	0.02	0.19	1	8484310	cg23834414	RERE;RERE;RERE	Body;TSS1500;Body	chr1:8483389-8484339	Island
hyper576	FGR_adequate_4	7.7E-07	0.21	0.04	0.17	1	111097901	cg04780572			chr1:111098066-111098362	N_Shore
hyper577	FGR_adequate_4	6.8E-02	0.16	0.02	0.14	1	223316531	cg17599809	TLR5	5'UTR	chr1:223316185-223316689	Island
hyper578	FGR_adequate_4	4.6E-02	0.07	0.02	0.05	1	236305619	cg17269430	GPR137B	TSS1500	chr1:236305534-236306674	Island
hyper579	FGR_adequate_4	4.5E-02	0.05	0.01	0.04	1	236305663	cg07425541	GPR137B	TSS200	chr1:236305534-236306674	Island
hyper580	FGR_adequate_4	1.3E-08	0.15	0.06	0.10	1	236305875	cg21434251	GPR137B;GPR137B	1stExon;5'UTR	chr1:236305534-236306674	Island
hyper581	FGR_adequate_4	0.0E+00	0.16	0.01	0.15	1	236306000	cg09169938	GPR137B	1stExon	chr1:236305534-236306674	Island
hyper582	FGR_adequate_4	2.5E-02	0.18	0.09	0.09	1	236306311	cg26534508	GPR137B	1stExon	chr1:236305534-236306674	Island
hyper583	FGR_adequate_4	1.2E-02	0.24	0.08	0.17	2	121200690	cg21856256			chr2:121200503-121200788	Island
hyper584	FGR_adequate_4	4.7E-02	0.18	0.09	0.09	3	10857800	cg26836233	SLC6A11	TSS200	chr3:10857687-10858447	Island
hyper585	FGR_adequate_4	4.3E-05	0.24	0.06	0.18	3	112051894	cg01031032	CD200;CD200	TSS200;TSS200	chr3:112051893-112052406	Island
hyper586	FGR_adequate_4	9.7E-11	0.48	0.06	0.42	3	196695938	cg05938285	PIGZ	TSS1500	chr3:196695319-196697065	Island
hyper587	FGR_adequate_4	7.7E-02	0.55	0.04	0.51	3	196697064	cg00828689	PIGZ	TSS1500	chr3:196695319-196697065	Island
hyper588	FGR_adequate_4	4.2E-05	0.14	0.07	0.07	4	73433671	cg07940440	ADAMTS3	Body	chr4:73433783-73434289	N_Shore
hyper589	FGR_adequate_4	1.9E-06	0.11	0.02	0.09	4	73433788	cg18123319	ADAMTS3	Body	chr4:73433783-73434289	Island
hyper590	FGR_adequate_4	7.0E-07	0.27	0.09	0.18	4	73434035	cg08232905	ADAMTS3	Body	chr4:73433783-73434289	Island
hyper591	FGR_adequate_4	0.0E+00	0.24	0.04	0.20	4	73434209	cg13643796	ADAMTS3	Body	chr4:73433783-73434289	Island
hyper592	FGR_adequate_4	2.2E-04	0.13	0.03	0.09	4	73434487	cg25889750	ADAMTS3;ADAMTS3	1stExon;5'UTR	chr4:73434855-73435321	N_Shore
hyper593	FGR_adequate_4	4.3E-09	0.29	0.07	0.22	4	73434591	cg15102770	ADAMTS3	TSS200	chr4:73434855-73435321	N_Shore
hyper594	FGR_adequate_4	4.4E-06	0.32	0.12	0.20	4	73434820	cg27185819	ADAMTS3	TSS1500	chr4:73434855-73435321	N_Shore
hyper595	FGR_adequate_4	0.0E+00	0.26	0.04	0.21	4	73434856	cg11243196	ADAMTS3	TSS1500	chr4:73434855-73435321	Island
hyper596	FGR_adequate_4	0.0E+00	0.16	0.02	0.14	4	73435229	cg08324945	ADAMTS3	TSS1500	chr4:73434855-73435321	Island
hyper597	FGR_adequate_4	4.5E-07	0.21	0.02	0.19	4	94749890	cg26124980	ATOH1	TSS200	chr4:94749495-94750998	Island
hyper598	FGR_adequate_4	8.1E-02	0.25	0.12	0.13	4	96468962	cg03008707	UNC5C	Body	chr4:96469456-96469667	N_Shore
hyper599	FGR_adequate_4	8.1E-04	0.17	0.07	0.10	4	96469067	cg17031751	UNC5C	Body	chr4:96469456-96469667	N_Shore
hyper600	FGR_adequate_4	2.3E-04	0.16	0.07	0.09	4	96469264	cg01788994	UNC5C	Body	chr4:96469456-96469667	N_Shore
hyper601	FGR_adequate_4	9.7E-11	0.20	0.06	0.14	4	96469286	cg12412390	UNC5C	Body	chr4:96469456-96469667	N_Shore
hyper602	FGR_adequate_4	6.6E-07	0.12	0.02	0.10	4	96469519	cg24870497	UNC5C	Body	chr4:96469456-96469667	Island
hyper603	FGR_adequate_4	0.0E+00	0.22	0.07	0.15	4	96470053	cg00329039	UNC5C;UNC5C	5'UTR;1stExon	chr4:96469859-96470994	Island
hyper604	FGR_adequate_4	2.7E-09	0.21	0.08	0.14	4	96470237	cg07234102	UNC5C	TSS200	chr4:96469859-96470994	Island
hyper605	FGR_adequate_4	0.0E+00	0.24	0.01	0.23	4	96470286	cg11723848	UNC5C	TSS200	chr4:96469859-96470994	Island
hyper606	FGR_adequate_4	8.8E-10	0.23	0.07	0.15	4	96470349	cg15984718	UNC5C	TSS200	chr4:96469859-96470994	Island
hyper607	FGR_adequate_4	0.0E+00	0.24	0.05	0.19	4	96470584	cg13265789	UNC5C	TSS1500	chr4:96469859-96470994	Island
hyper608	FGR_adequate_4	0.0E+00	0.27	0.01	0.26	4	96470887	cg21782409	UNC5C	TSS1500	chr4:96469859-96470994	Island
hyper609	FGR_adequate_4	3.5E-09	0.21	0.07	0.15	4	96470993	cg12802900	UNC5C	TSS1500	chr4:96469859-96470994	Island
hyper610	FGR_adequate_4	0.0E+00	0.26	0.06	0.19	4	96471105	cg12584684	UNC5C	TSS1500	chr4:96469456-96469667	S_Shore
hyper611	FGR_adequate_4	4.2E-09	0.18	0.05	0.14	4	96471143	cg15701178	UNC5C	TSS1500	chr4:96469456-96469667	S_Shore
hyper612	FGR_adequate_4	7.0E-03	0.15	0.02	0.13	4	134070416	cg27600205	PCDH10;PCDH10	TSS200;TSS200	chr4:134069162-134070442	Island
hyper613	FGR_adequate_4	4.1E-02	0.24	0.09	0.15	4	134070433	cg10524033	PCDH10;PCDH10	TSS200;TSS200	chr4:134069162-134070442	Island
hyper614	FGR_adequate_4	3.0E-02	0.26	0.09	0.17	4	134070437	cg01408654	PCDH10;PCDH10	TSS200;TSS200	chr4:134069162-134070442	Island
hyper615	FGR_adequate_4	9.0E-02	0.33	0.04	0.29	5	96143598	cg23215014	ERAP1;ERAP1;ERAP1;ERAP1	5'UTR;5'UTR;1stExon;1stExon	chr5:96143102-96143885	Island
hyper616	FGR_adequate_4	5.6E-07	0.06	0.01	0.05	6	29974715	cg04566848	HLA-J;NCRNA00171	Body;Body	chr6:29974220-29975369	Island
hyper617	FGR_adequate_4	5.4E-04	0.08	0.01	0.07	6	29974723	cg01814945	HLA-J;NCRNA00171	Body;Body	chr6:29974220-29975369	Island
hyper618	FGR_adequate_4	3.6E-02	0.22	0.05	0.17	6	29975068	cg21330423	HLA-J;NCRNA00171	Body;Body	chr6:29974220-29975369	Island
hyper619	FGR_adequate_4	3.5E-09	0.24	0.05	0.19	6	29975078	cg09659004	HLA-J;NCRNA00171	Body;Body	chr6:29974220-29975369	Island
hyper620	FGR_adequate_4	2.8E-05	0.27	0.03	0.24	6	114178220	cg02446434	MARCKS	TSS1500	chr6:114179734-114181226	N_Shore
hyper621	FGR_adequate_4	8.9E-03	0.21	0.07	0.15	6	114178324	cg20031659	MARCKS	TSS1500	chr6:114179734-114181226	N_Shore
hyper622	FGR_adequate_4	3.5E-04	0.12	0.04	0.08	6	114178410	cg07484590	MARCKS	TSS200	chr6:114179734-114181226	N_Shore
hyper623	FGR_adequate_4	4.6E-04	0.25	0.03	0.22	6	152128865	cg15980539	ESR1;ESR1;ESR1;ESR1;ESR1	5'UTR;5'UTR;1stExon;5'UTR;5'UT	chr6:152128822-152129771	Island
hyper624	FGR_adequate_4	4.3E-07	0.22	0.06	0.16	6	152129388	cg00920970	ESR1;ESR1;ESR1;ESR1	Body;Body;1stExon;Body	chr6:152128822-152129771	Island
hyper625	FGR_adequate_4	1.1E-03	0.19	0.08	0.12	6	152129400	cg20253551	ESR1;ESR1;ESR1;ESR1	Body;Body;1stExon;Body	chr6:152128822-152129771	Island
hyper626	FGR_adequate_4	1.4E-04	0.03	0.00	0.03	7	149290	cg13098082			chr7:149494-150038	N_Shore
hyper627	FGR_adequate_4	3.1E-02	0.18	0.08	0.10	7	63774181	cg14289939	LOC728927	5'UTR		
hyper628	FGR_adequate_4	1.1E-06	0.19	0.03	0.15	7	63774897	cg21439880	LOC728927	Body		
hyper629	FGR_adequate_4	3.0E-04	0.10	0.03	0.07	7	92462706	cg10779184	CDK6;CDK6	5'UTR;5'UTR	chr7:92462201-92466387	Island
hyper630	FGR_adequate_4	3.3E-05	0.17	0.04	0.13	7	124404822	cg02960853	GPR37	1stExon	chr7:124404700-124406189	Island
hyper631	FGR_adequate_4	1.7E-04	0.22	0.08	0.14	10	45496237	cg01183826	C10orf25;ZNF22	1stExon;TSS200	chr10:45496275-45496550	N_Shore
hyper632	FGR_adequate_4	7.8E-06	0.15	0.02	0.13	10	45496315	cg14539394	C10orf25;ZNF22;C10orf25;ZNF22	1stExon;1stExon;5'UTR;5'UTR	chr10:45496275-45496550	Island
hyper633	FGR_adequate_4	0.0E+00	0.18	0.02	0.16	10	60272754	cg07857251	BICC1	1stExon	chr10:60272467-60273456	Island
hyper634	FGR_adequate_4	0.0E+00	0.26	0.01	0.25	10	60272938	cg19940537	BICC1	1stExon	chr10:60272467-60273456	Island
hyper635	FGR_adequate_4	2.4E-02	0.14	0.04	0.10	10	60273225	cg11142981	BICC1	Body	chr10:60272467-60273456	Island
hyper636	FGR_adequate_4	3.6E-03	0.18	0.04	0.14	11	79152102	cg12246510	ODZ4	TSS1500	chr11:79148358-79152200	Island
hyper637	FGR_adequate_4	1.9E-10	0.23	0.04	0.19	12	107380962	cg14135757	MTERFD3;MTERFD3	TSS200;TSS200	chr12:107380558-107381076	Island
hyper638	FGR_adequate_4	9.2E-04	0.19	0.08	0.11	12	107381105	cg06758649	MTERFD3;MTERFD3	TSS200;TSS200	chr12:107380558-107381076	S_Shore
hyper639	FGR_adequate_4	5.1E-04	0.10	0.03	0.07	12	107713740	cg24966575	BTBD11	1stExon	chr12:107711603-107714107	Island
hyper640	FGR_adequate_4	6.8E-03	0.67	0.41	0.27	13	103523190	cg01783816	ERCC5	Body		

hyper641	FGR_adequate_4	2.9E-03	0.10	0.04	0.07	15	41952458	cg17178280	MGA;MGA	TSS200;TSS200	chr15:41952535-41953222	N_Shore
hyper642	FGR_adequate_4	5.7E-06	0.24	0.04	0.19	15	41952645	cg22546696	MGA;MGA;MGA;MGA	1stExon;1stExon;5'UTR;5'UTR	chr15:41952535-41953222	Island
hyper643	FGR_adequate_4	5.7E-08	0.12	0.02	0.09	15	41952827	cg24856258	MGA;MGA	5'UTR;5'UTR	chr15:41952535-41953222	Island
hyper644	FGR_adequate_4	5.6E-02	0.29	0.05	0.23	15	41953061	cg25826226	MGA;MGA	5'UTR;5'UTR	chr15:41952535-41953222	Island
hyper645	FGR_adequate_4	9.4E-06	0.19	0.03	0.16	16	3079708	cg07868599	CCDC64B	Body	chr16:3079536-3079954	Island
hyper646	FGR_adequate_4	2.9E-05	0.20	0.06	0.13	17	70536484	cg09672890			chr17:70536483-70536687	Island
hyper647	FGR_adequate_4	8.4E-04	0.26	0.10	0.16	19	9517858	cg23891996			chr19:9517468-9517986	Island
hyper648	FGR_adequate_4	8.4E-03	0.25	0.09	0.16	19	38042472	cg03975694	ZNF540;ZNF540	5'UTR;1stExon	chr19:38039561-38040395	S_Shelf
hyper649	FGR_adequate_4	1.6E-05	0.18	0.04	0.14	19	54926734	cg23695687	TTYH1;TTYH1	1stExon;1stExon	chr19:54927902-54928225	N_Shore
hyper650	FGR_adequate_4	8.2E-03	0.09	0.03	0.05	19	58326288	cg16615451	ZNF552	TSS200	chr19:58325855-58326330	Island
hyper651	FGR_adequate_4	1.6E-03	0.28	0.06	0.22	20	58508888	cg20800536			chr20:58507755-58509517	Island
hyper652	FGR_adequate_4	9.4E-04	0.34	0.07	0.27	20	58508922	cg02801359			chr20:58507755-58509517	Island
hyper653	FGR_adequate_4	4.7E-03	0.35	0.02	0.33	20	58508943	cg26690949			chr20:58507755-58509517	Island
hyper654	FGR_adequate_4	9.1E-04	0.25	0.09	0.16	20	58509242	cg00125414			chr20:58507755-58509517	Island
hyper655	FGR_adequate_4	7.6E-03	0.23	0.09	0.14	20	58509298	cg14536096			chr20:58507755-58509517	Island
hyper656	FGR_adequate_4	2.0E-03	0.09	0.01	0.08	21	47063285	cg01145054			chr21:47061773-47064308	Island
hyper657	FGR_adequate_5	0.0E+00	0.30	0.02	0.28	1	84464645	cg00773459	TTLL7;TTLL7	1stExon;5'UTR	chr1:84464223-84465232	Island
hyper658	FGR_adequate_5	1.6E-04	0.12	0.03	0.09	1	113261488	cg26044038			chr1:113261468-113261765	Island
hyper659	FGR_adequate_5	5.3E-05	0.09	0.02	0.07	1	113261613	cg10839663			chr1:113261468-113261765	Island
hyper660	FGR_adequate_5	4.5E-03	0.14	0.07	0.07	1	196577723	cg14664759	KCNT2	TSS1500	chr1:196577708-196578011	Island
hyper661	FGR_adequate_5	2.1E-02	0.07	0.02	0.05	1	234350003	cg10701282	SLC35F3	Body	chr1:234349884-234350471	Island
hyper662	FGR_adequate_5	8.3E-03	0.21	0.03	0.18	2	175201062	cg00153110	SP9	Body	chr2:175199463-175202639	Island
hyper663	FGR_adequate_5	0.0E+00	0.23	0.02	0.21	5	175792478	cg22424536	ARL10	TSS200	chr5:175792355-175793505	Island
hyper664	FGR_adequate_5	3.6E-03	0.26	0.02	0.24	5	175792498	cg09155701	ARL10	TSS200	chr5:175792355-175793505	Island
hyper665	FGR_adequate_5	4.4E-03	0.32	0.02	0.29	5	175792510	cg26720389	ARL10;ARL10	1stExon;5'UTR	chr5:175792355-175793505	Island
hyper666	FGR_adequate_5	7.8E-02	0.12	0.04	0.08	8	48100410	cg05746795			chr8:48100163-48100914	Island
hyper667	FGR_adequate_5	1.9E-08	0.10	0.01	0.09	8	48100429	cg02230854			chr8:48100163-48100914	Island
hyper668	FGR_adequate_5	0.0E+00	0.20	0.06	0.14	9	26956236	cg14408431	IFT74;IFT74;IFT74;IFT74	5'UTR;5'UTR;TSS200;TSS200	chr9:26956301-26956770	N_Shore
hyper669	FGR_adequate_5	3.6E-08	0.16	0.04	0.13	9	26956302	cg04415978	IFT74;IFT74;IFT74;IFT74	5'UTR;5'UTR;TSS200;TSS200	chr9:26956301-26956770	Island
hyper670	FGR_adequate_5	9.3E-04	0.07	0.02	0.05	13	38924368	cg04012509	UFM1	Body	chr13:38923957-38924413	Island
hyper671	FGR_adequate_5	4.8E-06	0.17	0.02	0.15	13	78493100	cg04390523	EDNRB;EDNRB;EDNRB	5'UTR;TSS200;TSS200	chr13:78492425-78493382	Island
hyper672	FGR_adequate_5	1.9E-02	0.19	0.09	0.10	13	78493182	cg01910869	EDNRB;EDNRB;EDNRB	TSS1500;5'UTR;TSS1500	chr13:78492425-78493382	Island
hyper673	FGR_adequate_5	1.6E-02	0.15	0.06	0.09	13	78493294	cg12602112	EDNRB;EDNRB;EDNRB	TSS1500;5'UTR;TSS1500	chr13:78492425-78493382	Island
hyper674	FGR_adequate_5	9.8E-03	0.18	0.07	0.11	13	78493297	cg19650157	EDNRB;EDNRB;EDNRB	TSS1500;5'UTR;TSS1500	chr13:78492425-78493382	Island
hyper675	FGR_adequate_5	3.1E-02	0.14	0.04	0.11	13	78493305	cg13434989	EDNRB;EDNRB;EDNRB	TSS1500;5'UTR;TSS1500	chr13:78492425-78493382	Island
hyper676	FGR_adequate_5	3.2E-02	0.16	0.06	0.10	13	78493313	cg10016380	EDNRB;EDNRB;EDNRB	TSS1500;5'UTR;TSS1500	chr13:78492425-78493382	Island
hyper677	FGR_adequate_5	6.9E-03	0.22	0.07	0.15	13	78493365	cg19742055	EDNRB;EDNRB;EDNRB	TSS1500;5'UTR;TSS1500	chr13:78492425-78493382	Island
hyper678	FGR_adequate_5	3.3E-04	0.20	0.07	0.13	13	78493561	cg06057566	EDNRB;EDNRB;EDNRB	TSS1500;5'UTR;TSS1500	chr13:78492425-78493382	S_Shore
hyper679	FGR_adequate_5	1.2E-04	0.20	0.03	0.16	13	78493631	cg25717994	EDNRB;EDNRB;EDNRB	TSS1500;5'UTR;TSS1500	chr13:78492425-78493382	S_Shore
hyper680	FGR_adequate_5	8.4E-07	0.15	0.02	0.14	13	78493651	cg09152886	EDNRB;EDNRB;EDNRB	TSS1500;5'UTR;TSS1500	chr13:78492425-78493382	S_Shore
hyper681	FGR_adequate_5	5.9E-04	0.18	0.08	0.10	13	78493854	cg16739796	EDNRB;EDNRB;EDNRB	TSS1500;5'UTR;TSS1500	chr13:78492425-78493382	S_Shore
hyper682	FGR_adequate_5	7.5E-05	0.17	0.05	0.11	13	78493874	cg21364111	EDNRB;EDNRB;EDNRB	TSS1500;5'UTR;TSS1500	chr13:78492425-78493382	S_Shore
hyper683	FGR_adequate_5	4.4E-02	0.19	0.08	0.12	13	78493920	cg12983394	EDNRB;EDNRB;EDNRB	TSS1500;5'UTR;TSS1500	chr13:78492425-78493382	S_Shore
hyper684	FGR_adequate_5	3.7E-03	0.05	0.01	0.04	13	108867893	cg05180121	LIG4;LIG4;LIG4	TSS1500;TSS200;5'UTR	chr13:108867107-108867364	S_Shore
hyper685	FGR_adequate_5	1.6E-03	0.33	0.05	0.28	15	83316223	cg27578811	CPEB1	Body	chr15:83315116-83317541	Island
hyper686	FGR_adequate_5	5.5E-02	0.23	0.11	0.12	15	83316911	cg24319381	CPEB1	TSS200	chr15:83315116-83317541	Island
hyper687	FGR_adequate_5	1.9E-05	0.33	0.12	0.21	16	85722694	cg27360527	GINS2	TSS200	chr16:85722209-85722941	Island
hyper688	FGR_adequate_5	0.0E+00	0.11	0.01	0.10	17	33569747	cg21383891	SLFN5	TSS1500	chr17:33569141-33570527	Island
hyper689	FGR_adequate_5	4.0E-05	0.20	0.03	0.17	19	46916741	cg06747432	CCDC8;CCDC8	1stExon;5'UTR	chr19:46916587-46916862	Island
hyper690	FGR_adequate_5	2.8E-03	0.19	0.05	0.14	20	22565240	cg06697267	FOXA2;FOXA2	5'UTR;TSS200	chr20:22562736-22566104	Island
hyper691	FGR_excessive_1	4.3E-07	0.32	0.12	0.20	1	47691158	cg10380348	TAL1	Body	chr1:47690980-47691727	Island
hyper692	FGR_excessive_1	2.0E-02	0.18	0.06	0.12	1	47691160	cg10810752	TAL1	Body	chr1:47690980-47691727	Island
hyper693	FGR_excessive_1	2.3E-08	0.22	0.07	0.15	1	77747875	cg17484154	AK5;AK5;AK5	1stExon;TSS1500;5'UTR	chr1:77747314-77748224	Island
hyper694	FGR_excessive_1	4.2E-07	0.32	0.02	0.31	1	77747987	cg04867733	AK5;AK5;AK5	1stExon;TSS1500;5'UTR	chr1:77747314-77748224	Island
hyper695	FGR_excessive_1	7.2E-03	0.21	0.03	0.18	1	91196332	cg14419975			chr1:91190489-91192804	S_Shelf
hyper696	FGR_excessive_1	4.9E-02	0.11	0.03	0.08	1	99470618	cg19365673	LPPR5;LPPR5	TSS200;TSS200	chr1:99469932-99470968	Island
hyper697	FGR_excessive_1	6.5E-04	0.24	0.12	0.13	1	116380316	cg11071926	NHLH2;NHLH2	3'UTR;3'UTR	chr1:116380359-116382364	N_Shore
hyper698	FGR_excessive_1	5.9E-03	0.33	0.13	0.20	1	116380417	cg21863110	NHLH2;NHLH2	3'UTR;3'UTR	chr1:116380359-116382364	Island
hyper699	FGR_excessive_1	0.0E+00	0.38	0.01	0.36	1	116380572	cg25979559	NHLH2;NHLH2	3'UTR;3'UTR	chr1:116380359-116382364	Island
hyper700	FGR_excessive_1	2.4E-04	0.21	0.08	0.13	1	116380946	cg00960552	NHLH2;NHLH2	Body;Body	chr1:116380359-116382364	Island
hyper701	FGR_excessive_1	9.2E-02	0.19	0.02	0.17	1	147624510	cg10127479			chr1:147623646-147624656	Island
hyper702	FGR_excessive_1	7.2E-08	0.30	0.11	0.19	1	239549830	cg08542090			chr1:239550108-239551339	N_Shore
hyper703	FGR_excessive_1	9.7E-03	0.12	0.07	0.06	1	239549853	cg23684246			chr1:239550108-239551339	N_Shore
hyper704	FGR_excessive_1	6.7E-04	0.16	0.05	0.11	1	239550177	cg22017581			chr1:239550108-239551339	Island
hyper705	FGR_excessive_1	1.1E-06	0.37	0.02	0.35	1	239550378	cg00371368			chr1:239550108-239551339	Island
hyper706	FGR_excessive_1	2.4E-03	0.33	0.08	0.25	2	37572289	cg17304496	QPCT	Body	chr2:37571676-37572397	Island
hyper707	FGR_excessive_1	9.0E-02	0.28	0.10	0.18	2	66803697	cg09902029			chr2:66802890-66804831	Island
hyper708	FGR_excessive_1	1.9E-10	0.26	0.07	0.18	2	69968558	cg19353425	ANXA4	TSS1500	chr2:69968749-69969592	N_Shore
hyper709	FGR_excessive_1	8.8E-10	0.23	0.04	0.19	2	69968602	cg17372745	ANXA4	TSS1500	chr2:69968749-69969592	N_Shore
hyper710	FGR_excessive_1	0.0E+00	0.30	0.01	0.29	2	69968853	cg09547034	ANXA4	TSS1500	chr2:69968749-69969592	Island
hyper711	FGR_excessive_1	0.0E+00	0.49	0.09	0.40	2	69968919	cg26034355	ANXA4	TSS1500	chr2:69968749-69969592	Island
hyper712	FGR_excessive_1	0.0E+00	0.43	0.09	0.35	2	69968926	cg03636174	ANXA4	TSS200	chr2:69968749-69969592	Island
hyper713	FGR_excessive_1	0.0E+00	0.38	0.03	0.35	2	69969004	cg08311172	ANXA4	TSS200	chr2:69968749-69969592	Island
hyper714	FGR_excessive_1	1.1E-09	0.42	0.08	0.33	2	69969029	cg10534439	ANXA4	TSS200	chr2:69968749-69969592	Island
hyper715	FGR_excessive_1	9.7E-11	0.42	0.08	0.35	2	69969033	cg00900124	ANXA4	TSS200	chr2:69968749-69969592	Island
hyper716	FGR_excessive_1	9.7E-11	0.39	0.06	0.33	2	69969052	cg20382057	ANXA4	TSS200	chr2:69968749-69969592	Island
hyper717	FGR_excessive_1	0.0E+00	0.47	0.02	0.46	2	69969063	cg08353937	ANXA4	TSS200	chr2:69968749-69969592	Island
hyper718	FGR_excessive_1	0.0E+00	0.36	0.01	0.35	2	69969079	cg05136786	ANXA4	TSS200	chr2:69968749-69969592	Island
hyper719	FGR_excessive_1	0.0E+00	0.35	0.05	0.30	2	69969221	cg11056435	ANXA4;ANXA4	5'UTR;1stExon	chr2:69968749-69969592	Island
hyper720	FGR_excessive_1	0.0E+00	0.39	0.05	0.34	2	69969227	cg21960922	ANXA4;ANXA4	5'UTR;1stExon	chr2:69968749-69969592	Island
hyper721	FGR_excessive_1	0.0E+00	0.41	0.04	0.37	2	69969370	cg19269713	ANXA4	5'UTR	chr2:69968749-69969592	Island
hyper722	FGR_excessive_1	1.1E-02	0.36	0.09	0.27	2	102090371	cg13274107	RFX8	5'UTR	chr2:102090370-102091130	Island
hyper723	FGR_excessive_1	3.5E-02	0.45	0.05	0.40	2	102090444	cg02878891	RFX8	5'UTR	chr2:102090370-102091130	Island
hyper724	FGR_excessive_1	1.0E-02	0.35	0.02	0.33	2	102090791	cg19987611	RFX8	5'UTR	chr2:102090370-102091130	Island
hyper725	FGR_excessive_1	2.2E-03	0.26	0.07	0.19	2	110969641	cg02169857	NCRNA00116	Body	chr2:110969852-110970222	N_Shore
hyper726	FGR_excessive_1	5.1E-02	0.39	0.09	0.31	2	110969853	cg08570243	NCRNA00116	Body	chr2:110969852-110970222	Island
hyper727	FGR_excessive_1	0.0E+00	0.40	0.09	0.31	2	157496625	ch.2.157204871R				
hyper728	FGR_excessive_1	1.0E-04	0.28	0.09	0.19	3	36985516	cg03052030	TRANK1	Body	chr3:36985540-36987041	N_Shore
hyper729	FGR_excessive_1	1.0E-06	0.25	0.07	0.17	3	36985697	cg07528097	TRANK1	Body	chr3:36985540-36987041	Island
hyper730	FGR_excessive_1	1.2E-02	0.05	0.01	0.04	3	49157911	cg13606999	USP19	5'UTR	chr3:49157768-49158463	Island
hyper731	FGR_excessive_1	3.5E-05	0.10	0.02	0.09	3	49158199	cg19979540	USP19;USP19	1stExon;5'UTR	chr3:49157768-49158463	Island
hyper732	FGR_excessive_1	8.8E-05	0.09	0.02	0.08	3	49158303	cg19452873	USP19	TSS200	chr3:49157768-49158463	Island
hyper733	FGR_excessive_1	6.4E-05	0.17	0.02	0.16	3	93781766	cg00558986	NSUN3;DHFRL1	TSS200;TSS200	chr3:93781648-93782108	Island
hyper734	FGR_excessive_1	7.8E-10	0.36	0.05	0.31	3	93781778	cg24143828	NSUN3;DHFRL1	TSS200;TSS200	chr3:93781648-93782108	Island
hyper735	FGR_excessive_1	1.9E-10	0.27	0.05	0.22	3	93781831	cg04883418	NSUN3;DHFRL1	TSS200;TSS200	chr3:93781648-93782108	Island
hyper736	FGR_excessive_1	1.5E-04	0.20	0.05	0.15	3	93782025	cg09619475	NSUN3;DHFRL1	Body;TSS1500	chr3:93781648-93782108	Island
hyper737	FGR_excessive_1	3.9E-08	0.21	0.09	0.12	4	22517195	cg26631477	GPR125	1stExon	chr4:22516856-22518048	Island
hyper738	FGR_excessive_1	2.7E-06	0.24	0.08	0.16	4	30719221	cg18449721			chr4:30718853-30719847	Island
hyper739	FGR_excessive_1	9.7E-10	0.12	0.01	0.11	4	95679381	cg21746997	BMPR1B	5'UTR	chr4:95678798-95679809	Island
hyper740	FGR_excessive_1	5.0E-02	0.31	0.08	0.23	4	155665093	cg26993729	LRAT	TSS200	chr4:155664819-155665833	Island
hyper741	FGR_excessive_1	7.8E-10	0.22	0.05	0.16	5	43043140	cg15015757	LOC153684	Body	chr5:43042107-43043477	Island
hyper742	FGR_excessive_1	5.8E-10	0.35	0.05	0.30	5	43043360	cg00741789	LOC153684	Body	chr5:43042107-43043477	Island
hyper743	FGR_excessive_1	2.0E-08	0.24	0.08	0.17	5	71402893	cg16478236	MAP1B	TSS1500	chr5:71402877-71404868	Island
hyper744	FGR_excessive_1	8.8E-10	0.19	0.06	0.13	5	71403045	cg02001410	MAP1B	TSS200	chr5:71402877-71404868	Island
hyper745	FGR_excessive_1	2.2E-02	0.14	0.05	0.09	5	71403058	cg26001333	MAP1B	TSS200	chr5:71402877-71404868	Island
hyper746	FGR_excessive_1	1.8E-09	0.23	0.03	0.20	5	71403320	cg15412498	MAP1B;MAP1B	1stExon;5'UTR	chr5:71402877-71404868	Island
hyper747	FGR_excessive_1	0.0E+00	0.24	0.01	0.23	5	71403420	cg07380496	MAP1B	1stExon	chr5:71402877-71404868	Island
hyper748	FGR_excessive_1	7.2E-07	0.10	0.01	0.09	5	71403502	cg02606808	MAP1B	1stExon	chr5:71402877-71404868	Island
hyper749	FGR_excessive_1	1.1E-02	0.17	0.07	0.10	5	157097845	cg01038640	C5orf52	TSS1500	chr5:157098263-157099041	N_Shore
hyper750	FGR_excessive_1	1.6E-03	0.43	0.02	0.42	5	174178256	cg24996482			chr5:174178214-174178928	Island
hyper751	FGR_excessive_1	1.6E-06	0.23	0.05	0.18	5	174178587	cg06916315			chr5:174178214-174178928	Island
hyper752	FGR_excessive_1	3.1E-07	0.24	0.08	0.16	5	174178647	cg24271863			chr5:174178214-174178928	Island
hyper753	FGR_excessive_1	7.4E-06	0.23	0.04	0.19	5	174178884	cg09491410			chr5:174178214-174178928	Island
hyper754	FGR_excessive_1	9.7E-11	0.37	0.04	0.33	6	30685409	cg03157605	MDC1;MDC1	1stExon;5'UTR	chr6:30684836-30685503	Island
hyper755	FGR_excessive_1	6.2E-02	0.31	0.06	0.25	6	33181412	cg20028058			chr6:33181010-33181256	S_Shore
hyper756	FGR_excessive_1	8.0E-06	0.07	0.01	0.06	7	21582745	cg16474297	DNAH11	TSS200	chr7:21582443-21583313	Island
hyper757	FGR_excessive_1	4.5E-02	0.11	0.07	0.05	7	21582749	cg08570341	DNAH11	TSS200	chr7:21582443-21583313	Island
hyper758	FGR_excessive_1	8.9E-02	0.06	0.03	0.03	7	21582758	cg01122187	DNAH11	TSS200	chr7:21582443-21583313	Island
hyper759	FGR_excessive_1	4.9E-02	0.37	0.08	0.29	8	23020966	cg23051664	TNFRSF10D	Body	chr8:23021056-23021588	N_Shore
hyper760	FGR_excessive_1	1.8E-02	0.17	0.07	0.10	8	63997977	cg27507591	TTPA	Body	chr8:63998227-63998737	N_Shore
hyper761	FGR_excessive_1	5.8E-04	0.18	0.06	0.13	8	63998164	cg23602533	TTPA	Body	chr8:63998227-63998737	N_Shore
hyper762	FGR_excessive_1	2.2E-09	0.25	0.08	0.18	8	63998228	cg13806106	TTPA	Body	chr8:63998227-63998737	Island
hyper763	FGR_excessive_1	0.0E+00	0.45	0.04	0.41	8	63998463	cg07362969	TTPA	1stExon	chr8:63998227-63998737	Island
hyper764	FGR_excessive_1	0.0E+00	0.31	0.02	0.28	8	63998640	cg07002711	TTPA	TSS200	chr8:63998227-63998737	Island
hyper765	FGR_excessive_1	4.5E-07	0.29	0.06	0.23	8	63998649	cg14826792	TTPA	TSS200	chr8:63998227-63998737	Island
hyper766	FGR_excessive_1	0.0E+00	0.29	0.02	0.27	8	63998667	cg13682477	TTPA	TSS200	chr8:63998227-63998737	Island
hyper767	FGR_excessive_1	0.0E+00	0.22	0.01	0.21	8	63998731	cg10815543	TTPA	TSS200	chr8:63998227-63998737	Island
hyper768	FGR_excessive_1	0.0E+00	0.28	0.07	0.21	8	63998736	cg16231500	TTPA	TSS200	chr8:63998227-63998737	Island
hyper769	FGR_excessive_1	1.3E-08	0.19	0.06	0.13	8	63998778	cg14817143	TTPA	TSS200	chr8:63998227-63998737	S_Shore

hyper770	FGR_excessive_1	4.7E-06	0.16	0.05	0.11	8	63999003	cg04163863	TTPA	TSS1500	chr8:63998227-63998737	S_Shore
hyper771	FGR_excessive_1	4.7E-02	0.18	0.06	0.11	8	75234078	cg03060802	JPH1	TSS1500	chr8:75232649-75234101	Island
hyper772	FGR_excessive_1	1.5E-04	0.44	0.22	0.22	9	114287300	cg13570660	ZNF483;ZNF483	TSS200;TSS200	chr9:114287380-114287695	N_Shore
hyper773	FGR_excessive_1	9.7E-10	0.24	0.07	0.17	9	114287383	cg02612963	ZNF483;ZNF483	TSS200;TSS200	chr9:114287380-114287695	Island
hyper774	FGR_excessive_1	0.0E+00	0.31	0.03	0.29	10	22540745	cg16663900			chr10:22540707-22542739	Island
hyper775	FGR_excessive_1	2.1E-06	0.23	0.06	0.17	10	22540824	cg16782935			chr10:22540707-22542739	Island
hyper776	FGR_excessive_1	1.6E-08	0.30	0.05	0.25	10	22541366	cg03167951			chr10:22540707-22542739	Island
hyper777	FGR_excessive_1	1.2E-07	0.19	0.07	0.12	10	52751119	cg26337884	PRKG1;PRKG1	1stExon;5'UTR	chr10:52750956-52751765	Island
hyper778	FGR_excessive_1	0.0E+00	0.17	0.01	0.17	10	52751341	cg05603630	PRKG1	1stExon	chr10:52750956-52751765	Island
hyper779	FGR_excessive_1	1.5E-05	0.17	0.06	0.11	10	64133888	cg01137532	ZNF365;ZNF365;ZNF365	TSS200;TSS200;TSS200	chr10:64133789-64134420	Island
hyper780	FGR_excessive_1	3.7E-02	0.09	0.02	0.07	10	94352821	cg00519463	KIF11	TSS200	chr10:94352667-94353159	Island
hyper781	FGR_excessive_1	7.3E-02	0.18	0.05	0.13	11	10324369	cg14750263			chr11:10324353-10324828	Island
hyper782	FGR_excessive_1	5.8E-07	0.24	0.02	0.22	11	93063692	cg18786535	CCDC67	TSS200	chr11:93063598-93064076	Island
hyper783	FGR_excessive_1	1.1E-03	0.23	0.02	0.21	12	7023515	cg22977254	ENO2	TSS200	chr12:7023261-7024089	Island
hyper784	FGR_excessive_1	5.1E-06	0.19	0.07	0.12	12	21680409	cg05161082	C12orf39	Body	chr12:21680408-21680982	Island
hyper785	FGR_excessive_1	1.1E-03	0.16	0.04	0.13	12	21680426	cg08347047	C12orf39	Body	chr12:21680408-21680982	Island
hyper786	FGR_excessive_1	2.5E-08	0.11	0.02	0.09	12	21680508	cg10437806	C12orf39	Body	chr12:21680408-21680982	Island
hyper787	FGR_excessive_1	2.5E-04	0.26	0.09	0.18	12	21681006	cg08828403	C12orf39	Body	chr12:21680408-21680982	S_Shore
hyper788	FGR_excessive_1	1.0E-06	0.20	0.03	0.17	12	66583048	cg26415547	IRAK3;IRAK3;IRAK3;IRAK3	1stExon;1stExon;5'UTR;5'UTR	chr12:66582695-66583345	Island
hyper789	FGR_excessive_1	2.5E-03	0.29	0.03	0.26	12	66583203	cg10389229	IRAK3;IRAK3	1stExon;1stExon	chr12:66582695-66583345	Island
hyper790	FGR_excessive_1	4.3E-02	0.25	0.09	0.16	12	85306474	cg14449051	SLC6A15;SLC6A15;SLC6A15;SL(	1stExon;5'UTR;5'UTR;1stExon;1st	chr12:85306282-85306829	Island
hyper791	FGR_excessive_1	2.4E-04	0.33	0.08	0.25	12	96183791	cg25253534	NTN4	Body	chr12:96183873-96185199	N_Shore
hyper792	FGR_excessive_1	2.7E-02	0.49	0.06	0.43	12	96184020	cg16829450	NTN4	Body	chr12:96183873-96185199	Island
hyper793	FGR_excessive_1	1.1E-02	0.47	0.04	0.44	12	96184558	cg15367106	NTN4	TSS200	chr12:96183873-96185199	Island
hyper794	FGR_excessive_1	7.7E-05	0.45	0.13	0.31	12	96184787	cg22828900	NTN4	TSS1500	chr12:96183873-96185199	Island
hyper795	FGR_excessive_1	3.3E-05	0.18	0.06	0.12	12	96184965	cg07461318	NTN4	TSS1500	chr12:96183873-96185199	Island
hyper796	FGR_excessive_1	9.7E-04	0.20	0.06	0.14	12	96185437	cg03100449	NTN4	TSS1500	chr12:96183873-96185199	S_Shore
hyper797	FGR_excessive_1	2.9E-03	0.05	0.01	0.04	12	109251281	cg10817500	SSH1;SSH1;SSH1;SSH1	5'UTR;5'UTR;1stExon;1stExon	chr12:109251205-109251586	Island
hyper798	FGR_excessive_1	7.4E-07	0.28	0.09	0.19	13	25744676	cg18815647	FAM123A;FAM123A	Body;1stExon	chr13:25743998-25746127	Island
hyper799	FGR_excessive_1	0.0E+00	0.17	0.01	0.16	14	52535758	cg13592399	NID2;NID2	5'UTR;1stExon	chr14:52534581-52536722	Island
hyper800	FGR_excessive_1	0.0E+00	0.28	0.06	0.22	14	52535887	cg01231779	NID2;NID2	5'UTR;1stExon	chr14:52534581-52536722	Island
hyper801	FGR_excessive_1	6.0E-08	0.22	0.09	0.13	14	52535949	cg16066272	NID2	TSS200	chr14:52534581-52536722	Island
hyper802	FGR_excessive_1	9.7E-11	0.26	0.08	0.18	14	52535953	cg10149976	NID2	TSS200	chr14:52534581-52536722	Island
hyper803	FGR_excessive_1	1.9E-03	0.20	0.08	0.12	14	52535964	cg06782686	NID2	TSS200	chr14:52534581-52536722	Island
hyper804	FGR_excessive_1	7.0E-06	0.18	0.04	0.14	14	52535973	cg01781974	NID2	TSS200	chr14:52534581-52536722	Island
hyper805	FGR_excessive_1	4.3E-05	0.25	0.12	0.13	14	52536022	cg00766845	NID2	TSS200	chr14:52534581-52536722	Island
hyper806	FGR_excessive_1	0.0E+00	0.42	0.06	0.35	14	52536147	cg22881914	NID2	TSS1500	chr14:52534581-52536722	Island
hyper807	FGR_excessive_1	9.1E-07	0.36	0.03	0.32	14	52536175	cg04701034	NID2	TSS1500	chr14:52534581-52536722	Island
hyper808	FGR_excessive_1	7.3E-02	0.32	0.11	0.21	14	76842507	cg09329564	ESRRB	5'UTR	chr14:76842506-76843846	Island
hyper809	FGR_excessive_1	1.3E-03	0.33	0.11	0.22	14	90849959	cg06027057			chr14:90848994-90850381	Island
hyper810	FGR_excessive_1	3.8E-03	0.10	0.01	0.09	14	102026086	cg16366843			chr14:102025989-102031567	Island
hyper811	FGR_excessive_1	2.2E-03	0.63	0.24	0.38	15	63034966	cg13821077	TLN2	Body		
hyper812	FGR_excessive_1	4.6E-07	0.13	0.02	0.11	16	56623312	cg16393726	MT3;MT3	5'UTR;1stExon	chr16:56623068-56623914	Island
hyper813	FGR_excessive_1	3.6E-09	0.24	0.04	0.20	16	80574553	cg07732115	DYNLRB2	TSS1500	chr16:80574741-80575090	N_Shore
hyper814	FGR_excessive_1	3.4E-03	0.17	0.05	0.11	16	80574580	cg02560638	DYNLRB2	TSS1500	chr16:80574741-80575090	N_Shore
hyper815	FGR_excessive_1	3.1E-04	0.21	0.03	0.19	16	80574757	cg27254295	DYNLRB2	TSS200	chr16:80574741-80575090	Island
hyper816	FGR_excessive_1	0.0E+00	0.34	0.01	0.32	16	80574926	cg06395091	DYNLRB2;DYNLRB2	1stExon;5'UTR	chr16:80574741-80575090	Island
hyper817	FGR_excessive_1	1.8E-05	0.36	0.07	0.29	16	80575018	cg00720137	DYNLRB2	Body	chr16:80574741-80575090	Island
hyper818	FGR_excessive_1	1.6E-04	0.36	0.07	0.29	17	11144509	cg18160402	SHISA6	TSS1500	chr17:11143487-11145416	Island
hyper819	FGR_excessive_1	0.0E+00	0.19	0.01	0.17	17	80477962	cg18242682	FOXK2	1stExon	chr17:80477098-80478553	Island
hyper820	FGR_excessive_1	1.4E-06	0.21	0.09	0.12	18	12076435	cg02272280			chr18:12076434-12076799	Island
hyper821	FGR_excessive_1	1.9E-08	0.24	0.07	0.17	19	24269890	cg09777776	ZNF254	TSS200		
hyper822	FGR_excessive_1	0.0E+00	0.30	0.03	0.27	19	24270007	cg04571847	ZNF254;ZNF254	1stExon;5'UTR		
hyper823	FGR_excessive_1	2.8E-08	0.31	0.09	0.22	19	24270073	cg02286642	ZNF254;ZNF254	1stExon;5'UTR		
hyper824	FGR_excessive_1	2.8E-03	0.16	0.03	0.13	19	24270468	cg01000657	ZNF254	Body		
hyper825	FGR_excessive_1	7.2E-04	0.25	0.06	0.19	19	36478250	cg18812980			chr19:36478097-36478330	Island
hyper826	FGR_excessive_1	6.9E-08	0.29	0.08	0.21	19	51831274	cg08874512	IGLON5	Body	chr19:51830064-51831145	S_Shore
hyper827	FGR_excessive_1	1.9E-10	0.28	0.10	0.18	19	51843601	cg03352428	VSIG10L	Body	chr19:51843075-51843926	Island
hyper828	FGR_excessive_1	0.0E+00	0.43	0.05	0.39	19	51843854	cg18563413	VSIG10L	Body	chr19:51843075-51843926	Island
hyper829	FGR_excessive_1	8.9E-08	0.24	0.09	0.15	19	51869353	cg10219093	ETFB	Body	chr19:51869400-51871587	N_Shore
hyper830	FGR_excessive_1	1.6E-02	0.22	0.08	0.13	19	51869678	cg08854169	ETFB	TSS200	chr19:51869400-51871587	Island
hyper831	FGR_excessive_1	0.0E+00	0.22	0.01	0.21	19	51869680	cg02666184	ETFB	TSS200	chr19:51869400-51871587	Island
hyper832	FGR_excessive_1	1.1E-05	0.18	0.06	0.11	19	51869687	cg20367072	ETFB	TSS200	chr19:51869400-51871587	Island
hyper833	FGR_excessive_1	0.0E+00	0.27	0.06	0.21	19	51870582	cg19319774	CLDND2;ETFB	Body;TSS1500	chr19:51869400-51871587	Island
hyper834	FGR_excessive_1	0.0E+00	0.31	0.06	0.25	19	51870611	cg07757224	CLDND2;ETFB	Body;TSS1500	chr19:51869400-51871587	Island
hyper835	FGR_excessive_1	0.0E+00	0.20	0.04	0.16	19	51870714	cg01226447	CLDND2;ETFB	Body;TSS1500	chr19:51869400-51871587	Island
hyper836	FGR_excessive_1	0.0E+00	0.33	0.10	0.24	19	51871466	cg05562817	CLDND2	Body	chr19:51869400-51871587	Island
hyper837	FGR_excessive_1	2.9E-03	0.21	0.05	0.17	20	10153355	cg23022785			chr20:10152768-10153193	S_Shore
hyper838	FGR_excessive_1	6.9E-02	0.18	0.03	0.15	20	32803935	cg21747188			chr20:32803823-32804181	Island
hyper839	FGR_excessive_1	1.4E-02	0.33	0.12	0.21	20	57268419	cg10073706	NPEPL1	Body	chr20:57267527-57268420	Island
hyper840	FGR_excessive_1	1.1E-04	0.16	0.05	0.11	22	42062693	cg10474712			chr22:42062790-42063002	N_Shore
hyper841	FGR_excessive_1	1.8E-07	0.19	0.06	0.13	22	42062791	cg11013544			chr22:42062790-42063002	Island
hyper842	FGR_excessive_1	2.1E-07	0.18	0.06	0.12	22	42062938	cg14458955			chr22:42062790-42063002	Island
hyper843	FGR_excessive_1	6.4E-05	0.15	0.05	0.09	22	42063028	cg10077672			chr22:42062790-42063002	S_Shore
hyper844	FGR_excessive_1	7.0E-02	0.23	0.04	0.18	22	46263165	cg22669623			chr22:46262065-46263747	Island
hyper845	FGR_excessive_2	9.1E-04	0.15	0.06	0.08	1	6052177	cg07420961	NPHP4	5'UTR	chr1:6051595-6052955	Island
hyper846	FGR_excessive_2	0.0E+00	0.25	0.01	0.25	1	84764278	cg24002839	SAMD13	Body	chr1:84764108-84764906	Island
hyper847	FGR_excessive_2	1.3E-09	0.25	0.07	0.17	1	84764375	cg16026299	SAMD13	Body	chr1:84764108-84764906	Island
hyper848	FGR_excessive_2	7.3E-02	0.14	0.06	0.08	1	165567358	cg16599568			chr1:165567265-165567530	Island
hyper849	FGR_excessive_2	9.7E-11	0.37	0.09	0.28	1	182758324	cg27583088			chr1:182758395-182759097	N_Shore
hyper850	FGR_excessive_2	0.0E+00	0.46	0.07	0.39	1	182758421	cg03994069			chr1:182758395-182759097	Island
hyper851	FGR_excessive_2	0.0E+00	0.39	0.12	0.27	1	182758699	cg06676233			chr1:182758395-182759097	Island
hyper852	FGR_excessive_2	0.0E+00	0.23	0.02	0.22	1	182758764	cg14844172			chr1:182758395-182759097	Island
hyper853	FGR_excessive_2	0.0E+00	0.35	0.01	0.34	1	182758792	cg07202210			chr1:182758395-182759097	Island
hyper854	FGR_excessive_2	0.0E+00	0.42	0.02	0.40	1	182758953	cg13559225			chr1:182758395-182759097	Island
hyper855	FGR_excessive_2	0.0E+00	0.39	0.06	0.33	1	222817468	cg08484944	MIA3	Body	chr1:222817467-222817671	Island
hyper856	FGR_excessive_2	0.0E+00	0.37	0.08	0.29	1	222817471	cg13088003	MIA3	Body	chr1:222817467-222817671	Island
hyper857	FGR_excessive_2	2.1E-02	0.26	0.13	0.13	1	232941055	cg02903907	KIAA1383	1stExon	chr1:232941054-232941707	Island
hyper858	FGR_excessive_2	5.7E-09	0.09	0.02	0.08	1	232941253	cg15542798	KIAA1383	1stExon	chr1:232941054-232941707	Island
hyper859	FGR_excessive_2	1.9E-08	0.09	0.02	0.08	1	234509341	cg12140468	C1orf31	TSS200	chr1:234508994-234509501	Island
hyper860	FGR_excessive_2	7.9E-02	0.11	0.05	0.05	2	120981274	cg07988989	TMEM185B	TSS1500	chr2:120980332-120981451	Island
hyper861	FGR_excessive_2	8.2E-05	0.24	0.04	0.20	2	160143690	cg14825887	WDSUB1;WDSUB1;WDSUB1	TSS1500;TSS1500;TSS1500	chr2:160142607-160143567	S_Shore
hyper862	FGR_excessive_2	2.0E-02	0.32	0.09	0.22	3	24563068	cg21904937			chr3:24562863-24563576	Island
hyper863	FGR_excessive_2	1.4E-04	0.26	0.02	0.24	3	24563278	cg22797570			chr3:24562863-24563576	Island
hyper864	FGR_excessive_2	1.6E-06	0.22	0.02	0.20	3	24563497	cg01442844			chr3:24562863-24563576	Island
hyper865	FGR_excessive_2	1.5E-03	0.13	0.02	0.11	4	4854690	cg17807131			chr4:4854613-4855210	Island
hyper866	FGR_excessive_2	0.0E+00	0.43	0.10	0.32	5	36241190	cg14725858	C5orf33;C5orf33	5'UTR;Body	chr5:36241003-36242664	Island
hyper867	FGR_excessive_2	4.1E-03	0.19	0.06	0.13	5	160974086	cg18643445	GABRB2;GABRB2	5'UTR;5'UTR	chr5:160974692-160975384	N_Shore
hyper868	FGR_excessive_2	1.2E-05	0.09	0.01	0.08	6	1312511	cg10853637	FOXQ1	TSS200	chr6:1311068-1314220	Island
hyper869	FGR_excessive_2	4.8E-02	0.23	0.07	0.15	6	1312517	cg10125626	FOXQ1	TSS200	chr6:1311068-1314220	Island
hyper870	FGR_excessive_2	2.9E-06	0.13	0.05	0.08	7	33944213	cg10073723	BMPER	TSS1500	chr7:33943665-33945509	Island
hyper871	FGR_excessive_2	5.4E-08	0.15	0.02	0.13	7	43798100	cg01745690	BLVRA	TSS200	chr7:43797823-43798802	Island
hyper872	FGR_excessive_2	9.3E-04	0.19	0.07	0.12	8	13424135	cg06448090	C8orf48	TSS1500		
hyper873	FGR_excessive_2	8.0E-07	0.18	0.04	0.14	8	104153148	cg06399148	BAALC;BAALC;C8orf56	1stExon;1stExon;Body	chr8:104152726-104153390	Island
hyper874	FGR_excessive_2	1.5E-07	0.15	0.07	0.08	8	104153173	cg10918202	BAALC;BAALC;C8orf56	1stExon;1stExon;Body	chr8:104152726-104153390	Island
hyper875	FGR_excessive_2	5.4E-08	0.18	0.04	0.14	9	100000768	cg01950665	KIAA1529	TSS200	chr9:100000463-100000820	Island
hyper876	FGR_excessive_2	5.1E-05	0.16	0.04	0.12	9	123605234	cg13460057	PSMD5;LOC253039	TSS200;TSS200	chr9:123605043-123605585	Island
hyper877	FGR_excessive_2	0.0E+00	0.56	0.03	0.53	10	98956178	cg26811352			chr10:98955994-98956285	Island
hyper878	FGR_excessive_2	1.0E-02	0.06	0.01	0.05	10	134901689	cg19396601	GPR123	5'UTR	chr10:134901198-134902619	Island
hyper879	FGR_excessive_2	1.4E-04	0.12	0.02	0.09	11	44341646	cg19520927			chr11:44341265-44341757	Island
hyper880	FGR_excessive_2	5.7E-03	0.04	0.01	0.03	11	76777993	cg08103551	CAPN5;CAPN5	1stExon;5'UTR	chr11:76777569-76778453	Island
hyper881	FGR_excessive_2	7.2E-02	0.23	0.03	0.20	13	26625766	cg16572375	SHISA2	TSS1500	chr13:26624725-26626265	Island
hyper882	FGR_excessive_2	1.1E-04	0.16	0.05	0.11	13	96204917	cg09469554	CLDN10;CLDN10;CLDN10	TSS200;Body;Body	chr13:96204691-96205496	Island
hyper883	FGR_excessive_2	5.3E-04	0.16	0.02	0.14	13	96204978	cg25032595	CLDN10;CLDN10;CLDN10;CLDN	5'UTR;1stExon;Body;Body	chr13:96204691-96205496	Island
hyper884	FGR_excessive_2	1.6E-04	0.48	0.24	0.25	14	101111348	cg16064425				
hyper885	FGR_excessive_2	1.9E-10	0.21	0.05	0.16	16	3507735	cg07537798	NAT15;NAT15;NAT15	TSS1500;5'UTR;TSS1500	chr16:3507545-3508606	Island
hyper886	FGR_excessive_2	1.9E-10	0.34	0.05	0.29	16	3508542	cg14187965	NAT15;NAT15;NAT15	5'UTR;5'UTR;5'UTR	chr16:3507545-3508606	Island
hyper887	FGR_excessive_2	0.0E+00	0.32	0.05	0.27	16	3508546	cg03831869	NAT15;NAT15;NAT15	5'UTR;5'UTR;5'UTR	chr16:3507545-3508606	Island
hyper888	FGR_excessive_2	2.4E-05	0.48	0.13	0.36	18	47792708	cg24041453	CCDC11	1stExon	chr18:47792742-47793036	N_Shore
hyper889	FGR_excessive_2	0.0E+00	0.39	0.05	0.34	18	47792794	cg27392024	CCDC11;CCDC11	1stExon;5'UTR	chr18:47792742-47793036	Island
hyper890	FGR_excessive_2	0.0E+00	0.29	0.01	0.29	18	47792917	cg18234979	CCDC11	TSS200	chr18:47792742-47793036	Island
hyper891	FGR_excessive_2	7.2E-09	0.36	0.04	0.33	18	47792928	cg24391240	CCDC11	TSS200	chr18:47792742-47793036	Island
hyper892	FGR_excessive_2	0.0E+00	0.32	0.01	0.31	18	47792949	cg06565620	CCDC11	TSS200	chr18:47792742-47793036	Island
hyper893	FGR_excessive_2	0.0E+00	0.33	0.09	0.24	18	47793000	cg08633810	CCDC11	TSS200	chr18:47792742-47793036	Island
hyper894	FGR_excessive_2	6.9E-02	0.04	0.01	0.03	18	60987770	cg07111678	BCL2;BCL2	TSS1500;TSS1500	chr18:60986621-60988286	Island
hyper895	FGR_excessive_2	3.9E-05	0.12	0.02	0.10	19	33864973	cg20000099	CEBPG	5'UTR	chr19:33864176-33865010	Island
hyper896	FGR_excessive_2	0.0E+00	0.55	0.06	0.49	19	52390810	cg06878361	ZNF577;ZNF577;ZNF577	Body;5'UTR;5'UTR	chr19:52390841-52391368	N_Shore
hyper897	FGR_excessive_2	0.0E+00	0.51	0.07	0.45	19	52391078	cg03562414	ZNF577;ZNF577;ZNF577	Body;5'UTR;5'UTR	chr19:52390841-52391368	Island
hyper898	FGR_excessive_2	0.0E+00	0.49	0.05	0.43	19	52391090	cg10635122	ZNF577;ZNF577;ZNF577;ZNF577	Body;5'UTR;5'UTR;1stExon	chr19:52390841-52391368	Island

hyper899	FGR_excessive_2	0.0E+00	0.42	0.01	0.41	19	52391166	cg24794228	ZNF577;ZNF577;ZNF577;ZNF577	Body;5'UTR;5'UTR;1stExon;1stEx	chr19:52390841-52391368	Island
hyper900	FGR_excessive_2	0.0E+00	0.49	0.05	0.45	19	52391304	cg11269599	ZNF577;ZNF577;ZNF577	TSS200;TSS200;TSS200	chr19:52390841-52391368	Island
hyper901	FGR_excessive_2	9.7E-11	0.42	0.09	0.33	19	52391350	cg22331349	ZNF577;ZNF577;ZNF577	TSS200;TSS200;TSS200	chr19:52390841-52391368	Island
hyper902	FGR_excessive_2	6.4E-05	0.18	0.06	0.12	21	39288941	cg23909013	KCNJ6	TSS1500	chr21:39287946-39289267	Island
hyper903	FGR_excessive_2	9.1E-03	0.15	0.06	0.09	21	47010461	cg02983163			chr21:47009996-47011163	Island
hyper904	FGR_excessive_3	3.7E-03	0.54	0.21	0.33	1	15242180	cg17596058	KIAA1026;KIAA1026	Body;Body		
hyper905	FGR_excessive_3	8.8E-02	0.30	0.08	0.23	1	110453914	cg22621745	CSF1;CSF1;CSF1;CSF1	Body;Body;Body;Body	chr1:110453143-110453889	S_Shore
hyper906	FGR_excessive_3	4.4E-03	0.22	0.06	0.16	1	160001329	cg24523950	PIGM	1stExon	chr1:160001295-160001989	Island
hyper907	FGR_excessive_3	0.0E+00	0.36	0.01	0.35	2	191045309	cg08350814	C2orf88;C2orf88;C2orf88;C2orf88	TSS1500;5'UTR;TSS1500;5'UTR	chr2:191044979-191045829	Island
hyper908	FGR_excessive_3	7.9E-07	0.19	0.02	0.17	2	191045430	cg06532037	C2orf88;C2orf88;C2orf88;C2orf88	5'UTR;TSS1500;TSS200;5'UTR	chr2:191044979-191045829	Island
hyper909	FGR_excessive_3	0.0E+00	0.43	0.10	0.33	2	191045632	cg14210694	C2orf88;C2orf88;C2orf88;C2orf88;	5'UTR;5'UTR;1stExon;5'UTR;TSS:	chr2:191044979-191045829	Island
hyper910	FGR_excessive_3	0.0E+00	0.45	0.13	0.31	2	191045635	cg11097717	C2orf88;C2orf88;C2orf88;C2orf88;	5'UTR;5'UTR;1stExon;5'UTR;TSS:	chr2:191044979-191045829	Island
hyper911	FGR_excessive_3	0.0E+00	0.38	0.02	0.36	2	191045668	cg15313226	C2orf88;C2orf88;C2orf88;C2orf88;	5'UTR;5'UTR;1stExon;5'UTR;TSS:	chr2:191044979-191045829	Island
hyper912	FGR_excessive_3	8.0E-06	0.39	0.14	0.25	2	191045697	cg19906093	C2orf88;C2orf88;C2orf88;C2orf88;	5'UTR;1stExon;5'UTR;5'UTR;1stE:	chr2:191044979-191045829	Island
hyper913	FGR_excessive_3	1.9E-10	0.24	0.05	0.19	2	191045885	cg22003435	C2orf88;C2orf88;C2orf88;C2orf88	5'UTR;5'UTR;5'UTR;5'UTR	chr2:191044979-191045829	S_Shore
hyper914	FGR_excessive_3	6.3E-07	0.29	0.09	0.21	3	89164038	cg05475667	EPHA3;EPHA3	Body;Body	chr3:89163635-89164107	Island
hyper915	FGR_excessive_3	1.3E-08	0.16	0.02	0.14	4	42659536	cg24533989	ATP8A1;ATP8A1	TSS1500;TSS1500	chr4:42658278-42659550	Island
hyper916	FGR_excessive_3	3.7E-02	0.25	0.11	0.14	5	95297364	cg10774586	ELL2	1stExon	chr5:95296368-95297438	Island
hyper917	FGR_excessive_3	4.3E-04	0.46	0.25	0.21	5	139554307	cg05598363	C5orf32	TSS1500	chr5:139554389-139555457	N_Shore
hyper918	FGR_excessive_3	2.6E-09	0.17	0.05	0.13	5	139554488	cg22434226	C5orf32	TSS200	chr5:139554389-139555457	Island
hyper919	FGR_excessive_3	4.0E-02	0.47	0.09	0.38	7	99155739	cg05165378	ZNF655;ZNF655;ZNF655;ZNF655	TSS1500;TSS1500;TSS1500;TSS	chr7:99155871-99156969	N_Shore
hyper920	FGR_excessive_3	3.0E-02	0.36	0.04	0.32	7	99155767	cg21950525	ZNF655;ZNF655;ZNF655;ZNF655	TSS1500;TSS1500;TSS1500;TSS	chr7:99155871-99156969	N_Shore
hyper921	FGR_excessive_3	2.3E-02	0.23	0.05	0.18	7	99156011	cg07121182	ZNF655;ZNF655;ZNF655;ZNF655	TSS200;TSS1500;TSS1500;TSS2	chr7:99155871-99156969	Island
hyper922	FGR_excessive_3	1.5E-05	0.61	0.08	0.53	9	140512455	cg14498394	C9orf37;EHMT1;EHMT1	5'UTR;TSS1500;TSS1500	chr9:140512892-140513420	N_Shore
hyper923	FGR_excessive_3	2.3E-09	0.36	0.10	0.26	11	22646597	cg03794550	FANCF	1stExon	chr11:22646706-22647441	N_Shore
hyper924	FGR_excessive_3	3.0E-07	0.22	0.08	0.14	11	22646931	cg00908551	FANCF	1stExon	chr11:22646706-22647441	Island
hyper925	FGR_excessive_4	3.3E-03	0.06	0.02	0.05	1	1407183	cg04286729	ATAD3B;ATAD3B	1stExon;5'UTR	chr1:1406844-1407821	Island
hyper926	FGR_excessive_4	6.7E-02	0.11	0.04	0.07	1	196137378	ch.1.194404001R				
hyper927	FGR_excessive_4	3.0E-03	0.29	0.03	0.26	1	197881540	cg13501359	LHX9	TSS200	chr1:197880016-197881637	Island
hyper928	FGR_excessive_4	4.6E-03	0.33	0.09	0.24	2	79738639	cg22350070	CTNNA2;CTNNA2	TSS1500;TSS1500	chr2:79739696-79740243	N_Shore
hyper929	FGR_excessive_4	4.7E-02	0.54	0.21	0.33	2	82120996	cg01296927				
hyper930	FGR_excessive_4	6.9E-04	0.23	0.12	0.11	2	223184510	cg16975959			chr2:223183013-223185468	Island
hyper931	FGR_excessive_4	3.2E-08	0.21	0.07	0.14	2	223184557	cg27506462			chr2:223183013-223185468	Island
hyper932	FGR_excessive_4	9.1E-03	0.09	0.03	0.06	2	233497957	cg26148236	EFHD1;EFHD1	TSS1500;Body	chr2:233497826-233499727	Island
hyper933	FGR_excessive_4	1.2E-03	0.15	0.05	0.09	6	32952826	cg25435686				
hyper934	FGR_excessive_4	4.6E-04	0.12	0.06	0.06	6	150184345	cg24761195	LRP11	Body	chr6:150184344-150185505	Island
hyper935	FGR_excessive_4	1.7E-07	0.16	0.06	0.11	6	160211790	cg10634217	TCP1;TCP1;MRPL18	TSS1500;TSS1500;Body	chr6:160209941-160212015	Island
hyper936	FGR_excessive_4	1.8E-04	0.18	0.07	0.10	6	160212014	cg16346065	TCP1;TCP1;MRPL18	TSS1500;TSS1500;Body	chr6:160209941-160212015	Island
hyper937	FGR_excessive_4	1.5E-02	0.21	0.10	0.11	6	160212070	cg22313322	TCP1;TCP1;MRPL18	TSS1500;TSS1500;Body	chr6:160209941-160212015	S_Shore
hyper938	FGR_excessive_4	1.6E-02	0.31	0.10	0.21	7	25989618	cg07184013	MIR148A	TSS200	chr7:25990012-25991320	N_Shore
hyper939	FGR_excessive_4	8.6E-07	0.43	0.08	0.34	7	25989735	cg25771013	MIR148A	TSS200	chr7:25990012-25991320	N_Shore
hyper940	FGR_excessive_4	3.9E-06	0.44	0.12	0.32	7	25989763	cg03853208	MIR148A	TSS200	chr7:25990012-25991320	N_Shore
hyper941	FGR_excessive_4	2.1E-09	0.38	0.07	0.31	7	25990013	cg16291657	MIR148A	TSS1500	chr7:25990012-25991320	Island
hyper942	FGR_excessive_4	0.0E+00	0.44	0.08	0.35	7	25990026	cg09596116	MIR148A	TSS1500	chr7:25990012-25991320	Island
hyper943	FGR_excessive_4	0.0E+00	0.55	0.07	0.48	7	25990679	cg12693702	MIR148A	TSS1500	chr7:25990012-25991320	Island
hyper944	FGR_excessive_4	0.0E+00	0.28	0.05	0.23	7	25990806	cg00399596	MIR148A	TSS1500	chr7:25990012-25991320	Island
hyper945	FGR_excessive_4	2.5E-07	0.06	0.01	0.05	7	25991025	cg18188200	MIR148A	TSS1500	chr7:25990012-25991320	Island
hyper946	FGR_excessive_4	2.8E-08	0.11	0.02	0.09	7	25991027	cg16872086	MIR148A	TSS1500	chr7:25990012-25991320	Island
hyper947	FGR_excessive_4	7.0E-09	0.27	0.08	0.19	7	25991107	cg15263946			chr7:25990012-25991320	Island
hyper948	FGR_excessive_4	5.0E-02	0.09	0.04	0.04	7	100807410	cg14814333	VGF	Body	chr7:100806279-100809064	Island
hyper949	FGR_excessive_4	2.3E-03	0.30	0.07	0.23	8	104310713	cg24115571	FZD6;FZD6;FZD6;FZD6	TSS1500;TSS1500;5'UTR;1stExo	chr8:104310809-104311620	N_Shore
hyper950	FGR_excessive_4	3.8E-02	0.44	0.06	0.38	8	104310886	cg00487870	FZD6;FZD6;FZD6	TSS200;5'UTR;TSS200	chr8:104310809-104311620	Island
hyper951	FGR_excessive_4	2.0E-03	0.06	0.01	0.04	9	140195998	cg13897564	NRARP;NRARP	1stExon;3'UTR	chr9:140195501-140197475	Island
hyper952	FGR_excessive_4	1.2E-03	0.15	0.06	0.09	13	31736577	cg12292402	HSPH1	TSS1500	chr13:31735109-31736670	Island
hyper953	FGR_excessive_4	3.8E-06	0.32	0.08	0.24	13	31736727	cg04459413	HSPH1	TSS1500	chr13:31735109-31736670	S_Shore
hyper954	FGR_excessive_4	2.6E-02	0.39	0.10	0.29	13	102069296	cg11621911	NALCN	TSS1500	chr13:102068116-102069258	S_Shore
hyper955	FGR_excessive_4	2.3E-02	0.33	0.09	0.24	16	31724470	cg22819888	ZNF720	TSS200	chr16:31724651-31725071	N_Shore
hyper956	FGR_excessive_4	1.0E-02	0.47	0.04	0.43	16	31725053	cg04747728	ZNF720	Body	chr16:31724651-31725071	Island
hyper957	FGR_excessive_4	1.4E-04	0.18	0.05	0.13	19	1020409	cg04169328	C19orf6;C19orf6	Body;Body	chr19:1020511-1021395	N_Shore
hyper958	FGR_excessive_4	7.2E-05	0.28	0.05	0.23	19	42681512	cg01290613			chr19:42681304-42681513	Island
hyper959	FGR_excessive_4	1.7E-02	0.05	0.02	0.03	19	49014774	cg26739378	LMTK3	Body	chr19:49014611-49014940	Island
hyper960	FGR_excessive_4	2.0E-02	0.37	0.05	0.32	20	9819697	cg02167438	PAK7;PAK7	TSS200;TSS200	chr20:9819271-9819861	Island
hyper961	FGR_excessive_4	2.0E-08	0.23	0.07	0.16	20	33297179	cg01118078	TP53INP2	Body	chr20:33296514-33298242	Island
hyper962	FGR_excessive_4	3.3E-04	0.22	0.09	0.13	20	33297218	cg26204638	TP53INP2	Body	chr20:33296514-33298242	Island
hyper963	FGR_excessive_4	2.7E-02	0.03	0.01	0.02	20	62339231	cg16400469	ARFRP1;ZGPAT;ZGPAT;ARFRP1	5'UTR;TSS200;5'UTR;1stExon;1st	chr20:62338659-62340274	Island
hyper964	FGR_excessive_4	1.7E-02	0.24	0.03	0.22	20	62496660	cg27656320	TPD52L2;TPD52L2;TPD52L2;TPD	5'UTR;1stExon;5'UTR;5'UTR;1stE:	chr20:62496243-62497196	Island
hyper965	FGR_excessive_4	1.7E-06	0.10	0.02	0.08	22	24093429	cg18803171	ZNF70	TSS200	chr22:24093026-24093621	Island
hyper966	N_insufficient_1	0.0E+00	0.53	0.07	0.46	1	567747	cg18024856	MIR1977	TSS1500		
hyper967	N_insufficient_1	2.1E-02	0.04	0.01	0.03	1	4716537	cg02483484	AJAP1;AJAP1	Body;Body	chr1:4713989-4716555	Island
hyper968	N_insufficient_1	5.3E-07	0.28	0.10	0.18	1	9242937	cg03521358			chr1:9242167-9243827	Island
hyper969	N_insufficient_1	3.3E-05	0.24	0.08	0.16	1	9242956	cg06658816			chr1:9242167-9243827	Island
hyper970	N_insufficient_1	3.5E-02	0.31	0.07	0.24	1	13910667	cg05140069	PDPN;PDPN;PDPN;PDPN	TSS1500;TSS1500;Body;Body	chr1:13910137-13910868	Island
hyper971	N_insufficient_1	9.8E-07	0.17	0.03	0.14	1	39269756	cg06546406			chr1:39269729-39270251	Island
hyper972	N_insufficient_1	2.6E-06	0.21	0.06	0.15	1	39269995	cg21848143			chr1:39269729-39270251	Island
hyper973	N_insufficient_1	3.0E-02	0.17	0.10	0.07	1	39270050	cg04267214			chr1:39269729-39270251	Island
hyper974	N_insufficient_1	7.1E-08	0.24	0.06	0.18	1	39270149	cg17834155			chr1:39269729-39270251	Island
hyper975	N_insufficient_1	9.6E-04	0.17	0.08	0.09	1	40157480	cg01775082	HPCAL4	TSS1500	chr1:40156930-40158514	Island
hyper976	N_insufficient_1	6.0E-03	0.18	0.05	0.14	1	45956974	cg00609097	TESK2	TSS200	chr1:45956345-45956893	S_Shore
hyper977	N_insufficient_1	2.1E-02	0.11	0.01	0.10	1	47697948	cg25764899			chr1:47696504-47698197	Island
hyper978	N_insufficient_1	2.2E-03	0.19	0.07	0.12	1	59248148	cg15156614	JUN	1stExon	chr1:59247461-59250940	Island
hyper979	N_insufficient_1	0.0E+00	0.18	0.01	0.18	1	63790044	cg18279094	FOXD3	1stExon	chr1:63782394-63790471	Island
hyper980	N_insufficient_1	4.5E-02	0.11	0.06	0.06	1	65533830	cg09473383			chr1:65533592-65533965	Island
hyper981	N_insufficient_1	6.9E-04	0.32	0.12	0.20	1	71512973	cg15201877	PTGER3;PTGER3;PTGER3;PTGI	1stExon;Body;1stExon;Body;1stEx	chr1:71512224-71513804	Island
hyper982	N_insufficient_1	9.8E-05	0.35	0.07	0.28	1	71513114	cg17218713	PTGER3;PTGER3;PTGER3;PTGI	1stExon;Body;1stExon;Body;1stEx	chr1:71512224-71513804	Island
hyper983	N_insufficient_1	9.8E-06	0.26	0.10	0.17	1	91316722	cg00371239			chr1:91316249-91317390	Island
hyper984	N_insufficient_1	2.5E-09	0.08	0.01	0.07	1	91316825	cg09257488			chr1:91316249-91317390	Island
hyper985	N_insufficient_1	1.9E-10	0.26	0.04	0.22	1	107682798	cg09822538	NTNG1;NTNG1;NTNG1;NTNG1;N	1stExon;1stExon;5'UTR;1stExon;5	chr1:107682889-107684463	N_Shore
hyper986	N_insufficient_1	3.5E-08	0.18	0.08	0.09	1	107682818	cg20170831	NTNG1;NTNG1;NTNG1;NTNG1;N	1stExon;1stExon;5'UTR;1stExon;5	chr1:107682889-107684463	N_Shore
hyper987	N_insufficient_1	2.4E-02	0.52	0.29	0.23	1	109102212	cg00157962	FAM102B	TSS1500	chr1:109102538-109103212	N_Shore
hyper988	N_insufficient_1	1.4E-02	0.26	0.05	0.22	1	156646746	cg20285128	NES	1stExon	chr1:156646292-156647260	Island
hyper989	N_insufficient_1	6.4E-04	0.23	0.08	0.15	1	164290198	cg23619357			chr1:164290515-164290767	N_Shore
hyper990	N_insufficient_1	1.9E-04	0.20	0.06	0.14	1	164290241	cg24043916			chr1:164290515-164290767	N_Shore
hyper991	N_insufficient_1	3.6E-05	0.26	0.07	0.19	1	167424194	cg22788606	CD247;CD247	Body;Body	chr1:167424088-167425216	Island
hyper992	N_insufficient_1	3.9E-10	0.34	0.10	0.24	1	167424562	cg00628228	CD247;CD247	Body;Body	chr1:167424088-167425216	Island
hyper993	N_insufficient_1	1.9E-10	0.27	0.07	0.21	1	167424664	cg04062187	CD247;CD247	Body;Body	chr1:167424088-167425216	Island
hyper994	N_insufficient_1	9.0E-06	0.29	0.05	0.24	1	167425134	cg18786479	CD247;CD247	Body;Body	chr1:167424088-167425216	Island
hyper995	N_insufficient_1	5.6E-04	0.32	0.10	0.22	1	167425186	cg16127719	CD247;CD247	Body;Body	chr1:167424088-167425216	Island
hyper996	N_insufficient_1	5.9E-06	0.24	0.04	0.20	1	214159131	cg06623935			chr1:214160798-214161034	N_Shore
hyper997	N_insufficient_1	8.4E-02	0.45	0.08	0.36	1	248100614	cg08944170	OR2L13;OR2L13	1stExon;5'UTR	chr1:248100325-248100726	Island
hyper998	N_insufficient_1	9.7E-11	0.22	0.05	0.17	2	21022431	cg25608973	C2orf43	5'UTR	chr2:21022564-21022934	N_Shore
hyper999	N_insufficient_1	6.7E-09	0.26	0.09	0.17	2	21022565	cg09109450	C2orf43	5'UTR	chr2:21022564-21022934	Island
hyper1000	N_insufficient_1	2.9E-10	0.33	0.04	0.29	2	21022587	cg20248458	C2orf43	5'UTR	chr2:21022564-21022934	Island
hyper1001	N_insufficient_1	4.9E-10	0.22	0.08	0.15	2	21022699	cg01816515	C2orf43	5'UTR	chr2:21022564-21022934	Island
hyper1002	N_insufficient_1	4.0E-06	0.19	0.06	0.13	2	21022844	cg01203385	C2orf43	TSS200	chr2:21022564-21022934	Island
hyper1003	N_insufficient_1	6.5E-06	0.14	0.02	0.12	2	21022877	cg26625629	C2orf43	TSS200	chr2:21022564-21022934	Island
hyper1004	N_insufficient_1	5.3E-06	0.30	0.05	0.25	2	21023042	cg14903097	C2orf43	TSS1500	chr2:21022564-21022934	S_Shore
hyper1005	N_insufficient_1	2.9E-04	0.11	0.02	0.09	2	27988858	cg07920525				
hyper1006	N_insufficient_1	7.3E-02	0.30	0.09	0.21	2	88752836	cg13491584	FOXI3	TSS1500	chr2:88751335-88752865	Island
hyper1007	N_insufficient_1	7.5E-05	0.08	0.01	0.07	2	97760749	cg12452265	FAHD2B	TSS200	chr2:97760184-97760756	Island
hyper1008	N_insufficient_1	6.6E-02	0.16	0.09	0.06	2	99553055	cg14122696	C2orf55	TSS1500	chr2:99552185-99553831	Island
hyper1009	N_insufficient_1	7.3E-04	0.25	0.09	0.16	2	227700566	cg07620889	RHBDD1;RHBDD1	TSS200;TSS200	chr2:227700313-227701115	Island
hyper1010	N_insufficient_1	2.9E-03	0.13	0.06	0.07	2	227700568	cg05680085	RHBDD1;RHBDD1	TSS200;TSS200	chr2:227700313-227701115	Island
hyper1011	N_insufficient_1	5.4E-02	0.10	0.02	0.08	2	228028776	cg26685735	COL4A3;COL4A4;COL4A3;COL4/	TSS1500;5'UTR;TSS1500;1stExo	chr2:228028679-228029733	Island
hyper1012	N_insufficient_1	5.6E-02	0.10	0.02	0.08	2	228029433	cg13951042	COL4A3;COL4A3;COL4A3;COL4/	1stExon;1stExon;5'UTR;5'UTR;1st	chr2:228028679-228029733	Island
hyper1013	N_insufficient_1	1.6E-02	0.40	0.07	0.34	2	237087548	cg17536603			chr2:237086727-237088463	Island
hyper1014	N_insufficient_1	0.0E+00	0.38	0.07	0.31	3	13922158	cg04969808	WNT7A	TSS1500	chr3:13919939-13922335	Island
hyper1015	N_insufficient_1	2.1E-07	0.16	0.03	0.13	3	19189456	cg01398050	KCNH8	TSS1500	chr3:19189688-19190100	N_Shore
hyper1016	N_insufficient_1	2.0E-06	0.33	0.08	0.25	3	19189709	cg24997944	KCNH8	TSS1500	chr3:19189688-19190100	Island
hyper1017	N_insufficient_1	0.0E+00	0.39	0.03	0.37	3	19189751	cg24668089	KCNH8	TSS1500	chr3:19189688-19190100	Island
hyper1018	N_insufficient_1	1.7E-04	0.31	0.10	0.21	3	19189930	cg18500192	KCNH8	TSS200	chr3:19189688-19190100	Island
hyper1019	N_insufficient_1	7.4E-02	0.12	0.05	0.07	3	24870666	cg16542478			chr3:24870674-24871369	N_Shore
hyper1020	N_insufficient_1	9.0E-02	0.19	0.07	0.12	3	24871017	cg02044223			chr3:24870674-24871369	Island
hyper1021	N_insufficient_1	0.0E+00	0.29	0.04	0.25	3	44903188	cg25189074	TMEM42;MIR564	TSS1500;TSS200	chr3:44902959-44903623	Island
hyper1022	N_insufficient_1	0.0E+00	0.22	0.03	0.20	3	44903330	cg07542475	MIR564;TMEM42	TSS200;TSS200	chr3:44902959-44903623	Island
hyper1023	N_insufficient_1	1.9E-10	0.34	0.02	0.33	3	44903463	cg17410922	MIR564;TMEM42	Body;1stExon	chr3:44902959-44903623	Island
hyper1024	N_insufficient_1	1.9E-10	0.34	0.02	0.32	3	44903516	cg26889990	TMEM42	1stExon	chr3:44902959-44903623	Island
hyper1025	N_insufficient_1	2.3E-08	0.36	0.12	0.24	3	44903551	cg24100506	TMEM42	1stExon	chr3:44902959-44903623	Island
hyper1026	N_insufficient_1	1.5E-06	0.31	0.07	0.24	3	44903730	cg22887911	TMEM42	Body	chr3:44902959-44903623	S_Shore
hyper1027	N_insufficient_1	4.2E-06	0.22	0.10	0.12	3	44904059	cg04682845	TMEM42	Body	chr3:44902959-44903623	S_Shore

hyper1028	N_insufficient_1	4.0E-03	0.47	0.13	0.34	3	45883628	cg05485520	LZTFL1	TSS200	chr3:45883221-45883860	Island
hyper1029	N_insufficient_1	5.3E-04	0.55	0.06	0.49	3	45883734	cg05648986	LZTFL1	TSS200	chr3:45883221-45883860	Island
hyper1030	N_insufficient_1	2.1E-04	0.17	0.07	0.11	3	67704629	cg07703372	SUCLG2	Body	chr3:67704628-67705051	Island
hyper1031	N_insufficient_1	5.8E-10	0.38	0.03	0.35	3	67704890	cg11115211	SUCLG2	Body	chr3:67704628-67705051	Island
hyper1032	N_insufficient_1	0.0E+00	0.35	0.03	0.32	3	67704970	cg05555772	SUCLG2	1stExon	chr3:67704628-67705051	Island
hyper1033	N_insufficient_1	1.2E-08	0.19	0.02	0.16	3	67705044	cg11161853	SUCLG2	TSS200	chr3:67704628-67705051	Island
hyper1034	N_insufficient_1	1.9E-10	0.21	0.03	0.19	3	67705048	cg24673768	SUCLG2	TSS200	chr3:67704628-67705051	Island
hyper1035	N_insufficient_1	2.1E-09	0.21	0.05	0.15	3	67705222	cg19433066	SUCLG2	TSS200	chr3:67704628-67705051	S_Shore
hyper1036	N_insufficient_1	0.0E+00	0.21	0.09	0.12	3	67705228	cg00837649	SUCLG2	TSS200	chr3:67704628-67705051	S_Shore
hyper1037	N_insufficient_1	3.3E-02	0.17	0.10	0.08	3	67705285	cg11197418	SUCLG2	TSS1500	chr3:67704628-67705051	S_Shore
hyper1038	N_insufficient_1	7.1E-04	0.16	0.08	0.09	3	125932279	cg06827562			chr3:125931735-125932535	Island
hyper1039	N_insufficient_1	2.5E-04	0.12	0.02	0.10	3	136538934	cg18740893	TMEM22;TMEM22;TMEM22	5'UTR;5'UTR;5'UTR	chr3:136537559-136539204	Island
hyper1040	N_insufficient_1	7.3E-02	0.15	0.08	0.07	3	138665006	cg10641721	FOXL2;C3orf72	1stExon;TSS1500	chr3:138663718-138666346	Island
hyper1041	N_insufficient_1	2.9E-08	0.33	0.07	0.26	3	138665654	cg05386493	FOXL2;C3orf72;FOXL2	1stExon;TSS1500;5'UTR	chr3:138663718-138666346	Island
hyper1042	N_insufficient_1	8.8E-04	0.28	0.09	0.20	3	138665930	cg13894046	FOXL2;C3orf72;FOXL2	1stExon;TSS200;5'UTR	chr3:138663718-138666346	Island
hyper1043	N_insufficient_1	7.3E-03	0.37	0.12	0.25	3	138665957	cg19592439	FOXL2;C3orf72;FOXL2	1stExon;TSS200;5'UTR	chr3:138663718-138666346	Island
hyper1044	N_insufficient_1	5.5E-08	0.31	0.02	0.29	3	138665984	cg25670330	FOXL2;C3orf72	TSS200;TSS200	chr3:138663718-138666346	Island
hyper1045	N_insufficient_1	0.0E+00	0.28	0.02	0.26	3	138666040	cg15720669	FOXL2;C3orf72	TSS200;TSS200	chr3:138663718-138666346	Island
hyper1046	N_insufficient_1	5.2E-07	0.18	0.03	0.15	3	138666048	cg04111064	FOXL2;C3orf72	TSS200;TSS200	chr3:138663718-138666346	Island
hyper1047	N_insufficient_1	0.0E+00	0.23	0.02	0.21	3	138666050	cg17802942	FOXL2;C3orf72	TSS200;TSS200	chr3:138663718-138666346	Island
hyper1048	N_insufficient_1	3.9E-10	0.36	0.08	0.28	3	138666054	cg17503456	FOXL2;C3orf72	TSS200;TSS200	chr3:138663718-138666346	Island
hyper1049	N_insufficient_1	0.0E+00	0.35	0.02	0.32	3	138666118	cg06186698	C3orf72;FOXL2;C3orf72	5'UTR;TSS200;1stExon	chr3:138663718-138666346	Island
hyper1050	N_insufficient_1	0.0E+00	0.35	0.02	0.33	3	138666144	cg01379240	C3orf72;FOXL2;C3orf72	5'UTR;TSS200;1stExon	chr3:138663718-138666346	Island
hyper1051	N_insufficient_1	5.3E-07	0.19	0.05	0.14	3	138666318	cg10838925	FOXL2;C3orf72	TSS1500;Body	chr3:138663718-138666346	Island
hyper1052	N_insufficient_1	4.8E-06	0.19	0.01	0.18	3	142682288	cg15852572	PAQR9	TSS200	chr3:142681137-142683268	Island
hyper1053	N_insufficient_1	4.6E-04	0.20	0.04	0.16	3	142682291	cg27406373	PAQR9	TSS200	chr3:142681137-142683268	Island
hyper1054	N_insufficient_1	3.9E-10	0.30	0.04	0.26	3	142682652	cg04912999	PAQR9	TSS1500	chr3:142681137-142683268	Island
hyper1055	N_insufficient_1	4.6E-08	0.26	0.06	0.20	3	142682682	cg17627617	PAQR9	TSS1500	chr3:142681137-142683268	Island
hyper1056	N_insufficient_1	2.0E-03	0.27	0.13	0.14	3	142839837	cg23749856	CHST2	Body	chr3:142837885-142840838	Island
hyper1057	N_insufficient_1	0.0E+00	0.41	0.03	0.38	3	182896670	cg17323383	MCF2L2	3'UTR	chr3:182896920-182897381	N_Shore
hyper1058	N_insufficient_1	0.0E+00	0.45	0.07	0.38	3	182896870	cg02929310	MCF2L2	3'UTR	chr3:182896920-182897381	N_Shore
hyper1059	N_insufficient_1	0.0E+00	0.35	0.08	0.27	3	182896995	cg21857959	MCF2L2	3'UTR	chr3:182896920-182897381	Island
hyper1060	N_insufficient_1	0.0E+00	0.43	0.01	0.42	3	182897196	cg20724810	MCF2L2	Body	chr3:182896920-182897381	Island
hyper1061	N_insufficient_1	0.0E+00	0.46	0.03	0.43	3	182897283	cg00794178	MCF2L2	Body	chr3:182896920-182897381	Island
hyper1062	N_insufficient_1	0.0E+00	0.46	0.05	0.41	3	182970833	cg17701886	MCF2L2;B3GNT5	Body;TSS200	chr3:182971429-182972635	N_Shore
hyper1063	N_insufficient_1	0.0E+00	0.37	0.06	0.31	3	182970846	cg01720186	MCF2L2;B3GNT5	Body;TSS200	chr3:182971429-182972635	N_Shore
hyper1064	N_insufficient_1	0.0E+00	0.40	0.05	0.35	3	182970850	cg15510249	MCF2L2;B3GNT5	Body;TSS200	chr3:182971429-182972635	N_Shore
hyper1065	N_insufficient_1	6.2E-09	0.46	0.08	0.38	3	182970903	cg19998675	MCF2L2;B3GNT5	Body;TSS200	chr3:182971429-182972635	N_Shore
hyper1066	N_insufficient_1	0.0E+00	0.35	0.01	0.34	3	182970927	cg20585088	MCF2L2;B3GNT5	Body;TSS200	chr3:182971429-182972635	N_Shore
hyper1067	N_insufficient_1	0.0E+00	0.42	0.02	0.40	3	182971009	cg12930573	MCF2L2;B3GNT5	Body;TSS200	chr3:182971429-182972635	N_Shore
hyper1068	N_insufficient_1	0.0E+00	0.51	0.01	0.50	3	182971025	cg16938490	MCF2L2;B3GNT5	Body;TSS200	chr3:182971429-182972635	N_Shore
hyper1069	N_insufficient_1	0.0E+00	0.44	0.05	0.40	3	182971069	cg02238826	MCF2L2;B3GNT5;B3GNT5	Body;1stExon;5'UTR	chr3:182971429-182972635	N_Shore
hyper1070	N_insufficient_1	0.0E+00	0.50	0.08	0.41	3	182971430	cg27034902	MCF2L2;B3GNT5	Body;5'UTR	chr3:182971429-182972635	Island
hyper1071	N_insufficient_1	0.0E+00	0.44	0.07	0.37	3	182971442	cg05658593	MCF2L2;B3GNT5	Body;5'UTR	chr3:182971429-182972635	Island
hyper1072	N_insufficient_1	0.0E+00	0.46	0.07	0.39	3	182971483	cg16579954	MCF2L2;B3GNT5	Body;5'UTR	chr3:182971429-182972635	Island
hyper1073	N_insufficient_1	0.0E+00	0.40	0.02	0.38	3	182972048	cg02958321	MCF2L2;B3GNT5	Body;5'UTR	chr3:182971429-182972635	Island
hyper1074	N_insufficient_1	0.0E+00	0.56	0.01	0.55	3	182972326	cg03657573	MCF2L2;B3GNT5	Body;5'UTR	chr3:182971429-182972635	Island
hyper1075	N_insufficient_1	3.6E-03	0.37	0.14	0.24	3	182972742	cg23655039	MCF2L2;B3GNT5	Body;5'UTR	chr3:182971429-182972635	S_Shore
hyper1076	N_insufficient_1	4.7E-04	0.22	0.10	0.12	3	186544945	cg09516202			chr3:186544916-186545471	Island
hyper1077	N_insufficient_1	2.7E-02	0.13	0.05	0.09	3	194406450	cg23670932	FAM43A	TSS200	chr3:194406440-194409045	Island
hyper1078	N_insufficient_1	9.1E-02	0.07	0.01	0.06	3	194406572	cg04689082	FAM43A	TSS200	chr3:194406440-194409045	Island
hyper1079	N_insufficient_1	3.2E-08	0.21	0.08	0.13	3	194407750	cg17064907	FAM43A	1stExon	chr3:194406440-194409045	Island
hyper1080	N_insufficient_1	8.8E-05	0.42	0.16	0.25	4	10458454	cg17524637	ZNF518B	5'UTR	chr4:10458129-10459353	Island
hyper1081	N_insufficient_1	0.0E+00	0.40	0.02	0.38	4	10458531	cg18795809	ZNF518B	5'UTR	chr4:10458129-10459353	Island
hyper1082	N_insufficient_1	0.0E+00	0.19	0.03	0.16	4	10458667	cg13542964	ZNF518B	5'UTR	chr4:10458129-10459353	Island
hyper1083	N_insufficient_1	1.6E-07	0.40	0.12	0.28	4	10458918	cg09534159	ZNF518B	5'UTR	chr4:10458129-10459353	Island
hyper1084	N_insufficient_1	1.9E-04	0.37	0.16	0.21	4	10459141	cg21099470	ZNF518B	TSS200	chr4:10458129-10459353	Island
hyper1085	N_insufficient_1	0.0E+00	0.26	0.07	0.18	4	10459163	cg12016437	ZNF518B	TSS200	chr4:10458129-10459353	Island
hyper1086	N_insufficient_1	0.0E+00	0.25	0.05	0.20	4	10459183	cg04673825	ZNF518B	TSS200	chr4:10458129-10459353	Island
hyper1087	N_insufficient_1	7.3E-05	0.34	0.02	0.32	4	10459219	cg16295725	ZNF518B	TSS200	chr4:10458129-10459353	Island
hyper1088	N_insufficient_1	1.7E-02	0.13	0.06	0.07	4	15402686	cg03290977	C1QTNF7;C1QTNF7	Body;5'UTR		
hyper1089	N_insufficient_1	5.6E-04	0.20	0.05	0.15	4	37245514	cg09295573	KIAA1239	TSS1500	chr4:37245250-37246979	Island
hyper1090	N_insufficient_1	5.0E-03	0.19	0.04	0.15	4	38869650	cg25943096	MIR574;FAM114A1	TSS200;5'UTR	chr4:38869401-38869780	Island
hyper1091	N_insufficient_1	5.6E-03	0.11	0.03	0.08	4	55094499	cg13002939	PDGFRA	TSS1500	chr4:55094321-55094593	Island
hyper1092	N_insufficient_1	2.3E-02	0.34	0.08	0.26	4	85419557	cg18589016	NKX6-1	TSS200	chr4:85417659-85420799	Island
hyper1093	N_insufficient_1	1.4E-02	0.17	0.02	0.16	4	122873018	cg12087304	TRPC3	TSS200	chr4:122871412-122873182	Island
hyper1094	N_insufficient_1	2.6E-03	0.25	0.15	0.11	4	123748753	cg12632152	FGF2	Body	chr4:123747740-123748980	Island
hyper1095	N_insufficient_1	3.1E-03	0.12	0.03	0.09	4	176923544	cg22478591	GPM6A;GPM6A;GPM6A;GPM6A	5'UTR;5'UTR;1stExon;1stExon	chr4:176923075-176923630	Island
hyper1096	N_insufficient_1	1.8E-03	0.20	0.09	0.11	5	19988706	cg19693760	CDH18;CDH18	TSS1500;TSS1500		
hyper1097	N_insufficient_1	0.0E+00	0.28	0.09	0.20	5	76011127	cg04528371	F2R	TSS1500	chr5:76011120-76012292	Island
hyper1098	N_insufficient_1	3.9E-10	0.18	0.03	0.15	5	76011138	cg24678700	F2R	TSS1500	chr5:76011120-76012292	Island
hyper1099	N_insufficient_1	9.7E-11	0.29	0.04	0.25	5	76011304	cg14718568	F2R	TSS1500	chr5:76011120-76012292	Island
hyper1100	N_insufficient_1	1.8E-05	0.14	0.06	0.08	5	76011428	cg13436055	F2R	TSS1500	chr5:76011120-76012292	Island
hyper1101	N_insufficient_1	3.9E-04	0.23	0.05	0.18	5	88178572	cg05948408	MEF2C;MEF2C	5'UTR;5'UTR	chr5:88179866-88180258	N_Shore
hyper1102	N_insufficient_1	7.0E-08	0.32	0.04	0.28	5	98104747	cg02075296	RGMB	TSS1500	chr5:98104578-98106270	Island
hyper1103	N_insufficient_1	1.8E-02	0.12	0.07	0.05	5	110409196	cg08354104	TSLP;TSLP;TSLP	1stExon;5'UTR;Body	chr5:110408704-110409039	S_Shore
hyper1104	N_insufficient_1	6.9E-03	0.28	0.11	0.17	5	113697861	cg07131533	KCNN2	TSS200	chr5:113696516-113699195	Island
hyper1105	N_insufficient_1	4.5E-02	0.16	0.09	0.07	5	114598137	cg02001558	PGGT1B	Body	chr5:114598301-114598657	N_Shore
hyper1106	N_insufficient_1	5.1E-04	0.15	0.08	0.08	5	114598306	cg07242620	PGGT1B	Body	chr5:114598301-114598657	Island
hyper1107	N_insufficient_1	0.0E+00	0.30	0.07	0.23	5	114598433	cg03966541	PGGT1B	1stExon	chr5:114598301-114598657	Island
hyper1108	N_insufficient_1	3.0E-04	0.11	0.05	0.06	5	114598574	cg26173203	PGGT1B	TSS200	chr5:114598301-114598657	Island
hyper1109	N_insufficient_1	1.8E-05	0.12	0.03	0.08	5	114598579	cg26201826	PGGT1B	TSS200	chr5:114598301-114598657	Island
hyper1110	N_insufficient_1	0.0E+00	0.34	0.06	0.28	5	114598649	cg06427561	PGGT1B	TSS200	chr5:114598301-114598657	Island
hyper1111	N_insufficient_1	0.0E+00	0.34	0.06	0.27	5	114598653	cg23027345	PGGT1B	TSS200	chr5:114598301-114598657	Island
hyper1112	N_insufficient_1	9.7E-11	0.30	0.06	0.24	5	114598656	cg15419211	PGGT1B	TSS200	chr5:114598301-114598657	Island
hyper1113	N_insufficient_1	1.3E-03	0.28	0.10	0.18	5	114598768	cg06017028	PGGT1B	TSS200	chr5:114598301-114598657	S_Shore
hyper1114	N_insufficient_1	1.3E-04	0.16	0.08	0.08	5	127420075	cg10423910	SLC12A2;FLJ33630	1stExon;TSS1500	chr5:127418594-127420512	Island
hyper1115	N_insufficient_1	5.4E-02	0.12	0.06	0.06	5	158737729	cg15152115				
hyper1116	N_insufficient_1	4.9E-04	0.19	0.08	0.11	6	7728986	cg10158886	BMP6	Body	chr6:7728885-7729108	Island
hyper1117	N_insufficient_1	5.4E-03	0.15	0.06	0.09	6	30068656	cg13085414			chr6:30068492-30068794	Island
hyper1118	N_insufficient_1	1.0E-04	0.17	0.02	0.16	6	30068667	cg25793630			chr6:30068492-30068794	Island
hyper1119	N_insufficient_1	2.3E-03	0.14	0.07	0.06	6	30068793	cg06053738			chr6:30068492-30068794	Island
hyper1120	N_insufficient_1	9.5E-03	0.15	0.08	0.07	6	30068851	cg13009440			chr6:30069750-30070160	N_Shore
hyper1121	N_insufficient_1	2.9E-08	0.09	0.01	0.08	6	33589172	cg27193704	ITPR3;ITPR3	5'UTR;1stExon	chr6:33588617-33589909	Island
hyper1122	N_insufficient_1	6.2E-03	0.08	0.02	0.07	6	71666682	cg16556906	B3GAT2;B3GAT2	1stExon;5'UTR	chr6:71665360-71667132	Island
hyper1123	N_insufficient_1	3.7E-04	0.25	0.03	0.22	6	91005162	cg12037305	BACH2;BACH2	5'UTR;5'UTR	chr6:91004795-91006944	Island
hyper1124	N_insufficient_1	1.1E-08	0.22	0.03	0.19	6	105584551	cg14159026	BVES;BVES	TSS1500;TSS200	chr6:105584148-105585621	Island
hyper1125	N_insufficient_1	2.9E-04	0.50	0.07	0.43	6	108439146	cg09211399			chr6:108437998-108441208	Island
hyper1126	N_insufficient_1	5.1E-02	0.52	0.10	0.43	6	108439520	cg22727304			chr6:108437998-108441208	Island
hyper1127	N_insufficient_1	3.3E-03	0.16	0.03	0.14	6	150262763	cg09567080	ULBP2	TSS1500	chr6:150262703-150263677	Island
hyper1128	N_insufficient_1	1.1E-04	0.29	0.06	0.22	6	150263194	cg17861551	ULBP2;ULBP2	5'UTR;1stExon	chr6:150262703-150263677	Island
hyper1129	N_insufficient_1	1.5E-08	0.42	0.10	0.32	6	150263354	cg12808992	ULBP2	Body	chr6:150262703-150263677	Island
hyper1130	N_insufficient_1	1.0E-03	0.25	0.12	0.13	7	2727683	cg12529027	AMZ1	5'UTR	chr7:2727682-2728392	Island
hyper1131	N_insufficient_1	3.0E-04	0.28	0.03	0.25	7	2727765	cg13607835	AMZ1	5'UTR	chr7:2727682-2728392	Island
hyper1132	N_insufficient_1	1.9E-02	0.14	0.07	0.06	7	38670597	cg04276723	AMPH;AMPH	Body;Body	chr7:38670439-38671008	Island
hyper1133	N_insufficient_1	1.3E-06	0.12	0.02	0.09	7	38670804	cg19875547	AMPH;AMPH	Body;Body	chr7:38670439-38671008	Island
hyper1134	N_insufficient_1	1.7E-04	0.14	0.04	0.10	7	38670840	cg07821629	AMPH;AMPH	Body;Body	chr7:38670439-38671008	Island
hyper1135	N_insufficient_1	9.7E-10	0.13	0.01	0.12	7	100230781	cg04739306	TFR2	Body	chr7:100230649-100231320	Island
hyper1136	N_insufficient_1	2.0E-05	0.32	0.06	0.26	7	100435192	cg08302003			chr7:100434982-100435193	Island
hyper1137	N_insufficient_1	6.8E-03	0.22	0.05	0.17	7	133812072	cg07355590	LRGUK	TSS200	chr7:133812005-133812233	Island
hyper1138	N_insufficient_1	5.1E-02	0.11	0.04	0.06	7	136553778	cg10454514	CHRM2;CHRM2;CHRM2;CHRM2	5'UTR;TSS200;TSS1500;5'UTR;T	chr7:136553854-136556194	N_Shore
hyper1139	N_insufficient_1	7.2E-04	0.31	0.09	0.22	7	137531801	cg18324707	DGKI	TSS200	chr7:137531125-137532382	Island
hyper1140	N_insufficient_1	1.4E-03	0.32	0.10	0.22	7	152622368	cg02432280			chr7:152622343-152623305	Island
hyper1141	N_insufficient_1	2.2E-04	0.36	0.05	0.31	7	152622455	cg00469207			chr7:152622343-152623305	Island
hyper1142	N_insufficient_1	1.0E-04	0.32	0.10	0.23	7	152622787	cg09605164			chr7:152622343-152623305	Island
hyper1143	N_insufficient_1	1.2E-06	0.14	0.04	0.10	8	1771973	cg10964367	ARHGEF10	TSS200	chr8:1771361-1772760	Island
hyper1144	N_insufficient_1	2.0E-05	0.14	0.05	0.10	8	1772080	cg06076323	ARHGEF10	TSS200	chr8:1771361-1772760	Island
hyper1145	N_insufficient_1	3.1E-06	0.16	0.03	0.13	8	10587769	cg26077100	SOX7	1stExon	chr8:10587304-10591109	Island
hyper1146	N_insufficient_1	0.0E+00	0.20	0.01	0.19	8	10588013	cg08056146	SOX7;SOX7	1stExon;5'UTR	chr8:10587304-10591109	Island
hyper1147	N_insufficient_1	3.7E-09	0.25	0.03	0.21	8	10588040	cg07233097	SOX7	TSS200	chr8:10587304-10591109	Island
hyper1148	N_insufficient_1	2.7E-09	0.28	0.08	0.20	8	10874016	cg04317977	XKR6	Body	chr8:10873324-10874174	Island
hyper1149	N_insufficient_1	2.1E-02	0.11	0.04	0.07	8	10874215	cg14637067	XKR6	Body	chr8:10873324-10874174	S_Shore
hyper1150	N_insufficient_1	1.5E-04	0.22	0.08	0.14	8	22408966	cg03601354	SORBS3	TSS1500	chr8:22408672-22409635	Island
hyper1151	N_insufficient_1	1.4E-05	0.18	0.04	0.14	8	22409000	cg13951572	SORBS3	TSS1500	chr8:22408672-22409635	Island
hyper1152	N_insufficient_1	1.3E-03	0.21	0.11	0.11	8	22409131	cg07459525	SORBS3	TSS200	chr8:22408672-22409635	Island
hyper1153	N_insufficient_1	5.3E-02	0.11	0.06	0.05	8	67874858	cg07922007			chr8:67873388-67875600	Island
hyper1154	N_insufficient_1	1.7E-04	0.25	0.04	0.21	8	72459953	cg07161721			chr8:72459564-72460523	Island
hyper1155	N_insufficient_1	3.3E-06	0.23	0.06	0.17	8	97507561	cg10292139	SDC2	Body	chr8:97505747-97507607	Island
hyper1156	N_insufficient_1	7.6E-05	0.17	0.09	0.07	8	97507592	cg16673702	SDC2	Body	chr8:97505747-97507607	Island

hyper1157	N_insufficient_1	1.5E-09	0.09	0.02	0.07	8	97507595	cg07146119	SDC2	Body	chr8:97505747-97507607	Island
hyper1158	N_insufficient_1	8.7E-04	0.21	0.09	0.12	8	97507666	cg20594401	SDC2	Body	chr8:97505747-97507607	S_Shore
hyper1159	N_insufficient_1	1.4E-09	0.23	0.03	0.19	8	143696133	cg04321580	ARC	TSS1500	chr8:143694430-143696166	Island
hyper1160	N_insufficient_1	3.8E-02	0.41	0.05	0.37	9	14313526	cg13518604	NFIB;NFIB	5'UTR;1stExon	chr9:14312952-14315351	Island
hyper1161	N_insufficient_1	4.4E-03	0.45	0.09	0.36	9	14313762	cg09965258	NFIB;NFIB	5'UTR;1stExon	chr9:14312952-14315351	Island
hyper1162	N_insufficient_1	2.9E-10	0.28	0.04	0.24	9	14314158	cg14455366	NFIB	TSS1500	chr9:14312952-14315351	Island
hyper1163	N_insufficient_1	5.8E-08	0.19	0.04	0.15	9	21974704	cg13601799	CDKN2A;CDKN2A;CDKN2A	1stExon;Body;1stExon	chr9:21974578-21975306	Island
hyper1164	N_insufficient_1	2.0E-05	0.19	0.04	0.15	9	96708224	cg13867237			chr9:96708116-96708487	Island
hyper1165	N_insufficient_1	1.7E-09	0.31	0.03	0.28	9	125109046	cg13655082			chr9:125109007-125109644	Island
hyper1166	N_insufficient_1	4.9E-10	0.28	0.08	0.19	9	125109120	cg12675848			chr9:125109007-125109644	Island
hyper1167	N_insufficient_1	0.0E+00	0.27	0.02	0.24	9	125109458	cg13808240			chr9:125109007-125109644	Island
hyper1168	N_insufficient_1	5.0E-07	0.20	0.07	0.14	9	125109542	cg03246694			chr9:125109007-125109644	Island
hyper1169	N_insufficient_1	1.6E-05	0.42	0.11	0.31	9	125133121	cg10493166	PTGS1;PTGS1	TSS200;TSS200		
hyper1170	N_insufficient_1	4.2E-03	0.47	0.05	0.42	9	125133318	cg12927153	PTGS1;PTGS1;PTGS1;PTGS1	5'UTR;5'UTR;1stExon;1stExon		
hyper1171	N_insufficient_1	2.0E-04	0.21	0.06	0.15	9	137533141	cg01753595	COL5A1	TSS1500	chr9:137533359-137534397	N_Shore
hyper1172	N_insufficient_1	1.4E-04	0.17	0.04	0.13	10	7451314	cg09060610	SFMBT2	5'UTR	chr10:7449376-7455339	Island
hyper1173	N_insufficient_1	8.3E-09	0.31	0.06	0.25	10	7451822	cg25291138	SFMBT2	5'UTR	chr10:7449376-7455339	Island
hyper1174	N_insufficient_1	6.4E-02	0.26	0.09	0.16	10	28287388	cg17862332	ARMC4	5'UTR	chr10:28287447-28288057	N_Shore
hyper1175	N_insufficient_1	3.1E-05	0.20	0.08	0.12	10	28287707	cg08555772	ARMC4	5'UTR	chr10:28287447-28288057	Island
hyper1176	N_insufficient_1	9.7E-11	0.31	0.04	0.28	10	28287879	cg00366818	ARMC4	5'UTR	chr10:28287447-28288057	Island
hyper1177	N_insufficient_1	3.2E-05	0.06	0.01	0.05	10	31609882	cg12758636	ZEB1;ZEB1;ZEB1;ZEB1;ZEB1	TSS200;Body;Body;Body;Body	chr10:31607227-31610094	Island
hyper1178	N_insufficient_1	9.8E-05	0.18	0.05	0.14	10	31609891	cg04430381	ZEB1;ZEB1;ZEB1;ZEB1;ZEB1	TSS200;Body;Body;Body;Body	chr10:31607227-31610094	Island
hyper1179	N_insufficient_1	9.4E-08	0.22	0.06	0.16	10	31609896	cg18465286	ZEB1;ZEB1;ZEB1;ZEB1;ZEB1	TSS200;Body;Body;Body;Body	chr10:31607227-31610094	Island
hyper1180	N_insufficient_1	6.4E-06	0.13	0.02	0.11	10	31609919	cg04667277	ZEB1;ZEB1;ZEB1;ZEB1;ZEB1	TSS200;Body;Body;Body;Body	chr10:31607227-31610094	Island
hyper1181	N_insufficient_1	6.6E-03	0.29	0.05	0.24	10	50887616	cg14526047	C10orf53;C10orf53	TSS200;TSS200	chr10:50887565-50887862	Island
hyper1182	N_insufficient_1	0.0E+00	0.29	0.04	0.25	10	88126089	cg03356747	GRID1	1stExon	chr10:88122924-88127364	Island
hyper1183	N_insufficient_1	4.6E-06	0.21	0.07	0.14	10	88126104	cg03370738	GRID1	1stExon	chr10:88122924-88127364	Island
hyper1184	N_insufficient_1	0.0E+00	0.19	0.01	0.18	10	88126299	cg03052869	GRID1	TSS200	chr10:88122924-88127364	Island
hyper1185	N_insufficient_1	0.0E+00	0.14	0.01	0.13	10	88126306	cg20649951	GRID1	TSS200	chr10:88122924-88127364	Island
hyper1186	N_insufficient_1	2.5E-02	0.11	0.05	0.05	10	90967189	cg10092652	CH25H	TSS200	chr10:90967555-90967900	N_Shore
hyper1187	N_insufficient_1	2.2E-03	0.39	0.14	0.25	10	97802928	cg26268044	CCNJ;CCNJ;CCNJ	TSS1500;TSS1500;TSS1500	chr10:97802871-97804262	Island
hyper1188	N_insufficient_1	6.8E-06	0.30	0.11	0.19	10	97802941	cg04590978	CCNJ;CCNJ;CCNJ	TSS1500;TSS1500;TSS1500	chr10:97802871-97804262	Island
hyper1189	N_insufficient_1	4.1E-03	0.43	0.25	0.18	10	97802959	cg01016800	CCNJ;CCNJ;CCNJ	TSS200;TSS200;TSS200	chr10:97802871-97804262	Island
hyper1190	N_insufficient_1	8.7E-05	0.18	0.07	0.10	10	97802977	cg08461586	CCNJ;CCNJ;CCNJ	TSS200;TSS200;TSS200	chr10:97802871-97804262	Island
hyper1191	N_insufficient_1	1.9E-10	0.26	0.09	0.18	10	97803045	cg00015024	CCNJ;CCNJ;CCNJ	TSS200;TSS200;TSS200	chr10:97802871-97804262	Island
hyper1192	N_insufficient_1	0.0E+00	0.29	0.09	0.20	10	97803048	cg11930665	CCNJ;CCNJ;CCNJ	TSS200;TSS200;TSS200	chr10:97802871-97804262	Island
hyper1193	N_insufficient_1	4.4E-04	0.35	0.20	0.16	10	97803058	cg08379212	CCNJ;CCNJ;CCNJ	TSS200;TSS200;TSS200	chr10:97802871-97804262	Island
hyper1194	N_insufficient_1	0.0E+00	0.36	0.01	0.35	10	97803386	cg05739190	CCNJ;CCNJ;CCNJ;CCNJ;CCNJ;(	1stExon;1stExon;5'UTR;5'UTR;1st	chr10:97802871-97804262	Island
hyper1195	N_insufficient_1	0.0E+00	0.31	0.02	0.30	10	97803525	cg22864416	CCNJ;CCNJ;CCNJ	5'UTR;5'UTR;5'UTR	chr10:97802871-97804262	Island
hyper1196	N_insufficient_1	4.6E-09	0.24	0.08	0.16	10	97804045	cg10317807	CCNJ;CCNJ;CCNJ	5'UTR;5'UTR;5'UTR	chr10:97802871-97804262	Island
hyper1197	N_insufficient_1	4.7E-02	0.14	0.05	0.09	10	102505365	cg04235519	PAX2;PAX2;PAX2;PAX2;PAX2	TSS200;TSS200;TSS200;TSS200	chr10:102503913-102506146	Island
hyper1198	N_insufficient_1	3.3E-02	0.14	0.07	0.07	10	116163160	cg20048434	AFAP1L2;AFAP1L2	Body;Body	chr10:116163391-116164599	N_Shore
hyper1199	N_insufficient_1	4.4E-06	0.22	0.09	0.13	10	116163392	cg20663200	AFAP1L2;AFAP1L2	Body;Body	chr10:116163391-116164599	Island
hyper1200	N_insufficient_1	0.0E+00	0.35	0.05	0.30	10	121652004	cg25418309	SEC23IP	TSS1500	chr10:121652003-121652507	Island
hyper1201	N_insufficient_1	0.0E+00	0.37	0.01	0.36	10	121652203	cg09776513	SEC23IP	TSS200	chr10:121652003-121652507	Island
hyper1202	N_insufficient_1	0.0E+00	0.40	0.01	0.39	10	121652237	cg04622176	SEC23IP;SEC23IP	1stExon;5'UTR	chr10:121652003-121652507	Island
hyper1203	N_insufficient_1	4.4E-09	0.51	0.07	0.45	10	123873748	cg23828763	TACC2;TACC2	Body;Body	chr10:123872119-123873462	S_Shore
hyper1204	N_insufficient_1	5.7E-03	0.23	0.04	0.19	10	124907540	cg17498296	HMX2	TSS200	chr10:124907283-124911035	Island
hyper1205	N_insufficient_1	5.5E-07	0.30	0.02	0.28	10	124907553	cg22603247	HMX2	TSS200	chr10:124907283-124911035	Island
hyper1206	N_insufficient_1	9.8E-05	0.17	0.08	0.08	10	124909415	cg04820634	HMX2	Body	chr10:124907283-124911035	Island
hyper1207	N_insufficient_1	7.6E-03	0.17	0.08	0.09	10	128076260	cg08602190	ADAM12;ADAM12	Body;Body	chr10:128076155-128077482	Island
hyper1208	N_insufficient_1	7.0E-05	0.28	0.08	0.20	10	128077080	cg00922781	ADAM12;ADAM12;ADAM12;ADAI	1stExon;5'UTR;5'UTR;1stExon	chr10:128076155-128077482	Island
hyper1209	N_insufficient_1	1.9E-02	0.38	0.09	0.29	10	133850544	cg16447865			chr10:133849676-133850826	Island
hyper1210	N_insufficient_1	7.5E-02	0.18	0.05	0.13	11	12031097	cg05106294	DKK3;DKK3;DKK3	TSS1500;TSS200;TSS1500	chr11:12029737-12030841	S_Shore
hyper1211	N_insufficient_1	1.8E-02	0.25	0.09	0.16	11	12031099	cg01286655	DKK3;DKK3;DKK3	TSS1500;TSS200;TSS1500	chr11:12029737-12030841	S_Shore
hyper1212	N_insufficient_1	6.7E-04	0.18	0.09	0.09	11	44971593	cg24061542	TP53I11;TP53I11	5'UTR;5'UTR	chr11:44971048-44972685	Island
hyper1213	N_insufficient_1	6.5E-08	0.18	0.07	0.11	11	44971602	cg07916488	TP53I11;TP53I11	5'UTR;5'UTR	chr11:44971048-44972685	Island
hyper1214	N_insufficient_1	1.1E-07	0.13	0.01	0.11	11	44971833	cg24331312	TP53I11;TP53I11	5'UTR;5'UTR	chr11:44971048-44972685	Island
hyper1215	N_insufficient_1	2.6E-05	0.13	0.02	0.11	11	44971894	cg07301180	TP53I11;TP53I11	5'UTR;5'UTR	chr11:44971048-44972685	Island
hyper1216	N_insufficient_1	6.5E-08	0.12	0.02	0.10	11	44972293	cg22623062	TP53I11;TP53I11;TP53I11;TP53I1	5'UTR;1stExon;5'UTR;1stExon	chr11:44971048-44972685	Island
hyper1217	N_insufficient_1	2.5E-03	0.35	0.12	0.23	11	56059643	cg22629065	OR8H1	TSS1500		
hyper1218	N_insufficient_1	1.6E-05	0.22	0.04	0.18	11	59333490	cg14665813				
hyper1219	N_insufficient_1	1.3E-06	0.14	0.06	0.08	11	59333608	cg18442286				
hyper1220	N_insufficient_1	5.8E-05	0.16	0.03	0.12	11	69590113	cg23964057	FGF4;FGF4	1stExon;5'UTR	chr11:69588826-69590546	Island
hyper1221	N_insufficient_1	1.6E-06	0.16	0.03	0.14	11	69590282	cg18947739	FGF4	TSS200	chr11:69588826-69590546	Island
hyper1222	N_insufficient_1	4.1E-03	0.10	0.03	0.07	11	110583036	cg03813168	ARHGAP20	5'UTR	chr11:110581839-110583883	Island
hyper1223	N_insufficient_1	1.8E-02	0.13	0.06	0.07	11	111411756	cg04610224	LAYN	Body	chr11:111410932-111412199	Island
hyper1224	N_insufficient_1	7.2E-06	0.39	0.05	0.34	11	113930430	cg09719477	ZBTB16;ZBTB16;ZBTB16	1stExon;TSS1500;5'UTR	chr11:113929633-113932190	Island
hyper1225	N_insufficient_1	1.1E-04	0.49	0.19	0.30	11	113931305	cg09890653	ZBTB16;ZBTB16;ZBTB16	5'UTR;1stExon;5'UTR	chr11:113929633-113932190	Island
hyper1226	N_insufficient_1	2.1E-09	0.32	0.01	0.31	11	120894801	cg06747745	TBCEL;TBCEL	TSS200;TSS200	chr11:120894689-120895082	Island
hyper1227	N_insufficient_1	1.3E-03	0.27	0.02	0.24	11	128563634	cg08590601	FLI1;FLI1	TSS200;5'UTR	chr11:128562671-128565011	Island
hyper1228	N_insufficient_1	1.2E-03	0.12	0.04	0.08	12	1639527	cg05998639			chr12:1639033-1640750	Island
hyper1229	N_insufficient_1	3.0E-05	0.18	0.06	0.12	12	1639534	cg05641033			chr12:1639033-1640750	Island
hyper1230	N_insufficient_1	8.6E-04	0.15	0.08	0.07	12	8332881	cg18554736	FAM66C	Body	chr12:8332788-8333193	Island
hyper1231	N_insufficient_1	2.1E-02	0.38	0.11	0.27	12	9217763	cg10605084	LOC144571	TSS200	chr12:9217328-9217715	S_Shore
hyper1232	N_insufficient_1	5.0E-02	0.14	0.02	0.12	12	54144855	cg05385164			chr12:54144562-54145470	Island
hyper1233	N_insufficient_1	3.8E-04	0.31	0.11	0.20	12	54144921	cg06858186			chr12:54144562-54145470	Island
hyper1234	N_insufficient_1	7.0E-08	0.30	0.07	0.24	12	64062564	cg04455164	DPY19L2	TSS1500	chr12:64061836-64062774	Island
hyper1235	N_insufficient_1	7.6E-03	0.13	0.04	0.09	12	66217818	cg16409497	HMGA2;RPSAP52;HMGA2	TSS1500;Body;TSS1500	chr12:66217627-66220181	Island
hyper1236	N_insufficient_1	7.0E-03	0.33	0.12	0.22	12	92535822	cg08832851	BTG1	3'UTR	chr12:92538710-92540333	N_Shelf
hyper1237	N_insufficient_1	3.7E-02	0.30	0.06	0.24	12	113914511	cg05412058			chr12:113914440-113914657	Island
hyper1238	N_insufficient_1	1.3E-04	0.18	0.08	0.10	13	100631545	cg17933512			chr13:100631752-100635526	N_Shore
hyper1239	N_insufficient_1	1.4E-03	0.24	0.08	0.16	13	100631588	cg25962735			chr13:100631752-100635526	N_Shore
hyper1240	N_insufficient_1	7.4E-02	0.40	0.06	0.35	13	110438288	cg08510264	IRS2	1stExon	chr13:110434466-110440180	Island
hyper1241	N_insufficient_1	7.2E-07	0.31	0.06	0.25	13	110438578	cg02320481	IRS2;IRS2	5'UTR;1stExon	chr13:110434466-110440180	Island
hyper1242	N_insufficient_1	3.0E-03	0.09	0.02	0.08	13	110438906	cg14341579	IRS2;IRS2	5'UTR;1stExon	chr13:110434466-110440180	Island
hyper1243	N_insufficient_1	1.2E-07	0.13	0.02	0.11	13	110439453	cg01939883	IRS2	TSS1500	chr13:110434466-110440180	Island
hyper1244	N_insufficient_1	2.7E-03	0.34	0.10	0.24	14	29235984	cg19714132	FOXG1	TSS1500	chr14:29236835-29237832	N_Shore
hyper1245	N_insufficient_1	6.6E-02	0.16	0.06	0.10	14	31344535	cg10128164	COCH;COCH	Body;Body	chr14:31343355-31344757	Island
hyper1246	N_insufficient_1	5.7E-02	0.12	0.03	0.09	14	33403060	cg03298319			chr14:33402094-33404079	Island
hyper1247	N_insufficient_1	4.3E-04	0.29	0.07	0.22	14	33403900	cg01935149			chr14:33402094-33404079	Island
hyper1248	N_insufficient_1	5.9E-03	0.14	0.05	0.09	14	33404180	cg05082965			chr14:33402094-33404079	S_Shore
hyper1249	N_insufficient_1	4.8E-06	0.25	0.09	0.16	14	36994153	cg01509464			chr14:36993488-36994488	Island
hyper1250	N_insufficient_1	1.7E-03	0.10	0.05	0.05	14	67878973	cg20141916	PLEK2	TSS200	chr14:67878534-67879167	Island
hyper1251	N_insufficient_1	9.1E-02	0.37	0.14	0.23	14	67879166	cg26225496	PLEK2	TSS1500	chr14:67878534-67879167	Island
hyper1252	N_insufficient_1	1.3E-03	0.32	0.10	0.22	14	73704842	cg21912093	PAPLN	5'UTR	chr14:73704054-73705106	Island
hyper1253	N_insufficient_1	6.5E-09	0.23	0.07	0.17	14	74058486	cg13785718	ACOT4;ACOT4	5'UTR;1stExon	chr14:74058400-74059116	Island
hyper1254	N_insufficient_1	1.2E-09	0.23	0.07	0.15	14	74058654	cg10733249	ACOT4;ACOT4	5'UTR;1stExon	chr14:74058400-74059116	Island
hyper1255	N_insufficient_1	1.3E-07	0.34	0.04	0.29	14	74058881	cg18167179	ACOT4	1stExon	chr14:74058400-74059116	Island
hyper1256	N_insufficient_1	9.2E-02	0.16	0.05	0.11	14	74059005	cg24720580	ACOT4	1stExon	chr14:74058400-74059116	Island
hyper1257	N_insufficient_1	5.9E-05	0.19	0.01	0.17	14	95236123	cg10042799	GSC	1stExon	chr14:95234612-95236569	Island
hyper1258	N_insufficient_1	3.7E-03	0.22	0.02	0.20	14	99738493	cg09256131	BCL11B;BCL11B	TSS1500;TSS1500	chr14:99739801-99740920	N_Shore
hyper1259	N_insufficient_1	9.0E-05	0.34	0.06	0.28	15	34786594	cg03062549			chr15:34786492-34788138	Island
hyper1260	N_insufficient_1	5.4E-02	0.25	0.12	0.13	15	48470205	cg03297593	MYEF2	Body	chr15:48470007-48470628	Island
hyper1261	N_insufficient_1	4.3E-07	0.26	0.10	0.16	15	48470256	cg20612002	MYEF2	Body	chr15:48470007-48470628	Island
hyper1262	N_insufficient_1	1.7E-07	0.34	0.11	0.22	15	48470321	cg09704159	MYEF2	1stExon	chr15:48470007-48470628	Island
hyper1263	N_insufficient_1	3.1E-04	0.21	0.04	0.16	15	48470612	cg22700091	MYEF2	TSS200	chr15:48470007-48470628	Island
hyper1264	N_insufficient_1	1.4E-02	0.23	0.08	0.15	15	85113590	cg11777390	UBE2QP1	Body	chr15:85113435-85114361	Island
hyper1265	N_insufficient_1	5.1E-02	0.29	0.05	0.24	15	92936818	cg26739280	ST8SIA2	TSS1500	chr15:92936677-92938521	Island
hyper1266	N_insufficient_1	0.0E+00	0.28	0.01	0.27	15	99646202	cg08892705	SYNM;SYNM	1stExon;1stExon	chr15:99645030-99646444	Island
hyper1267	N_insufficient_1	2.2E-06	0.32	0.10	0.22	15	100890168	cg12240002	FLJ42289;FLJ42289	Body;Body	chr15:100890242-100890494	N_Shore
hyper1268	N_insufficient_1	1.4E-06	0.33	0.06	0.27	16	216100	cg26976732	HBM	Body	chr16:214343-216720	Island
hyper1269	N_insufficient_1	6.8E-03	0.06	0.01	0.04	16	47178067	cg01122672	NETO2	TSS200	chr16:47176787-47178446	Island
hyper1270	N_insufficient_1	2.6E-06	0.19	0.07	0.12	16	55358636	cg01549404	IRX6;IRX6	5'UTR;1stExon	chr16:55357521-55359040	Island
hyper1271	N_insufficient_1	3.2E-03	0.09	0.01	0.07	16	55513480	cg10079740	MMP2	1stExon	chr16:55513220-55513526	Island
hyper1272	N_insufficient_1	6.0E-03	0.15	0.03	0.12	16	57023329	cg03056682	NLRC5	TSS200	chr16:57023190-57023768	Island
hyper1273	N_insufficient_1	4.9E-05	0.12	0.04	0.08	16	57023398	cg00881185	NLRC5	TSS200	chr16:57023190-57023768	Island
hyper1274	N_insufficient_1	5.6E-05	0.11	0.01	0.10	16	57023483	cg26963976	NLRC5;NLRC5	5'UTR;1stExon	chr16:57023190-57023768	Island
hyper1275	N_insufficient_1	8.5E-08	0.08	0.01	0.07	16	57023494	cg02169333	NLRC5;NLRC5	5'UTR;1stExon	chr16:57023190-57023768	Island
hyper1276	N_insufficient_1	8.0E-04	0.18	0.03	0.14	16	62070164	cg27120816	CDH8	TSS200	chr16:62069121-62070634	Island
hyper1277	N_insufficient_1	1.7E-02	0.23	0.02	0.21	16	62070184	cg01718742	CDH8	TSS200	chr16:62069121-62070634	Island
hyper1278	N_insufficient_1	8.5E-07	0.08	0.01	0.07	16	86543519	cg07769121	FOXF1	TSS1500	chr16:86541430-86545228	Island
hyper1279	N_insufficient_1	4.2E-06	0.36	0.03	0.33	16	89160395	cg07065570	ACSF3;ACSF3;ACSF3;ACSF3;AC	Body;1stExon;5'UTR;5'UTR;1stEx	chr16:89160018-89160706	Island
hyper1280	N_insufficient_1	7.3E-04	0.40	0.03	0.37	16	89160400	cg27329533	ACSF3;ACSF3;ACSF3;ACSF3	Body;5'UTR;5'UTR;1stExon	chr16:89160018-89160706	Island
hyper1281	N_insufficient_1	6.6E-03	0.14	0.07	0.08	16	89160798	cg16555341	ACSF3;ACSF3;ACSF3	Body;5'UTR;5'UTR	chr16:89160018-89160706	S_Shore
hyper1282	N_insufficient_1	3.8E-06	0.16	0.02	0.14	17	6938938	cg01107801	SLC16A13	TSS1500	chr17:6939137-6940090	N_Shore
hyper1283	N_insufficient_1	0.0E+00	0.19	0.01	0.17	17	6939138	cg03569506	SLC16A13	TSS1500	chr17:6939137-6940090	Island
hyper1284	N_insufficient_1	0.0E+00	0.20	0.02	0.19	17	6939140	cg21577651	SLC16A13	TSS1500	chr17:6939137-6940090	Island
hyper1285	N_insufficient_1	2.1E-09	0.21	0.03	0.18	17	6939145	cg24535234	SLC16A13	TSS1500	chr17:6939137-6940090	Island

hyper1286	N_insufficient_1	0.0E+00	0.26	0.04	0.22	17	6939282	cg09543792	SLC16A13	TSS200	chr17:6939137-6940090	Island
hyper1287	N_insufficient_1	0.0E+00	0.33	0.05	0.27	17	6939299	cg22884899	SLC16A13	TSS200	chr17:6939137-6940090	Island
hyper1288	N_insufficient_1	5.8E-10	0.27	0.03	0.24	17	6939304	cg25241322	SLC16A13	TSS200	chr17:6939137-6940090	Island
hyper1289	N_insufficient_1	1.3E-04	0.31	0.12	0.19	17	6939695	cg02805871	SLC16A13;SLC16A13	5'UTR;1stExon	chr17:6939137-6940090	Island
hyper1290	N_insufficient_1	1.7E-02	0.19	0.08	0.11	17	8906601	cg07848706			chr17:8906236-8908080	Island
hyper1291	N_insufficient_1	4.1E-06	0.13	0.06	0.06	17	19437003	cg15014549	SLC47A1	TSS200	chr17:19436788-19437692	Island
hyper1292	N_insufficient_1	1.9E-04	0.14	0.06	0.08	17	19437013	cg07829432	SLC47A1	TSS200	chr17:19436788-19437692	Island
hyper1293	N_insufficient_1	0.0E+00	0.18	0.03	0.16	17	19437157	cg13004927	SLC47A1	TSS200	chr17:19436788-19437692	Island
hyper1294	N_insufficient_1	9.2E-05	0.38	0.07	0.31	17	31619231	cg09677243	ACCN1;ACCN1;ACCN1	1stExon;5'UTR;Body	chr17:31618345-31620465	Island
hyper1295	N_insufficient_1	2.0E-02	0.23	0.07	0.15	17	31620378	cg23763736	ACCN1;ACCN1	TSS1500;Body	chr17:31618345-31620465	Island
hyper1296	N_insufficient_1	2.1E-02	0.10	0.06	0.04	17	35014438	cg00371418			chr17:35014272-35014596	Island
hyper1297	N_insufficient_1	6.1E-06	0.12	0.02	0.10	17	39890494	cg01777228	HAP1;HAP1;HAP1	1stExon;1stExon;1stExon	chr17:39889956-39891489	Island
hyper1298	N_insufficient_1	2.0E-03	0.19	0.04	0.16	17	40913055	cg01101510	LOC100190938;RAMP2;LOC1001	Body;TSS200;Body	chr17:40912816-40913553	Island
hyper1299	N_insufficient_1	3.9E-03	0.17	0.05	0.12	17	40913076	cg12627273	LOC100190938;RAMP2;LOC1001	Body;TSS200;Body	chr17:40912816-40913553	Island
hyper1300	N_insufficient_1	5.4E-04	0.08	0.02	0.05	17	42061083	cg27375198	PYY	5'UTR	chr17:42061046-42061643	Island
hyper1301	N_insufficient_1	1.6E-02	0.07	0.02	0.05	17	47307743	cg03259494	PHOSPHO1;PHOSPHO1	5'UTR;5'UTR	chr17:47307740-47308276	Island
hyper1302	N_insufficient_1	2.1E-02	0.36	0.07	0.30	17	54991064	cg20613151	TRIM25	1stExon	chr17:54990832-54991771	Island
hyper1303	N_insufficient_1	2.8E-04	0.07	0.02	0.05	17	54991616	cg19695198	TRIM25	TSS1500	chr17:54990832-54991771	Island
hyper1304	N_insufficient_1	3.9E-02	0.11	0.05	0.07	17	54991697	cg00938453	TRIM25	TSS1500	chr17:54990832-54991771	Island
hyper1305	N_insufficient_1	9.8E-03	0.12	0.03	0.08	17	58227265	cg23010302	CA4	TSS200	chr17:58227241-58227720	Island
hyper1306	N_insufficient_1	4.9E-07	0.11	0.02	0.09	17	58227267	cg05248618	CA4	TSS200	chr17:58227241-58227720	Island
hyper1307	N_insufficient_1	0.0E+00	0.62	0.12	0.50	17	58498466	cg06531671	C17orf64	TSS1500	chr17:58498711-58499332	N_Shore
hyper1308	N_insufficient_1	0.0E+00	0.45	0.06	0.38	17	58498712	cg22191510	C17orf64	TSS1500	chr17:58498711-58499332	Island
hyper1309	N_insufficient_1	0.0E+00	0.61	0.10	0.51	17	58498871	cg10179315	C17orf64	TSS1500	chr17:58498711-58499332	Island
hyper1310	N_insufficient_1	0.0E+00	0.62	0.01	0.61	17	58498977	cg09695735	C17orf64	TSS1500	chr17:58498711-58499332	Island
hyper1311	N_insufficient_1	5.4E-03	0.12	0.03	0.09	18	20716451	cg26610812	CABLES1;CABLES1	1stExon;Body	chr18:20714968-20717071	Island
hyper1312	N_insufficient_1	1.9E-10	0.32	0.05	0.27	18	21851455	cg06997114	OSBPL1A;OSBPL1A	Body;5'UTR	chr18:21851399-21852169	Island
hyper1313	N_insufficient_1	0.0E+00	0.40	0.07	0.34	18	21852118	cg19925062	OSBPL1A;OSBPL1A;OSBPL1A	Body;1stExon;5'UTR	chr18:21851399-21852169	Island
hyper1314	N_insufficient_1	1.9E-08	0.25	0.01	0.24	18	21852325	cg03384992	OSBPL1A;OSBPL1A	Body;TSS200	chr18:21851399-21852169	S_Shore
hyper1315	N_insufficient_1	1.6E-09	0.29	0.04	0.25	18	21852327	cg26398656	OSBPL1A;OSBPL1A	Body;TSS200	chr18:21851399-21852169	S_Shore
hyper1316	N_insufficient_1	7.8E-10	0.37	0.05	0.32	18	21852333	cg06593258	OSBPL1A;OSBPL1A	Body;TSS200	chr18:21851399-21852169	S_Shore
hyper1317	N_insufficient_1	6.0E-03	0.34	0.12	0.23	18	21852436	cg21840153	OSBPL1A;OSBPL1A	Body;TSS1500	chr18:21851399-21852169	S_Shore
hyper1318	N_insufficient_1	6.4E-04	0.22	0.03	0.19	18	22929808	cg25015867	ZNF521	Body	chr18:22930018-22930904	N_Shore
hyper1319	N_insufficient_1	1.5E-04	0.24	0.05	0.19	18	22930283	cg21678445	ZNF521	Body	chr18:22930018-22930904	Island
hyper1320	N_insufficient_1	1.3E-09	0.21	0.05	0.17	18	43651686	cg22038796	PSTPIP2	Body	chr18:43651796-43652455	N_Shore
hyper1321	N_insufficient_1	0.0E+00	0.20	0.02	0.19	18	43651835	cg13768737	PSTPIP2	Body	chr18:43651796-43652455	Island
hyper1322	N_insufficient_1	0.0E+00	0.24	0.02	0.22	18	43652232	cg11864882	PSTPIP2;PSTPIP2	1stExon;5'UTR	chr18:43651796-43652455	Island
hyper1323	N_insufficient_1	0.0E+00	0.32	0.01	0.31	18	43652255	cg17329670	PSTPIP2	TSS200	chr18:43651796-43652455	Island
hyper1324	N_insufficient_1	5.8E-10	0.27	0.07	0.20	18	43652366	cg21949781	PSTPIP2	TSS200	chr18:43651796-43652455	Island
hyper1325	N_insufficient_1	0.0E+00	0.22	0.05	0.17	18	43652398	cg24026435	PSTPIP2	TSS200	chr18:43651796-43652455	Island
hyper1326	N_insufficient_1	1.5E-08	0.22	0.04	0.18	18	43652430	cg03405114	PSTPIP2	TSS200	chr18:43651796-43652455	Island
hyper1327	N_insufficient_1	3.9E-10	0.20	0.06	0.14	18	43652437	cg14304186	PSTPIP2	TSS200	chr18:43651796-43652455	Island
hyper1328	N_insufficient_1	4.1E-08	0.19	0.06	0.12	18	43652454	cg26226511	PSTPIP2	TSS1500	chr18:43651796-43652455	Island
hyper1329	N_insufficient_1	3.2E-02	0.24	0.12	0.12	18	43652592	cg16730908	PSTPIP2	TSS1500	chr18:43651796-43652455	S_Shore
hyper1330	N_insufficient_1	1.3E-06	0.19	0.07	0.12	18	70211835	cg27376271	CBLN2	TSS200	chr18:70208974-70211790	S_Shore
hyper1331	N_insufficient_1	3.6E-02	0.12	0.05	0.08	19	3585609	cg26797585	GIPC3	1stExon	chr19:3585357-3585947	Island
hyper1332	N_insufficient_1	6.8E-02	0.17	0.06	0.11	19	7795619	cg13950603	CLEC4G	Body	chr19:7794793-7796410	Island
hyper1333	N_insufficient_1	1.1E-02	0.19	0.04	0.15	19	30017829	cg18777119	VSTM2B	Body	chr19:30015781-30021367	Island
hyper1334	N_insufficient_1	1.5E-04	0.16	0.05	0.11	19	45281236	cg18612786	CBLC;CBLC	1stExon;1stExon	chr19:45281133-45281355	Island
hyper1335	N_insufficient_1	3.6E-02	0.20	0.05	0.15	19	55690876	cg14047019	SYT5	5'UTR	chr19:55690182-55691623	Island
hyper1336	N_insufficient_1	3.9E-05	0.25	0.06	0.19	19	58629828	cg03145999	ZSCAN18;ZSCAN18	TSS200;TSS200	chr19:58629309-58630241	Island
hyper1337	N_insufficient_1	1.2E-03	0.27	0.07	0.19	20	10199536	cg16564824	SNAP25;SNAP25;SNAP25;SNAP	1stExon;5'UTR;1stExon;5'UTR	chr20:10200088-10200384	N_Shore
hyper1338	N_insufficient_1	4.4E-03	0.09	0.03	0.07	20	13201328	cg15577927	ISM1	TSS1500	chr20:13200670-13202616	Island
hyper1339	N_insufficient_1	5.8E-02	0.11	0.03	0.07	20	30458044	cg21578322	TTLL9;DUSP15;DUSP15;DUSP15	TSS1500;TSS200;TSS1500;Body	chr20:30457907-30458712	Island
hyper1340	N_insufficient_1	7.9E-06	0.06	0.01	0.05	20	30458061	cg16313278	TTLL9;DUSP15;DUSP15;DUSP15	TSS1500;TSS200;TSS1500;Body	chr20:30457907-30458712	Island
hyper1341	N_insufficient_1	5.8E-02	0.42	0.25	0.17	20	61809035	cg02065637	MIR124-3	TSS1500	chr20:61806254-61810867	Island
hyper1342	N_insufficient_1	2.0E-08	0.17	0.01	0.16	20	62103627	cg08003150	KCNQ2;KCNQ2;KCNQ2;KCNQ2;I	1stExon;1stExon;1stExon;1stExon	chr20:62102541-62103995	Island
hyper1343	N_insufficient_1	2.1E-02	0.19	0.08	0.11	21	28338836	cg03202077	ADAMTS5;ADAMTS5	1stExon;5'UTR	chr21:28337856-28340237	Island
hyper1344	N_insufficient_1	6.0E-06	0.11	0.02	0.09	22	19510977	cg21872764	CLDN5;CLDN5;CLDN5	1stExon;3'UTR;3'UTR	chr22:19510871-19512254	Island
hyper1345	N_insufficient_1	2.1E-03	0.17	0.08	0.08	22	21311670	cg10785109			chr22:21311336-21312201	Island
hyper1346	N_insufficient_1	3.0E-04	0.21	0.08	0.13	22	47133964	cg27326469	CERK	1stExon		
hyper1347	N_insufficient_1	5.0E-03	0.06	0.01	0.05	22	50242492	cg15219689			chr22:50241735-50243918	Island
hyper1348	N_insufficient_2	1.1E-02	0.07	0.02	0.05	1	16174780	cg06526137	SPEN;FLJ37453	Body;TSS200	chr1:16173889-16175396	Island
hyper1349	N_insufficient_2	4.9E-03	0.09	0.02	0.07	1	38512238	cg05336094	POU3F1	1stExon	chr1:38510102-38513642	Island
hyper1350	N_insufficient_2	8.6E-02	0.06	0.02	0.04	2	14772731	cg20720059	FAM84A	TSS200	chr2:14772377-14775809	Island
hyper1351	N_insufficient_2	6.6E-03	0.21	0.04	0.17	2	29033897	cg04786857	SPDYA;SPDYA	5'UTR;5'UTR	chr2:29033351-29034011	Island
hyper1352	N_insufficient_2	6.4E-05	0.11	0.02	0.09	2	219724539	cg06157334	WNT6	TSS200	chr2:219723702-219725010	Island
hyper1353	N_insufficient_2	2.2E-02	0.41	0.23	0.19	2	233404334	cg27382389	CHRNG	TSS200		
hyper1354	N_insufficient_2	5.7E-03	0.05	0.01	0.04	2	242626259	cg18361031	DTYMK;DTYMK;DTYMK;DTYMK;	5'UTR;5'UTR;1stExon;Body;1stEx	chr2:242625961-242626720	Island
hyper1355	N_insufficient_2	4.4E-05	0.05	0.01	0.05	2	242641384	cg15561235	ING5	TSS200	chr2:242640843-242642456	Island
hyper1356	N_insufficient_2	1.9E-02	0.04	0.01	0.03	3	11034279	cg11021744	SLC6A1	TSS200	chr3:11034446-11035384	N_Shore
hyper1357	N_insufficient_2	8.1E-05	0.05	0.01	0.04	3	107809973	cg12363545	CD47;CD47;CD47	TSS200;TSS200;TSS200	chr3:107809375-107810543	Island
hyper1358	N_insufficient_2	1.3E-02	0.16	0.08	0.08	3	128327499	cg08602596			chr3:128326937-128327507	Island
hyper1359	N_insufficient_2	5.4E-03	0.21	0.07	0.13	3	167813154	cg03208482	GOLIM4;GOLIM4	5'UTR;1stExon	chr3:167812829-167814102	Island
hyper1360	N_insufficient_2	3.0E-06	0.22	0.08	0.13	4	164253938	cg11064073	NPY1R	TSS200	chr4:164252954-164253471	S_Shore
hyper1361	N_insufficient_2	4.6E-04	0.18	0.08	0.09	4	164254082	cg17500103	NPY1R	TSS1500	chr4:164252954-164253471	S_Shore
hyper1362	N_insufficient_2	2.7E-09	0.24	0.05	0.19	4	164254111	cg06734271	NPY1R	TSS1500	chr4:164252954-164253471	S_Shore
hyper1363	N_insufficient_2	8.7E-03	0.04	0.02	0.03	4	166248807	cg27324455	SC4MOL;SC4MOL	TSS200;TSS200	chr4:166248454-166249063	Island
hyper1364	N_insufficient_2	5.7E-02	0.29	0.11	0.18	4	172734347	cg05295006	GALNTL6	TSS1500	chr4:172733734-172735118	Island
hyper1365	N_insufficient_2	1.1E-04	0.22	0.07	0.15	4	172734718	cg26588943	GALNTL6;GALNTL6	1stExon;5'UTR	chr4:172733734-172735118	Island
hyper1366	N_insufficient_2	2.6E-02	0.25	0.04	0.21	4	172734776	cg03413465	GALNTL6;GALNTL6	1stExon;5'UTR	chr4:172733734-172735118	Island
hyper1367	N_insufficient_2	2.4E-02	0.09	0.03	0.06	5	893022	cg05193570	TRIP13;TRIP13;BRD9;TRIP13;TR	1stExon;5'UTR;TSS200;1stExon;5	chr5:892522-893479	Island
hyper1368	N_insufficient_2	3.5E-03	0.06	0.02	0.04	5	39074677	cg25355076	RICTOR	TSS200	chr5:39073881-39075236	Island
hyper1369	N_insufficient_2	1.1E-02	0.11	0.04	0.07	5	128796595	cg21806022	ADAMTS19	Body	chr5:128795503-128797417	Island
hyper1370	N_insufficient_2	1.1E-05	0.12	0.06	0.07	5	128796702	cg01517311	ADAMTS19	Body	chr5:128795503-128797417	Island
hyper1371	N_insufficient_2	4.8E-02	0.15	0.07	0.08	5	128796754	cg18319921	ADAMTS19	Body	chr5:128795503-128797417	Island
hyper1372	N_insufficient_2	2.0E-06	0.17	0.04	0.13	5	128797013	cg10821115	ADAMTS19	Body	chr5:128795503-128797417	Island
hyper1373	N_insufficient_2	0.0E+00	0.25	0.08	0.17	6	10887367	cg03320827	SYCP2L	1stExon	chr6:10887184-10887622	Island
hyper1374	N_insufficient_2	1.0E-08	0.23	0.06	0.18	6	10887587	cg23233404	SYCP2L	Body	chr6:10887184-10887622	Island
hyper1375	N_insufficient_2	3.1E-08	0.23	0.07	0.16	6	10887672	cg09105523	SYCP2L	Body	chr6:10887184-10887622	S_Shore
hyper1376	N_insufficient_2	3.6E-02	0.26	0.10	0.16	6	29760659	cg18457685	HCG4	Body	chr6:29759946-29760903	Island
hyper1377	N_insufficient_2	9.3E-03	0.28	0.10	0.18	6	29760675	cg09112760	HCG4	Body	chr6:29759946-29760903	Island
hyper1378	N_insufficient_2	1.9E-03	0.15	0.08	0.07	6	31795241	cg12433886	HSPA1B	TSS1500	chr6:31795467-31797384	N_Shore
hyper1379	N_insufficient_2	7.4E-03	0.04	0.01	0.03	6	33217183	cg05186755			chr6:33216103-33217463	Island
hyper1380	N_insufficient_2	7.5E-02	0.09	0.03	0.06	6	152958133	cg23353893	SYNE1;SYNE1	5'UTR;TSS200	chr6:152957810-152958472	Island
hyper1381	N_insufficient_2	8.7E-06	0.64	0.43	0.20	7	94287242	cg22820921	PEG10;PEG10	5'UTR;5'UTR	chr7:94284858-94286527	S_Shore
hyper1382	N_insufficient_2	3.8E-02	0.15	0.09	0.07	8	12612631	cg04573959	LONRF1	1stExon	chr8:12611609-12613562	Island
hyper1383	N_insufficient_2	6.4E-04	0.14	0.02	0.11	8	25903852	cg06541501	EBF2	TSS1500	chr8:25900562-25905842	Island
hyper1384	N_insufficient_2	5.8E-02	0.05	0.01	0.04	8	103666201	cg08596637	KLF10;KLF10	Body;TSS200	chr8:103666157-103668861	Island
hyper1385	N_insufficient_2	0.0E+00	0.32	0.08	0.24	9	21802598	cg00230302	MTAP	TSS200	chr9:21802524-21802981	Island
hyper1386	N_insufficient_2	8.8E-02	0.06	0.01	0.05	10	99094544	cg08421900	FRAT2	TSS200	chr10:99093406-99094955	Island
hyper1387	N_insufficient_2	7.2E-02	0.07	0.02	0.05	11	65658459	cg07582047	CCDC85B	1stExon	chr11:65657874-65658970	Island
hyper1388	N_insufficient_2	6.1E-02	0.20	0.12	0.08	11	67888858	cg11343870	CHKA;CHKA	TSS200;TSS200	chr11:67887977-67889768	Island
hyper1389	N_insufficient_2	3.0E-04	0.19	0.07	0.13	11	124981114	cg01965112			chr11:124981319-124981769	N_Shore
hyper1390	N_insufficient_2	2.1E-03	0.24	0.07	0.16	11	124981320	cg05259966			chr11:124981319-124981769	Island
hyper1391	N_insufficient_2	1.5E-08	0.23	0.07	0.17	11	124981580	cg01912984			chr11:124981319-124981769	Island
hyper1392	N_insufficient_2	1.3E-09	0.23	0.04	0.19	11	124981869	cg23156348			chr11:124981319-124981769	S_Shore
hyper1393	N_insufficient_2	4.6E-03	0.15	0.06	0.09	12	19282832	cg25764464	PLEKHA5;PLEKHA5	Body;Body	chr12:19282641-19283901	Island
hyper1394	N_insufficient_2	1.9E-10	0.24	0.07	0.16	12	51566569	cg04543012	TFCP2;TFCP2	5'UTR;1stExon	chr12:51566680-51567072	N_Shore
hyper1395	N_insufficient_2	6.4E-03	0.19	0.08	0.11	12	51566731	cg19016289	TFCP2	TSS200	chr12:51566680-51567072	Island
hyper1396	N_insufficient_2	4.0E-02	0.14	0.03	0.12	12	51566756	cg13113115	TFCP2	TSS200	chr12:51566680-51567072	Island
hyper1397	N_insufficient_2	4.1E-05	0.17	0.05	0.12	12	51566772	cg13008978	TFCP2	TSS200	chr12:51566680-51567072	Island
hyper1398	N_insufficient_2	0.0E+00	0.32	0.05	0.27	12	51566859	cg18751588	TFCP2	TSS200	chr12:51566680-51567072	Island
hyper1399	N_insufficient_2	0.0E+00	0.37	0.00	0.36	12	51566990	cg22790973	TFCP2	TSS1500	chr12:51566680-51567072	Island
hyper1400	N_insufficient_2	1.9E-10	0.24	0.06	0.17	12	51567112	cg20927059	TFCP2	TSS1500	chr12:51566680-51567072	S_Shore
hyper1401	N_insufficient_2	2.0E-02	0.03	0.01	0.03	12	57623759	cg21697007	SHMT2;SHMT2;SHMT2;SHMT2;S	Body;TSS1500;Body;TSS200;TSS	chr12:57623420-57623903	Island
hyper1402	N_insufficient_2	8.2E-09	0.16	0.05	0.12	12	59989652	cg08367275			chr12:59989651-59990507	Island
hyper1403	N_insufficient_2	5.6E-06	0.10	0.03	0.07	12	59989742	cg04915780			chr12:59989651-59990507	Island
hyper1404	N_insufficient_2	3.0E-02	0.12	0.07	0.06	12	59990159	cg07614306			chr12:59989651-59990507	Island
hyper1405	N_insufficient_2	2.9E-02	0.05	0.01	0.03	14	24610583	cg21171130	FAM158A	5'UTR	chr14:24610406-24611158	Island
hyper1406	N_insufficient_2	3.4E-03	0.05	0.01	0.04	15	31507907	cg10079107			chr15:31507806-31508352	Island
hyper1407	N_insufficient_2	1.9E-02	0.08	0.02	0.06	15	72766486	cg25990494	ARIH1	TSS200	chr15:72766260-72767693	Island
hyper1408	N_insufficient_2	5.2E-02	0.04	0.01	0.03	15	102029948	cg11990980	PCSK6;PCSK6;PCSK6;PCSK6;P(	1stExon;1stExon;5'UTR;5'UTR;1st	chr15:102028860-102030608	Island
hyper1409	N_insufficient_2	2.1E-02	0.06	0.02	0.04	16	9185541	cg07393984	C16orf72;C16orf72	1stExon;5'UTR	chr16:9184146-9186689	Island
hyper1410	N_insufficient_2	7.1E-04	0.07	0.02	0.05	16	12009405	cg21875234	GSPT1;GSPT1;GSPT1	1stExon;1stExon;5'UTR	chr16:12008880-12010114	Island
hyper1411	N_insufficient_2	1.7E-02	0.05	0.01	0.04	16	28936502	cg04863568	RABEP2;RABEP2	1stExon;5'UTR	chr16:28936053-28937036	Island
hyper1412	N_insufficient_2	1.9E-07	0.08	0.01	0.07	17	27894392	cg13110711	ABHD15;TP53I13	TSS1500;TSS1500	chr17:27893086-27896078	Island
hyper1413	N_insufficient_2	7.4E-02	0.09	0.03	0.06	17	29886672	cg20286236	MIR193A	TSS1500	chr17:29885900-29887377	Island
hyper1414	N_insufficient_2	1.3E-03	0.17	0.09	0.07	17	48049475	cg08756712	DLX4;DLX4	Body;TSS1500	chr17:48048953-48050616	Island

hyper1415	N_insufficient_2	5.9E-02	0.11	0.03	0.08	18	5296031	cg07305215	ZFP161;ZFP161;ZFP161;ZFP161;	1stExon;5'UTR;1stExon;TSS1500;	chr18:5295323-5297099	Island
hyper1416	N_insufficient_2	5.5E-06	0.41	0.17	0.24	18	15197867	cg00279001			chr18:15197650-15198309	Island
hyper1417	N_insufficient_2	2.4E-07	0.54	0.14	0.40	18	15198067	cg01301382			chr18:15197650-15198309	Island
hyper1418	N_insufficient_2	1.6E-04	0.03	0.00	0.02	19	4909554	cg09317102	UHRF1;UHRF1;UHRF1	5'UTR;1stExon;TSS1500	chr19:4909262-4910256	Island
hyper1419	N_insufficient_2	7.0E-04	0.14	0.04	0.10	19	6464082	cg25637722	CRB3;CRB3	TSS200;TSS200	chr19:6463991-6464780	Island
hyper1420	N_insufficient_2	2.7E-05	0.17	0.09	0.08	19	56904755	cg11740878	ZNF582	5'UTR	chr19:56904636-56905355	Island
hyper1421	N_insufficient_2	1.6E-03	0.21	0.05	0.17	19	56905032	cg20984085	ZNF582	TSS200	chr19:56904636-56905355	Island
hyper1422	N_insufficient_2	9.0E-02	0.06	0.02	0.04	20	61847713	cg08514185	YTHDF1	TSS200	chr20:61846843-61848103	Island
hyper1423	N_insufficient_2	4.7E-04	0.08	0.01	0.07	20	62258574	cg13628960	GMEB2	TSS200	chr20:62257589-62259476	Island
hyper1424	N_insufficient_2	2.4E-06	0.06	0.01	0.05	22	30753142	cg18031436	SF3A1;SF3A1;CCDC157	TSS1500;TSS1500;5'UTR	chr22:30752583-30753214	Island
hyper1425	N_insufficient_2	7.1E-05	0.05	0.01	0.03	22	42915677	cg13919496	RRP7A	Body	chr22:42915512-42915999	Island
hyper1426	N_insufficient_3	7.6E-04	0.05	0.01	0.04	1	26799365	cg13064914	HMGN2	Body	chr1:26798366-26799842	Island
hyper1427	N_insufficient_3	6.9E-02	0.10	0.03	0.07	1	70033739	cg23730181			chr1:70032967-70034495	Island
hyper1428	N_insufficient_3	2.3E-02	0.15	0.09	0.06	1	70033902	cg21004042			chr1:70032967-70034495	Island
hyper1429	N_insufficient_3	7.5E-04	0.18	0.10	0.08	1	115632038	cg03614513	TSPAN2	1stExon	chr1:115631850-115632584	Island
hyper1430	N_insufficient_3	9.6E-04	0.04	0.00	0.03	1	115632236	cg01522456	TSPAN2	TSS200	chr1:115631850-115632584	Island
hyper1431	N_insufficient_3	9.4E-05	0.13	0.04	0.09	1	115632455	cg06043114	TSPAN2	TSS1500	chr1:115631850-115632584	Island
hyper1432	N_insufficient_3	8.7E-05	0.12	0.04	0.09	1	154297643	cg06824583	ATP8B2;AQP10	TSS1500;3'UTR	chr1:154298205-154298544	N_Shore
hyper1433	N_insufficient_3	3.0E-02	0.28	0.11	0.18	1	154297985	cg12644885	ATP8B2	TSS200	chr1:154298205-154298544	N_Shore
hyper1434	N_insufficient_3	3.3E-02	0.30	0.04	0.25	1	214160848	cg23992410	PROX1	TSS1500	chr1:214160798-214161034	Island
hyper1435	N_insufficient_3	2.9E-10	0.11	0.01	0.10	1	214161251	cg25347098	PROX1	TSS1500	chr1:214161197-214161415	Island
hyper1436	N_insufficient_3	3.8E-02	0.18	0.09	0.09	1	214161306	cg14019317	PROX1	TSS1500	chr1:214161197-214161415	Island
hyper1437	N_insufficient_3	7.4E-05	0.11	0.02	0.09	1	214161371	cg23260547	PROX1	TSS1500	chr1:214161197-214161415	Island
hyper1438	N_insufficient_3	4.1E-03	0.20	0.08	0.12	1	218520325	cg06899755	TGFB2;TGFB2	1stExon;1stExon	chr1:218520287-218520506	Island
hyper1439	N_insufficient_3	5.6E-04	0.13	0.03	0.10	1	218520468	cg22021178	TGFB2;TGFB2	Body;Body	chr1:218520287-218520506	Island
hyper1440	N_insufficient_3	5.7E-09	0.26	0.02	0.24	1	239550303	cg13570831			chr1:239550108-239551339	Island
hyper1441	N_insufficient_3	1.4E-09	0.10	0.01	0.10	1	245317971	cg11586600	KIF26B	TSS1500	chr1:245316547-245320247	Island
hyper1442	N_insufficient_3	1.0E-04	0.12	0.02	0.10	1	245317980	cg09065617	KIF26B	TSS1500	chr1:245316547-245320247	Island
hyper1443	N_insufficient_3	1.2E-06	0.18	0.01	0.16	1	245318209	cg01787565	KIF26B	TSS200	chr1:245316547-245320247	Island
hyper1444	N_insufficient_3	1.5E-03	0.14	0.04	0.10	1	245318229	cg12054521	KIF26B	TSS200	chr1:245316547-245320247	Island
hyper1445	N_insufficient_3	1.2E-06	0.15	0.04	0.11	1	245318245	cg13888026	KIF26B	TSS200	chr1:245316547-245320247	Island
hyper1446	N_insufficient_3	5.8E-09	0.14	0.02	0.12	1	245318335	cg19252551	KIF26B;KIF26B	1stExon;5'UTR	chr1:245316547-245320247	Island
hyper1447	N_insufficient_3	5.2E-03	0.10	0.04	0.06	1	245318734	cg03284440	KIF26B	1stExon	chr1:245316547-245320247	Island
hyper1448	N_insufficient_3	2.6E-06	0.08	0.02	0.07	1	245318817	cg02054059	KIF26B	Body	chr1:245316547-245320247	Island
hyper1449	N_insufficient_3	3.1E-06	0.09	0.02	0.07	2	60776713	cg16874579	BCL11A;BCL11A;BCL11A	Body;Body;Body	chr2:60776596-60778157	Island
hyper1450	N_insufficient_3	1.6E-06	0.20	0.03	0.17	2	60777051	cg26580134	BCL11A;BCL11A;BCL11A	Body;Body;Body	chr2:60776596-60778157	Island
hyper1451	N_insufficient_3	1.9E-05	0.21	0.09	0.12	2	60777279	cg00732907	BCL11A;BCL11A;BCL11A	Body;Body;Body	chr2:60776596-60778157	Island
hyper1452	N_insufficient_3	2.4E-05	0.28	0.05	0.23	2	159173778	cg21514997	CCDC148;CCDC148	Body;Body		
hyper1453	N_insufficient_3	2.5E-04	0.28	0.05	0.24	2	239140169	cg07323141	LOC151174;LOC643387;LOC151	Body;TSS200;Body	chr2:239140096-239140659	Island
hyper1454	N_insufficient_3	5.1E-04	0.36	0.02	0.34	2	239140182	cg23497383	LOC151174;LOC643387;LOC151	Body;TSS200;Body	chr2:239140096-239140659	Island
hyper1455	N_insufficient_3	6.5E-03	0.32	0.02	0.30	2	239140190	cg13530938	LOC151174;LOC643387;LOC151	Body;TSS200;Body	chr2:239140096-239140659	Island
hyper1456	N_insufficient_3	1.5E-03	0.12	0.05	0.07	3	38080790	cg00807586	DLEC1;DLEC1	1stExon;1stExon	chr3:38080628-38081187	Island
hyper1457	N_insufficient_3	5.3E-02	0.14	0.08	0.06	3	48700425	cg12091542	CELSR3	TSS200	chr3:48698335-48701667	Island
hyper1458	N_insufficient_3	5.8E-10	0.25	0.01	0.24	3	115503520	cg18176703			chr3:115502725-115503581	Island
hyper1459	N_insufficient_3	3.4E-08	0.19	0.03	0.16	3	132757741	cg19430967	TMEM108;TMEM108	5'UTR;5'UTR	chr3:132756985-132757895	Island
hyper1460	N_insufficient_3	9.4E-04	0.12	0.03	0.09	3	142839578	cg08774368	CHST2	5'UTR	chr3:142837885-142840838	Island
hyper1461	N_insufficient_3	9.0E-04	0.05	0.01	0.04	4	926294	cg19987229	TMEM175;TMEM175;GAK	1stExon;5'UTR;TSS200	chr4:925580-926488	Island
hyper1462	N_insufficient_3	1.2E-06	0.06	0.01	0.05	4	85504051	cg03403966	CDS1	TSS200	chr4:85503546-85504893	Island
hyper1463	N_insufficient_3	3.8E-03	0.25	0.06	0.19	4	109089890	cg05354432	LEF1;LEF1;LEF1;LEF1;LEF1;LEF	5'UTR;1stExon;5'UTR;1stExon;5'U	chr4:109087747-109090407	Island
hyper1464	N_insufficient_3	2.9E-04	0.19	0.05	0.14	4	113436625	cg08929548	NEUROG2	Body	chr4:113435898-113438002	Island
hyper1465	N_insufficient_3	2.3E-05	0.20	0.06	0.15	5	53545	cg05648010			chr5:53111-54264	Island
hyper1466	N_insufficient_3	0.0E+00	0.27	0.03	0.23	5	53564	cg22807585			chr5:53111-54264	Island
hyper1467	N_insufficient_3	2.0E-03	0.17	0.08	0.09	5	53886	cg14101494			chr5:53111-54264	Island
hyper1468	N_insufficient_3	3.5E-03	0.21	0.06	0.14	5	60627447	cg02486078	ZSWIM6	TSS1500	chr5:60626505-60629809	Island
hyper1469	N_insufficient_3	3.3E-06	0.31	0.09	0.22	5	60629795	cg07939285	ZSWIM6	Body	chr5:60626505-60629809	Island
hyper1470	N_insufficient_3	3.5E-02	0.31	0.09	0.22	5	159200479	cg00028918			chr5:159200237-159200692	Island
hyper1471	N_insufficient_3	2.8E-08	0.29	0.06	0.23	6	1609827	cg03869498	FOXC1	TSS1500	chr6:1604606-1615866	Island
hyper1472	N_insufficient_3	5.8E-02	0.28	0.11	0.16	6	1610102	cg20020464	FOXC1	TSS1500	chr6:1604606-1615866	Island
hyper1473	N_insufficient_3	4.4E-07	0.26	0.05	0.21	6	1610120	cg23572376	FOXC1	TSS1500	chr6:1604606-1615866	Island
hyper1474	N_insufficient_3	6.2E-05	0.13	0.06	0.07	6	1610197	cg23867624	FOXC1	TSS1500	chr6:1604606-1615866	Island
hyper1475	N_insufficient_3	0.0E+00	0.25	0.02	0.23	6	1610669	cg04780909	FOXC1	TSS200	chr6:1604606-1615866	Island
hyper1476	N_insufficient_3	3.1E-07	0.32	0.13	0.20	6	1610673	cg11815762	FOXC1	TSS200	chr6:1604606-1615866	Island
hyper1477	N_insufficient_3	1.1E-05	0.37	0.11	0.26	6	1611451	cg20491838	FOXC1	1stExon	chr6:1604606-1615866	Island
hyper1478	N_insufficient_3	1.9E-10	0.17	0.02	0.16	6	1611712	cg01912921	FOXC1	1stExon	chr6:1604606-1615866	Island
hyper1479	N_insufficient_3	1.6E-03	0.25	0.10	0.15	6	1611850	cg10365447	FOXC1	1stExon	chr6:1604606-1615866	Island
hyper1480	N_insufficient_3	4.6E-08	0.17	0.02	0.15	6	3227751	cg11522018	TUBB2B	1stExon	chr6:3227025-3229688	Island
hyper1481	N_insufficient_3	1.0E-02	0.13	0.03	0.10	6	3227949	cg10926113	TUBB2B;TUBB2B	1stExon;5'UTR	chr6:3227025-3229688	Island
hyper1482	N_insufficient_3	6.3E-02	0.08	0.03	0.05	6	13486415	cg21348771	C6orf114;GFOD1	TSS200;Body	chr6:13486324-13488792	Island
hyper1483	N_insufficient_3	8.4E-03	0.13	0.05	0.09	6	32939317	cg17013193	BRD2;BRD2	5'UTR;5'UTR	chr6:32939275-32940954	Island
hyper1484	N_insufficient_3	9.1E-05	0.12	0.02	0.10	6	32939335	cg10061292	BRD2;BRD2	5'UTR;5'UTR	chr6:32939275-32940954	Island
hyper1485	N_insufficient_3	1.1E-05	0.10	0.02	0.08	6	32939364	cg07211678	BRD2;BRD2	5'UTR;5'UTR	chr6:32939275-32940954	Island
hyper1486	N_insufficient_3	6.6E-07	0.14	0.02	0.11	6	32939426	cg26226566	BRD2;BRD2	5'UTR;5'UTR	chr6:32939275-32940954	Island
hyper1487	N_insufficient_3	3.0E-03	0.16	0.08	0.08	6	32939507	cg21174644	BRD2;BRD2	5'UTR;5'UTR	chr6:32939275-32940954	Island
hyper1488	N_insufficient_3	1.7E-07	0.14	0.05	0.09	6	46702683	cg04454506	PLA2G7;PLA2G7	5'UTR;5'UTR	chr6:46702736-46703316	N_Shore
hyper1489	N_insufficient_3	4.6E-09	0.22	0.06	0.16	6	46702790	cg21269330	PLA2G7;PLA2G7	5'UTR;5'UTR	chr6:46702736-46703316	Island
hyper1490	N_insufficient_3	0.0E+00	0.20	0.00	0.20	6	46702983	cg18630040	PLA2G7;PLA2G7;PLA2G7	1stExon;5'UTR;5'UTR	chr6:46702736-46703316	Island
hyper1491	N_insufficient_3	2.2E-03	0.19	0.05	0.14	6	110679107	cg10259748	C6orf186	Body	chr6:110678854-110679108	Island
hyper1492	N_insufficient_3	3.4E-05	0.16	0.02	0.14	6	166401700	cg02465374	LOC441177;C6orf176;C6orf176	Body;TSS200;TSS200	chr6:166401526-166402659	Island
hyper1493	N_insufficient_3	7.8E-03	0.31	0.09	0.22	6	166419641	cg12856646			chr6:166421891-166422241	N_Shelf
hyper1494	N_insufficient_3	1.1E-02	0.22	0.09	0.13	6	168719954	cg05070558	DACT2	Body	chr6:168719681-168720495	Island
hyper1495	N_insufficient_3	3.4E-02	0.16	0.08	0.08	7	64734517	cg26337123			chr7:64734488-64734692	Island
hyper1496	N_insufficient_3	4.0E-02	0.25	0.08	0.17	7	95226035	cg19329121	PDK4	TSS200	chr7:95225503-95226194	Island
hyper1497	N_insufficient_3	2.9E-02	0.10	0.04	0.06	7	100291184	cg05401159			chr7:100291183-100292266	Island
hyper1498	N_insufficient_3	1.2E-02	0.20	0.03	0.17	7	112726653	cg03119639	GPR85;GPR85;GPR85;GPR85	5'UTR;TSS1500;TSS200;5'UTR		
hyper1499	N_insufficient_3	2.1E-02	0.22	0.04	0.18	7	140773578	cg15928538			chr7:140772358-140774560	Island
hyper1500	N_insufficient_3	1.4E-07	0.19	0.03	0.16	7	140773905	cg07028821			chr7:140772358-140774560	Island
hyper1501	N_insufficient_3	2.0E-07	0.31	0.11	0.20	7	140774104	cg09344986			chr7:140772358-140774560	Island
hyper1502	N_insufficient_3	2.3E-09	0.26	0.02	0.25	7	140774161	cg18750086			chr7:140772358-140774560	Island
hyper1503	N_insufficient_3	2.8E-04	0.27	0.03	0.23	7	140774522	cg17554604			chr7:140772358-140774560	Island
hyper1504	N_insufficient_3	1.3E-07	0.20	0.04	0.16	8	6691879	cg21493703	XKR5	Body	chr8:6691833-6693135	Island
hyper1505	N_insufficient_3	3.6E-07	0.28	0.08	0.20	8	6692470	cg05234760	XKR5	Body	chr8:6691833-6693135	Island
hyper1506	N_insufficient_3	4.4E-02	0.14	0.09	0.06	8	16859451	cg11192975	FGF20	1stExon	chr8:16859044-16859452	Island
hyper1507	N_insufficient_3	9.6E-04	0.04	0.01	0.03	8	22014129	cg01039695	LGI3;LGI3	1stExon;5'UTR	chr8:22014152-22014559	N_Shore
hyper1508	N_insufficient_3	1.0E-05	0.13	0.03	0.10	8	41166990	cg24319902	SFRP1	TSS200	chr8:41165852-41167140	Island
hyper1509	N_insufficient_3	1.0E-02	0.19	0.09	0.11	8	41167087	cg01495122	SFRP1	TSS200	chr8:41165852-41167140	Island
hyper1510	N_insufficient_3	1.1E-03	0.09	0.02	0.07	8	41167107	cg21517947	SFRP1	TSS200	chr8:41165852-41167140	Island
hyper1511	N_insufficient_3	2.4E-02	0.14	0.05	0.10	8	41167109	cg17816908	SFRP1	TSS200	chr8:41165852-41167140	Island
hyper1512	N_insufficient_3	1.3E-02	0.20	0.07	0.14	8	70747067	cg08419026	SLCO5A1;SLCO5A1	5'UTR;TSS1500	chr8:70744098-70747441	Island
hyper1513	N_insufficient_3	5.4E-02	0.26	0.09	0.17	8	140716495	cg01717150	KCNK9	TSS1500	chr8:140714585-140718259	Island
hyper1514	N_insufficient_3	2.1E-02	0.30	0.12	0.18	8	140716505	cg16595365	KCNK9	TSS1500	chr8:140714585-140718259	Island
hyper1515	N_insufficient_3	9.1E-04	0.02	0.00	0.02	8	145150394	cg25803766	CYC1	Body	chr8:145149550-145150440	Island
hyper1516	N_insufficient_3	2.7E-02	0.15	0.04	0.11	9	114434	cg15543173			chr9:112916-114839	Island
hyper1517	N_insufficient_3	2.1E-09	0.36	0.11	0.25	9	38424431	cg16918846	IGFBPL1;IGFBPL1	5'UTR;1stExon	chr9:38423947-38424584	Island
hyper1518	N_insufficient_3	2.3E-05	0.18	0.09	0.10	9	77112187	cg02908587	RORB	TSS200	chr9:77112712-77113583	N_Shore
hyper1519	N_insufficient_3	6.2E-02	0.16	0.02	0.14	9	113341925	cg13632816	SVEP1;SVEP1	1stExon;5'UTR	chr9:113341213-113342029	Island
hyper1520	N_insufficient_3	0.0E+00	0.26	0.02	0.23	10	22634432	cg05099508	SPAG6;SPAG6;SPAG6;SPAG6	1stExon;5'UTR;5'UTR;1stExon	chr10:22634000-22634862	Island
hyper1521	N_insufficient_3	0.0E+00	0.30	0.02	0.28	10	22634439	cg24031355	SPAG6;SPAG6;SPAG6;SPAG6	1stExon;5'UTR;5'UTR;1stExon	chr10:22634000-22634862	Island
hyper1522	N_insufficient_3	4.6E-09	0.08	0.01	0.07	10	64565336	cg20721121	ADO	1stExon	chr10:64564435-64565818	Island
hyper1523	N_insufficient_3	8.6E-06	0.23	0.08	0.15	10	86300587	cg02514686			chr10:86300399-86300953	Island
hyper1524	N_insufficient_3	4.0E-05	0.22	0.03	0.19	10	86300605	cg19732605			chr10:86300399-86300953	Island
hyper1525	N_insufficient_3	0.0E+00	0.35	0.03	0.32	11	13300592	cg15603424	ARNTL;ARNTL;ARNTL	5'UTR;5'UTR;5'UTR	chr11:13298796-13300735	Island
hyper1526	N_insufficient_3	2.9E-05	0.22	0.02	0.20	11	43602845	cg15556502	MIR129-2	TSS200	chr11:43602545-43603215	Island
hyper1527	N_insufficient_3	5.0E-06	0.21	0.02	0.18	11	43602847	cg14416371	MIR129-2	TSS200	chr11:43602545-43603215	Island
hyper1528	N_insufficient_3	1.5E-04	0.15	0.04	0.11	11	43602857	cg14944647	MIR129-2	TSS200	chr11:43602545-43603215	Island
hyper1529	N_insufficient_3	8.2E-03	0.17	0.02	0.15	11	43602879	cg01939477	MIR129-2	TSS200	chr11:43602545-43603215	Island
hyper1530	N_insufficient_3	2.2E-02	0.19	0.04	0.15	11	63768040	cg03423149	MACROD1	Body	chr11:63766058-63768666	Island
hyper1531	N_insufficient_3	2.1E-02	0.14	0.05	0.09	12	562272	cg03879460			chr12:562221-563234	Island
hyper1532	N_insufficient_3	4.4E-02	0.14	0.06	0.08	12	76741916	cg12013332	BBS10	Body	chr12:76741909-76742286	Island
hyper1533	N_insufficient_3	7.0E-07	0.25	0.07	0.18	12	76742097	cg10027114	BBS10	1stExon	chr12:76741909-76742286	Island
hyper1534	N_insufficient_3	9.4E-07	0.22	0.08	0.14	12-Jan	76742306	cg18530189	BBS10	TSS200	chr12:76741909-76742286	S_Shore
hyper1535	N_insufficient_3	4.3E-07	0.16	0.07	0.09	12-Jan	76742361	cg26248486	BBS10	TSS200	chr12:76741909-76742286	S_Shore
hyper1536	N_insufficient_3	3.0E-07	0.19	0.07	0.12	12-Jan	76742363	cg21360685	BBS10	TSS200	chr12:76741909-76742286	S_Shore
hyper1537	N_insufficient_3	2.0E-05	0.23	0.06	0.18	12	113903210	cg10229294	LHX5	Body	chr12:113900750-113906442	Island
hyper1538	N_insufficient_3	4.0E-03	0.25	0.06	0.19	12	113903232	cg11527326	LHX5	Body	chr12:113900750-113906442	Island
hyper1539	N_insufficient_3	4.5E-05	0.15	0.06	0.09	13	101240654	cg02162202			chr13:101240689-101241226	N_Shore
hyper1540	N_insufficient_3	4.2E-07	0.17	0.05	0.12	13	101240690	cg18976765			chr13:101240689-101241226	Island
hyper1541	N_insufficient_3	4.9E-03	0.24	0.08	0.15	14	52780921	cg08423052	PTGER2	TSS200	chr14:52780687-52781969	Island
hyper1542	N_insufficient_3	1.7E-03	0.62	0.31	0.31	15	34781705	cg18556777				
hyper1543	N_insufficient_3	6.7E-02	0.62	0.25	0.37	15	34806349	cg13761724			chr15:34806490-34807382	N_Shore

hyper1544	N_insufficient_3	7.2E-02	0.22	0.04	0.18	15	40212176	cg08364767	GPR176	1stExon	chr15:40211961-40213444	Island
hyper1545	N_insufficient_3	6.9E-02	0.24	0.10	0.14	15	40212188	cg11119742	GPR176	1stExon	chr15:40211961-40213444	Island
hyper1546	N_insufficient_3	3.9E-03	0.16	0.04	0.12	15	60296159	cg26268621	FOXB1	TSS1500	chr15:60296135-60298520	Island
hyper1547	N_insufficient_3	6.4E-09	0.24	0.02	0.22	15	60296169	cg16611005	FOXB1	TSS1500	chr15:60296135-60298520	Island
hyper1548	N_insufficient_3	9.1E-05	0.09	0.02	0.06	15	84034416	cg06842989				
hyper1549	N_insufficient_3	4.2E-07	0.15	0.05	0.10	15	96960214	cg11852671			chr15:96959341-96960531	Island
hyper1550	N_insufficient_3	6.7E-07	0.22	0.05	0.17	15	96960331	cg26470794			chr15:96959341-96960531	Island
hyper1551	N_insufficient_3	6.4E-07	0.20	0.09	0.11	15	96960392	cg20387258			chr15:96959341-96960531	Island
hyper1552	N_insufficient_3	7.5E-06	0.18	0.06	0.11	15	96960574	cg18496247			chr15:96959341-96960531	S_Shore
hyper1553	N_insufficient_3	8.1E-03	0.18	0.09	0.10	16	25703018	cg02390209	HS3ST4	TSS1500	chr16:25702955-25704631	Island
hyper1554	N_insufficient_3	2.3E-06	0.19	0.06	0.13	16	54320670	cg24662961	IRX3	TSS1500	chr16:54317821-54324604	Island
hyper1555	N_insufficient_3	3.3E-04	0.03	0.00	0.03	16	67694967	cg06287927	ACD;ACD;PARD6A;ACD;PARD6	TSS1500;TSS1500;1stExon;TSS1	chr16:67693451-67695560	Island
hyper1556	N_insufficient_3	7.0E-04	0.18	0.02	0.15	17	35293930	cg24761507	LHX1	TSS1500	chr17:35291899-35300875	Island
hyper1557	N_insufficient_3	7.4E-05	0.22	0.04	0.18	17	35294230	cg12279877	LHX1	TSS1500	chr17:35291899-35300875	Island
hyper1558	N_insufficient_3	0.0E+00	0.24	0.03	0.21	17	40333464	cg26949829	KCNH4	TSS200	chr17:40332597-40333471	Island
hyper1559	N_insufficient_3	6.3E-05	0.17	0.06	0.12	17	45056008	cg22674412	RPRML;RPRML	3'UTR;1stExon	chr17:45055317-45056838	Island
hyper1560	N_insufficient_3	0.0E+00	0.29	0.03	0.26	17	54991456	cg20676542	TRIM25	TSS200	chr17:54990832-54991771	Island
hyper1561	N_insufficient_3	1.5E-03	0.22	0.08	0.14	17	71898264	cg13990321			chr17:71897970-71898420	Island
hyper1562	N_insufficient_3	2.3E-05	0.11	0.02	0.09	18	78005649	cg05872073	PARD6G	TSS1500	chr18:78004028-78005438	S_Shore
hyper1563	N_insufficient_3	5.7E-03	0.14	0.05	0.09	18	78005665	cg00870662	PARD6G	TSS1500	chr18:78004028-78005438	S_Shore
hyper1564	N_insufficient_3	2.7E-07	0.28	0.08	0.20	19	10120092	cg12122464	COL5A3	Body	chr19:10120750-10121231	N_Shore
hyper1565	N_insufficient_3	4.8E-02	0.19	0.12	0.07	19	17536024	cg22641093	FAM125A	3'UTR		
hyper1566	N_insufficient_3	1.4E-06	0.23	0.07	0.17	19	35615444	cg04778274	LGI4	3'UTR	chr19:35615412-35615665	Island
hyper1567	N_insufficient_3	5.9E-03	0.20	0.08	0.12	19	35615579	cg11620066	LGI4	3'UTR	chr19:35615412-35615665	Island
hyper1568	N_insufficient_3	0.0E+00	0.14	0.01	0.13	19	35615638	cg27316970	LGI4	3'UTR	chr19:35615412-35615665	Island
hyper1569	N_insufficient_3	4.3E-02	0.15	0.08	0.07	19	58951778	cg24366702	ZNF132	TSS200	chr19:58951214-58952250	Island
hyper1570	N_insufficient_3	2.3E-02	0.05	0.01	0.03	19	58951814	cg00868383	ZNF132	TSS1500	chr19:58951214-58952250	Island
hyper1571	N_insufficient_3	6.8E-03	0.18	0.05	0.13	19	58951885	cg07878486	ZNF132	TSS1500	chr19:58951214-58952250	Island
hyper1572	N_insufficient_4	5.9E-06	0.23	0.03	0.20	1	35247397	cg18262616	GJB3;GJB3	TSS1500;5'UTR	chr1:35246876-35247599	Island
hyper1573	N_insufficient_4	2.1E-04	0.18	0.05	0.13	1	240161249	cg13239420			chr1:240160837-240161616	Island
hyper1574	N_insufficient_4	0.0E+00	0.16	0.01	0.15	1	240161445	cg21854408			chr1:240160837-240161616	Island
hyper1575	N_insufficient_4	2.4E-07	0.25	0.09	0.16	2	133174094	cg23410738	GPR39	TSS200	chr2:133173532-133174369	Island
hyper1576	N_insufficient_4	3.5E-03	0.32	0.11	0.21	2	133174117	cg13136510	GPR39	TSS200	chr2:133173532-133174369	Island
hyper1577	N_insufficient_4	0.0E+00	0.24	0.02	0.22	2	133174136	cg22866015	GPR39	TSS200	chr2:133173532-133174369	Island
hyper1578	N_insufficient_4	2.8E-06	0.16	0.04	0.12	2	133174168	cg02570563	GPR39;GPR39	1stExon;5'UTR	chr2:133173532-133174369	Island
hyper1579	N_insufficient_4	1.9E-03	0.07	0.02	0.05	2	175547399	cg25075147	WIPF1	5'UTR	chr2:175546862-175547880	Island
hyper1580	N_insufficient_4	7.9E-02	0.13	0.07	0.06	3	79067437	cg01793593	ROBO1;ROBO1;ROBO1;ROBO1	Body;Body;Body;Body	chr3:79067882-79068566	N_Shore
hyper1581	N_insufficient_4	1.3E-02	0.10	0.02	0.07	3	79067920	cg07467520	ROBO1;ROBO1;ROBO1;ROBO1;	1stExon;1stExon;1stExon;5'UTR;5	chr3:79067882-79068566	Island
hyper1582	N_insufficient_4	3.4E-02	0.14	0.06	0.08	3	79068292	cg15709235	ROBO1;ROBO1;ROBO1;ROBO1;	1stExon;1stExon;1stExon;5'UTR;5	chr3:79067882-79068566	Island
hyper1583	N_insufficient_4	2.5E-02	0.14	0.09	0.05	3	79068813	cg27221334	ROBO1;ROBO1;ROBO1;ROBO1	TSS1500;TSS1500;Body;TSS150	chr3:79067882-79068566	S_Shore
hyper1584	N_insufficient_4	1.6E-07	0.13	0.04	0.10	3	79068831	cg24784794	ROBO1;ROBO1;ROBO1;ROBO1	TSS1500;TSS1500;Body;TSS150	chr3:79067882-79068566	S_Shore
hyper1585	N_insufficient_4	6.8E-02	0.14	0.09	0.04	3	79068857	cg08233594	ROBO1;ROBO1;ROBO1;ROBO1	TSS1500;TSS1500;Body;TSS150	chr3:79067882-79068566	S_Shore
hyper1586	N_insufficient_4	1.6E-06	0.44	0.06	0.38	4	39448975	cg13732582	KLB	Body	chr4:39448184-39449054	Island
hyper1587	N_insufficient_4	1.2E-04	0.14	0.02	0.12	4	141419169	cg09064304			chr4:141419007-141419504	Island
hyper1588	N_insufficient_4	1.4E-05	0.09	0.02	0.07	5	1931251	cg03595755			chr5:1930754-1931986	Island
hyper1589	N_insufficient_4	4.9E-02	0.30	0.05	0.25	5	5140646	cg22784954	ADAMTS16	1stExon	chr5:5139441-5140941	Island
hyper1590	N_insufficient_4	1.4E-02	0.10	0.04	0.06	5	149682233	cg25780607	ARSI;ARSI	5'UTR;1stExon	chr5:149681702-149682601	Island
hyper1591	N_insufficient_4	6.9E-04	0.12	0.06	0.05	6	30180785	cg02957918	TRIM26	5'UTR	chr6:30180933-30182187	N_Shore
hyper1592	N_insufficient_4	7.6E-03	0.15	0.07	0.08	6	30180804	cg26560341	TRIM26	5'UTR	chr6:30180933-30182187	N_Shore
hyper1593	N_insufficient_4	3.2E-04	0.17	0.08	0.09	6	30180820	cg13083523	TRIM26	5'UTR	chr6:30180933-30182187	N_Shore
hyper1594	N_insufficient_4	7.1E-04	0.11	0.04	0.07	6	30180863	cg16392144	TRIM26	5'UTR	chr6:30180933-30182187	N_Shore
hyper1595	N_insufficient_4	2.5E-04	0.13	0.02	0.11	6	30180962	cg18068919	TRIM26	5'UTR	chr6:30180933-30182187	Island
hyper1596	N_insufficient_4	2.8E-06	0.22	0.05	0.18	6	30181014	cg10793756	TRIM26	5'UTR	chr6:30180933-30182187	Island
hyper1597	N_insufficient_4	1.1E-05	0.30	0.05	0.24	6	30181025	cg16797983	TRIM26	5'UTR	chr6:30180933-30182187	Island
hyper1598	N_insufficient_4	1.1E-04	0.32	0.07	0.25	6	30181032	cg18556540	TRIM26	5'UTR	chr6:30180933-30182187	Island
hyper1599	N_insufficient_4	8.7E-06	0.28	0.06	0.23	6	30181036	cg16808910	TRIM26	5'UTR	chr6:30180933-30182187	Island
hyper1600	N_insufficient_4	3.4E-05	0.03	0.00	0.03	6	30181173	cg12103149	TRIM26	TSS200	chr6:30180933-30182187	Island
hyper1601	N_insufficient_4	0.0E+00	0.16	0.01	0.15	6	30181175	cg05864696	TRIM26	TSS200	chr6:30180933-30182187	Island
hyper1602	N_insufficient_4	3.8E-04	0.09	0.02	0.07	6	30181183	cg20067477	TRIM26	TSS200	chr6:30180933-30182187	Island
hyper1603	N_insufficient_4	2.9E-06	0.12	0.02	0.10	6	30181189	cg26937613	TRIM26	TSS200	chr6:30180933-30182187	Island
hyper1604	N_insufficient_4	3.7E-07	0.15	0.03	0.12	6	30181194	cg13118072	TRIM26	TSS200	chr6:30180933-30182187	Island
hyper1605	N_insufficient_4	2.3E-02	0.25	0.11	0.13	6	30181212	cg01962105	TRIM26	TSS200	chr6:30180933-30182187	Island
hyper1606	N_insufficient_4	3.8E-05	0.24	0.07	0.17	6	30181302	cg09084877	TRIM26	TSS200	chr6:30180933-30182187	Island
hyper1607	N_insufficient_4	6.0E-09	0.28	0.04	0.25	6	30181335	cg02210602	TRIM26	TSS200	chr6:30180933-30182187	Island
hyper1608	N_insufficient_4	1.9E-08	0.25	0.03	0.23	6	30181383	cg24243375	TRIM26	TSS1500	chr6:30180933-30182187	Island
hyper1609	N_insufficient_4	1.7E-08	0.22	0.08	0.14	6	30181425	cg10037053	TRIM26	TSS1500	chr6:30180933-30182187	Island
hyper1610	N_insufficient_4	1.5E-05	0.15	0.05	0.09	6	30181448	cg14694232	TRIM26	TSS1500	chr6:30180933-30182187	Island
hyper1611	N_insufficient_4	5.7E-06	0.19	0.06	0.13	6	30181488	cg04274844	TRIM26	TSS1500	chr6:30180933-30182187	Island
hyper1612	N_insufficient_4	1.2E-06	0.13	0.04	0.08	6	30181645	cg18841691	TRIM26	TSS1500	chr6:30180933-30182187	Island
hyper1613	N_insufficient_4	3.9E-04	0.12	0.02	0.09	6	43970084	cg25360181	C6orf223	Body	chr6:43970073-43970922	Island
hyper1614	N_insufficient_4	5.2E-05	0.14	0.06	0.08	6	46097650	cg14229611	ENPP4	TSS200	chr6:46097694-46097953	N_Shore
hyper1615	N_insufficient_4	2.6E-03	0.32	0.15	0.17	6	46097671	cg23634318	ENPP4	TSS200	chr6:46097694-46097953	N_Shore
hyper1616	N_insufficient_4	9.3E-05	0.32	0.07	0.25	6	46097695	cg06768203	ENPP4	TSS200	chr6:46097694-46097953	Island
hyper1617	N_insufficient_4	1.7E-02	0.12	0.03	0.10	6	46097784	cg08211306	ENPP4;ENPP4	5'UTR;1stExon	chr6:46097694-46097953	Island
hyper1618	N_insufficient_4	1.1E-03	0.13	0.02	0.11	6	100062572	cg18900649	PRDM13	Body	chr6:100060979-100062657	Island
hyper1619	N_insufficient_4	1.2E-02	0.16	0.05	0.11	6	170604676	cg20745457			chr6:170604411-170606479	Island
hyper1620	N_insufficient_4	1.3E-08	0.19	0.05	0.14	6	170605003	cg02134248			chr6:170604411-170606479	Island
hyper1621	N_insufficient_4	1.9E-10	0.31	0.03	0.28	6	170605623	cg12123671			chr6:170604411-170606479	Island
hyper1622	N_insufficient_4	3.7E-02	0.12	0.04	0.08	7	24796981	cg07320646	DFNA5;DFNA5;DFNA5	5'UTR;5'UTR;5'UTR	chr7:24796541-24797487	Island
hyper1623	N_insufficient_4	8.2E-02	0.24	0.05	0.19	7	24797192	cg07293520	DFNA5;DFNA5;DFNA5	5'UTR;5'UTR;TSS200	chr7:24796541-24797487	Island
hyper1624	N_insufficient_4	8.8E-03	0.16	0.01	0.14	7	91510198	cg00390769	MTERF	TSS200	chr7:91509997-91510427	Island
hyper1625	N_insufficient_4	1.2E-04	0.15	0.03	0.12	8	21924153	cg23897733	EPB49;EPB49;EPB49;EPB49;EPI	5'UTR;5'UTR;5'UTR;5'UTR;5'UTR	chr8:21923840-21924633	Island
hyper1626	N_insufficient_4	2.8E-07	0.16	0.04	0.12	8	27629759	cg02032552	CCDC25	Body	chr8:27629753-27630234	Island
hyper1627	N_insufficient_4	0.0E+00	0.26	0.02	0.24	8	27629879	cg14666006	CCDC25	Body	chr8:27629753-27630234	Island
hyper1628	N_insufficient_4	7.0E-08	0.25	0.08	0.17	8	27630019	cg08773844	CCDC25	Body	chr8:27629753-27630234	Island
hyper1629	N_insufficient_4	7.2E-03	0.12	0.05	0.07	8	27630342	cg13467649	CCDC25	TSS200	chr8:27631846-27632323	N_Shore
hyper1630	N_insufficient_4	5.2E-02	0.15	0.08	0.08	9	124498633	cg24015249	DAB2IP	Body	chr9:124498513-124498962	Island
hyper1631	N_insufficient_4	8.7E-04	0.08	0.01	0.07	10	82116365	cg11854806	DYDC2;DYDC1	TSS200;5'UTR	chr10:82116202-82117120	Island
hyper1632	N_insufficient_4	4.5E-02	0.18	0.07	0.12	10	82116392	cg16771783	DYDC2;DYDC1	TSS200;5'UTR	chr10:82116202-82117120	Island
hyper1633	N_insufficient_4	1.6E-04	0.11	0.02	0.10	11	32457158	cg19570244	WT1;WIT1;WT1;WT1;WT1	TSS200;TSS200;TSS200;TSS200	chr11:32454874-32457311	Island
hyper1634	N_insufficient_4	3.0E-03	0.32	0.11	0.21	11	112160734	cg05164022			chr11:112160815-112161201	N_Shore
hyper1635	N_insufficient_4	2.3E-03	0.34	0.04	0.31	11	112160850	cg09076521			chr11:112160815-112161201	Island
hyper1636	N_insufficient_4	2.6E-04	0.32	0.09	0.23	11	112161200	cg13064429			chr11:112160815-112161201	Island
hyper1637	N_insufficient_4	1.6E-02	0.18	0.05	0.13	12	26348437	cg02963114	SSPN;SSPN;SSPN	5'UTR;1stExon;TSS200	chr12:26348260-26349130	Island
hyper1638	N_insufficient_4	1.7E-05	0.13	0.04	0.09	12	32832894	cg06077899	DNM1L;DNM1L;DNM1L	Body;Body;Body	chr12:32832130-32832978	Island
hyper1639	N_insufficient_4	2.6E-02	0.06	0.02	0.04	13	100632250	cg18656829			chr13:100631752-100635526	Island
hyper1640	N_insufficient_4	0.0E+00	0.41	0.08	0.34	13	100740910	cg22856141	PCCA;PCCA	TSS1500;TSS1500	chr13:100740956-100741805	N_Shore
hyper1641	N_insufficient_4	0.0E+00	0.49	0.04	0.44	13	100740987	cg23446109	PCCA;PCCA	TSS1500;TSS1500	chr13:100740956-100741805	Island
hyper1642	N_insufficient_4	0.0E+00	0.43	0.05	0.38	13	100741026	cg23235217	PCCA;PCCA	TSS1500;TSS1500	chr13:100740956-100741805	Island
hyper1643	N_insufficient_4	0.0E+00	0.45	0.04	0.40	13	100741041	cg10877050	PCCA;PCCA	TSS1500;TSS1500	chr13:100740956-100741805	Island
hyper1644	N_insufficient_4	0.0E+00	0.55	0.01	0.54	13	100741161	cg16681130	PCCA;PCCA	TSS200;TSS200	chr13:100740956-100741805	Island
hyper1645	N_insufficient_4	0.0E+00	0.50	0.01	0.49	13	100741163	cg08608532	PCCA;PCCA	TSS200;TSS200	chr13:100740956-100741805	Island
hyper1646	N_insufficient_4	0.0E+00	0.52	0.02	0.50	13	100741287	cg00224563	PCCA;PCCA	TSS200;TSS200	chr13:100740956-100741805	Island
hyper1647	N_insufficient_4	1.9E-10	0.52	0.12	0.40	13	100741297	cg10883266	PCCA;PCCA	TSS200;TSS200	chr13:100740956-100741805	Island
hyper1648	N_insufficient_4	0.0E+00	0.63	0.03	0.59	13	100741364	cg00463171	PCCA;PCCA;PCCA;PCCA	1stExon;5'UTR;1stExon;5'UTR	chr13:100740956-100741805	Island
hyper1649	N_insufficient_4	0.0E+00	0.44	0.07	0.38	13	100741804	cg09754122	PCCA;PCCA	Body;Body	chr13:100740956-100741805	Island
hyper1650	N_insufficient_4	1.6E-05	0.48	0.15	0.32	13	100742055	cg10230711	PCCA;PCCA	Body;Body	chr13:100740956-100741805	S_Shore
hyper1651	N_insufficient_4	1.2E-07	0.18	0.06	0.12	15	90198226	cg22690294	KIF7	5'UTR	chr15:90198100-90198833	Island
hyper1652	N_insufficient_4	0.0E+00	0.17	0.01	0.16	17	1613572	cg19118797	TLCD2;TLCD2	5'UTR;1stExon	chr17:1613226-1614110	Island
hyper1653	N_insufficient_4	1.9E-10	0.13	0.01	0.11	17	1613581	cg25678929	TLCD2;TLCD2	5'UTR;1stExon	chr17:1613226-1614110	Island
hyper1654	N_insufficient_4	5.7E-05	0.12	0.04	0.08	17	1613851	cg13311509	TLCD2	TSS200	chr17:1613226-1614110	Island
hyper1655	N_insufficient_4	6.4E-04	0.24	0.06	0.18	17	77774880	cg26459419			chr17:77770672-77778468	Island
hyper1656	N_insufficient_5	1.3E-04	0.23	0.07	0.15	3	85007817	cg15355387	CADM2;CADM2	TSS1500;TSS1500	chr3:85008284-85008837	N_Shore
hyper1657	N_insufficient_5	5.1E-03	0.16	0.08	0.08	3	85007992	cg25649680	CADM2;CADM2	TSS200;TSS200	chr3:85008284-85008837	N_Shore
hyper1658	N_insufficient_5	6.0E-07	0.16	0.07	0.09	3	101231982	cg04223389	SENP7;SENP7;SENP7;SENP7	1stExon;1stExon;5'UTR;5'UTR	chr3:101231610-101232173	Island
hyper1659	N_insufficient_5	7.7E-02	0.18	0.07	0.11	3	143690495	cg07885039	C3orf58	TSS200	chr3:143689733-143692279	Island
hyper1660	N_insufficient_5	4.9E-02	0.20	0.11	0.09	5	126205009	cg23155911	41701	3'UTR		
hyper1661	N_insufficient_5	9.0E-02	0.14	0.06	0.08	5	126205045	cg25568066	41701	3'UTR		
hyper1662	N_insufficient_5	6.9E-05	0.24	0.03	0.22	5	126205081	cg06918474	41701	3'UTR		
hyper1663	N_insufficient_5	1.5E-04	0.23	0.06	0.17	6	7390716	cg02013957	CAGE1;RIOK1;CAGE1;CAGE1	TSS1500;Body;TSS1500;TSS150	chr6:7389634-7390317	S_Shore
hyper1664	N_insufficient_5	4.0E-05	0.25	0.06	0.19	6	30483837	cg22765426				
hyper1665	N_insufficient_5	6.1E-07	0.15	0.05	0.10	6	30483908	cg16013925				
hyper1666	N_insufficient_5	1.6E-04	0.13	0.05	0.08	6	30483923	cg22410026				
hyper1667	N_insufficient_5	0.0E+00	0.86	0.09	0.76	6	30690501	cg19748937	TUBB	Body	chr6:30688624-30689530	S_Shore
hyper1668	N_insufficient_5	7.1E-03	0.16	0.08	0.08	6	159125489	cg16700296	SYTL3	Body		
hyper1669	N_insufficient_5	1.5E-03	0.19	0.07	0.12	7	14029592	cg05605257	ETV1;ETV1;ETV1;ETV1;ETV1;ET	1stExon;5'UTR;5'UTR;5'UTR;1stE	chr7:14029378-14029593	Island
hyper1670	N_insufficient_5	6.6E-02	0.19	0.05	0.13	7	14029680	cg10386045	ETV1;ETV1;ETV1;ETV1	TSS200;5'UTR;5'UTR;TSS200	chr7:14030965-14031213	N_Shore
hyper1671	N_insufficient_5	8.8E-05	0.10	0.03	0.07	7	14030127	cg01958221	ETV1;ETV1;ETV1;ETV1	5'UTR;TSS1500;5'UTR;TSS1500	chr7:14030965-14031213	N_Shore
hyper1672	N_insufficient_5	1.4E-02	0.19	0.08	0.10	7	14030255	cg02881625	ETV1;ETV1;ETV1;ETV1	5'UTR;TSS1500;5'UTR;TSS1500	chr7:14030965-14031213	N_Shore

hyper1673	N_insufficient_5	4.8E-02	0.18	0.05	0.13	7	14030351	cg05918135	ETV1;ETV1;ETV1;ETV1	5'UTR;TSS1500;5'UTR;TSS1500	chr7:14030965-14031213	N_Shore
hyper1674	N_insufficient_5	2.4E-03	0.22	0.08	0.14	7	14030466	cg11897145	ETV1;ETV1;ETV1;ETV1	5'UTR;TSS1500;5'UTR;TSS1500	chr7:14030965-14031213	N_Shore
hyper1675	N_insufficient_5	5.8E-10	0.13	0.02	0.12	7	14030482	cg06561666	ETV1;ETV1;ETV1;ETV1	5'UTR;TSS1500;5'UTR;TSS1500	chr7:14030965-14031213	N_Shore
hyper1676	N_insufficient_5	1.1E-03	0.11	0.02	0.09	7	14030807	cg00328593	ETV1;ETV1;ETV1;ETV1;ETV1	5'UTR;1stExon;TSS1500;5'UTR;T	chr7:14030965-14031213	N_Shore
hyper1677	N_insufficient_5	8.9E-04	0.15	0.05	0.10	7	19155806	cg04043591	TWIST1	3'UTR	chr7:19156050-19158042	N_Shore
hyper1678	N_insufficient_5	0.0E+00	0.31	0.08	0.23	7	121512875	cg21423724	PTPRZ1	TSS1500	chr7:121513046-121513911	N_Shore
hyper1679	N_insufficient_5	0.0E+00	0.42	0.05	0.37	7	121513058	cg05931096	PTPRZ1	TSS200	chr7:121513046-121513911	Island
hyper1680	N_insufficient_5	0.0E+00	0.34	0.08	0.25	7	121513065	cg00826384	PTPRZ1	TSS200	chr7:121513046-121513911	Island
hyper1681	N_insufficient_5	0.0E+00	0.38	0.09	0.29	7	121513704	cg07757861	PTPRZ1	Body	chr7:121513046-121513911	Island
hyper1682	N_insufficient_5	0.0E+00	0.23	0.02	0.22	7	121513910	cg06631603	PTPRZ1	Body	chr7:121513046-121513911	Island
hyper1683	N_insufficient_5	1.1E-09	0.29	0.04	0.25	7	121513944	cg22410750	PTPRZ1	Body	chr7:121513046-121513911	S_Shore
hyper1684	N_insufficient_5	1.5E-09	0.22	0.05	0.17	8	23584098	cg19719133			chr8:23584001-23584761	Island
hyper1685	N_insufficient_5	9.7E-11	0.46	0.14	0.32	9	79792074	cg10975354	VPS13A;VPS13A;LOC100286938	TSS1500;TSS1500;Body;TSS150	chr9:79792070-79793328	Island
hyper1686	N_insufficient_5	0.0E+00	0.32	0.08	0.24	9	127703551	cg14452096	GOLGA1	TSS200	chr9:127703131-127703629	Island
hyper1687	N_insufficient_5	0.0E+00	0.31	0.06	0.26	9	127703617	cg09227136	GOLGA1	TSS1500	chr9:127703131-127703629	Island
hyper1688	N_insufficient_5	2.1E-03	0.49	0.17	0.32	12	39538854	cg20762480			chr12:39538383-39539500	Island
hyper1689	N_insufficient_5	0.0E+00	0.27	0.05	0.22	12	39539408	cg09788416			chr12:39538383-39539500	Island
hyper1690	N_insufficient_5	6.0E-09	0.27	0.08	0.19	12	39539422	cg03468072			chr12:39538383-39539500	Island
hyper1691	N_insufficient_5	2.4E-02	0.10	0.04	0.06	12	125534043	cg13938881			chr12:125534060-125534527	N_Shore
hyper1692	N_insufficient_5	5.4E-02	0.14	0.05	0.09	12	125534110	cg21265540			chr12:125534060-125534527	Island
hyper1693	N_insufficient_5	1.1E-06	0.20	0.08	0.12	12	125534526	cg15701313			chr12:125534060-125534527	Island
hyper1694	N_insufficient_5	4.7E-09	0.15	0.02	0.14	14	105116681	cg23656707			chr14:105116496-105116923	Island
hyper1695	N_insufficient_5	5.0E-07	0.37	0.13	0.23	15	51386254	cg06813578	TNFAIP8L3	Body	chr15:51385861-51387203	Island
hyper1696	N_insufficient_5	0.0E+00	0.31	0.10	0.21	15	51386452	cg17180386	TNFAIP8L3	Body	chr15:51385861-51387203	Island
hyper1697	N_insufficient_5	0.0E+00	0.41	0.04	0.37	15	51386759	cg07218022	TNFAIP8L3	Body	chr15:51385861-51387203	Island
hyper1698	N_insufficient_5	2.6E-07	0.29	0.03	0.26	15	51387571	cg20642189	TNFAIP8L3	Body	chr15:51387532-51387755	Island
hyper1699	N_insufficient_5	1.2E-02	0.03	0.01	0.03	16	12009700	cg01571407	GSPT1;GSPT1;GSPT1;GSPT1;G	1stExon;5'UTR;1stExon;5'UTR;5'U	chr16:12008880-12010114	Island
hyper1700	N_insufficient_5	6.5E-03	0.20	0.09	0.11	17	19015961	cg16919567			chr17:19014144-19014604	S_Shore
hyper1701	N_insufficient_5	6.1E-02	0.19	0.09	0.10	18	5542879	cg26790372	EPB41L3	5'UTR	chr18:5542847-5543054	Island
hyper1702	N_insufficient_5	1.2E-02	0.29	0.08	0.21	18	47088306	cg27631427	LIPG	TSS200	chr18:47087879-47088528	Island
hyper1703	N_insufficient_5	7.1E-04	0.27	0.07	0.20	18	47088309	cg21574400	LIPG	TSS200	chr18:47087879-47088528	Island
hyper1704	N_insufficient_5	1.1E-06	0.30	0.06	0.25	18	47088343	cg14320593	LIPG	TSS200	chr18:47087879-47088528	Island
hyper1705	N_insufficient_5	1.0E-05	0.22	0.06	0.16	18	47088373	cg00692763	LIPG	TSS200	chr18:47087879-47088528	Island
hyper1706	N_insufficient_5	9.7E-04	0.22	0.08	0.13	18	47088421	cg11038073	LIPG	TSS200	chr18:47087879-47088528	Island
hyper1707	N_insufficient_5	8.1E-04	0.23	0.08	0.16	18	47088432	cg14254867	LIPG;LIPG	5'UTR;1stExon	chr18:47087879-47088528	Island
hyper1708	N_insufficient_5	3.9E-10	0.25	0.04	0.20	19	9648995	cg25227274	ZNF426	5'UTR	chr19:9648934-9649410	Island
hyper1709	N_insufficient_5	1.9E-10	0.17	0.03	0.14	19	9649241	cg11845033	ZNF426	5'UTR	chr19:9648934-9649410	Island
hyper1710	N_insufficient_5	1.5E-06	0.28	0.10	0.18	19	9649376	cg21285525	ZNF426	TSS200	chr19:9648934-9649410	Island
hyper1711	N_adequate_1	1.1E-02	0.13	0.04	0.09	1	110453107	cg05005301	CSF1;CSF1;CSF1;CSF1	TSS200;TSS200;TSS200;TSS200	chr1:110453143-110453889	N_Shore
hyper1712	N_adequate_1	4.2E-02	0.13	0.07	0.06	2	211341126	cg20604456	LANCL1;LANCL1;CPS1;LANCL1	5'UTR;5'UTR;TSS1500;5'UTR	chr2:211341181-211341536	N_Shore
hyper1713	N_adequate_1	3.5E-03	0.08	0.01	0.07	2	211341199	cg15764953	LANCL1;LANCL1;CPS1;LANCL1	5'UTR;5'UTR;TSS1500;5'UTR	chr2:211341181-211341536	Island
hyper1714	N_adequate_1	7.2E-06	0.10	0.02	0.08	2	219861150	cg14508391			chr2:219860927-219861242	Island
hyper1715	N_adequate_1	0.0E+00	0.18	0.02	0.16	3	9957221	cg23350106	IL17RE;IL17RE;IL17RE	Body;Body;Body	chr3:9956822-9957664	Island
hyper1716	N_adequate_1	4.2E-03	0.13	0.06	0.07	3	9957531	cg05845178	IL17RE;IL17RC;IL17RE;IL17RE;IL	3'UTR;TSS1500;3'UTR;3'UTR;TS	chr3:9956822-9957664	Island
hyper1717	N_adequate_1	1.4E-09	0.25	0.08	0.17	3	154797083	cg07862554	MME;MME;MME;MME	TSS1500;TSS1500;TSS1500;TSS	chr3:154797398-154797988	N_Shore
hyper1718	N_adequate_1	8.8E-10	0.20	0.08	0.12	3	154797399	cg08085954	MME;MME;MME;MME	TSS200;TSS1500;TSS1500;TSS1	chr3:154797398-154797988	Island
hyper1719	N_adequate_1	0.0E+00	0.27	0.08	0.19	3	154797508	cg03648334	MME;MME;MME;MME;MME	TSS1500;1stExon;TSS200;TSS15	chr3:154797398-154797988	Island
hyper1720	N_adequate_1	0.0E+00	0.24	0.05	0.19	3	154797563	cg17344755	MME;MME;MME;MME	TSS1500;TSS200;TSS1500;5'UTI	chr3:154797398-154797988	Island
hyper1721	N_adequate_1	1.6E-09	0.27	0.09	0.18	3	154797589	cg16580737	MME;MME;MME;MME	TSS1500;TSS200;TSS1500;5'UTI	chr3:154797398-154797988	Island
hyper1722	N_adequate_1	0.0E+00	0.37	0.05	0.31	3	154797677	cg21496658	MME;MME;MME;MME	TSS1500;TSS200;TSS1500;5'UTI	chr3:154797398-154797988	Island
hyper1723	N_adequate_1	5.8E-10	0.36	0.09	0.26	3	154797779	cg22471230	MME;MME;MME;MME;MME	5'UTR;TSS1500;TSS200;1stExon;	chr3:154797398-154797988	Island
hyper1724	N_adequate_1	0.0E+00	0.29	0.02	0.27	3	154797867	cg02108850	MME;MME;MME;MME	5'UTR;TSS1500;TSS200;5'UTR	chr3:154797398-154797988	Island
hyper1725	N_adequate_1	0.0E+00	0.34	0.07	0.27	3	154797892	cg26872138	MME;MME;MME;MME	TSS200;5'UTR;TSS200;5'UTR	chr3:154797398-154797988	Island
hyper1726	N_adequate_1	0.0E+00	0.31	0.02	0.29	3	154797917	cg23209255	MME;MME;MME;MME	TSS200;5'UTR;TSS200;5'UTR	chr3:154797398-154797988	Island
hyper1727	N_adequate_1	0.0E+00	0.29	0.02	0.26	3	154797947	cg22955973	MME;MME;MME;MME	TSS200;5'UTR;TSS200;5'UTR	chr3:154797398-154797988	Island
hyper1728	N_adequate_1	0.0E+00	0.26	0.01	0.25	3	154797958	cg20216802	MME;MME;MME;MME;MME	TSS200;5'UTR;1stExon;5'UTR;5'U	chr3:154797398-154797988	Island
hyper1729	N_adequate_1	5.8E-10	0.24	0.07	0.17	3	154798079	cg05962239	MME;MME;MME;MME;MME;MME	5'UTR;5'UTR;1stExon;5'UTR;1stE	chr3:154797398-154797988	S_Shore
hyper1730	N_adequate_1	1.6E-03	0.14	0.08	0.07	4	56502116	cg01943185	NMU	Body	chr4:56501912-56502523	Island
hyper1731	N_adequate_1	0.0E+00	0.23	0.01	0.21	4	56502392	cg27191517	NMU;NMU	1stExon;5'UTR	chr4:56501912-56502523	Island
hyper1732	N_adequate_1	3.1E-08	0.25	0.09	0.16	4	56502469	cg26536813	NMU	TSS200	chr4:56501912-56502523	Island
hyper1733	N_adequate_1	1.4E-03	0.09	0.04	0.05	4	56502664	cg04427852	NMU	TSS200	chr4:56501912-56502523	S_Shore
hyper1734	N_adequate_1	1.4E-02	0.16	0.05	0.10	10	8374293	cg08860607				
hyper1735	N_adequate_1	6.9E-04	0.29	0.11	0.18	10	44880236	cg25721625	CXCL12;CXCL12;CXCL12	Body;Body;Body	chr10:44879714-44882391	Island
hyper1736	N_adequate_1	0.0E+00	0.17	0.01	0.16	10	44880542	cg06048524	CXCL12;CXCL12;CXCL12	TSS200;TSS200;TSS200	chr10:44879714-44882391	Island
hyper1737	N_adequate_1	0.0E+00	0.22	0.02	0.20	10	44880545	cg17267805	CXCL12;CXCL12;CXCL12	TSS200;TSS200;TSS200	chr10:44879714-44882391	Island
hyper1738	N_adequate_1	2.3E-03	0.03	0.00	0.03	10	44880559	cg23407507	CXCL12;CXCL12;CXCL12	TSS200;TSS200;TSS200	chr10:44879714-44882391	Island
hyper1739	N_adequate_1	0.0E+00	0.11	0.01	0.10	10	45496482	cg19188595	C10orf25;ZNF22	TSS200;5'UTR	chr10:45496275-45496550	Island
hyper1740	N_adequate_1	1.6E-02	0.07	0.01	0.06	10	45496672	cg14260521	ZNF22;C10orf25	5'UTR;TSS1500	chr10:45496275-45496550	S_Shore
hyper1741	N_adequate_1	2.2E-02	0.76	0.55	0.21	11	2722407	cg26963277	KCNQ1OT1;KCNQ1;KCNQ1	TSS1500;Body;Body	chr11:2720410-2722087	S_Shore
hyper1742	N_adequate_1	6.9E-04	0.16	0.02	0.14	15	82555113	cg13512069	EFTUD1;EFTUD1;FAM154B	TSS200;TSS200;TSS200	chr15:82554785-82555476	Island
hyper1743	N_adequate_1	5.5E-02	0.19	0.08	0.11	15	84749130	cg16464483			chr15:84748593-84749285	Island
hyper1744	N_adequate_1	2.9E-03	0.17	0.07	0.10	16	74565916	ch.16.1684049R	GLG1;GLG1;GLG1;GLG1;GLG1	Body;Body;Body;Body;Body		
hyper1745	N_adequate_1	4.8E-08	0.14	0.03	0.11	17	16310359	cg03781505			chr17:16310306-16310914	Island
hyper1746	N_adequate_1	4.4E-03	0.26	0.13	0.13	17	42635310	cg25228126	FZD2	1stExon	chr17:42634829-42636851	Island
hyper1747	N_adequate_1	1.5E-04	0.13	0.02	0.11	17	46659318	cg24672833			chr17:46658948-46660003	Island
hyper1748	N_adequate_1	7.2E-03	0.78	0.63	0.15	20	57427146	cg24617313	GNAS;GNAS;GNAS;GNASAS	TSS1500;TSS1500;3'UTR;TSS15	chr20:57427691-57427995	N_Shore
hyper1749	N_adequate_1	3.9E-10	0.34	0.06	0.28	22	33197638	cg20500237	SYN3;TIMP3;TIMP3;SYN3;SYN3	Body;1stExon;5'UTR;Body;Body	chr22:33197380-33198267	Island
hyper1750	N_adequate_1	0.0E+00	0.31	0.07	0.24	22	33198055	cg05288803	SYN3;TIMP3;SYN3;SYN3	Body;1stExon;Body;Body	chr22:33197380-33198267	Island
hyper1751	N_adequate_2	1.3E-03	0.09	0.02	0.06	2	101435167	cg00581731	NPAS2	TSS1500	chr2:101434979-101437453	Island
hyper1752	N_adequate_2	1.5E-02	0.39	0.10	0.30	2	157292113	cg22033586	GPD2;GPD2;GPD2	1stExon;TSS1500;5'UTR	chr2:157292102-157292646	Island
hyper1753	N_adequate_2	4.3E-06	0.41	0.04	0.37	2	157292411	cg09770905	GPD2;GPD2	TSS1500;5'UTR	chr2:157292102-157292646	Island
hyper1754	N_adequate_2	1.4E-03	0.09	0.02	0.07	2	157292608	cg21233754	GPD2;GPD2	TSS1500;5'UTR	chr2:157292102-157292646	Island
hyper1755	N_adequate_2	2.5E-02	0.10	0.05	0.05	4	91048916	cg01518219	FAM190A;FAM190A	5'UTR;1stExon	chr4:91048411-91049682	Island
hyper1756	N_adequate_2	9.0E-05	0.06	0.01	0.05	4	121843785	cg13155001	PRDM5;PRDM5	1stExon;5'UTR	chr4:121843148-121844193	Island
hyper1757	N_adequate_2	4.6E-02	0.15	0.07	0.08	5	1931544	cg13143349			chr5:1930754-1931986	Island
hyper1758	N_adequate_2	6.9E-02	0.32	0.15	0.17	5	32709859	cg22697364			chr5:32709910-32714450	N_Shore
hyper1759	N_adequate_2	1.2E-04	0.07	0.01	0.06	5	119799864	cg27107171	PRR16	TSS200	chr5:119799821-119800148	Island
hyper1760	N_adequate_2	0.0E+00	0.33	0.05	0.29	8	65711067	cg12755298	CYP7B1	1stExon	chr8:65710990-65711722	Island
hyper1761	N_adequate_2	1.6E-08	0.19	0.05	0.14	8	65711327	cg04418492	CYP7B1;CYP7B1	1stExon;5'UTR	chr8:65710990-65711722	Island
hyper1762	N_adequate_2	5.6E-07	0.15	0.03	0.12	8	65711369	cg15160198	CYP7B1	TSS200	chr8:65710990-65711722	Island
hyper1763	N_adequate_2	7.4E-05	0.26	0.08	0.18	8	85094904	cg01134282	RALYL;RALYL;RALYL	TSS1500;TSS1500;TSS1500	chr8:85096759-85097247	N_Shore
hyper1764	N_adequate_2	9.7E-11	0.13	0.02	0.10	8	122654045	cg04719491	HAS2;HAS2AS	TSS1500;Body	chr8:122651665-122652389	S_Shore
hyper1765	N_adequate_2	8.8E-10	0.31	0.08	0.23	10	43134234	cg13590978	ZNF33B	TSS1500	chr10:43133523-43134150	S_Shore
hyper1766	N_adequate_2	5.2E-02	0.36	0.09	0.27	10	64134155	cg15591678	ZNF365;ZNF365;ZNF365;ZNF365	5'UTR;5'UTR;1stExon;1stExon;1st	chr10:64133789-64134420	Island
hyper1767	N_adequate_2	9.6E-03	0.09	0.02	0.07	11	34460557	cg01847719	CAT;CAT	1stExon;5'UTR	chr11:34460165-34460857	Island
hyper1768	N_adequate_2	2.2E-03	0.29	0.12	0.17	12	7592993	cg11296759	CD163L1	Body	chr12:7592406-7593030	Island
hyper1769	N_adequate_2	7.0E-03	0.14	0.06	0.08	13	20702278	cg21271681			chr13:20701817-20702634	Island
hyper1770	N_adequate_2	4.5E-04	0.22	0.07	0.14	13	20702426	cg25296860			chr13:20701817-20702634	Island
hyper1771	N_adequate_2	4.6E-03	0.19	0.06	0.13	13	28366759	cg10071824	GSX1	TSS200	chr13:28366549-28368505	Island
hyper1772	N_adequate_2	1.0E-05	0.19	0.07	0.12	15	68177265	cg18760920			chr15:68177160-68177415	Island
hyper1773	N_adequate_2	9.1E-06	0.12	0.04	0.09	15	68177334	cg19925445			chr15:68177160-68177415	Island
hyper1774	N_adequate_2	1.3E-07	0.15	0.03	0.12	15	74658244	cg02916102	CYP11A1;CYP11A1;CYP11A1	1stExon;Body;5'UTR	chr15:74658038-74658574	Island
hyper1775	N_adequate_2	6.0E-06	0.36	0.06	0.30	15	100882213	cg20578175	ADAMTS17	TSS200	chr15:100880957-100882438	Island
hyper1776	N_adequate_2	3.1E-02	0.15	0.08	0.07	17	1012967	cg15369512	ABR;ABR;ABR	Body;TSS1500;Body	chr17:1011844-1012968	Island
hyper1777	N_adequate_2	5.9E-02	0.34	0.09	0.25	19	7460276	cg09353354	ARHGEF18	5'UTR	chr19:7459779-7460376	Island
hyper1778	N_adequate_2	5.9E-07	0.29	0.06	0.23	19	7460327	cg08356214	ARHGEF18	5'UTR	chr19:7459779-7460376	Island
hyper1779	N_adequate_2	1.8E-09	0.12	0.02	0.10	19	38747079	cg07595291	PPP1R14A;PPP1R14A	5'UTR;1stExon	chr19:38746638-38747379	Island
hyper1780	N_adequate_2	7.2E-08	0.32	0.02	0.30	19	38747174	cg20232102	PPP1R14A	TSS200	chr19:38746638-38747379	Island
hyper1781	N_adequate_2	0.0E+00	0.31	0.02	0.29	19	38747201	cg13564825	PPP1R14A	TSS200	chr19:38746638-38747379	Island
hyper1782	N_adequate_2	1.3E-02	0.23	0.09	0.13	19	38747285	cg14131526	PPP1R14A	TSS200	chr19:38746638-38747379	Island
hyper1783	N_adequate_2	6.5E-04	0.21	0.07	0.14	19	38747292	cg22730305	PPP1R14A	TSS200	chr19:38746638-38747379	Island
hyper1784	N_adequate_2	1.9E-10	0.30	0.02	0.28	19	38747355	cg09515953	PPP1R14A	TSS200	chr19:38746638-38747379	Island
hyper1785	N_adequate_2	0.0E+00	0.27	0.01	0.26	19	38747378	cg02571816	PPP1R14A	TSS1500	chr19:38746638-38747379	Island
hyper1786	N_adequate_2	1.0E-03	0.43	0.04	0.39	19	41018876	cg17768491	SPTBN4	Body	chr19:41018525-41019486	Island
hyper1787	N_adequate_2	2.6E-08	0.21	0.07	0.15	19	45147715	cg01496416	PVR;PVR;PVR;PVR	Body;Body;Body;Body	chr19:45147074-45147721	Island
hyper1788	N_adequate_2	2.2E-02	0.26	0.12	0.13	20	7917212	cg00407901	HAO1	Body		
hyper1789	N_adequate_2	3.4E-05	0.20	0.05	0.15	22	50919817	cg06367659	ADM2	TSS200	chr22:50919453-50920376	Island
hyper1790	N_adequate_2	1.3E-07	0.19	0.02	0.18	22	50919823	cg14630839	ADM2	TSS200	chr22:50919453-50920376	Island
hyper1791	N_adequate_3	1.1E-02	0.05	0.01	0.04	2	177005403	cg02877791			chr2:177005364-177005666	Island
hyper1792	N_adequate_3	2.8E-02	0.19	0.11	0.08	2	177005719	cg17278528			chr2:177004452-177004693	S_Shore
hyper1793	N_adequate_3	6.0E-04	0.23	0.09	0.15	2	219757464	cg23937893	WNT10A	Body	chr2:219757458-219758001	Island
hyper1794	N_adequate_3	0.0E+00	0.25	0.02	0.22	2	219757724	cg26146971	WNT10A	Body	chr2:219757458-219758001	Island
hyper1795	N_adequate_3	1.2E-04	0.24	0.06	0.18	3	115502895	cg11826708			chr3:115502725-115503581	Island
hyper1796	N_adequate_3	9.7E-03	0.27	0.09	0.17	3	115503319	cg26044026			chr3:115502725-115503581	Island
hyper1797	N_adequate_3	2.6E-04	0.04	0.01	0.03	3	119396027	cg24850932	COX17	Body	chr3:119395989-119396387	Island
hyper1798	N_adequate_3	4.3E-02	0.26	0.08	0.18	4	156129735	cg03202738	NPY2R	TSS200	chr4:156129168-156130209	Island
hyper1799	N_adequate_3	1.2E-08	0.26	0.07	0.19	7	63767753	cg09382800			chr7:63767750-63767991	Island
hyper1800	N_adequate_3	6.3E-06	0.24	0.02	0.22	7	63767841	cg23849078			chr7:63767750-63767991	Island
hyper1801	N_adequate_3	1.9E-02	0.09	0.03	0.06	7	64363934	cg00511228	ZNF273;ZNF273	Body;Body		

hyper1802	N_adequate_3	7.4E-05	0.27	0.06	0.21	7	64466808	cg14059817				
hyper1803	N_adequate_3	9.3E-04	0.35	0.06	0.29	7	64466846	cg04321566				
hyper1804	N_adequate_3	1.1E-04	0.30	0.09	0.21	7	64467056	cg26268843				
hyper1805	N_adequate_3	2.4E-03	0.07	0.03	0.04	8	22722817	cg10611863	PEBP4	Body	chr8:22722313-22723552	Island
hyper1806	N_adequate_3	4.6E-05	0.05	0.01	0.04	10	99393498	cg06124827	MORN4;MORN4	TSS200;5'UTR	chr10:99392786-99393375	S_Shore
hyper1807	N_adequate_3	1.3E-02	0.32	0.13	0.19	11	75917251	cg20633317	WNT11	Body	chr11:75917271-75917926	N_Shore
hyper1808	N_adequate_3	0.0E+00	0.29	0.06	0.23	15	95388002	cg26237512			chr15:95388001-95388438	Island
hyper1809	N_adequate_3	0.0E+00	0.32	0.10	0.23	19	24097753	cg20649823				
hyper1810	N_adequate_3	0.0E+00	0.71	0.14	0.57	19	45909864	cg23964739	CD3EAP;CD3EAP;PPP1R13L	5'UTR;1stExon;TSS1500	chr19:45909062-45910124	Island
hyper1811	N_adequate_3	1.5E-04	0.27	0.13	0.14	19	52872916	cg22080027	ZNF880	TSS1500	chr19:52873084-52873572	N_Shore
hyper1812	N_adequate_3	3.3E-08	0.12	0.02	0.11	19	56015381	cg27308346	SSC5D	Body	chr19:56014811-56015333	Island
hyper1813	N_adequate_3	4.0E-03	0.10	0.02	0.08	20	61051438	cg18278265	GATA5	TSS1500	chr20:61049361-61051897	Island
hyper1814	N_adequate_4	1.2E-02	0.27	0.09	0.19	1	55462126	cg18384681			chr1:55462688-55463010	N_Shore
hyper1815	N_adequate_4	1.1E-06	0.28	0.04	0.24	1	55462274	cg02392553			chr1:55462688-55463010	N_Shore
hyper1816	N_adequate_4	0.0E+00	0.29	0.07	0.22	1	155231827	cg02768471	SCAMP3;SCAMP3	Body;Body	chr1:155231812-155232312	Island
hyper1817	N_adequate_4	3.2E-02	0.11	0.04	0.07	1	155232227	cg18279150	SCAMP3;SCAMP3	TSS200;TSS200	chr1:155231812-155232312	Island
hyper1818	N_adequate_4	2.9E-08	0.26	0.06	0.19	1	155232251	cg20795117	SCAMP3;SCAMP3	TSS200;TSS200	chr1:155231812-155232312	Island
hyper1819	N_adequate_4	3.4E-05	0.18	0.05	0.13	1	155232344	cg24034289	SCAMP3;SCAMP3	TSS200;TSS200	chr1:155231812-155232312	S_Shore
hyper1820	N_adequate_4	1.6E-08	0.18	0.06	0.12	1	155232379	cg01745448	SCAMP3;SCAMP3	TSS200;TSS200	chr1:155231812-155232312	S_Shore
hyper1821	N_adequate_4	1.9E-02	0.30	0.09	0.21	2	45029285	cg19893751			chr2:45028955-45029318	Island
hyper1822	N_adequate_4	3.0E-05	0.22	0.07	0.15	2	45029729	cg13732857			chr2:45029728-45030035	Island
hyper1823	N_adequate_4	6.4E-05	0.32	0.06	0.26	4	124342	cg12717203	ZNF718	Body	chr4:124332-124841	Island
hyper1824	N_adequate_4	2.4E-02	0.19	0.04	0.16	4	125112	cg10197305	ZNF718	Body	chr4:124332-124841	S_Shore
hyper1825	N_adequate_4	2.1E-02	0.17	0.09	0.08	4	467047	cg05856015	ZNF721;ABCA11P	5'UTR;Body	chr4:467471-467839	N_Shore
hyper1826	N_adequate_4	3.1E-02	0.28	0.04	0.24	4	3464874	cg25237365	DOK7;DOK7	TSS200;TSS200	chr4:3464799-3465440	Island
hyper1827	N_adequate_4	1.5E-02	0.26	0.04	0.23	4	3464887	cg23757738	DOK7;DOK7	TSS200;TSS200	chr4:3464799-3465440	Island
hyper1828	N_adequate_4	3.6E-07	0.36	0.04	0.31	4	3464996	cg21733154	DOK7;DOK7	TSS200;TSS200	chr4:3464799-3465440	Island
hyper1829	N_adequate_4	3.8E-03	0.33	0.10	0.23	4	3464998	cg02062230	DOK7;DOK7	TSS200;TSS200	chr4:3464799-3465440	Island
hyper1830	N_adequate_4	4.6E-02	0.30	0.08	0.22	4	3465004	cg17960516	DOK7;DOK7	TSS200;TSS200	chr4:3464799-3465440	Island
hyper1831	N_adequate_4	4.2E-06	0.40	0.04	0.36	5	169064451	cg08862890	DOCK2	Body	chr5:169064270-169064702	Island
hyper1832	N_adequate_4	1.0E-03	0.17	0.02	0.15	7	91808500	cg17063962			chr7:91808331-91808632	Island
hyper1833	N_adequate_4	0.0E+00	0.15	0.00	0.14	8	143751204	cg10279794	JRK;JRK	5'UTR;5'UTR	chr8:143750759-143751448	Island
hyper1834	N_adequate_4	1.2E-06	0.09	0.02	0.07	8	143751325	cg26325135	JRK;JRK	5'UTR;5'UTR	chr8:143750759-143751448	Island
hyper1835	N_adequate_4	3.6E-02	0.42	0.04	0.38	10	75531995	cg17426063	FUT11	TSS200	chr10:75531970-75532817	Island
hyper1836	N_adequate_4	7.4E-02	0.43	0.06	0.37	10	75532169	cg23588893	FUT11	1stExon	chr10:75531970-75532817	Island
hyper1837	N_adequate_4	2.7E-02	0.45	0.09	0.36	10	75533025	cg12419678	FUT11	Body	chr10:75531970-75532817	S_Shore
hyper1838	N_adequate_4	2.4E-03	0.22	0.05	0.17	10	75533400	cg15838173	FUT11	Body	chr10:75531970-75532817	S_Shore
hyper1839	N_adequate_4	1.7E-08	0.20	0.01	0.19	10	98480463	cg12382864	PIK3AP1	TSS200	chr10:98479725-98480487	Island
hyper1840	N_adequate_4	1.1E-03	0.32	0.11	0.21	10	98480465	cg24478788	PIK3AP1	TSS200	chr10:98479725-98480487	Island
hyper1841	N_adequate_4	3.3E-03	0.26	0.08	0.18	10	98480474	cg07316170	PIK3AP1	TSS200	chr10:98479725-98480487	Island
hyper1842	N_adequate_4	2.5E-07	0.22	0.06	0.16	11	60673866	cg07588442	PRPF19;PRPF19	1stExon;5'UTR	chr11:60673597-60674184	Island
hyper1843	N_adequate_4	4.9E-10	0.50	0.09	0.42	12	6420034	cg20667825	PLEKHG6;PLEKHG6	5'UTR;TSS200	chr12:6419603-6420024	S_Shore
hyper1844	N_adequate_4	1.6E-06	0.15	0.06	0.09	14	23938784	cg05833251	NGDN;NGDN	TSS200;TSS200		
hyper1845	N_adequate_4	0.0E+00	0.24	0.07	0.16	14	23938824	cg05873158	NGDN;NGDN	TSS200;TSS200		
hyper1846	N_adequate_4	0.0E+00	0.28	0.07	0.21	14	23938846	cg26122402	NGDN;NGDN	TSS200;TSS200		
hyper1847	N_adequate_4	2.1E-03	0.17	0.03	0.14	14	23938929	cg05200925	NGDN;NGDN	1stExon;1stExon		
hyper1848	N_adequate_4	1.6E-05	0.16	0.05	0.11	14	23938996	cg04960285	NGDN;NGDN	Body;Body		
hyper1849	N_adequate_4	2.9E-06	0.16	0.05	0.11	14	23939157	cg10235955	NGDN;NGDN	Body;Body		
hyper1850	N_adequate_4	4.1E-02	0.21	0.09	0.12	16	5121709	cg00547018	ALG1	TSS200	chr16:5121749-5122192	N_Shore
hyper1851	N_adequate_4	5.8E-02	0.35	0.11	0.24	18	67067721	cg05588496	DOK6	TSS1500	chr18:67067509-67069168	Island
hyper1852	N_adequate_4	5.7E-02	0.44	0.08	0.36	18	67067867	cg27347269	DOK6	TSS1500	chr18:67067509-67069168	Island
hyper1853	N_adequate_4	1.7E-03	0.13	0.04	0.09	19	40950431	cg14150973	SERTAD3	TSS200	chr19:40950025-40950777	Island
hyper1854	N_adequate_4	7.4E-05	0.34	0.10	0.24	19	49925720	cg08567644	PTH2	3'UTR	chr19:49925626-49926012	Island
hyper1855	N_adequate_4	2.1E-05	0.31	0.10	0.20	19	49925731	cg05248581	PTH2	Body	chr19:49925626-49926012	Island
hyper1856	N_adequate_4	2.6E-02	0.31	0.18	0.13	19	49925920	cg11315979	PTH2	Body	chr19:49925626-49926012	Island
hyper1857	N_adequate_5	3.2E-05	0.14	0.02	0.12	1	49242757	cg03603214	AGBL4;BEND5	Body;TSS1500	chr1:49242371-49242810	Island
hyper1858	N_adequate_5	0.0E+00	0.24	0.03	0.20	1	184006285	cg05777716	GLT25D2	1stExon	chr1:184005359-184006766	Island
hyper1859	N_adequate_5	0.0E+00	0.30	0.04	0.26	1	184006496	cg15359501	GLT25D2;GLT25D2	5'UTR;1stExon	chr1:184005359-184006766	Island
hyper1860	N_adequate_5	3.2E-02	0.22	0.10	0.12	1	184006765	cg18073970	GLT25D2;GLT25D2	5'UTR;1stExon	chr1:184005359-184006766	Island
hyper1861	N_adequate_5	2.2E-03	0.13	0.06	0.08	1	223302861	cg16585333	TLR5	5'UTR		
hyper1862	N_adequate_5	5.5E-03	0.10	0.03	0.07	3	160822545	cg12349858	B3GALNT1;B3GALNT1;B3GALNT	5'UTR;5'UTR;5'UTR;5'UTR;5'UTR	chr3:160822494-160823260	Island
hyper1863	N_adequate_5	5.2E-04	0.19	0.11	0.09	3	160822661	cg23252387	B3GALNT1;B3GALNT1;B3GALNT	5'UTR;5'UTR;5'UTR;1stExon;5'UT	chr3:160822494-160823260	Island
hyper1864	N_adequate_5	7.8E-10	0.19	0.09	0.11	3	160822711	cg25469234	B3GALNT1;B3GALNT1;B3GALNT	5'UTR;5'UTR;5'UTR;TSS200;5'UT	chr3:160822494-160823260	Island
hyper1865	N_adequate_5	5.7E-09	0.18	0.05	0.13	3	160822728	cg16496687	B3GALNT1;B3GALNT1;B3GALNT	5'UTR;5'UTR;5'UTR;TSS200;5'UT	chr3:160822494-160823260	Island
hyper1866	N_adequate_5	0.0E+00	0.19	0.02	0.18	3	160822911	cg20841906	B3GALNT1;B3GALNT1;B3GALNT	5'UTR;5'UTR;5'UTR;5'UTR;TSS15	chr3:160822494-160823260	Island
hyper1867	N_adequate_5	2.2E-03	0.04	0.00	0.04	4	57976561	cg01959562	IGFBP7	TSS200	chr4:57975860-57976916	Island
hyper1868	N_adequate_5	1.4E-04	0.04	0.01	0.04	4	57976573	cg03357180	IGFBP7	TSS200	chr4:57975860-57976916	Island
hyper1869	N_adequate_5	3.7E-03	0.06	0.01	0.05	5	167956394	cg14743594	FBLL1	TSS200	chr5:167956219-167957371	Island
hyper1870	N_adequate_5	4.7E-04	0.20	0.05	0.15	6	116601073	cg15056247	DSE;TSPYL1;TSPYL1	TSS1500;1stExon;5'UTR	chr6:116600494-116601174	Island
hyper1871	N_adequate_5	3.2E-05	0.18	0.06	0.12	8	95732141	cg14329673	DPY19L4;DPY19L4	1stExon;5'UTR	chr8:95731798-95732449	Island
hyper1872	N_adequate_5	5.8E-02	0.11	0.07	0.05	8	146126807	cg04151839	ZNF250;ZNF250;ZNF250;ZNF250	5'UTR;1stExon;1stExon;5'UTR	chr8:146126088-146128055	Island
hyper1873	N_adequate_5	5.2E-05	0.04	0.01	0.03	8	146126815	cg13592003	ZNF250;ZNF250;ZNF250;ZNF250	5'UTR;1stExon;1stExon;5'UTR	chr8:146126088-146128055	Island
hyper1874	N_adequate_5	1.9E-10	0.12	0.01	0.11	11	8289974	cg12740527			chr11:8289532-8290322	Island
hyper1875	N_adequate_5	3.6E-05	0.05	0.01	0.04	11	8290308	cg20555682			chr11:8289532-8290322	Island
hyper1876	N_adequate_5	1.7E-08	0.29	0.05	0.24	11	79151611	cg06892009	ODZ4;ODZ4	5'UTR;1stExon	chr11:79148358-79152200	Island
hyper1877	N_adequate_5	5.5E-02	0.21	0.03	0.18	11	79151719	cg04983516	ODZ4	TSS200	chr11:79148358-79152200	Island
hyper1878	N_adequate_5	4.8E-02	0.37	0.21	0.16	11	79151791	cg05218311	ODZ4	TSS200	chr11:79148358-79152200	Island
hyper1879	N_adequate_5	3.3E-06	0.20	0.08	0.13	11	79151811	cg09673208	ODZ4	TSS200	chr11:79148358-79152200	Island
hyper1880	N_adequate_5	9.8E-05	0.16	0.06	0.10	11	79151814	cg05099909	ODZ4	TSS200	chr11:79148358-79152200	Island
hyper1881	N_adequate_5	2.1E-07	0.12	0.03	0.08	11	82444798	cg09617579	FAM181B;FAM181B	1stExon;5'UTR	chr11:82443435-82444976	Island
hyper1882	N_adequate_5	1.9E-04	0.12	0.03	0.09	12	26348331	cg03206503	SSPN;SSPN;SSPN	5'UTR;1stExon;TSS200	chr12:26348260-26349130	Island
hyper1883	N_adequate_5	8.5E-03	0.11	0.02	0.09	12	26348333	cg07725355	SSPN;SSPN;SSPN	5'UTR;1stExon;TSS200	chr12:26348260-26349130	Island
hyper1884	N_adequate_5	1.8E-06	0.15	0.04	0.11	12	123849313	cg09174610			chr12:123848617-123849919	Island
hyper1885	N_adequate_5	1.6E-04	0.15	0.03	0.12	13	25321731	cg04901121			chr13:25319668-25322255	Island
hyper1886	N_adequate_5	3.9E-04	0.22	0.07	0.16	16	70284893	cg11996632	EXOSC6;EXOSC6	3'UTR;1stExon	chr16:70284892-70285956	Island
hyper1887	N_adequate_5	8.1E-07	0.34	0.04	0.31	16	70284928	cg02727363	EXOSC6;EXOSC6	3'UTR;1stExon	chr16:70284892-70285956	Island
hyper1888	N_adequate_5	6.2E-05	0.18	0.05	0.12	16	70285408	cg27468401	EXOSC6	1stExon	chr16:70284892-70285956	Island
hyper1889	N_adequate_5	1.9E-10	0.23	0.02	0.21	16	70285806	cg09594067	EXOSC6;EXOSC6	5'UTR;1stExon	chr16:70284892-70285956	Island
hyper1890	N_adequate_5	3.1E-08	0.28	0.03	0.25	16	70285816	cg08164529	EXOSC6;EXOSC6	5'UTR;1stExon	chr16:70284892-70285956	Island
hyper1891	N_adequate_5	6.2E-09	0.26	0.06	0.19	16	70285955	cg14357669	EXOSC6	TSS200	chr16:70284892-70285956	Island
hyper1892	N_adequate_5	5.7E-03	0.14	0.07	0.07	17	12927897	cg13723217				
hyper1893	N_adequate_5	3.6E-04	0.21	0.03	0.18	19	52408259	cg19864007	ZNF649	TSS200		
hyper1894	N_adequate_5	1.0E-04	0.32	0.10	0.22	19	52408322	cg06728974	ZNF649	TSS200		
hyper1895	N_adequate_5	1.2E-05	0.31	0.08	0.23	19	52408324	cg16619071	ZNF649	TSS200		
hyper1896	N_adequate_5	3.8E-02	0.29	0.08	0.21	19	52408336	cg04425820	ZNF649	TSS200		
hyper1897	N_adequate_5	5.9E-02	0.21	0.06	0.15	19	52408348	cg22636429	ZNF649	TSS200		
hyper1898	N_adequate_5	1.2E-05	0.20	0.05	0.15	19	52408350	cg01930621	ZNF649	TSS200		
hyper1899	N_adequate_6	3.9E-10	0.32	0.08	0.24	1	2574349	cg26398836			chr1:2574030-2574864	Island
hyper1900	N_adequate_6	8.2E-04	0.34	0.08	0.26	1	2574549	cg10117795			chr1:2574030-2574864	Island
hyper1901	N_adequate_6	2.1E-03	0.21	0.06	0.15	1	2574695	cg01030973			chr1:2574030-2574864	Island
hyper1902	N_adequate_6	4.0E-02	0.19	0.10	0.10	2	240323809	cg08987387	HDAC4	TSS1500	chr2:240321777-240323919	Island
hyper1903	N_adequate_6	8.1E-03	0.17	0.06	0.12	6	84562778	cg03818920	RIPPLY2	TSS1500	chr6:84562820-84563758	N_Shore
hyper1904	N_adequate_6	1.9E-10	0.38	0.14	0.24	12	3862473	cg03485694	EFCAB4B;EFCAB4B;EFCAB4B	TSS200;TSS200;TSS200	chr12:3862068-3862606	Island
hyper1905	N_adequate_6	0.0E+00	0.30	0.08	0.22	12	50339482	cg08872738				
hyper1906	N_adequate_6	1.5E-06	0.14	0.03	0.11	12	50339529	cg11704528				
hyper1907	N_adequate_6	0.0E+00	0.25	0.05	0.20	13	112849476	cg05059349			chr13:112848575-112849221	S_Shore
hyper1908	N_adequate_6	4.1E-07	0.26	0.11	0.15	15	95870054	cg04878701			chr15:95869955-95870322	Island
hyper1909	N_adequate_6	1.8E-09	0.35	0.11	0.24	15	95870112	cg07057579			chr15:95869955-95870322	Island
hyper1910	N_adequate_6	4.8E-09	0.19	0.07	0.12	15	95870321	cg26056703			chr15:95869955-95870322	Island
hyper1911	N_adequate_6	0.0E+00	0.29	0.06	0.24	17	79615824	cg07073561			chr17:79614850-79615559	S_Shore
hyper1912	N_adequate_6	9.8E-03	0.11	0.04	0.07	19	551401	cg06423211			chr19:551290-551542	Island
hyper1913	N_adequate_6	1.0E-02	0.15	0.07	0.09	19	2169160	cg27073066	DOT1L	Body	chr19:2163632-2165603	S_Shelf
hyper1914	N_adequate_6	0.0E+00	0.37	0.09	0.29	19	56165307	cg24793625	U2AF2;U2AF2	TSS200;TSS200	chr19:56163662-56167257	Island
hyper1915	N_adequate_6	0.0E+00	0.18	0.02	0.16	20	278758	cg05792285	ZCCHC3	1stExon	chr20:277831-279155	Island
hyper1916	N_adequate_6	6.1E-03	0.30	0.05	0.26	20	53092334	cg20107395	DOK5;DOK5	1stExon;5'UTR	chr20:53091733-53092993	Island
hyper1917	N_adequate_7	3.5E-04	0.15	0.04	0.11	1	968395	cg06712559	AGRN	Body	chr1:967966-970238	Island
hyper1918	N_adequate_7	2.5E-08	0.08	0.01	0.06	1	40254859	cg13458335	BMP8B	TSS1500	chr1:40253683-40255172	Island
hyper1919	N_adequate_7	8.3E-07	0.21	0.10	0.10	1	183386237	cg08244866	NMNAT2	Body	chr1:183386000-183387664	Island
hyper1920	N_adequate_7	8.8E-02	0.29	0.05	0.23	2	75787718	cg07724816	FAM176A;FAM176A	5'UTR;5'UTR	chr2:75787717-75788312	Island
hyper1921	N_adequate_7	1.8E-03	0.43	0.07	0.36	2	75788423	cg00156336	FAM176A;FAM176A	5'UTR;TSS1500	chr2:75787129-75787420	S_Shore
hyper1922	N_adequate_7	4.6E-03	0.35	0.15	0.20	2	75788552	cg16331051	FAM176A;FAM176A	5'UTR;TSS1500	chr2:75787129-75787420	S_Shore
hyper1923	N_adequate_7	5.1E-09	0.30	0.01	0.29	2	99552737	cg20852890	C2orf55	TSS200	chr2:99552185-99553831	Island
hyper1924	N_adequate_7	8.7E-04	0.11	0.03	0.07	2	99552827	cg16721459	C2orf55	TSS200	chr2:99552185-99553831	Island
hyper1925	N_adequate_7	0.0E+00	0.28	0.02	0.26	2	236579007	cg27076454	AGAP1;AGAP1	Body;Body	chr2:236578080-236580153	Island
hyper1926	N_adequate_7	3.1E-07	0.11	0.02	0.08	3	159943101	cg12177207	LOC401097	TSS1500	chr3:159942916-159944431	Island
hyper1927	N_adequate_7	8.5E-04	0.15	0.06	0.10	3	173115774	cg07489890	NLGN1	TSS1500	chr3:173115404-173115775	Island
hyper1928	N_adequate_7	6.5E-02	0.64	0.49	0.15	3	173303193	cg06304401	NLGN1	5'UTR		
hyper1929	N_adequate_7	1.9E-02	0.30	0.11	0.19	3	194408071	cg00806461	FAM43A	1stExon	chr3:194406440-194409045	Island
hyper1930	N_adequate_7	0.0E+00	0.15	0.01	0.14	4	15704680	cg00731785	BST1;BST1	1stExon;5'UTR	chr4:15704640-15705000	Island

hyper1931	N_adequate_7	2.7E-06	0.22	0.09	0.12	4	15704795	cg13528854	BST1	1stExon	chr4:15704640-15705000	Island
hyper1932	N_adequate_7	1.3E-06	0.22	0.10	0.12	4	15704844	cg17924813	BST1	1stExon	chr4:15704640-15705000	Island
hyper1933	N_adequate_7	5.2E-02	0.24	0.09	0.15	5	42995369	cg24676817			chr5:42995122-42995415	Island
hyper1934	N_adequate_7	8.0E-05	0.14	0.06	0.08	5	43042108	cg16970804	LOC153684	TSS200	chr5:43042107-43043477	Island
hyper1935	N_adequate_7	0.0E+00	0.16	0.02	0.14	5	43042177	cg16628484	LOC153684	TSS200	chr5:43042107-43043477	Island
hyper1936	N_adequate_7	2.6E-03	0.22	0.12	0.10	5	43042233	cg22196610	LOC153684	TSS200	chr5:43042107-43043477	Island
hyper1937	N_adequate_7	0.0E+00	0.21	0.01	0.20	5	43042288	cg04710680	LOC153684	Body	chr5:43042107-43043477	Island
hyper1938	N_adequate_7	2.0E-02	0.19	0.09	0.11	5	172386894	cg17360650	RPL26L1;LOC100268168;LOC10(	Body;TSS1500;TSS1500	chr5:172386893-172387102	Island
hyper1939	N_adequate_7	4.6E-08	0.14	0.03	0.11	5	180479829	cg00050152	BTNL9	Body	chr5:180479585-180480959	Island
hyper1940	N_adequate_7	0.0E+00	0.16	0.02	0.14	5	180480234	cg26600608	BTNL9	Body	chr5:180479585-180480959	Island
hyper1941	N_adequate_7	1.0E-06	0.26	0.03	0.24	7	138720287	cg11590647	ZC3HAV1L	Body	chr7:138720044-138721019	Island
hyper1942	N_adequate_7	3.1E-04	0.34	0.08	0.26	7	138720898	cg14347989	ZC3HAV1L	TSS200	chr7:138720044-138721019	Island
hyper1943	N_adequate_7	7.6E-04	0.44	0.07	0.37	7	138720909	cg02657292	ZC3HAV1L	TSS200	chr7:138720044-138721019	Island
hyper1944	N_adequate_7	1.5E-04	0.23	0.09	0.14	7	138721065	cg24251035	ZC3HAV1L	TSS1500	chr7:138720044-138721019	S_Shore
hyper1945	N_adequate_7	1.3E-02	0.18	0.07	0.11	8	38614832	cg11071960	TACC1	5'UTR	chr8:38614414-38615236	Island
hyper1946	N_adequate_7	2.2E-05	0.21	0.10	0.11	8	146176331	cg08699181	ZNF16;ZNF16	TSS200;TSS200	chr8:146175835-146176337	Island
hyper1947	N_adequate_7	6.8E-10	0.18	0.04	0.13	8	146176336	cg07534467	ZNF16;ZNF16	TSS200;TSS200	chr8:146175835-146176337	Island
hyper1948	N_adequate_7	1.9E-10	0.25	0.07	0.18	8	146176420	cg11914370	ZNF16;ZNF16	TSS200;TSS200	chr8:146175835-146176337	S_Shore
hyper1949	N_adequate_7	5.5E-02	0.38	0.17	0.21	8	146176436	cg13734177	ZNF16;ZNF16	TSS200;TSS200	chr8:146175835-146176337	S_Shore
hyper1950	N_adequate_7	1.4E-02	0.06	0.01	0.04	10	15413437	cg18375586	FAM171A1	TSS1500	chr10:15412419-15413040	S_Shore
hyper1951	N_adequate_7	4.8E-02	0.21	0.07	0.15	11	2161892	cg13791131	IGF2AS;INS-IGF2;IGF2;IGF2;IGF:	Body;Body;5'UTR;5'UTR;Body	chr11:2158951-2162484	Island
hyper1952	N_adequate_7	1.4E-03	0.22	0.08	0.14	11	60775233	cg01877352	CD6	Body	chr11:60775036-60776307	Island
hyper1953	N_adequate_7	7.8E-02	0.38	0.04	0.34	13	33590273	cg05855588	KL	TSS1500	chr13:33589928-33591428	Island
hyper1954	N_adequate_7	3.5E-02	0.34	0.03	0.30	13	33590495	cg05116906	KL	TSS200	chr13:33589928-33591428	Island
hyper1955	N_adequate_7	1.8E-05	0.23	0.03	0.20	14	27066404	cg07559273	NOVA1;NOVA1;NOVA1	Body;Body;Body	chr14:27066313-27066578	Island
hyper1956	N_adequate_7	9.7E-10	0.28	0.08	0.19	15	55790446	cg11560374	DYX1C1;DYX1C1;DYX1C1	Body;Body;Body	chr15:55790301-55790754	Island
hyper1957	N_adequate_7	0.0E+00	0.27	0.01	0.26	15	55790509	cg21053602	DYX1C1;DYX1C1;DYX1C1	Body;Body;Body	chr15:55790301-55790754	Island
hyper1958	N_adequate_7	0.0E+00	0.29	0.08	0.21	15	55790608	cg18344452	DYX1C1;DYX1C1;DYX1C1	5'UTR;5'UTR;5'UTR	chr15:55790301-55790754	Island
hyper1959	N_adequate_7	0.0E+00	0.26	0.06	0.20	15	55790753	cg07033615	DYX1C1;DYX1C1;DYX1C1	5'UTR;5'UTR;5'UTR	chr15:55790301-55790754	Island
hyper1960	N_adequate_7	2.3E-05	0.26	0.07	0.19	15	101458615	cg24163563	LRRK1	TSS1500	chr15:101458286-101460223	Island
hyper1961	N_adequate_7	3.0E-07	0.35	0.04	0.30	15	101458783	cg06679538	LRRK1	TSS1500	chr15:101458286-101460223	Island
hyper1962	N_adequate_7	2.4E-08	0.32	0.04	0.28	15	101459283	cg25331703	LRRK1	TSS200	chr15:101458286-101460223	Island
hyper1963	N_adequate_7	2.9E-02	0.36	0.08	0.28	15	101460077	cg10253457	LRRK1	5'UTR	chr15:101458286-101460223	Island
hyper1964	N_adequate_7	1.0E-03	0.31	0.10	0.21	16	71518618	cg03711682	ZNF19	5'UTR		
hyper1965	N_adequate_7	0.0E+00	0.31	0.03	0.28	16	90085722	cg11239016	DBNDD1	1stExon	chr16:90085109-90086419	Island
hyper1966	N_adequate_7	7.2E-08	0.07	0.01	0.06	17	6555443	cg24184221	MED31;C17orf100	TSS1500;1stExon	chr17:6554832-6555585	Island
hyper1967	N_adequate_7	1.2E-02	0.14	0.07	0.07	19	45931688	cg26322788			chr19:45931687-45932295	Island
hyper1968	N_adequate_7	0.0E+00	0.23	0.01	0.21	19	47933586	cg01263386	SLC8A2	Body	chr19:47933295-47933827	Island
hyper1969	N_adequate_7	2.8E-02	0.28	0.10	0.18	20	61050560	cg15749748	GATA5	Body	chr20:61049361-61051897	Island
hyper1970	N_adequate_7	7.9E-03	0.39	0.04	0.35	22	31218629	cg17330672	OSBP2	Body	chr22:31218226-31218893	Island
hyper1971	N_adequate_8	3.6E-03	0.20	0.08	0.12	1	14925856	cg00185566	KIAA1026;KIAA1026	Body;Body	chr1:14924610-14925993	Island
hyper1972	N_adequate_8	3.0E-06	0.31	0.10	0.20	1	15850417	cg05660106	CASP9;CASP9	Body;Body	chr1:15850493-15850699	N_Shore
hyper1973	N_adequate_8	2.6E-03	0.33	0.03	0.30	1	21766547	cg10019095	NBPF3	TSS200	chr1:21766272-21767224	Island
hyper1974	N_adequate_8	1.5E-03	0.26	0.09	0.17	1	101701734	cg05160678	S1PR1	TSS1500	chr1:101701785-101702068	N_Shore
hyper1975	N_adequate_8	3.6E-02	0.35	0.11	0.24	1	101701965	cg05338680	S1PR1	TSS1500	chr1:101701785-101702068	Island
hyper1976	N_adequate_8	1.8E-05	0.13	0.04	0.09	1	148556046	cg18376472			chr1:148555640-148556626	Island
hyper1977	N_adequate_8	0.0E+00	0.12	0.02	0.11	1	148556049	cg06616806			chr1:148555640-148556626	Island
hyper1978	N_adequate_8	6.3E-02	0.28	0.18	0.11	1	156096301	cg03946955	LMNA;LMNA;LMNA	Body;Body;Body		
hyper1979	N_adequate_8	4.9E-04	0.07	0.01	0.06	2	30144547	cg13552710	ALK	TSS200	chr2:30142831-30144624	Island
hyper1980	N_adequate_8	7.2E-05	0.08	0.02	0.06	2	43202474	cg04011671				
hyper1981	N_adequate_8	2.7E-07	0.16	0.02	0.14	2	43202481	cg03717364				
hyper1982	N_adequate_8	2.3E-02	0.18	0.07	0.11	2	145277843	cg26522240	ZEB2;ZEB2;ZEB2;ZEB2;ZEB2	Body;5'UTR;1stExon;1stExon;5'U1	chr2:145281736-145282269	N_Shelf
hyper1983	N_adequate_8	1.2E-06	0.24	0.09	0.16	2	228736089	cg14646075	WDR69	TSS1500	chr2:228736230-228736544	N_Shore
hyper1984	N_adequate_8	4.0E-05	0.29	0.10	0.18	2	228736135	cg14329157	WDR69	TSS200	chr2:228736230-228736544	N_Shore
hyper1985	N_adequate_8	9.6E-09	0.33	0.08	0.25	2	228736145	cg22079747	WDR69	TSS200	chr2:228736230-228736544	N_Shore
hyper1986	N_adequate_8	3.8E-06	0.32	0.02	0.30	2	228736253	cg00063174	WDR69	TSS200	chr2:228736230-228736544	Island
hyper1987	N_adequate_8	6.9E-08	0.29	0.02	0.28	2	228736258	cg20647610	WDR69	TSS200	chr2:228736230-228736544	Island
hyper1988	N_adequate_8	6.0E-08	0.31	0.02	0.29	2	228736357	cg23807890	WDR69;WDR69	1stExon;5'UTR	chr2:228736230-228736544	Island
hyper1989	N_adequate_8	9.1E-07	0.28	0.08	0.21	2	228736449	cg21577049	WDR69	Body	chr2:228736230-228736544	Island
hyper1990	N_adequate_8	2.9E-06	0.14	0.02	0.12	2	228736459	cg01216370	WDR69	Body	chr2:228736230-228736544	Island
hyper1991	N_adequate_8	3.1E-03	0.19	0.05	0.14	3	27756844	cg16700000			chr3:27756660-27756941	Island
hyper1992	N_adequate_8	1.8E-02	0.19	0.04	0.14	3	27756851	cg05409218			chr3:27756660-27756941	Island
hyper1993	N_adequate_8	2.9E-07	0.28	0.05	0.22	9	69786306	cg14346461			chr9:69785274-69787720	Island
hyper1994	N_adequate_8	2.9E-05	0.08	0.02	0.07	11	64059307	cg10028227	KCNK4	5'UTR	chr11:64058808-64060118	Island
hyper1995	N_adequate_8	6.2E-07	0.32	0.12	0.19	12	122231455	cg09088508	RHOF	1stExon	chr12:122230903-122232359	Island
hyper1996	N_adequate_8	3.9E-09	0.13	0.03	0.10	12	122231626	cg03341523	RHOF	TSS200	chr12:122230903-122232359	Island
hyper1997	N_adequate_8	2.2E-05	0.18	0.10	0.07	12	122231757	cg25776635	RHOF	TSS200	chr12:122230903-122232359	Island
hyper1998	N_adequate_8	1.4E-07	0.16	0.06	0.09	12	122231765	cg24097153	RHOF	TSS200	chr12:122230903-122232359	Island
hyper1999	N_adequate_8	1.9E-10	0.16	0.05	0.11	12	122231866	cg02539714	RHOF	TSS1500	chr12:122230903-122232359	Island
hyper2000	N_adequate_8	0.0E+00	0.31	0.08	0.23	12	122231927	cg26269863	RHOF	TSS1500	chr12:122230903-122232359	Island
hyper2001	N_adequate_8	8.0E-02	0.33	0.10	0.23	15	72565024	cg08294267	PARP6	TSS1500	chr15:72564442-72565165	Island
hyper2002	N_adequate_8	1.5E-06	0.29	0.05	0.24	16	744861	cg03349020	FBXL16	Body	chr16:743924-745943	Island
hyper2003	N_adequate_8	1.5E-02	0.39	0.10	0.30	16	32857551	cg06781853			chr16:32857241-32858277	Island
hyper2004	N_adequate_9	4.1E-02	0.04	0.01	0.03	1	153939849	cg22228373	SLC39A1;CREB3L4	5'UTR;TSS1500	chr1:153939643-153939980	Island
hyper2005	N_adequate_9	2.0E-08	0.25	0.02	0.23	2	242823485	cg23370306			chr2:242823446-242823970	Island
hyper2006	N_adequate_9	9.6E-04	0.12	0.02	0.10	2	242823679	cg16595261			chr2:242823446-242823970	Island
hyper2007	N_adequate_9	6.7E-07	0.25	0.03	0.22	3	14643854	cg22991512			chr3:14643718-14644830	Island
hyper2008	N_adequate_9	9.0E-03	0.15	0.02	0.13	3	14643925	cg12146386			chr3:14643718-14644830	Island
hyper2009	N_adequate_9	2.6E-05	0.20	0.11	0.09	3	139258822	cg06543018	RBP1;RBP1;RBP1	TSS200;TSS200;TSS200	chr3:139257712-139257949	S_Shore
hyper2010	N_adequate_9	9.0E-06	0.16	0.03	0.13	3	139258939	cg23363832	RBP1;RBP1;RBP1	TSS1500;TSS1500;TSS1500	chr3:139257712-139257949	S_Shore
hyper2011	N_adequate_9	2.7E-02	0.07	0.01	0.05	4	4387917	cg20460797	D4S234E;D4S234E	TSS200;TSS200	chr4:4387593-4389919	Island
hyper2012	N_adequate_9	2.8E-02	0.07	0.01	0.07	4	113436232	cg10511618	NEUROG2	Body	chr4:113435898-113438002	Island
hyper2013	N_adequate_9	1.6E-02	0.14	0.08	0.06	5	140893101	cg00987031			chr5:140892913-140893189	Island
hyper2014	N_adequate_9	4.3E-05	0.17	0.04	0.13	6	159590231	cg00157796	FNDC1	TSS200	chr6:159589636-159591319	Island
hyper2015	N_adequate_9	0.0E+00	0.33	0.06	0.27	7	21467723	cg27007358	SP4;SP4	5'UTR;1stExon	chr7:21466646-21467806	Island
hyper2016	N_adequate_9	1.8E-06	0.18	0.05	0.14	7	27135723	cg26744375	HOXA1;HOXA1	TSS200;TSS200	chr7:27135342-27136736	Island
hyper2017	N_adequate_9	4.6E-02	0.18	0.02	0.16	8	70746312	cg26753018	SLCO5A1;SLCO5A1;SLCO5A1	5'UTR;TSS1500;TSS1500	chr8:70744098-70747441	Island
hyper2018	N_adequate_9	5.3E-02	0.39	0.08	0.32	8	143532613	cg02707869			chr8:143531118-143534495	Island
hyper2019	N_adequate_9	7.2E-08	0.37	0.08	0.29	8	143534100	cg07300200			chr8:143531118-143534495	Island
hyper2020	N_adequate_9	5.9E-05	0.17	0.06	0.11	11	110583549	cg04974587	ARHGAP20	TSS200	chr11:110581839-110583883	Island
hyper2021	N_adequate_9	1.7E-05	0.37	0.13	0.24	11	110583599	cg26135012	ARHGAP20	TSS200	chr11:110581839-110583883	Island
hyper2022	N_adequate_9	5.0E-02	0.37	0.05	0.32	12	3600317	cg06029846	PRMT8	TSS200	chr12:3599966-3603008	Island
hyper2023	N_adequate_9	8.8E-03	0.29	0.05	0.24	12	5018984	cg12080391	KCNA1	TSS200	chr12:5018585-5021171	Island
hyper2024	N_adequate_9	1.2E-06	0.54	0.15	0.39	12	30949050	cg17037963			chr12:30948548-30949757	Island
hyper2025	N_adequate_9	1.5E-09	0.38	0.05	0.32	12	30949073	cg08949339			chr12:30948548-30949757	Island
hyper2026	N_adequate_9	1.9E-10	0.33	0.07	0.26	12	30949414	cg11821068			chr12:30948548-30949757	Island
hyper2027	N_adequate_9	1.6E-07	0.45	0.11	0.34	12	31917778	ch.12.706878F				
hyper2028	N_adequate_9	7.8E-07	0.20	0.03	0.18	12	34492938	cg01837362			chr12:34493973-34496968	N_Shore
hyper2029	N_adequate_9	2.4E-07	0.08	0.01	0.07	12	46385625	cg04385631	SFRS2IP	TSS1500	chr12:46385607-46385887	Island
hyper2030	N_adequate_9	1.1E-06	0.15	0.02	0.13	12	130527104	cg10233825	LOC100190940	TSS1500	chr12:130526915-130527117	Island
hyper2031	N_adequate_9	3.3E-02	0.25	0.09	0.16	12	130645423	cg23381749			chr12:130645327-130648988	Island
hyper2032	N_adequate_9	8.3E-08	0.20	0.09	0.11	12	130646129	cg17537177	FZD10	TSS1500	chr12:130645327-130648988	Island
hyper2033	N_adequate_9	9.6E-03	0.14	0.03	0.11	12	130646256	cg18209212	FZD10	TSS1500	chr12:130645327-130648988	Island
hyper2034	N_adequate_9	0.0E+00	0.26	0.02	0.24	12	130647213	cg15928093	FZD10;FZD10	5'UTR;1stExon	chr12:130645327-130648988	Island
hyper2035	N_adequate_9	3.4E-05	0.40	0.14	0.26	12	130647289	cg27232145	FZD10;FZD10	5'UTR;1stExon	chr12:130645327-130648988	Island
hyper2036	N_adequate_9	1.1E-04	0.59	0.27	0.32	12	132987128	cg16608087			chr12:132990777-132990987	N_Shelf
hyper2037	N_adequate_9	3.7E-02	0.18	0.07	0.11	13	27335089	cg00590851	GPR12	TSS200	chr13:27334226-27335205	Island
hyper2038	N_adequate_9	1.2E-02	0.12	0.06	0.06	15	26327496	cg18139256			chr15:26327495-26327896	Island
hyper2039	N_adequate_9	9.5E-04	0.25	0.04	0.20	15	60296469	cg07060006	FOXB1;FOXB1	5'UTR;1stExon	chr15:60296135-60298520	Island
hyper2040	N_adequate_9	8.6E-02	0.11	0.03	0.08	16	1031458	cg02049499	SOX8	TSS1500	chr16:1029878-1035327	Island
hyper2041	N_adequate_9	0.0E+00	0.39	0.01	0.38	16	31453948	cg06476192	ZNF843	5'UTR	chr16:31453943-31454561	Island
hyper2042	N_adequate_9	4.2E-04	0.16	0.07	0.09	16	31454170	cg02699540	ZNF843;ZNF843	1stExon;5'UTR	chr16:31453943-31454561	Island
hyper2043	N_adequate_9	5.4E-07	0.31	0.02	0.29	19	40562022	cg20732626	ZNF780B	5'UTR	chr19:40561820-40562150	Island
hyper2044	N_adequate_9	4.6E-04	0.16	0.04	0.11	19	58609744	cg18428688	ZSCAN18;ZSCAN18;ZSCAN18;Z!	Body;TSS200;TSS200;TSS200	chr19:58609338-58609988	Island
hyper2045	N_adequate_9	1.7E-02	0.13	0.05	0.07	19	58609764	cg07657064	ZSCAN18;ZSCAN18;ZSCAN18;Z!	Body;TSS200;TSS200;TSS200	chr19:58609338-58609988	Island
hyper2046	N_adequate_9	1.2E-02	0.22	0.10	0.12	19	58666209	cg15517658			chr19:58666023-58666833	Island
hyper2047	N_adequate_9	1.0E-02	0.08	0.02	0.06	19	58666456	cg02352723			chr19:58666023-58666833	Island
hyper2048	N_adequate_9	3.6E-07	0.14	0.02	0.12	20	53092233	cg00741836	DOK5	TSS200	chr20:53091733-53092993	Island
hyper2049	N_adequate_9	8.9E-02	0.06	0.01	0.05	22	20790985	cg20401551	SCARF2;SCARF2	Body;Body	chr22:20790638-20792665	Island
hyper2050	N_excessive_1	7.8E-10	0.16	0.02	0.14	1	63782639	cg25761793			chr1:63782394-63790471	Island
hyper2051	N_excessive_1	1.8E-06	0.21	0.06	0.15	1	210110998	cg07581146	SYT14;SYT14;SYT14;SYT14;SYT	TSS1500;TSS1500;TSS1500;TSS	chr1:210111179-210112054	N_Shore
hyper2052	N_excessive_1	2.4E-02	0.11	0.04	0.07	1	248020692	cg23054189	TRIM58	1stExon	chr1:248020330-248021252	Island
hyper2053	N_excessive_1	2.2E-04	0.09	0.03	0.06	2	1858740	cg05974816	MYT1L	Body		
hyper2054	N_excessive_1	2.8E-07	0.24	0.07	0.16	2	20646880	cg01419675	RHOB;RHOB	5'UTR;1stExon	chr2:20646428-20647988	Island
hyper2055	N_excessive_1	3.0E-02	0.36	0.16	0.20	2	27341458	cg22740783	CGREF1;CGREF1;CGREF1	5'UTR;5'UTR;5'UTR	chr2:27341567-27341923	N_Shore
hyper2056	N_excessive_1	2.8E-04	0.28	0.07	0.21	2	162101083	cg08807892			chr2:162101439-162101650	N_Shore
hyper2057	N_excessive_1	1.9E-10	0.30	0.06	0.24	2	198363565	cg17128646	HSPD1;HSPE1;HSPD1	Body;TSS1500;Body	chr2:198363689-198365405	N_Shore
hyper2058	N_excessive_1	0.0E+00	0.34	0.04	0.30	2	198363868	cg04183933	HSPD1;HSPD1;HSPE1	5'UTR;5'UTR;TSS1500	chr2:198363689-198365405	Island
hyper2059	N_excessive_1	0.0E+00	0.59	0.09	0.50	2	198364074	cg06611076	HSPD1;HSPD1;HSPE1	5'UTR;5'UTR;TSS1500	chr2:198363689-198365405	Island

hyper2060	N_excessive_1	0.0E+00	0.41	0.04	0.37	2	198364115	cg05654645	HSPD1;HSPD1;HSPE1	5'UTR;5'UTR;TSS1500	chr2:198363689-198365405	Island
hyper2061	N_excessive_1	0.0E+00	0.57	0.10	0.48	2	198364282	cg08602604	HSPD1;HSPD1;HSPE1	5'UTR;5'UTR;TSS1500	chr2:198363689-198365405	Island
hyper2062	N_excessive_1	0.0E+00	0.46	0.01	0.45	2	198364585	cg05767788	HSPD1;HSPD1;HSPD1;HSPE1	1stExon;5'UTR;5'UTR;TSS200	chr2:198363689-198365405	Island
hyper2063	N_excessive_1	0.0E+00	0.40	0.02	0.38	2	198364596	cg23159081	HSPD1;HSPD1;HSPD1;HSPE1	1stExon;5'UTR;5'UTR;TSS200	chr2:198363689-198365405	Island
hyper2064	N_excessive_1	0.0E+00	0.35	0.05	0.30	2	198364743	cg00437134	HSPE1;HSPE1;HSPD1;HSPD1	1stExon;5'UTR;5'UTR;TSS200	chr2:198363689-198365405	Island
hyper2065	N_excessive_1	5.8E-10	0.36	0.09	0.28	2	198364931	cg25273507	HSPE1;HSPE1;HSPD1;HSPD1;H	1stExon;5'UTR;5'UTR;1stExon;TS	chr2:198363689-198365405	Island
hyper2066	N_excessive_1	0.0E+00	0.46	0.04	0.42	2	198365065	cg10884341	HSPE1;HSPE1;HSPD1;HSPD1	1stExon;5'UTR;TSS1500;TSS200	chr2:198363689-198365405	Island
hyper2067	N_excessive_1	0.0E+00	0.46	0.01	0.45	2	198365119	cg02208776	HSPE1;HSPE1;HSPD1;HSPD1	1stExon;5'UTR;TSS1500;TSS200	chr2:198363689-198365405	Island
hyper2068	N_excessive_1	0.0E+00	0.40	0.03	0.38	2	198365139	cg27005255	HSPE1;HSPE1;HSPD1;HSPD1	1stExon;5'UTR;TSS1500;TSS200	chr2:198363689-198365405	Island
hyper2069	N_excessive_1	1.1E-07	0.21	0.03	0.18	2	237076726	cg16023545	GBX2	TSS200	chr2:237071794-237078762	Island
hyper2070	N_excessive_1	3.8E-05	0.28	0.03	0.26	2	237076811	cg21426003	GBX2	TSS200	chr2:237071794-237078762	Island
hyper2071	N_excessive_1	2.5E-04	0.36	0.04	0.32	2	237076815	cg19761848	GBX2	TSS200	chr2:237071794-237078762	Island
hyper2072	N_excessive_1	0.0E+00	0.31	0.07	0.24	3	59035660	cg19830192	C3orf67;C3orf67	5'UTR;1stExon	chr3:59035406-59035944	Island
hyper2073	N_excessive_1	0.0E+00	0.12	0.01	0.11	3	59035796	cg03187299	C3orf67	TSS200	chr3:59035406-59035944	Island
hyper2074	N_excessive_1	0.0E+00	0.82	0.07	0.75	5	38557386	cg18848688	LIFR;LIFR	TSS1500;5'UTR	chr5:38556222-38557563	Island
hyper2075	N_excessive_1	1.3E-02	0.31	0.10	0.20	5	95159614	cg10949007	GLRX;GLRX	TSS1500;TSS1500		
hyper2076	N_excessive_1	1.1E-05	0.14	0.05	0.09	6	29720641	cg01426208			chr6:29720381-29721101	Island
hyper2077	N_excessive_1	3.9E-10	0.47	0.10	0.38	6	32941126	cg02448295	BRD2;BRD2	Body;Body	chr6:32939275-32940954	S_Shore
hyper2078	N_excessive_1	0.0E+00	0.25	0.07	0.18	7	56119028	cg03532005	CCT6A;PSPH;CCT6A;PSPH	TSS1500;5'UTR;TSS1500;1stExor	chr7:56119045-56119963	N_Shore
hyper2079	N_excessive_1	2.3E-09	0.27	0.08	0.19	7	56119046	cg02753865	CCT6A;PSPH;CCT6A;PSPH	TSS1500;5'UTR;TSS1500;1stExor	chr7:56119045-56119963	Island
hyper2080	N_excessive_1	0.0E+00	0.33	0.05	0.28	7	56119079	cg01830765	CCT6A;PSPH;CCT6A;PSPH	TSS1500;5'UTR;TSS1500;1stExor	chr7:56119045-56119963	Island
hyper2081	N_excessive_1	1.6E-09	0.29	0.05	0.24	7	56119212	cg22128468	PSPH;PSPH;CCT6A;CCT6A	5'UTR;1stExon;TSS200;TSS200	chr7:56119045-56119963	Island
hyper2082	N_excessive_1	8.6E-06	0.14	0.03	0.12	7	56119218	cg10749792	PSPH;PSPH;CCT6A;CCT6A	5'UTR;1stExon;TSS200;TSS200	chr7:56119045-56119963	Island
hyper2083	N_excessive_1	7.6E-05	0.86	0.36	0.50	9	138413253	cg00040426	LCN1	TSS200		
hyper2084	N_excessive_1	1.5E-06	0.15	0.05	0.11	10	28822390	cg00955290	WAC;WAC;WAC	TSS200;Body;TSS200	chr10:28821189-28823092	Island
hyper2085	N_excessive_1	6.8E-10	0.40	0.07	0.33	11	61879966	cg13875901				
hyper2086	N_excessive_1	0.0E+00	0.35	0.07	0.28	12	81331457	cg08480223	LIN7A	1stExon	chr12:81330608-81331514	Island
hyper2087	N_excessive_1	0.0E+00	0.31	0.08	0.23	12	81331469	cg17061244	LIN7A	1stExon	chr12:81330608-81331514	Island
hyper2088	N_excessive_1	0.0E+00	0.38	0.06	0.33	12	81331486	cg23441441	LIN7A	1stExon	chr12:81330608-81331514	Island
hyper2089	N_excessive_1	8.6E-03	0.33	0.05	0.28	12	81331660	cg21152690	LIN7A;LIN7A	1stExon;5'UTR	chr12:81330608-81331514	S_Shore
hyper2090	N_excessive_1	6.9E-02	0.27	0.06	0.21	12	81331845	cg14874351	LIN7A	TSS200	chr12:81330608-81331514	S_Shore
hyper2091	N_excessive_1	4.3E-02	0.18	0.08	0.10	14	100438957	cg21054473			chr14:100437381-100438863	S_Shore
hyper2092	N_excessive_1	8.0E-02	0.25	0.08	0.17	16	49518414	cg09231171				
hyper2093	N_excessive_1	5.1E-07	0.36	0.08	0.28	19	52489783	cg24162630	ZNF350	5'UTR		
hyper2094	N_excessive_1	5.9E-03	0.31	0.03	0.28	19	52490058	cg02573825	ZNF350;ZNF350	1stExon;5'UTR		
hyper2095	N_excessive_1	5.8E-10	0.36	0.08	0.29	19	52490069	cg27565719	ZNF350;ZNF350	1stExon;5'UTR		
hyper2096	N_excessive_1	1.9E-10	0.42	0.07	0.35	19	52490101	cg27654641	ZNF350	TSS200		
hyper2097	N_excessive_1	4.3E-07	0.34	0.07	0.27	19	52490173	cg03577153	ZNF350	TSS200		
hyper2098	N_excessive_1	1.5E-08	0.42	0.07	0.35	19	52490199	cg25835225	ZNF350	TSS200		
hyper2099	N_excessive_1	1.0E-08	0.33	0.05	0.28	19	52490211	cg05017253	ZNF350	TSS200		
hyper2100	N_excessive_1	0.0E+00	0.40	0.07	0.33	19	52490223	cg18649745	ZNF350	TSS200		
hyper2101	N_excessive_1	8.2E-02	0.23	0.04	0.19	22	21386914	cg05684406	SLC7A4	TSS200	chr22:21386493-21387000	Island
hyper2102	N_excessive_1	1.1E-06	0.22	0.07	0.16	22	33453893	cg14062468	SYN3	5'UTR	chr22:33453892-33454505	Island
hyper2103	N_excessive_1	4.2E-07	0.09	0.02	0.08	22	33454324	cg05206884	SYN3;SYN3	5'UTR;1stExon	chr22:33453892-33454505	Island
hyper2104	N_excessive_2	3.5E-03	0.07	0.02	0.05	1	21058625	cg04902671	SH2D5;SH2D5	5'UTR;5'UTR	chr1:21058006-21059366	Island
hyper2105	N_excessive_2	0.0E+00	0.08	0.00	0.08	1	100503596	cg24955406	HIAT1	TSS200	chr1:100503482-100504404	Island
hyper2106	N_excessive_2	6.6E-03	0.04	0.01	0.03	1	154377970	cg03957204	IL6R;IL6R;IL6R;IL6R	5'UTR;1stExon;1stExon;5'UTR	chr1:154377670-154378445	Island
hyper2107	N_excessive_2	8.7E-03	0.23	0.03	0.20	1	166853578	cg26248173			chr1:166853253-166853790	Island
hyper2108	N_excessive_2	1.9E-08	0.07	0.01	0.06	2	24583331	cg18920904	ITSN2;ITSN2;ITSN2;ITSN2;ITSN2	1stExon;1stExon;5'UTR;5'UTR;5'U	chr2:24582620-24583611	Island
hyper2109	N_excessive_2	6.3E-03	0.11	0.02	0.08	2	25149977	cg15423357				
hyper2110	N_excessive_2	5.0E-07	0.40	0.16	0.25	3	185826590	cg17466567	ETV5	5'UTR	chr3:185825462-185827003	Island
hyper2111	N_excessive_2	3.4E-04	0.06	0.01	0.05	4	10118516	cg10699215	WDR1;WDR1;WDR1;WDR1	1stExon;5'UTR;5'UTR;1stExon	chr4:10117575-10118952	Island
hyper2112	N_excessive_2	9.7E-09	0.73	0.46	0.27	4	14361291	cg05036183				
hyper2113	N_excessive_2	7.6E-04	0.22	0.08	0.14	4	40751770	cg19786920	NSUN7	TSS200	chr4:40751842-40752493	N_Shore
hyper2114	N_excessive_2	1.3E-04	0.18	0.01	0.17	4	40751845	cg01143804	NSUN7	TSS200	chr4:40751842-40752493	Island
hyper2115	N_excessive_2	1.2E-04	0.30	0.05	0.25	4	40752415	cg08508988	NSUN7	5'UTR	chr4:40751842-40752493	Island
hyper2116	N_excessive_2	5.1E-09	0.05	0.01	0.04	5	16935695	cg19510792	MYO10	Body	chr5:16935555-16936408	Island
hyper2117	N_excessive_2	8.3E-03	0.28	0.06	0.22	5	57879402	cg26580801	RAB3C	Body	chr5:57878725-57879177	S_Shore
hyper2118	N_excessive_2	6.4E-08	0.14	0.04	0.10	5	70883065	cg16320199	MCCC2	TSS200	chr5:70882936-70883413	Island
hyper2119	N_excessive_2	0.0E+00	0.12	0.01	0.12	5	70883072	cg18788891	MCCC2	TSS200	chr5:70882936-70883413	Island
hyper2120	N_excessive_2	1.3E-08	0.08	0.01	0.07	5	70883087	cg24034966	MCCC2	TSS200	chr5:70882936-70883413	Island
hyper2121	N_excessive_2	2.4E-04	0.16	0.08	0.08	5	70883173	cg18359921	MCCC2;MCCC2	5'UTR;1stExon	chr5:70882936-70883413	Island
hyper2122	N_excessive_2	9.7E-11	0.26	0.07	0.19	5	70883341	cg24558977	MCCC2	1stExon	chr5:70882936-70883413	Island
hyper2123	N_excessive_2	7.8E-10	0.22	0.03	0.20	5	72598965	cg11795854			chr5:72598958-72599656	Island
hyper2124	N_excessive_2	4.1E-06	0.18	0.07	0.11	5	72599143	cg02495823			chr5:72598958-72599656	Island
hyper2125	N_excessive_2	6.4E-09	0.33	0.08	0.25	5	72599228	cg15168727			chr5:72598958-72599656	Island
hyper2126	N_excessive_2	2.7E-02	0.16	0.05	0.11	6	10415653	cg11063729	TFAP2A;TFAP2A	Body;TSS200	chr6:10417384-10417842	N_Shore
hyper2127	N_excessive_2	5.6E-03	0.18	0.10	0.09	6	26271587	cg02909790	HIST1H3G	1stExon	chr6:26271345-26271588	Island
hyper2128	N_excessive_2	2.4E-02	0.17	0.07	0.10	6	26271716	cg14273822	HIST1H3G;HIST1H2BI	TSS200;TSS1500	chr6:26272470-26272696	N_Shore
hyper2129	N_excessive_2	1.5E-03	0.21	0.08	0.13	6	26271718	cg00036011	HIST1H3G;HIST1H2BI	TSS200;TSS1500	chr6:26272470-26272696	N_Shore
hyper2130	N_excessive_2	1.3E-03	0.17	0.08	0.09	6	26272695	cg23233200	HIST1H3G;HIST1H2BI	TSS1500;TSS1500	chr6:26272470-26272696	Island
hyper2131	N_excessive_2	2.6E-02	0.23	0.11	0.12	6	28956312	cg16622920			chr6:28956106-28956578	Island
hyper2132	N_excessive_2	3.8E-03	0.24	0.03	0.21	6	32145342	cg06529658	RNF5P1;RNF5;AGPAT1;AGPAT1	TSS1500;TSS1500;TSS1500;5'UTR		
hyper2133	N_excessive_2	5.9E-02	0.30	0.07	0.23	6	32145470	cg03237964	RNF5P1;RNF5;AGPAT1	TSS1500;TSS1500;5'UTR		
hyper2134	N_excessive_2	8.4E-02	0.22	0.05	0.18	6	32145543	cg01074928	RNF5P1;RNF5;AGPAT1	TSS1500;TSS1500;5'UTR		
hyper2135	N_excessive_2	7.0E-02	0.24	0.06	0.18	6	32145904	cg08049198	AGPAT1;RNF5P1;RNF5	TSS200;TSS1500;TSS1500		
hyper2136	N_excessive_2	5.8E-02	0.31	0.06	0.25	6	32146548	cg02973270	AGPAT1;RNF5;RNF5P1	TSS1500;Body;Body		
hyper2137	N_excessive_2	1.3E-02	0.43	0.14	0.29	6	163746175	cg21229979	LOC285796	TSS1500		
hyper2138	N_excessive_2	1.7E-02	0.09	0.02	0.06	7	30029238	cg20554228	SCRN1;SCRN1;SCRN1;SCRN1	5'UTR;Body;5'UTR;5'UTR	chr7:30028518-30029822	Island
hyper2139	N_excessive_2	4.5E-03	0.05	0.02	0.03	7	30029390	cg18003762	SCRN1;SCRN1;SCRN1;SCRN1;S	5'UTR;Body;1stExon;5'UTR;5'UTR	chr7:30028518-30029822	Island
hyper2140	N_excessive_2	3.1E-08	0.07	0.01	0.07	7	44925112	cg16770798	PURB	TSS200		
hyper2141	N_excessive_2	4.5E-02	0.13	0.06	0.06	7	131377089	cg20816493				
hyper2142	N_excessive_2	8.2E-02	0.29	0.06	0.24	8	43131353	cg01754525			chr8:43131177-43131487	Island
hyper2143	N_excessive_2	8.6E-04	0.29	0.07	0.22	8	43131431	cg11826726			chr8:43131177-43131487	Island
hyper2144	N_excessive_2	6.9E-02	0.17	0.10	0.07	8	142396947	cg26860265				
hyper2145	N_excessive_2	1.3E-02	0.13	0.06	0.06	8	142397012	cg05027079				
hyper2146	N_excessive_2	8.9E-04	0.13	0.02	0.11	8	142397080	cg21449229				
hyper2147	N_excessive_2	4.8E-06	0.51	0.05	0.46	9	115818857	cg14016089	ZFP37	1stExon	chr9:115818836-115819343	Island
hyper2148	N_excessive_2	1.6E-03	0.51	0.02	0.49	9	115819158	cg14633455	ZFP37	TSS200	chr9:115818836-115819343	Island
hyper2149	N_excessive_2	1.7E-03	0.48	0.08	0.40	9	115819190	cg17749443	ZFP37	TSS200	chr9:115818836-115819343	Island
hyper2150	N_excessive_2	1.5E-02	0.56	0.16	0.40	9	115819364	cg03454353	ZFP37	TSS1500	chr9:115818836-115819343	S_Shore
hyper2151	N_excessive_2	4.4E-03	0.13	0.03	0.10	9	136114331	cg14248367				
hyper2152	N_excessive_2	8.5E-02	0.20	0.10	0.10	10	88392088	cg26830291			chr10:88391746-88392242	Island
hyper2153	N_excessive_2	7.1E-03	0.35	0.14	0.21	10	89419292	cg26477576	PAPSS2;PAPSS2	TSS200;TSS200	chr10:89419346-89420027	N_Shore
hyper2154	N_excessive_2	1.9E-09	0.41	0.08	0.33	10	89419373	cg17916473	PAPSS2;PAPSS2	TSS200;TSS200	chr10:89419346-89420027	Island
hyper2155	N_excessive_2	0.0E+00	0.35	0.06	0.29	10	89419375	cg25968799	PAPSS2;PAPSS2	TSS200;TSS200	chr10:89419346-89420027	Island
hyper2156	N_excessive_2	0.0E+00	0.34	0.02	0.32	10	89419384	cg05628107	PAPSS2;PAPSS2	TSS200;TSS200	chr10:89419346-89420027	Island
hyper2157	N_excessive_2	5.7E-08	0.39	0.09	0.30	10	89419623	cg22897130	PAPSS2;PAPSS2;PAPSS2;PAPS	1stExon;1stExon;5'UTR;5'UTR	chr10:89419346-89420027	Island
hyper2158	N_excessive_2	2.2E-08	0.37	0.11	0.27	10	89419725	cg14433217	PAPSS2;PAPSS2;PAPSS2;PAPS	1stExon;1stExon;5'UTR;5'UTR	chr10:89419346-89420027	Island
hyper2159	N_excessive_2	9.3E-04	0.08	0.03	0.05	10	118976184	cg06526882			chr10:118976049-118976706	Island
hyper2160	N_excessive_2	1.4E-04	0.12	0.03	0.08	10	118976304	cg26834887			chr10:118976049-118976706	Island
hyper2161	N_excessive_2	7.8E-10	0.11	0.01	0.10	11	2291260	cg11373604	ASCL2	1stExon	chr11:2290104-2292932	Island
hyper2162	N_excessive_2	3.0E-02	0.23	0.13	0.10	11	2291264	cg11419064	ASCL2	1stExon	chr11:2290104-2292932	Island
hyper2163	N_excessive_2	7.9E-02	0.05	0.01	0.04	11	7273378	cg22723056	SYT9;SYT9	1stExon;5'UTR	chr11:7272757-7274235	Island
hyper2164	N_excessive_2	7.3E-02	0.30	0.08	0.22	11	28131864	cg05739036	METT5D1;METT5D1	5'UTR;5'UTR	chr11:28131863-28132221	Island
hyper2165	N_excessive_2	5.5E-05	0.22	0.05	0.17	11	28132195	cg06271956	METT5D1;METT5D1	5'UTR;5'UTR	chr11:28131863-28132221	Island
hyper2166	N_excessive_2	5.7E-04	0.07	0.02	0.05	11	116969293	cg22786506	SIK3	TSS1500	chr11:116968849-116969512	Island
hyper2167	N_excessive_2	2.2E-02	0.20	0.06	0.14	14	55907198	cg21836586	TBPL2	1stExon	chr14:55907197-55907433	Island
hyper2168	N_excessive_2	5.5E-02	0.28	0.06	0.22	14	55907374	cg23251248	TBPL2	TSS200	chr14:55907197-55907433	Island
hyper2169	N_excessive_2	1.6E-08	0.11	0.01	0.10	14	59104825	cg13215643	DACT1;DACT1;DACT1;DACT1	5'UTR;1stExon;5'UTR;1stExon	chr14:59104299-59105577	Island
hyper2170	N_excessive_2	5.2E-08	0.30	0.09	0.21	14	103059453	cg15104723	RCOR1	1stExon	chr14:103057926-103060044	Island
hyper2171	N_excessive_2	1.1E-04	0.08	0.02	0.06	14	105190481	cg19730687	ADSSL1	TSS200	chr14:105189927-105190864	Island
hyper2172	N_excessive_2	1.9E-10	0.24	0.04	0.20	15	99640746	cg18315040			chr15:99640592-99641026	Island
hyper2173	N_excessive_2	6.5E-03	0.16	0.09	0.07	17	48624416	cg15780578	SPATA20	TSS200	chr17:48624415-48624723	Island
hyper2174	N_excessive_2	6.8E-06	0.18	0.08	0.10	17	48624421	cg18925864	SPATA20	TSS200	chr17:48624415-48624723	Island
hyper2175	N_excessive_2	8.2E-02	0.15	0.07	0.08	17	48624694	cg24438145	SPATA20	Body	chr17:48624415-48624723	Island
hyper2176	N_excessive_2	1.0E-03	0.07	0.01	0.06	17	63556315	cg18392085	AXIN2	5'UTR	chr17:63555902-63557362	Island
hyper2177	N_excessive_2	1.2E-03	0.11	0.02	0.09	17	74534090	cg02396496	CYGB	TSS1500	chr17:74533281-74534566	Island
hyper2178	N_excessive_2	9.8E-08	0.19	0.05	0.14	19	4472180	cg02761476	HDGF2;HDGF2	TSS200;TSS200	chr19:4472119-4473123	Island
hyper2179	N_excessive_2	3.9E-02	0.28	0.11	0.17	19	20189112	cg20972142	ZNF90	Body		
hyper2180	N_excessive_2	0.0E+00	0.25	0.02	0.23	20	44540777	cg06768188	PLTP;PLTP;PLTP;PLTP	5'UTR;5'UTR;1stExon;1stExon	chr20:44540445-44540957	Island
hyper2181	N_excessive_2	1.9E-10	0.23	0.02	0.21	20	44540794	cg11331678	PLTP;PLTP	TSS200;TSS200	chr20:44540445-44540957	Island
hyper2182	N_excessive_2	3.0E-07	0.31	0.04	0.27	20	44540813	cg27280575	PLTP;PLTP	TSS200;TSS200	chr20:44540445-44540957	Island
hyper2183	N_excessive_2	4.7E-02	0.19	0.09	0.10	20	48184683	cg11746287	PTGIS;PTGIS	5'UTR;1stExon	chr20:48184193-48184833	Island
hyper2184	N_excessive_3	1.7E-02	0.08	0.03	0.06	1	33429891	cg14834760	RNF19B;RNF19B	1stExon;1stExon	chr1:33429531-33430513	Island
hyper2185	N_excessive_3	5.7E-09	0.79	0.16	0.63	2	8422258	cg00594040				
hyper2186	N_excessive_3	2.0E-05	0.12	0.01	0.11	2	238969453	cg20679309	SCLY	TSS200	chr2:238969080-238969919	Island
hyper2187	N_excessive_3	4.0E-03	0.06	0.02	0.04	3	48646722	cg27278382	UQCRC1	Body	chr3:48646620-48647404	Island
hyper2188	N_excessive_3	3.2E-03	0.05	0.01	0.04	4	1795240	cg06883949	FGFR3;FGFR3;FGFR3	5'UTR;5'UTR;5'UTR	chr4:1794442-1797132	Island

hyper2189	N_excessive_3	6.6E-05	0.18	0.06	0.12	4	8582101	cg06643298	GPR78	TSS200	chr4:8582036-8583364	Island
hyper2190	N_excessive_3	6.3E-06	0.13	0.04	0.09	4	8582110	cg21523251	GPR78	TSS200	chr4:8582036-8583364	Island
hyper2191	N_excessive_3	4.2E-08	0.26	0.08	0.19	4	8582565	cg05658182	GPR78;GPR78	1stExon;5'UTR	chr4:8582036-8583364	Island
hyper2192	N_excessive_3	2.9E-10	0.18	0.03	0.15	4	83931713	cg20451727	LIN54;LIN54;LIN54;LIN54;LIN54	1stExon;5'UTR;5'UTR;5'UTR;1stE	chr4:83931642-83932194	Island
hyper2193	N_excessive_3	2.1E-09	0.32	0.10	0.21	4	83931902	cg16854524	LIN54;LIN54;LIN54;LIN54;LIN54	1stExon;5'UTR;5'UTR;5'UTR;1stE	chr4:83931642-83932194	Island
hyper2194	N_excessive_3	2.1E-12	0.16	0.05	0.11	4	83932010	cg03421743	LIN54;LIN54;LIN54	5'UTR;TSS200;TSS200	chr4:83931642-83932194	Island
hyper2195	N_excessive_3	1.9E-10	0.15	0.01	0.14	4	83932058	cg19517378	LIN54;LIN54;LIN54	5'UTR;TSS200;TSS200	chr4:83931642-83932194	Island
hyper2196	N_excessive_3	3.3E-07	0.19	0.03	0.16	4	83932168	cg10724425	LIN54;LIN54;LIN54	5'UTR;TSS200;TSS200	chr4:83931642-83932194	Island
hyper2197	N_excessive_3	3.2E-06	0.20	0.05	0.15	4	83932170	cg23896056	LIN54;LIN54;LIN54	5'UTR;TSS200;TSS200	chr4:83931642-83932194	Island
hyper2198	N_excessive_3	1.8E-09	0.18	0.05	0.13	4	83932193	cg27187586	LIN54;LIN54;LIN54	TSS1500;5'UTR;TSS1500	chr4:83931642-83932194	Island
hyper2199	N_excessive_3	5.8E-10	0.19	0.10	0.09	4	83932227	cg00748821	LIN54;LIN54;LIN54	TSS1500;5'UTR;TSS1500	chr4:83933332-83934370	N_Shore
hyper2200	N_excessive_3	0.0E+00	0.33	0.06	0.28	4	119786577	cg02064368				
hyper2201	N_excessive_3	1.1E-05	0.18	0.04	0.14	5	1931376	cg11439596			chr5:1930754-1931986	Island
hyper2202	N_excessive_3	9.8E-03	0.25	0.07	0.18	5	2038899	cg00213479			chr5:2038527-2038949	Island
hyper2203	N_excessive_3	2.1E-08	0.23	0.05	0.17	5	78429774	cg22188827				
hyper2204	N_excessive_3	9.5E-03	0.06	0.01	0.05	5	179125478	cg08691740	CANX;CANX	TSS1500;TSS1500	chr5:179125341-179126340	Island
hyper2205	N_excessive_3	0.0E+00	0.72	0.04	0.68	7	130353515	cg04678743	TSGA13;COPG2;COPG2	3'UTR;5'UTR;1stExon	chr7:130353070-130353979	Island
hyper2206	N_excessive_3	2.6E-04	0.27	0.10	0.17	8	144635260	cg08688335	GSDMD	TSS1500	chr8:144635308-144636026	N_Shore
hyper2207	N_excessive_3	0.0E+00	0.38	0.06	0.32	8	144691562	cg26507094	PYCRL	Body	chr8:144691400-144692269	Island
hyper2208	N_excessive_3	1.1E-03	0.16	0.08	0.08	9	27528300	cg14173147	MOBKL2B	5'UTR	chr9:27528357-27528725	N_Shore
hyper2209	N_excessive_3	2.1E-02	0.29	0.05	0.23	10	51572203	cg25794402	NCOA4;NCOA4;NCOA4;NCOA4	TSS200;5'UTR;Body;Body	chr10:51572198-51572620	Island
hyper2210	N_excessive_3	1.2E-02	0.30	0.05	0.25	10	51572303	cg07588964	NCOA4;NCOA4;NCOA4;NCOA4	TSS200;5'UTR;Body;Body	chr10:51572198-51572620	Island
hyper2211	N_excessive_3	2.2E-02	0.30	0.12	0.18	10	103539953	cg20653518			chr10:103538815-103539988	Island
hyper2212	N_excessive_3	2.2E-02	0.93	0.68	0.25	10	127464165	cg11254426	MMP21	Body	chr10:127464156-127464385	Island
hyper2213	N_excessive_3	3.0E-03	0.26	0.03	0.23	11	61062837	cg27175288	VWCE	TSS200	chr11:61061751-61063063	Island
hyper2214	N_excessive_3	2.4E-03	0.48	0.27	0.21	11	64058704	cg25881850	KCNK4	TSS200	chr11:64058808-64060118	N_Shore
hyper2215	N_excessive_3	1.3E-05	0.11	0.01	0.09	12	121975243	cg07474126	KDM2B;KDM2B	Body;Body	chr12:121975028-121976140	Island
hyper2216	N_excessive_3	7.8E-04	0.10	0.01	0.09	12	124086727	cg06606198	DDX55	1stExon	chr12:124086217-124087015	Island
hyper2217	N_excessive_3	1.1E-04	0.16	0.07	0.08	15	79576298	cg22694275	ANKRD34C	5'UTR	chr15:79574830-79575211	S_Shore
hyper2218	N_excessive_3	8.7E-02	0.15	0.02	0.13	16	725543	cg07847424	RHBDL1	TSS1500	chr16:729438-735815	N_Shelf
hyper2219	N_excessive_3	3.3E-05	0.25	0.10	0.14	16	1660153	cg20322611	IFT140	5'UTR	chr16:1660054-1665095	Island
hyper2220	N_excessive_3	2.5E-04	0.21	0.05	0.16	16	15982174	cg06490162	C16orf63	Body	chr16:15982245-15982672	N_Shore
hyper2221	N_excessive_3	7.3E-05	0.15	0.01	0.14	16	15982478	cg16401214	C16orf63	TSS200	chr16:15982245-15982672	Island
hyper2222	N_excessive_3	7.2E-04	0.23	0.01	0.22	16	15982508	cg06879630	C16orf63	TSS200	chr16:15982245-15982672	Island
hyper2223	N_excessive_3	1.7E-05	0.21	0.03	0.18	16	15982637	cg09516144	C16orf63	TSS200	chr16:15982245-15982672	Island
hyper2224	N_excessive_3	1.0E-02	0.85	0.32	0.53	16	89009557	cg07630078	CBFA2T3	Body	chr16:89006334-89008600	S_Shore
hyper2225	N_excessive_3	4.2E-03	0.08	0.01	0.07	16	89788035	cg25719439	C16orf7;ZNF276;ZNF276;ZNF276	TSS1500;5'UTR;1stExon;5'UTR	chr16:89786837-89789089	Island
hyper2226	N_excessive_3	2.4E-02	0.11	0.04	0.07	17	4871094	cg02111792	SPAG7	1stExon	chr17:4870189-4871710	Island
hyper2227	N_excessive_3	1.3E-02	0.20	0.02	0.18	17	42083989	cg22241593	NAGS	Body	chr17:42082027-42084972	Island
hyper2228	N_excessive_3	6.9E-03	0.21	0.08	0.13	17	42084612	cg00483503	NAGS	Body	chr17:42082027-42084972	Island
hyper2229	N_excessive_3	1.1E-04	0.16	0.04	0.12	17	64962417	cg20410810	CACNG4	Body	chr17:64961007-64962321	S_Shore
hyper2230	N_excessive_3	1.3E-03	0.21	0.08	0.13	18	52495796	cg14135988	RAB27B	TSS200	chr18:52495604-52496007	Island
hyper2231	N_excessive_3	3.7E-02	0.16	0.08	0.08	19	1470810	cg13368085	APC2	3'UTR	chr19:1465206-1471241	Island
hyper2232	N_excessive_3	4.3E-06	0.26	0.08	0.18	19	5536532	cg12542584			chr19:5536531-5536789	Island
hyper2233	N_excessive_3	1.3E-02	0.34	0.07	0.27	19	5536626	cg23922259			chr19:5536531-5536789	Island
hyper2234	N_excessive_3	1.3E-05	0.31	0.07	0.24	19	10541267	cg14965524	PDE4A;PDE4A	Body;TSS1500	chr19:10542662-10543120	N_Shore
hyper2235	N_excessive_3	1.1E-04	0.17	0.02	0.14	19	10541304	cg02605007	PDE4A;PDE4A	Body;TSS1500	chr19:10542662-10543120	N_Shore
hyper2236	N_excessive_3	0.0E+00	0.37	0.00	0.36	19	10541478	cg18345115	PDE4A;PDE4A	TSS200;Body	chr19:10542662-10543120	N_Shore
hyper2237	N_excessive_3	8.9E-02	0.21	0.11	0.10	19	10542200	cg09997271	PDE4A;PDE4A;PDE4A	Body;Body;TSS1500	chr19:10542662-10543120	N_Shore
hyper2238	N_excessive_3	0.0E+00	0.30	0.04	0.26	19	10543010	cg19355182	PDE4A;PDE4A;PDE4A	Body;TSS200;Body	chr19:10542662-10543120	Island
hyper2239	N_excessive_3	2.4E-08	0.32	0.10	0.22	19	10543050	cg11863536	PDE4A;PDE4A;PDE4A	Body;TSS200;Body	chr19:10542662-10543120	Island
hyper2240	N_excessive_3	4.0E-06	0.36	0.12	0.24	19	10543144	cg05240570	PDE4A;PDE4A;PDE4A	Body;1stExon;Body	chr19:10542662-10543120	S_Shore
hyper2241	N_excessive_3	3.4E-03	0.12	0.02	0.10	19	19754386	cg20038169	GMIP;GMIP	1stExon;5'UTR	chr19:19754284-19754507	Island
hyper2242	N_excessive_3	3.9E-10	0.34	0.09	0.25	19	53073242	cg20125424	ZNF701	TSS1500	chr19:53073308-53074039	N_Shore
hyper2243	N_excessive_3	3.9E-10	0.11	0.01	0.10	20	60718612	cg11012980	PSMA7;SS18L1	TSS200;TSS1500	chr20:60717598-60719667	Island
hyper2244	N_excessive_3	4.0E-02	0.13	0.03	0.10	20	62205648	cg19131103	PRIC285	TSS200	chr20:62205585-62205887	Island
hyper2245	N_excessive_3	2.4E-04	0.12	0.02	0.11	21	45759228	cg11105585	C21orf2;C21orf2	1stExon;5'UTR	chr21:45758975-45759525	Island
hyper2246	N_excessive_4	1.3E-03	0.04	0.01	0.03	1	6453452	cg22346119	ACOT7	1stExon	chr1:6452814-6454185	Island
hyper2247	N_excessive_4	0.0E+00	0.42	0.07	0.35	1	28975167	cg00956350	RNU11	Body		
hyper2248	N_excessive_4	1.4E-09	0.37	0.08	0.29	1	152080471	cg14661959	TCHH	Body	chr1:152079997-152081705	Island
hyper2249	N_excessive_4	1.2E-04	0.38	0.05	0.33	1	152080543	cg04938315	TCHH	Body	chr1:152079997-152081705	Island
hyper2250	N_excessive_4	9.7E-10	0.44	0.04	0.40	1	152081002	cg05523911	TCHH	Body	chr1:152079997-152081705	Island
hyper2251	N_excessive_4	7.4E-09	0.07	0.01	0.06	1	160175286	cg23659592	PEA15;PEA15	5'UTR;1stExon	chr1:160175132-160175702	Island
hyper2252	N_excessive_4	1.3E-06	0.11	0.02	0.09	1	160175429	cg27510197	PEA15	5'UTR	chr1:160175132-160175702	Island
hyper2253	N_excessive_4	5.2E-03	0.15	0.08	0.07	1	160175593	cg18177682	PEA15	5'UTR	chr1:160175132-160175702	Island
hyper2254	N_excessive_4	3.8E-08	0.25	0.08	0.16	2	27319374	cg23562406	KHK;KHK	Body;Body		
hyper2255	N_excessive_4	1.8E-04	0.17	0.02	0.15	2	101767891	cg00420922	TBC1D8	TSS200	chr2:101767042-101768259	Island
hyper2256	N_excessive_4	2.0E-02	0.04	0.01	0.03	2	241938321	cg21384492	SNED1	1stExon	chr2:241937716-241938468	Island
hyper2257	N_excessive_4	5.7E-05	0.20	0.04	0.16	3	146262421	cg10679755	PLSCR1;PLSCR1	1stExon;5'UTR	chr3:146262126-146262505	Island
hyper2258	N_excessive_4	9.4E-06	0.22	0.07	0.15	4	108910565	cg08335618	HADH	TSS1500	chr4:108910770-108911370	N_Shore
hyper2259	N_excessive_4	1.9E-10	0.14	0.01	0.13	4	108910787	cg21721155	HADH	TSS200	chr4:108910770-108911370	Island
hyper2260	N_excessive_4	0.0E+00	0.23	0.01	0.21	4	108911074	cg19932974	HADH;HADH	5'UTR;1stExon	chr4:108910770-108911370	Island
hyper2261	N_excessive_4	9.7E-11	0.32	0.09	0.23	4	108911077	cg00612467	HADH;HADH	5'UTR;1stExon	chr4:108910770-108911370	Island
hyper2262	N_excessive_4	5.8E-10	0.34	0.10	0.24	4	108911347	cg07156296	HADH	Body	chr4:108910770-108911370	Island
hyper2263	N_excessive_4	0.0E+00	0.28	0.10	0.19	4	108911521	cg18523371	HADH	Body	chr4:108910770-108911370	S_Shore
hyper2264	N_excessive_4	1.9E-03	0.41	0.02	0.39	4	140216770	cg11373049	NDUFC1	Body	chr4:140216727-140217078	Island
hyper2265	N_excessive_4	1.5E-03	0.54	0.04	0.50	4	140216885	cg07830416	NDUFC1	1stExon	chr4:140216727-140217078	Island
hyper2266	N_excessive_4	5.5E-06	0.44	0.04	0.40	4	140216998	cg07723855	NDUFC1	TSS200	chr4:140216727-140217078	Island
hyper2267	N_excessive_4	0.0E+00	0.35	0.01	0.34	4	140217063	cg22127491	NDUFC1	TSS200	chr4:140216727-140217078	Island
hyper2268	N_excessive_4	1.9E-08	0.42	0.04	0.38	4	140217077	cg25491088	NDUFC1	TSS200	chr4:140216727-140217078	Island
hyper2269	N_excessive_4	2.5E-02	0.32	0.08	0.24	6	10694962	cg23811837	C6orf52;PAK1IP1;C6orf52;C6orf52	Body;TSS1500;TSS200;TSS200	chr6:10694617-10695352	Island
hyper2270	N_excessive_4	6.7E-02	0.28	0.07	0.22	6	10695044	cg22249279	C6orf52;C6orf52;PAK1IP1;C6orf52	TSS1500;TSS1500;TSS200;TSS2	chr6:10694617-10695352	Island
hyper2271	N_excessive_4	9.7E-03	0.17	0.06	0.11	6	10695274	cg16211334	C6orf52;C6orf52;C6orf52;PAK1IP1	TSS1500;TSS1500;TSS1500;1stE	chr6:10694617-10695352	Island
hyper2272	N_excessive_4	5.0E-04	0.23	0.02	0.21	6	10695336	cg09251771	C6orf52;C6orf52;C6orf52;PAK1IP1	TSS1500;TSS1500;TSS1500;Body	chr6:10694617-10695352	Island
hyper2273	N_excessive_4	6.4E-05	0.23	0.06	0.17	6	10695351	cg06645096	C6orf52;C6orf52;C6orf52;PAK1IP1	TSS1500;TSS1500;TSS1500;Body	chr6:10694617-10695352	Island
hyper2274	N_excessive_4	8.5E-02	0.28	0.07	0.21	6	10695389	cg20329376	C6orf52;C6orf52;C6orf52;PAK1IP1	TSS1500;TSS1500;TSS1500;Body	chr6:10694617-10695352	S_Shore
hyper2275	N_excessive_4	2.1E-02	0.14	0.07	0.07	6	31803718	cg12060786	C6orf48;C6orf48;SNORD52	5'UTR;5'UTR;TSS1500	chr6:31802598-31802823	S_Shore
hyper2276	N_excessive_4	2.7E-02	0.40	0.07	0.33	7	1068230	cg14085527	C7orf50;C7orf50;C7orf50	Body;Body;Body	chr7:1066931-1068245	Island
hyper2277	N_excessive_4	1.4E-02	0.22	0.05	0.17	7	1105730	cg14525935	C7orf50;C7orf50;C7orf50	Body;Body;Body	chr7:1105259-1105974	Island
hyper2278	N_excessive_4	0.0E+00	0.47	0.09	0.38	10	116852470	cg23635429	ATRNL1	TSS1500	chr10:116852261-116854094	Island
hyper2279	N_excessive_4	0.0E+00	0.39	0.05	0.34	10	116852687	cg23764381	ATRNL1	TSS1500	chr10:116852261-116854094	Island
hyper2280	N_excessive_4	0.0E+00	0.40	0.08	0.32	10	116852791	cg21921619	ATRNL1	TSS1500	chr10:116852261-116854094	Island
hyper2281	N_excessive_4	0.0E+00	0.47	0.04	0.44	10	116852865	cg05071623	ATRNL1	TSS1500	chr10:116852261-116854094	Island
hyper2282	N_excessive_4	0.0E+00	0.71	0.12	0.59	10	116853118	cg04335339	ATRNL1	TSS200	chr10:116852261-116854094	Island
hyper2283	N_excessive_4	1.8E-09	0.46	0.03	0.42	10	116853468	cg09248482	ATRNL1;ATRNL1	5'UTR;1stExon	chr10:116852261-116854094	Island
hyper2284	N_excessive_4	1.4E-03	0.07	0.03	0.05	10	126605450	cg10190329			chr10:126605120-126606212	Island
hyper2285	N_excessive_4	6.0E-06	0.14	0.02	0.12	11	27740876	cg05733135	BDNF;BDNF;BDNF;BDNF;BDNF	Body;5'UTR;5'UTR;5'UTR;5'UTR	chr11:27740644-27741236	Island
hyper2286	N_excessive_4	3.4E-04	0.07	0.02	0.05	12	110151678	cg16483426	C12orf34	TSS1500	chr12:110151327-110152758	Island
hyper2287	N_excessive_4	2.0E-02	0.14	0.04	0.11	13	30169825	cg02756849	SLC7A1	TSS200	chr13:30168901-30170246	Island
hyper2288	N_excessive_4	6.7E-02	0.13	0.05	0.08	13	38443634	cg15071251	TRPC4;TRPC4;TRPC4;TRPC4;TF	5'UTR;5'UTR;5'UTR;5'UTR;5'UTR	chr13:38443682-38444228	N_Shore
hyper2289	N_excessive_4	1.9E-02	0.12	0.07	0.05	13	78271874	cg05577945	SLAIN1	TSS1500	chr13:78271801-78272906	Island
hyper2290	N_excessive_4	2.8E-03	0.16	0.09	0.07	13	78271876	cg03117416	SLAIN1	TSS1500	chr13:78271801-78272906	Island
hyper2291	N_excessive_4	0.0E+00	0.28	0.08	0.19	14	21151223	cg25327013	ANG;RNASE4	TSS1500;TSS1500	chr14:21150918-21151224	Island
hyper2292	N_excessive_4	5.6E-02	0.13	0.07	0.06	14	69445989	cg25126812	ACTN1;ACTN1;ACTN1;ACTN1;AC	1stExon;5'UTR;1stExon;5'UTR;1st	chr14:69444639-69446826	Island
hyper2293	N_excessive_4	0.0E+00	0.47	0.06	0.42	16	77224286	cg01612187	MON1B	TSS1500	chr16:77224168-77225143	Island
hyper2294	N_excessive_4	8.1E-02	0.12	0.07	0.05	18	76635712	cg15241499			chr18:76639260-76639744	N_Shelf
hyper2295	N_excessive_4	1.4E-04	0.08	0.01	0.07	19	38755293	cg19337852	SPINT2;SPINT2;SPINT2;SPINT2	5'UTR;1stExon;5'UTR;1stExon	chr19:38754846-38755699	Island
hyper2296	N_excessive_4	1.7E-04	0.08	0.01	0.07	19	38755330	cg16655905	SPINT2;SPINT2;SPINT2;SPINT2	5'UTR;1stExon;5'UTR;1stExon	chr19:38754846-38755699	Island
hyper2297	N_excessive_4	1.7E-02	0.12	0.07	0.05	21	28216559	cg01444578	ADAMTS1	1stExon	chr21:28216558-28218117	Island
hyper2298	N_excessive_4	1.2E-07	0.17	0.05	0.12	21	28217676	cg00472814	ADAMTS1;ADAMTS1	1stExon;5'UTR	chr21:28216558-28218117	Island
hyper2299	N_excessive_4	6.5E-04	0.17	0.09	0.08	21	28217735	cg12179179	ADAMTS1	TSS200	chr21:28216558-28218117	Island
hyper2300	N_excessive_5	3.7E-04	0.22	0.04	0.19	1	6673306	cg10949451	PHF13	TSS1500	chr1:6672786-6675741	Island
hyper2301	N_excessive_5	3.5E-09	0.48	0.06	0.41	1	10057158	cg18086187	RBP7	TSS200	chr1:10057121-10058108	Island
hyper2302	N_excessive_5	0.0E+00	0.40	0.01	0.39	1	10057303	cg23107691	RBP7;RBP7	1stExon;5'UTR	chr1:10057121-10058108	Island
hyper2303	N_excessive_5	0.0E+00	0.39	0.02	0.37	1	10057305	cg25397076	RBP7;RBP7	1stExon;5'UTR	chr1:10057121-10058108	Island
hyper2304	N_excessive_5	0.0E+00	0.40	0.02	0.38	1	10057312	cg27561954	RBP7;RBP7	1stExon;5'UTR	chr1:10057121-10058108	Island
hyper2305	N_excessive_5	0.0E+00	0.41	0.08	0.33	1	24105262	cg24044651	C1orf128	Body	chr1:24104502-24105263	Island
hyper2306	N_excessive_5	2.7E-03	0.23	0.04	0.20	1	31158158	cg17484671			chr1:31158009-31158261	Island
hyper2307	N_excessive_5	2.3E-03	0.06	0.01	0.05	1	39957298	cg08748615	BMP8A	TSS200	chr1:39956424-39958137	Island
hyper2308	N_excessive_5	7.9E-02	0.13	0.06	0.07	1	72749015	cg01425670	NEGR1	TSS1500	chr1:72748471-72749736	Island
hyper2309	N_excessive_5	2.1E-02	0.17	0.05	0.11	1	72750124	cg21774281			chr1:72748471-72749736	S_Shore
hyper2310	N_excessive_5	8.4E-02	0.28	0.08	0.20	1	98519504	cg21525592			chr1:98519001-98519983	Island
hyper2311	N_excessive_5	3.8E-02	0.28	0.04	0.24	1	145473631	cg10178263	ANKRD34A	Body	chr1:145472980-145474702	Island
hyper2312	N_excessive_5	9.7E-05	0.09	0.02	0.08	1	145473698	cg18280463	ANKRD34A	Body	chr1:145472980-145474702	Island
hyper2313	N_excessive_5	1.8E-02	0.18	0.06	0.12	1	145473700	cg01229562	ANKRD34A	Body	chr1:145472980-145474702	Island
hyper2314	N_excessive_5	2.1E-03	0.04	0.01	0.04	1	200992656	cg26228351	KIF21B;KIF21B	5'UTR;1stExon	chr1:200992282-200992839	Island
hyper2315	N_excessive_5	5.8E-10	0.48	0.08	0.40	1	206785415	cg10662787	LGTN	Body	chr1:206785804-206786030	N_Shore
hyper2316	N_excessive_5	2.5E-08	0.52	0.08	0.44	1	206785660	cg07998070	LGTN	1stExon	chr1:206785804-206786030	N_Shore
hyper2317	N_excessive_5	1.9E-07	0.64	0.08	0.55	1	206786170	cg21155316	LGTN	TSS1500	chr1:206785804-206786030	S_Shore

hyper2318	N_excessive_5	1.2E-07	0.61	0.10	0.51	1	206786174	cg16966520	LGTN	TSS1500	chr1:206785804-206786030	S_Shore
hyper2319	N_excessive_5	1.5E-06	0.66	0.15	0.52	1	206786178	cg16639185	LGTN	TSS1500	chr1:206785804-206786030	S_Shore
hyper2320	N_excessive_5	8.8E-07	0.70	0.14	0.56	1	206786180	cg21936179	LGTN	TSS1500	chr1:206785804-206786030	S_Shore
hyper2321	N_excessive_5	2.2E-05	0.36	0.03	0.32	1	213124896	cg04907523	VASH2;VASH2;VASH2	Body;5'UTR;5'UTR	chr1:213123647-213125092	Island
hyper2322	N_excessive_5	3.7E-05	0.19	0.06	0.12	1	218338772	cg11537367			chr1:218337780-218339033	Island
hyper2323	N_excessive_5	3.0E-08	0.05	0.00	0.05	2	9614578	cg05986044	IAH1	TSS200	chr2:9614194-9615235	Island
hyper2324	N_excessive_5	1.1E-03	0.22	0.08	0.14	2	17721764	cg18735601	VSNL1	TSS200	chr2:17721537-17722021	Island
hyper2325	N_excessive_5	1.6E-02	0.21	0.06	0.15	2	17721988	cg01307387	VSNL1;VSNL1	1stExon;5'UTR	chr2:17721537-17722021	Island
hyper2326	N_excessive_5	5.6E-04	0.29	0.02	0.27	2	20101779	cg26693529	TTC32	TSS200	chr2:20101004-20102067	Island
hyper2327	N_excessive_5	3.2E-03	0.21	0.05	0.16	2	20102066	cg17420218	TTC32	TSS1500	chr2:20101004-20102067	Island
hyper2328	N_excessive_5	2.2E-07	0.21	0.07	0.14	2	44395004	cg04920474	PPM1B;PPM1B;PPM1B;PPM1B;F	TSS1500;TSS1500;TSS1500;TSS	chr2:44394983-44396629	Island
hyper2329	N_excessive_5	5.7E-06	0.36	0.11	0.25	2	71115994	cg08793877			chr2:71115927-71116412	Island
hyper2330	N_excessive_5	3.6E-03	0.07	0.02	0.05	2	121070622	cg24070213				
hyper2331	N_excessive_5	2.1E-02	0.16	0.06	0.10	2	131099506	cg20670292	IMP4;CCDC115	TSS1500;Body	chr2:131099469-131100863	Island
hyper2332	N_excessive_5	3.8E-06	0.38	0.04	0.34	2	142887886	cg20443778	LRP1B	Body	chr2:142887724-142888553	Island
hyper2333	N_excessive_5	1.1E-02	0.39	0.11	0.28	2	142888052	cg10634922	LRP1B	Body	chr2:142887724-142888553	Island
hyper2334	N_excessive_5	1.5E-06	0.24	0.07	0.17	2	142888598	cg02322989	LRP1B;LRP1B	5'UTR;1stExon	chr2:142887724-142888553	S_Shore
hyper2335	N_excessive_5	3.2E-05	0.07	0.02	0.05	2	154335184	cg17942639	RPRM;RPRM	1stExon;5'UTR	chr2:154334449-154335458	Island
hyper2336	N_excessive_5	1.9E-04	0.24	0.05	0.19	2	162094118	cg21211367			chr2:162094460-162096195	N_Shore
hyper2337	N_excessive_5	4.6E-06	0.14	0.03	0.11	2	162094696	cg00322319			chr2:162094460-162096195	Island
hyper2338	N_excessive_5	4.4E-06	0.20	0.06	0.15	2	162094836	cg09064687			chr2:162094460-162096195	Island
hyper2339	N_excessive_5	1.3E-09	0.26	0.02	0.24	2	162094867	cg26915774			chr2:162094460-162096195	Island
hyper2340	N_excessive_5	2.0E-03	0.24	0.06	0.18	2	171674437	cg01763173	GAD1;GAD1	5'UTR;5'UTR	chr2:171672310-171675447	Island
hyper2341	N_excessive_5	3.9E-07	0.20	0.06	0.13	2	172951501	cg16347858	DLX1;DLX1	Body;Body	chr2:172951289-172952159	Island
hyper2342	N_excessive_5	5.6E-06	0.19	0.03	0.16	2	176046185	cg16884400	ATP5G3;ATP5G3;ATP5G3	5'UTR;1stExon;5'UTR	chr2:176045969-176046504	Island
hyper2343	N_excessive_5	6.5E-03	0.13	0.06	0.07	2	176046495	cg19713083	ATP5G3;ATP5G3	TSS200;TSS200	chr2:176045969-176046504	Island
hyper2344	N_excessive_5	6.5E-02	0.14	0.06	0.08	2	176867409	cg23322292	KIAA1715	TSS1500	chr2:176866693-176867188	S_Shore
hyper2345	N_excessive_5	9.0E-03	0.09269903	0.02	0.07	2	192110021	cg04692993	MYO1B;MYO1B;MYO1B	TSS200;TSS1500;TSS200	chr2:192109828-192110940	Island
hyper2346	N_excessive_5	4.2E-02	0.2548827	0.07	0.18	2	230933543	cg16273133	SLC16A14;SLC16A14	1stExon;5'UTR	chr2:230932468-230933550	Island
hyper2347	N_excessive_5	4.2E-06	0.1449195	0.04	0.10	3	8543014	cg20595215	LMCD1	TSS1500	chr3:8543201-8543579	N_Shore
hyper2348	N_excessive_5	2.5E-02	0.3731982	0.08	0.30	3	44754398	cg04974323	ZNF502;ZNF502;ZNF502;ZNF502	5'UTR;5'UTR;5'UTR;5'UTR	chr3:44754099-44754399	Island
hyper2349	N_excessive_5	2.6E-02	0.0830604	0.02	0.06	3	50242735	cg16468914	SLC38A3;SLC38A3	1stExon;5'UTR	chr3:50242550-50243589	Island
hyper2350	N_excessive_5	3.9E-02	0.3980304	0.17	0.23	3	109128982	cg20382831	FLJ25363;FLJ25363	1stExon;5'UTR		
hyper2351	N_excessive_5	2.3E-04	0.1539849	0.02	0.13	3	142837995	cg19442495	CHST2	TSS1500	chr3:142837885-142840838	Island
hyper2352	N_excessive_5	4.5E-08	0.6302117	0.22	0.41	3	170408349	cg01092546				
hyper2353	N_excessive_5	6.7E-03	0.2839854	0.02	0.26	3	192959176	cg07068191	HRASLS;HRASLS;MGC2889	1stExon;5'UTR;TSS1500	chr3:192958709-192959373	Island
hyper2354	N_excessive_5	6.5E-03	0.322541	0.03	0.29	3	196366437	cg08026502	LRRC33	TSS1500	chr3:196366394-196366919	Island
hyper2355	N_excessive_5	4.1E-02	0.37211	0.14	0.24	4	30720741	cg01532230	PCDH7;PCDH7;PCDH7	TSS1500;TSS1500;TSS1500	chr4:30721204-30724842	N_Shore
hyper2356	N_excessive_5	5.9E-06	0.09533128	0.01	0.08	4	44728642	cg09662896	GNPDA2	TSS200	chr4:44728033-44728719	Island
hyper2357	N_excessive_5	1.5E-05	0.1430921	0.02	0.13	4	44728647	cg25512518	GNPDA2	TSS200	chr4:44728033-44728719	Island
hyper2358	N_excessive_5	2.5E-03	0.1025355	0.04	0.07	4	90031969	cg17656165			chr4:90031904-90033005	Island
hyper2359	N_excessive_5	1.0E-02	0.3039339	0.02	0.29	4	113437909	cg10871779	NEUROG2	TSS1500	chr4:113435898-113438002	Island
hyper2360	N_excessive_5	2.1E-06	0.1784283	0.06	0.12	4	129474594	cg07174665				
hyper2361	N_excessive_5	2.3E-02	0.2049494	0.04	0.16	4	156874415	cg10592446	CTSO	Body	chr4:156874740-156875083	N_Shore
hyper2362	N_excessive_5	7.9E-03	0.3603713	0.06	0.30	4	156874602	cg11754095	CTSO	Body	chr4:156874740-156875083	N_Shore
hyper2363	N_excessive_5	6.3E-04	0.2381632	0.09	0.15	4	180979522	cg21229339			chr4:180979038-180980619	Island
hyper2364	N_excessive_5	1.6E-05	0.1827952	0.05	0.13	4	187646301	cg18894054	FAT1	TSS1500	chr4:187644319-187648253	Island
hyper2365	N_excessive_5	6.7E-07	0.2982114	0.09	0.21	4	187647344	cg22501604			chr4:187644319-187648253	Island
hyper2366	N_excessive_5	4.5E-06	0.1669849	0.03	0.14	4	187647402	cg20274950			chr4:187644319-187648253	Island
hyper2367	N_excessive_5	4.9E-02	0.2716151	0.12	0.15	5	272203	cg21748578	PDCD6	Body	chr5:272139-272485	Island
hyper2368	N_excessive_5	2.4E-02	0.1278426	0.05	0.07	5	1633830	cg18250340	LOC728613	Body	chr5:1633901-1634224	N_Shore
hyper2369	N_excessive_5	1.0E-03	0.394485	0.11	0.29	5	1875058	cg06654549			chr5:1874907-1879032	Island
hyper2370	N_excessive_5	4.0E-03	0.2432682	0.02	0.22	5	50679151	cg11039614	ISL1;ISL1	5'UTR;1stExon	chr5:50678632-50679375	Island
hyper2371	N_excessive_5	3.0E-05	0.1745401	0.05	0.12	5	54516372	cg15335436			chr5:54516267-54516919	Island
hyper2372	N_excessive_5	4.0E-02	0.1477816	0.04	0.11	5	54516879	cg14608384			chr5:54516267-54516919	Island
hyper2373	N_excessive_5	9.6E-03	0.3335182	0.13	0.20	5	60921535	cg04569837			chr5:60921534-60922472	Island
hyper2374	N_excessive_5	4.6E-02	0.2951182	0.10	0.20	5	63461930	cg10372047	RNF180;RNF180	5'UTR;5'UTR	chr5:63461448-63462106	Island
hyper2375	N_excessive_5	5.8E-02	0.2431545	0.09	0.16	5	113697632	cg23615676	KCNN2	TSS1500	chr5:113696516-113699195	Island
hyper2376	N_excessive_5	2.2E-03	0.3856588	0.04	0.34	5	114515936	cg10569615	TRIM36;TRIM36;TRIM36;TRIM36;	1stExon;1stExon;1stExon;5'UTR;5	chr5:114514716-114516220	Island
hyper2377	N_excessive_5	2.1E-04	0.1663377	0.03	0.13	5	114516141	cg09463882	TRIM36;TRIM36;TRIM36;TRIM36;	5'UTR;1stExon;1stExon;5'UTR;5	chr5:114514716-114516220	Island
hyper2378	N_excessive_5	1.4E-04	0.2375454	0.04	0.20	5	114516315	cg04450862	TRIM36;TRIM36;TRIM36	TSS200;TSS200;TSS200	chr5:114514716-114516220	S_Shore
hyper2379	N_excessive_5	4.4E-04	0.3764512	0.06	0.32	5	128796168	cg15034135	ADAMTS19	1stExon	chr5:128795503-128797417	Island
hyper2380	N_excessive_5	2.7E-02	0.3254394	0.09	0.23	5	128796266	cg25309017	ADAMTS19	Body	chr5:128795503-128797417	Island
hyper2381	N_excessive_5	1.9E-03	0.2215271	0.09	0.14	5	146833171	cg20564764	DPYSL3;DPYSL3	5'UTR;1stExon	chr5:146832560-146833938	Island
hyper2382	N_excessive_5	7.5E-03	0.4342609	0.10	0.33	5	178368123	cg03234732	ZNF454	TSS200	chr5:178367620-178368725	Island
hyper2383	N_excessive_5	4.5E-08	0.4941208	0.02	0.47	6	27064769	cg11183365			chr6:27064628-27065004	Island
hyper2384	N_excessive_5	2.9E-10	0.4338505	0.03	0.41	6	27064968	cg22058855			chr6:27064628-27065004	Island
hyper2385	N_excessive_5	1.0E-07	0.3363522	0.05	0.29	6	27065003	cg12298212			chr6:27064628-27065004	Island
hyper2386	N_excessive_5	1.9E-07	0.2929942	0.08	0.21	6	39760416	cg14135551	DAAM2	TSS1500	chr6:39760066-39760426	Island
hyper2387	N_excessive_5	7.8E-02	0.3056509	0.05	0.26	6	42946962	cg04457536	PEX6;PEX6	1stExon;5'UTR	chr6:42946310-42947127	Island
hyper2388	N_excessive_5	6.3E-03	0.1780632	0.05	0.13	6	42947056	cg26304593	PEX6	TSS200	chr6:42946310-42947127	Island
hyper2389	N_excessive_5	1.2E-03	0.4510594	0.26	0.19	6	87832220	cg08192165			chr6:87832219-87832610	Island
hyper2390	N_excessive_5	2.6E-08	0.4899381	0.10	0.39	6	87832609	cg18625538			chr6:87832219-87832610	Island
hyper2391	N_excessive_5	4.9E-05	0.4135285	0.10	0.31	6	87832666	cg13164087			chr6:87832219-87832610	S_Shore
hyper2392	N_excessive_5	9.0E-03	0.3094874	0.10	0.21	6	88875622	cg26556436	CNR1;CNR1;CNR1;CNR1;CNR1;	5'UTR;5'UTR;1stExon;1stExon;5'U	chr6:88875397-88877449	Island
hyper2393	N_excessive_5	1.7E-08	0.2795798	0.04	0.24	6	88875836	cg07251193	CNR1;CNR1;CNR1;CNR1	TSS200;TSS200;TSS200;TSS200	chr6:88875397-88877449	Island
hyper2394	N_excessive_5	1.1E-07	0.3570535	0.03	0.33	6	88875840	cg20622089	CNR1;CNR1;CNR1;CNR1	TSS200;TSS200;TSS200;TSS200	chr6:88875397-88877449	Island
hyper2395	N_excessive_5	4.4E-04	0.1834499	0.08	0.10	6	100061504	cg08933939	PRDM13	Body	chr6:100060979-100062657	Island
hyper2396	N_excessive_5	1.5E-07	0.1526267	0.02	0.13	7	5633086	cg22447539	FSCN1	1stExon	chr7:5632335-5634555	Island
hyper2397	N_excessive_5	8.2E-03	0.432141	0.13	0.30	7	27213966	cg01397139	HOXA10;HOXA10	TSS200;Body	chr7:27212416-27214396	Island
hyper2398	N_excessive_5	2.6E-03	0.07853214	0.03	0.05	7	29724905	cg12042055	LOC646762	TSS200	chr7:29724188-29725436	Island
hyper2399	N_excessive_5	8.3E-02	0.1697295	0.10	0.07	7	35293884	cg23602974	TBX20;TBX20	TSS200;TSS200	chr7:35292985-35294674	Island
hyper2400	N_excessive_5	8.0E-04	0.115034	0.02	0.09	7	43965618	cg13088374	URGCP;UBE2D4	5'UTR;TSS1500	chr7:43965806-43966400	N_Shore
hyper2401	N_excessive_5	9.2E-02	0.1461122	0.06	0.09	7	43965691	cg08084737	URGCP;UBE2D4	5'UTR;TSS1500	chr7:43965806-43966400	N_Shore
hyper2402	N_excessive_5	4.4E-02	0.2199683	0.07	0.15	7	119912723	cg25241964	KCND2	TSS1500	chr7:119912840-119913883	N_Shore
hyper2403	N_excessive_5	1.1E-07	0.2184149	0.06	0.15	8	13424419	cg23089838	C8orf48;C8orf48	1stExon;5'UTR		
hyper2404	N_excessive_5	0.0E+00	0.5578743	0.02	0.53	8	27491209	cg04264299	SCARA3;SCARA3	TSS1500;TSS1500	chr8:27490959-27491775	Island
hyper2405	N_excessive_5	0.0E+00	0.4157137	0.04	0.38	8	27491257	cg10605130	SCARA3;SCARA3	TSS1500;TSS1500	chr8:27490959-27491775	Island
hyper2406	N_excessive_5	0.0E+00	0.3764749	0.10	0.28	8	27491259	cg03891330	SCARA3;SCARA3	TSS1500;TSS1500	chr8:27490959-27491775	Island
hyper2407	N_excessive_5	3.0E-00	0.4883233	0.12	0.37	8	27491676	cg22951989	SCARA3;SCARA3;SCARA3;SCAI	5'UTR;1stExon;1stExon;5'UTR	chr8:27490959-27491775	Island
hyper2408	N_excessive_5	9.1E-02	0.4752111	0.34	0.14	8	27492077	cg26847866	SCARA3;SCARA3	Body;Body	chr8:27490959-27491775	S_Shore
hyper2409	N_excessive_5	1.0E-03	0.161837	0.04	0.12	8	123793590	cg21008098	ZHX2	TSS1500	chr8:123792369-123794942	Island
hyper2410	N_excessive_5	2.0E-05	0.2649547	0.08	0.19	8	123793727	cg17793819	ZHX2	TSS200	chr8:123792369-123794942	Island
hyper2411	N_excessive_5	2.4E-02	0.218405	0.06	0.16	8	123793823	cg01448923	ZHX2	TSS200	chr8:123792369-123794942	Island
hyper2412	N_excessive_5	3.1E-02	0.122251	0.05	0.08	8	123793830	cg08886760	ZHX2	TSS200	chr8:123792369-123794942	Island
hyper2413	N_excessive_5	5.8E-03	0.1751468	0.07	0.11	8	123793836	cg01812826	ZHX2	TSS200	chr8:123792369-123794942	Island
hyper2414	N_excessive_5	2.5E-04	0.3179697	0.09	0.23	8	123793873	cg23181591	ZHX2	TSS200	chr8:123792369-123794942	Island
hyper2415	N_excessive_5	9.9E-07	0.2688099	0.07	0.20	8	123793925	cg13935507	ZHX2;ZHX2	5'UTR;1stExon	chr8:123792369-123794942	Island
hyper2416	N_excessive_5	2.3E-05	0.1655992	0.05	0.12	8	123794166	cg03796881	ZHX2;ZHX2	5'UTR;1stExon	chr8:123792369-123794942	Island
hyper2417	N_excessive_5	2.9E-04	0.1835627	0.03	0.16	8	125463682	cg00846483	TRMT12	1stExon		
hyper2418	N_excessive_5	1.5E-02	0.2459897	0.11	0.13	9	38621011	cg08577743	C9orf122;ANKRD18A	TSS200;TSS1500	chr9:38619908-38622631	Island
hyper2419	N_excessive_5	7.4E-02	0.09051349	0.04	0.05	9	86322725	cg14374504	UBQLN1;UBQLN1;UBQLN1;UBQ	5'UTR;1stExon;1stExon;5'UTR	chr9:86322017-86323215	Island
hyper2420	N_excessive_5	5.7E-03	0.3275436	0.07	0.25	9	118916493	cg02873524	PAPPA	1stExon	chr9:118916362-118917175	Island
hyper2421	N_excessive_5	9.0E-06	0.1887442	0.09	0.10	10	122619	cg25993111			chr10:122102-122620	Island
hyper2422	N_excessive_5	7.4E-02	0.2826293	0.09	0.19	10	13043887	cg13423980	CCDC3	TSS200	chr10:13043094-13043781	S_Shore
hyper2423	N_excessive_5	0.0E+00	0.3322406	0.01	0.32	10	21783855	cg22889755	C10orf114	3'UTR	chr10:21783198-21786420	Island
hyper2424	N_excessive_5	7.8E-03	0.1703164	0.05	0.12	10	21783930	cg05385047	C10orf114	3'UTR	chr10:21783198-21786420	Island
hyper2425	N_excessive_5	1.4E-03	0.1284767	0.05	0.08	10	35929604	cg15166332	FZD8	1stExon	chr10:35928501-35931893	Island
hyper2426	N_excessive_5	1.2E-04	0.2489427	0.10	0.14	10	43249161	cg04799489			chr10:43248523-43250912	Island
hyper2427	N_excessive_5	1.5E-02	0.1876701	0.04	0.14	10	43250569	cg16325777			chr10:43248523-43250912	Island
hyper2428	N_excessive_5	5.7E-03	0.2504539	0.11	0.14	10	52751764	cg00099869	PRKG1	Body	chr10:52750956-52751765	Island
hyper2429	N_excessive_5	0.0E+00	0.2411534	0.02	0.23	10	81741554	cg00205263			chr10:81741228-81742293	Island
hyper2430	N_excessive_5	3.6E-06	0.1737274	0.05	0.12	10	81741832	cg18762849			chr10:81741228-81742293	Island
hyper2431	N_excessive_5	7.8E-02	0.4949253	0.13	0.37	10	104629122	cg00086670	AS3MT	TSS200	chr10:104629038-104629479	Island
hyper2432	N_excessive_5	5.2E-02	0.4985968	0.09	0.41	10	104629142	cg08856941	AS3MT	TSS200	chr10:104629038-104629479	Island
hyper2433	N_excessive_5	8.5E-03	0.4942849	0.04	0.46	10	104629151	cg24592962	AS3MT	TSS200	chr10:104629038-104629479	Island
hyper2434	N_excessive_5	1.9E-04	0.2844322	0.05	0.24	10	118896776	cg27533288	VAX1;VAX1	Body;Body	chr10:118895963-118898037	Island
hyper2435	N_excessive_5	1.3E-08	0.5732605	0.13	0.44	10	118897194	cg21242918	VAX1;VAX1	Body;Body	chr10:118895963-118898037	Island
hyper2436	N_excessive_5	3.0E-02	0.191571	0.04	0.15	10	124896232	cg03453917	HMX3	Body	chr10:124894075-124897366	Island
hyper2437	N_excessive_5	1.2E-06	0.3378965	0.05	0.29	11	2905931	cg22865058	CDKN1C;CDKN1C;CDKN1C	Body;1stExon;Body	chr11:2905023-2907024	Island
hyper2438	N_excessive_5	1.1E-05	0.3323525	0.05	0.28	11	2906285	cg04548378	CDKN1C;CDKN1C;CDKN1C	Body;1stExon;Body	chr11:2905023-2907024	Island
hyper2439	N_excessive_5	3.8E-02	0.3021553	0.04	0.26	11	2907008	cg14598198	CDKN1C;CDKN1C;CDKN1C	TSS200;TSS200;TSS200	chr11:2905023-2907024	Island
hyper2440	N_excessive_5	4.4E-02	0.3269416	0.04	0.29	11	2907057	cg01730944	CDKN1C;CDKN1C;CDKN1C	TSS200;TSS200;TSS200	chr11:2907308-2907675	N_Shore
hyper2441	N_excessive_5	1.8E-02	0.4590865	0.05	0.41	11	2907071	cg26718354	CDKN1C;CDKN1C;CDKN1C	TSS200;TSS200;TSS200	chr11:2907308-2907675	N_Shore
hyper2442	N_excessive_5	4.6E-02	0.04622454	0.01	0.04	11	2907337	cg06191719	CDKN1C;CDKN1C;CDKN1C	TSS1500;TSS1500;TSS1500	chr11:2907308-2907675	Island
hyper2443	N_excessive_5	3.0E-02	0.165471	0.06	0.11	11	2950620	cg18140683	PHLDA2;PHLDA2	5'UTR;1stExon	chr11:2949788-2951756	Island
hyper2444	N_excessive_5	1.1E-03	0.1701033	0.02	0.15	11	2950632	cg01505590	PHLDA2;PHLDA2	5'UTR;1stExon	chr11:2949788-2951756	Island
hyper2445	N_excessive_5	1.7E-05	0.2365835	0.06	0.18	11	2951046	cg18175880	PHLDA2	TSS1500	chr11:2949788-2951756	Island
hyper2446	N_excessive_5	1.6E-06	0.4910795	0.03	0.47	11	13689489	cg23790345	FAR1	TSS1500	chr11:13689588-13690724	N_Shore

hyper2447	N_excessive_5	4.1E-02	0.5226871	0.03	0.49	11	13690702	cg04012924	FAR1	5'UTR	chr11:13689588-13690724	Island
hyper2448	N_excessive_5	1.6E-05	0.3570966	0.10	0.25	11	24518737	cg06931933	LUZP2;LUZP2	5'UTR;1stExon	chr11:24518224-24518738	Island
hyper2449	N_excessive_5	3.2E-03	0.2827678	0.02	0.27	11	31831974	cg18270629	PAX6;PAX6;PAX6	5'UTR;5'UTR;5'UTR	chr11:31831620-31839038	Island
hyper2450	N_excessive_5	1.3E-04	0.2192658	0.03	0.19	11	31832246	cg26848086	PAX6;PAX6;PAX6	5'UTR;5'UTR;5'UTR	chr11:31831620-31839038	Island
hyper2451	N_excessive_5	3.3E-03	0.09628962	0.03	0.07	11	32112632	cg03576787	RCN1;RCN1	5'UTR;1stExon	chr11:32112394-32113277	Island
hyper2452	N_excessive_5	4.5E-02	0.1013444	0.03	0.07	11	32112634	cg04677061	RCN1;RCN1	5'UTR;1stExon	chr11:32112394-32113277	Island
hyper2453	N_excessive_5	2.9E-03	0.4632607	0.06	0.40	11	44331108	cg24335138	ALX4	Body	chr11:44330542-44331761	Island
hyper2454	N_excessive_5	1.2E-07	0.07647201	0.01	0.07	11	62105152	cg13799206	ASRGL1;ASRGL1	5'UTR;5'UTR	chr11:62104779-62105636	Island
hyper2455	N_excessive_5	0.0E+00	0.534848	0.03	0.50	11	63438716	cg14589563	ATL3	Body	chr11:63438301-63439314	Island
hyper2456	N_excessive_5	3.0E-03	0.2878025	0.07	0.22	11	63438816	cg27568631	ATL3;ATL3	5'UTR;1stExon	chr11:63438301-63439314	Island
hyper2457	N_excessive_5	2.6E-04	0.05754682	0.01	0.05	11	63439157	cg02761940	ATL3	TSS200	chr11:63438301-63439314	Island
hyper2458	N_excessive_5	3.3E-03	0.102944	0.04	0.07	11	63439207	cg27042081	ATL3	TSS200	chr11:63438301-63439314	Island
hyper2459	N_excessive_5	3.6E-02	0.3285916	0.01	0.32	11	66336125	cg17381565	CTSF	TSS200	chr11:66335575-66336151	Island
hyper2460	N_excessive_5	4.7E-09	0.4028866	0.07	0.33	11	94884121	cg22049858			chr11:94883647-94884811	Island
hyper2461	N_excessive_5	6.4E-02	0.3171716	0.11	0.21	11	124736661	cg05086956	ROBO3	Body	chr11:124735069-124736465	S_Shore
hyper2462	N_excessive_5	3.5E-03	0.1544504	0.09	0.07	11	125365803	cg06309855	FEZ1;FEZ1	5'UTR;5'UTR	chr11:125365137-125366319	Island
hyper2463	N_excessive_5	1.4E-02	0.1349568	0.05	0.08	12	58027199	cg24905909	B4GALNT1	TSS1500	chr12:58025661-58027056	S_Shore
hyper2464	N_excessive_5	5.3E-02	0.1880773	0.08	0.11	12	94542156	cg13488680	PLXNC1	TSS1500	chr12:94541727-94544000	Island
hyper2465	N_excessive_5	9.1E-02	0.07511911	0.02	0.06	12	121904973	cg15340887	KDM2B;KDM2B	Body;Body	chr12:121905463-121905956	N_Shore
hyper2466	N_excessive_5	1.6E-06	0.322732	0.07	0.26	12	124246391	cg06745944	DNAH10	TSS1500	chr12:124246524-124247254	N_Shore
hyper2467	N_excessive_5	1.5E-02	0.4567332	0.17	0.29	12	124246434	cg27125645	DNAH10	TSS1500	chr12:124246524-124247254	N_Shore
hyper2468	N_excessive_5	3.6E-06	0.3757852	0.04	0.34	12	124246446	cg10575376	DNAH10	TSS1500	chr12:124246524-124247254	N_Shore
hyper2469	N_excessive_5	1.8E-04	0.3378074	0.07	0.27	12	124246525	cg26986180	DNAH10	TSS1500	chr12:124246524-124247254	Island
hyper2470	N_excessive_5	4.8E-02	0.5462338	0.12	0.42	12	124247047	cg01240444	DNAH10;DNAH10	1stExon;5'UTR	chr12:124246524-124247254	Island
hyper2471	N_excessive_5	4.6E-03	0.4382882	0.07	0.37	12	124247223	cg11026555	DNAH10	Body	chr12:124246524-124247254	Island
hyper2472	N_excessive_5	2.9E-03	0.03126758	0.00	0.03	13	20532920	cg21004366	ZMYM2;ZMYM2;ZMYM2;ZMYM2	1stExon;5'UTR;5'UTR;1stExon	chr13:20531548-20533630	Island
hyper2473	N_excessive_5	3.2E-02	0.6270647	0.41	0.21	13	33002597	cg19907326	N4BP2L1;N4BP2L1	TSS1500;TSS1500	chr13:33001249-33002078	S_Shore
hyper2474	N_excessive_5	7.0E-02	0.1672384	0.04	0.13	13	96743895	cg27513979	HS6ST3	Body	chr13:96742138-96744162	Island
hyper2475	N_excessive_5	7.9E-04	0.3528457	0.08	0.27	14	27066771	cg15602241	NOVA1;NOVA1;NOVA1;NOVA1;N	5'UTR;5'UTR;5'UTR;1stExon;1stE	chr14:27066313-27066578	S_Shore
hyper2476	N_excessive_5	2.8E-03	0.3130897	0.08	0.23	14	60097124	cg19490868	RTN1;RTN1;RTN1	Body;Body;Body	chr14:60097208-60097553	N_Shore
hyper2477	N_excessive_5	3.5E-03	0.3025842	0.08	0.23	14	60097209	cg05565239	RTN1;RTN1;RTN1	Body;Body;1stExon	chr14:60097208-60097553	Island
hyper2478	N_excessive_5	3.1E-06	0.1833213	0.09	0.10	14	71288541	cg12919976			chr14:71288403-71288957	Island
hyper2479	N_excessive_5	0.0E+00	0.3638572	0.02	0.35	14	77422934	cg00999865			chr14:77422842-77423094	Island
hyper2480	N_excessive_5	1.2E-05	0.3124688	0.10	0.22	14	77423080	cg18366782			chr14:77422842-77423094	Island
hyper2481	N_excessive_5	0.0E+00	0.2817187	0.07	0.21	14	77423093	cg25555279			chr14:77422842-77423094	Island
hyper2482	N_excessive_5	2.9E-07	0.2139657	0.08	0.14	14	77423234	cg14260355			chr14:77422842-77423094	S_Shore
hyper2483	N_excessive_5	2.4E-03	0.08729123	0.03	0.06	15	42565760	cg27313857	GANC;TMEM87A;TMEM87A	TSS1500;TSS200;TSS200	chr15:42565452-42565791	Island
hyper2484	N_excessive_5	7.7E-06	0.1738459	0.03	0.14	15	61520001	cg04132917	RORA	Body	chr15:61519621-61520031	Island
hyper2485	N_excessive_5	6.0E-08	0.4490493	0.10	0.35	15	61520931	cg18136062	RORA	Body	chr15:61520423-61521716	Island
hyper2486	N_excessive_5	5.4E-03	0.3362514	0.09	0.25	15	61521000	cg09112687	RORA	Body	chr15:61520423-61521716	Island
hyper2487	N_excessive_5	2.9E-08	0.3144593	0.08	0.23	16	3415077	cg09999114			chr16:3414964-3415167	Island
hyper2488	N_excessive_5	8.3E-04	0.2037538	0.07	0.14	16	3415256	cg02898051			chr16:3414964-3415167	S_Shore
hyper2489	N_excessive_5	6.9E-04	0.1955134	0.09	0.11	16	30194484	cg06030619	CORO1A	TSS1500	chr16:30194747-30195357	N_Shore
hyper2490	N_excessive_5	2.4E-04	0.2374803	0.04	0.20	16	79634723	cg02962380	MAF;MAF	TSS200;TSS200	chr16:79632619-79635537	Island
hyper2491	N_excessive_5	1.7E-03	0.2770669	0.04	0.24	16	84002370	cg02070685	NECAB2	1stExon	chr16:84002269-84002860	Island
hyper2492	N_excessive_5	7.2E-04	0.3457963	0.11	0.24	16	84002599	cg02899772	NECAB2	Body	chr16:84002269-84002860	Island
hyper2493	N_excessive_5	3.0E-04	0.1223969	0.02	0.10	17	8925203	cg07616990	NTN1	5'UTR	chr17:8924167-8926920	Island
hyper2494	N_excessive_5	4.9E-03	0.1575761	0.03	0.13	17	15244482	cg22264930	TEKT3	5'UTR	chr17:15244705-15245126	N_Shore
hyper2495	N_excessive_5	1.6E-09	0.3591559	0.05	0.31	17	36102740	cg03433642	HNF1B;HNF1B	Body;Body	chr17:36102033-36104766	Island
hyper2496	N_excessive_5	6.4E-03	0.2660848	0.05	0.22	17	66286791	cg07407787	ARSG;SLC16A6	5'UTR;5'UTR	chr17:66286541-66288532	Island
hyper2497	N_excessive_5	2.6E-03	0.07689697	0.02	0.05	17	78010476	cg07441156	CCDC40;TBC1D16	1stExon;TSS1500	chr17:78009076-78011084	Island
hyper2498	N_excessive_5	4.4E-05	0.05211311	0.01	0.05	18	8706720	cg19669959			chr18:8704721-8707557	Island
hyper2499	N_excessive_5	6.6E-05	0.1645087	0.08	0.08	18	20139442	cg07114353			chr18:20139484-20140246	N_Shore
hyper2500	N_excessive_5	1.2E-04	0.1597035	0.07	0.09	18	20139642	cg12429953			chr18:20139484-20140246	Island
hyper2501	N_excessive_5	5.2E-05	0.1740218	0.06	0.11	18	20139875	cg01676182			chr18:20139484-20140246	Island
hyper2502	N_excessive_5	2.5E-08	0.2212508	0.08	0.14	18	20139913	cg05760402			chr18:20139484-20140246	Island
hyper2503	N_excessive_5	9.9E-08	0.6008995	0.03	0.57	18	47087108	cg20486897	LIPG	TSS1500	chr18:47087075-47087446	Island
hyper2504	N_excessive_5	3.7E-05	0.6212655	0.07	0.55	18	47087215	cg07277041	LIPG	TSS1500	chr18:47087075-47087446	Island
hyper2505	N_excessive_5	6.1E-02	0.05973423	0.01	0.05	19	925903	cg07752304	ARID3A	TSS200	chr19:925120-926816	Island
hyper2506	N_excessive_5	3.8E-02	0.2198596	0.11	0.11	19	9434523	cg23179379	ZNF559	TSS1500	chr19:9434784-9435144	N_Shore
hyper2507	N_excessive_5	4.8E-03	0.4124852	0.06	0.35	19	12203029	cg20773651	ZNF788	TSS200	chr19:12203028-12203503	Island
hyper2508	N_excessive_5	6.6E-03	0.5352734	0.08	0.46	19	12203066	cg09489445	ZNF788	TSS200	chr19:12203028-12203503	Island
hyper2509	N_excessive_5	4.8E-04	0.4198285	0.05	0.37	19	12203198	cg06384763	ZNF788	Body	chr19:12203028-12203503	Island
hyper2510	N_excessive_5	1.0E-07	0.4003082	0.04	0.36	19	12203315	cg01172903	ZNF788	Body	chr19:12203028-12203503	Island
hyper2511	N_excessive_5	4.2E-06	0.4638691	0.04	0.42	19	12203349	cg11767984	ZNF788	Body	chr19:12203028-12203503	Island
hyper2512	N_excessive_5	2.3E-07	0.2869674	0.07	0.22	19	12203644	cg01404753	ZNF788	Body	chr19:12203028-12203503	S_Shore
hyper2513	N_excessive_5	1.9E-06	0.4323865	0.06	0.37	19	37997730	cg07482795	ZNF793	TSS200	chr19:37997790-37998125	N_Shore
hyper2514	N_excessive_5	1.9E-08	0.2388238	0.09	0.15	20	18118240	cg18396157	PET117	TSS1500	chr20:18117962-18118989	Island
hyper2515	N_excessive_5	2.8E-09	0.06624232	0.00	0.06	20	30795610	cg25987077	POFUT1;PLAGL2;POFUT1	TSS200;TSS200;TSS200	chr20:30795282-30795841	Island
hyper2516	N_excessive_5	2.6E-03	0.1446201	0.04	0.11	20	37434950	cg25932164	PPP1R16B	5'UTR	chr20:37434206-37435592	Island
hyper2517	N_excessive_5	2.7E-04	0.08435883	0.02	0.06	21	22370758	cg25818292	NCAM2;NCAM2	5'UTR;1stExon	chr21:22369423-22370582	S_Shore
hyper2518	N_excessive_5	2.1E-04	0.1008918	0.03	0.08	21	38935991	cg23484755			chr21:38935854-38936546	Island
hyper2519	N_excessive_5	8.7E-04	0.1114774	0.03	0.08	22	31090747	cg08170140	OSBP2	TSS200	chr22:31090200-31091334	Island
hyper2520	N_excessive_5	6.4E-09	0.1356723	0.01	0.12	22	39268508	cg04215348	CBX6	TSS1500	chr22:39267539-39269160	Island
hyper2521	N_excessive_5	2.5E-06	0.06266803	0.01	0.05	22	45404843	cg01033463	PHF21B;PHF21B	Body;Body	chr22:45403036-45406372	Island

Supplementary Table 2: list of 977 hypomethylated outliers

#	Sample_ID	Outlier-test P-value (adjusted)	Outlier's beta-value	Mean of the beta-values of others	delta-beta	CHR	MAPINFO_hg19	TargetID	UCSC_REFGENE_NAME	UCSC_REFGENE_GROUP	UCSC_CPG_ISLANDS_NAME	RELATION_TO_ UCSC_CPG_ISLAND
hypo1	FGR_insufficient_1	6.1E-16	0.41	0.90	-0.49	1	1415868	cg00695214	ATAD3B	Body	chr1:1417285-1417753	N_Shore
hypo2	FGR_insufficient_1	6.5E-03	0.25	0.62	-0.37	1	91190620	cg15452970			chr1:91190489-91192804	Island
hypo3	FGR_insufficient_1	7.1E-03	0.57	0.86	-0.29	1	223565773	cg26572518	C1orf65	TSS1500	chr1:223566642-223567268	N_Shore
hypo4	FGR_insufficient_1	1.1E-03	0.20	0.70	-0.50	2	30478251	cg26176018	LBH	Body		
hypo5	FGR_insufficient_1	3.5E-05	0.33	0.54	-0.21	2	62732892	cg10848792	TMEM17	Body	chr2:62732758-62733497	Island
hypo6	FGR_insufficient_1	4.7E-03	0.13	0.46	-0.32	2	62733114	cg07290273	TMEM17	Body	chr2:62732758-62733497	Island
hypo7	FGR_insufficient_1	4.0E-04	0.22	0.54	-0.31	2	62733485	cg17444943	TMEM17;TMEM17	5'UTR;1stExon	chr2:62732758-62733497	Island
hypo8	FGR_insufficient_1	1.9E-02	0.17	0.54	-0.37	2	62733576	cg12385425	TMEM17;TMEM17	5'UTR;1stExon	chr2:62732758-62733497	S_Shore
hypo9	FGR_insufficient_1	6.6E-04	0.64	0.87	-0.23	2	67387038	cg07850407				
hypo10	FGR_insufficient_1	2.0E-02	0.63	0.92	-0.28	2	128965633	cg04724176				
hypo11	FGR_insufficient_1	8.8E-02	0.13	0.56	-0.44	2	134024093	cg12915892	NCKAP5;NCKAP5	Body;Body	chr2:134023947-134024467	Island
hypo12	FGR_insufficient_1	2.5E-03	0.28	0.64	-0.36	2	134024266	cg22232500	NCKAP5;NCKAP5	Body;Body	chr2:134023947-134024467	Island
hypo13	FGR_insufficient_1	1.9E-02	0.66	0.90	-0.24	2	184125416	cg07423985			chr2:184125343-184125583	Island
hypo14	FGR_insufficient_1	6.2E-03	0.55	0.84	-0.29	3	10202595	cg18073702			chr3:10206449-10207024	N_Shelf
hypo15	FGR_insufficient_1	1.4E-04	0.75	0.95	-0.20	3	134758333	cg07049323	EPHB1	Body		
hypo16	FGR_insufficient_1	1.2E-02	0.39	0.75	-0.36	3	197842485	cg01392017			chr3:197840490-197840783	S_Shore
hypo17	FGR_insufficient_1	6.5E-02	0.43	0.83	-0.40	4	114926897	cg04581571				
hypo18	FGR_insufficient_1	6.9E-06	0.81	0.96	-0.14	5	6749408	cg00530298	POLS	Body		
hypo19	FGR_insufficient_1	3.6E-02	0.55	0.82	-0.27	5	97971808	cg06803420				
hypo20	FGR_insufficient_1	6.5E-06	0.58	0.85	-0.27	5	138842004	cg08331427	ECSCR	Body		
hypo21	FGR_insufficient_1	5.4E-02	0.62	0.86	-0.25	5	140871464	cg16032518	PCDHGA4;PCDHGC3;PCDHGA1	Body;Body;Body;Body;Body;Body;	chr5:140871064-140872335	Island
hypo22	FGR_insufficient_1	9.3E-02	0.68	0.84	-0.16	5	140871590	cg17297238	PCDHGA4;PCDHGC3;PCDHGA1	Body;Body;Body;Body;Body;Body;	chr5:140871064-140872335	Island
hypo23	FGR_insufficient_1	3.8E-07	0.64	0.93	-0.29	6	28874479	cg20544852	TRIM27	Body		
hypo24	FGR_insufficient_1	2.4E-03	0.49	0.82	-0.34	6	32034322	cg08895562	TNXB	Body		
hypo25	FGR_insufficient_1	1.5E-03	0.76	0.88	-0.12	6	72294420	cg07816653			chr6:72294372-72294988	Island
hypo26	FGR_insufficient_1	2.0E-02	0.06	0.59	-0.52	6	170338591	cg12173150			chr6:170338214-170339677	Island
hypo27	FGR_insufficient_1	9.7E-10	0.54	0.85	-0.31	7	27070361	cg26572901				
hypo28	FGR_insufficient_1	2.7E-02	0.69	0.90	-0.21	8	70312989	cg25827124				
hypo29	FGR_insufficient_1	2.5E-02	0.78	0.95	-0.17	9	135432492	cg13725340	C9orf171	Body		
hypo30	FGR_insufficient_1	3.2E-02	0.30	0.65	-0.35	10	50887049	cg05834477	C10orf53;C10orf53	TSS1500;TSS1500	chr10:50887565-50887862	N_Shore
hypo31	FGR_insufficient_1	1.0E-02	0.53	0.88	-0.34	10	134992080	cg03580836	KNDC1	Body	chr10:134993121-134993324	N_Shore
hypo32	FGR_insufficient_1	9.8E-04	0.66	0.86	-0.20	11	503807	cg19223782	RNH1;RNH1;RNH1;RNH1;RNH1;	5'UTR;5'UTR;5'UTR;5'UTR;5'UTR	chr11:504189-504659	N_Shore
hypo33	FGR_insufficient_1	6.9E-02	0.77	0.94	-0.17	11	3435091	cg04128776			chr11:3435033-3435239	Island
hypo34	FGR_insufficient_1	5.3E-02	0.77	0.95	-0.18	11	36596040	cg25154959	RAG1	Body		
hypo35	FGR_insufficient_1	3.0E-04	0.61	0.86	-0.25	12	33589594	cg06721860	SYT10	Body	chr12:33591763-33593050	N_Shelf
hypo36	FGR_insufficient_1	2.6E-03	0.45	0.79	-0.34	12	52800873	cg20679157	KRT82	TSS1500		
hypo37	FGR_insufficient_1	5.2E-04	0.73	0.96	-0.22	13	112905745	cg01562175				
hypo38	FGR_insufficient_1	2.5E-05	0.90	0.98	-0.07	14	20786069	cg25091820	CCNB1IP1;CCNB1IP1;CCNB1IP1	5'UTR;5'UTR;5'UTR;5'UTR		
hypo39	FGR_insufficient_1	8.9E-11	0.52	0.85	-0.33	16	2239275	cg03524868	CASKIN1	Body	chr16:2236712-2236939	S_Shelf
hypo40	FGR_insufficient_1	5.1E-08	0.57	0.87	-0.30	17	3872291	cg11252995				
hypo41	FGR_insufficient_1	9.1E-04	0.33	0.73	-0.40	18	55095177	cg05735180			chr18:55094825-55096310	Island
hypo42	FGR_insufficient_1	1.6E-04	0.25	0.71	-0.47	18	55095249	cg15222899			chr18:55094825-55096310	Island
hypo43	FGR_insufficient_1	8.8E-02	0.04	0.39	-0.35	18	55102005	cg25800170	ONECUT2	TSS1500	chr18:55102427-55102708	N_Shore
hypo44	FGR_insufficient_1	2.6E-02	0.90	0.98	-0.08	21	47344376	cg10125522	PCBP3;PCBP3	Body;Body	chr21:47343715-47345139	Island
hypo45	FGR_insufficient_2	2.2E-06	0.59	0.84	-0.26	1	1344064	cg25939543	MRPL20	TSS1500	chr1:1342004-1342952	S_Shore
hypo46	FGR_insufficient_2	2.1E-06	0.43	0.84	-0.41	1	10388646	cg19773385	KIF1B	Body	chr1:10386183-10386390	S_Shelf
hypo47	FGR_insufficient_2	3.7E-13	0.49	0.86	-0.37	1	161009463	cg22110569	TSTD1;TSTD1;TSTD1;USF1;USF	TSS1500;TSS1500;TSS1500;3'UT	chr1:161008377-161008830	S_Shore
hypo48	FGR_insufficient_2	2.1E-15	0.48	0.88	-0.40	2	8593963	cg07903001			chr2:8596907-8597573	N_Shelf
hypo49	FGR_insufficient_2	4.8E-02	0.45	0.80	-0.36	2	32483287	cg24609304	NLRC4	5'UTR		
hypo50	FGR_insufficient_2	2.9E-03	0.47	0.73	-0.27	2	32483392	cg23459416	NLRC4	5'UTR		
hypo51	FGR_insufficient_2	2.0E-14	0.49	0.91	-0.41	2	236864888	cg13192735	AGAP1;AGAP1	Body;Body	chr2:236867652-236867906	N_Shelf
hypo52	FGR_insufficient_2	2.5E-07	0.54	0.88	-0.34	4	3444147	cg07364841	HGFAC	Body		
hypo53	FGR_insufficient_2	7.3E-09	0.34	0.73	-0.39	4	6306335	cg18764836			chr4:6303533-6304210	S_Shelf
hypo54	FGR_insufficient_2	3.5E-02	0.42	0.68	-0.25	4	83674694	cg18948646	SCD5;MIR575;SCD5	Body;TSS200;Body		
hypo55	FGR_insufficient_2	1.8E-06	0.43	0.74	-0.31	5	864385	cg15052217	BRD9;BRD9;BRD9	Body;3'UTR;3'UTR	chr5:865317-865642	N_Shore
hypo56	FGR_insufficient_2	2.5E-02	0.43	0.66	-0.23	6	31936565	cg22098742	SKIV2L	Body	chr6:31939730-31940559	N_Shelf
hypo57	FGR_insufficient_2	6.7E-15	0.50	0.93	-0.43	7	1949247	cg03530573	MAD1L1;MAD1L1;MAD1L1	Body;Body;Body	chr7:1950279-1950482	N_Shore
hypo58	FGR_insufficient_2	2.2E-05	0.42	0.76	-0.34	7	66307208	cg22769911	LOC729156	Body	chr7:66309503-66310040	N_Shelf
hypo59	FGR_insufficient_2	1.9E-05	0.14	0.66	-0.52	7	66308541	cg07448840	LOC729156	Body	chr7:66309503-66310040	N_Shore
hypo60	FGR_insufficient_2	6.9E-04	0.71	0.83	-0.12	7	66364844	cg13511293			chr7:66366966-66367234	N_Shelf
hypo61	FGR_insufficient_2	3.9E-02	0.40	0.68	-0.28	7	80544479	cg15556990	SEMA3C	Body	chr7:80548306-80548726	N_Shelf
hypo62	FGR_insufficient_2	5.2E-14	0.60	0.93	-0.33	8	42306711	cg18334848	SLC20A2	Body		
hypo63	FGR_insufficient_2	2.8E-07	0.55	0.83	-0.28	8	129073877	cg24146714	PVT1	Body		
hypo64	FGR_insufficient_2	4.2E-14	0.53	0.90	-0.37	10	13696795	cg04686117	FRMD4A	Body	chr10:13698562-13699540	N_Shore
hypo65	FGR_insufficient_2	2.3E-11	0.59	0.92	-0.33	10	27146906	cg10311020	ABI1;ABI1;ABI1;ABI1	Body;Body;Body;Body	chr10:27149166-27150229	N_Shelf
hypo66	FGR_insufficient_2	2.0E-06	0.53	0.78	-0.25	10	32639458	cg20306821			chr10:32635217-32636655	S_Shelf
hypo67	FGR_insufficient_2	4.3E-10	0.61	0.92	-0.30	10	127571375	cg08199003	DHX32	TSS1500		
hypo68	FGR_insufficient_2	1.6E-04	0.56	0.88	-0.32	11	18271401	cg03687333	SAA2;SAA2	TSS1500;TSS1500		
hypo69	FGR_insufficient_2	7.6E-10	0.54	0.87	-0.33	12	68056168	cg19968199	DYRK2;DYRK2	3'UTR;3'UTR		
hypo70	FGR_insufficient_2	2.0E-02	0.42	0.79	-0.37	12	77260534	cg26994885	CSRP2	5'UTR		
hypo71	FGR_insufficient_2	1.2E-14	0.50	0.88	-0.38	15	101567894	cg24950894	LRRK1	Body		
hypo72	FGR_insufficient_2	5.9E-07	0.86	0.97	-0.10	16	50613442	cg09930155	NKD1	Body	chr16:50613108-50613701	Island
hypo73	FGR_insufficient_2	1.1E-03	0.70	0.86	-0.15	16	67757411	cg01114989	RANBP10	3'UTR	chr16:67752849-67753446	S_Shelf
hypo74	FGR_insufficient_2	6.8E-03	0.30	0.46	-0.15	20	52209811	cg09123431			chr20:52209590-52211360	Island
hypo75	FGR_insufficient_2	3.3E-03	0.41	0.54	-0.14	22	17090862	cg26581886	psiTPTE22	Body	chr22:17090455-17091224	Island
hypo76	FGR_insufficient_2	2.6E-09	0.55	0.88	-0.34	22	38225114	cg22082700			chr22:38220589-38221619	S_Shelf
hypo77	FGR_insufficient_3	1.1E-09	0.65	0.92	-0.27	1	33744259	cg02020616	ZNF362	Body	chr1:33745741-33745990	N_Shore
hypo78	FGR_insufficient_3	1.9E-05	0.62	0.83	-0.22	2	239403239	cg17850055				
hypo79	FGR_insufficient_3	9.8E-08	0.48	0.83	-0.35	3	13864471	cg21224025	WNT7A	Body	chr3:13860434-13860882	S_Shelf
hypo80	FGR_insufficient_3	1.7E-08	0.51	0.79	-0.28	4	184686701	cg16039660				
hypo81	FGR_insufficient_3	2.2E-02	0.57	0.88	-0.31	4	187418419	cg12937770			chr4:187422034-187422244	N_Shelf
hypo82	FGR_insufficient_3	1.4E-03	0.09	0.51	-0.42	5	92178	cg10165894			chr5:92007-92438	Island
hypo83	FGR_insufficient_3	1.8E-02	0.16	0.55	-0.39	5	92376	cg25622036			chr5:92007-92438	Island
hypo84	FGR_insufficient_3	3.1E-04	0.74	0.89	-0.16	5	663787	cg22936884	TPPP	3'UTR	chr5:665187-665401	N_Shore
hypo85	FGR_insufficient_3	1.5E-10	0.59	0.91	-0.32	5	31361135	cg20539449				
hypo86	FGR_insufficient_3	1.4E-04	0.70	0.88	-0.18	5	165513101	cg14069726				
hypo87	FGR_insufficient_3	1.7E-12	0.60	0.94	-0.35	6	30156892	cg23814883	TRIM26	Body		
hypo88	FGR_insufficient_3	2.5E-09	0.50	0.86	-0.37	6	91003200	cg03035849	BACH2;BACH2	5'UTR;5'UTR	chr6:91004795-91006944	N_Shore
hypo89	FGR_insufficient_3	1.2E-12	0.34	0.88	-0.55	6	110504754	cg10512408	CDC40	Body	chr6:110500025-110500966	S_Shelf
hypo90	FGR_insufficient_3	1.4E-05	0.65	0.85	-0.21	7	11468680	cg05232241	THSD7A	Body		
hypo91	FGR_insufficient_3	6.7E-02	0.69	0.87	-0.18	7	11558886	cg03914237	THSD7A	Body		
hypo92	FGR_insufficient_3	5.9E-02	0.70	0.90	-0.20	7	13935467	cg02971555	ETV1;ETV1;ETV1;ETV1;ETV1;ET	3'UTR;3'UTR;3'UTR;3'UTR;3'UTR;3'UTR;3'UTR		
hypo93	FGR_insufficient_3	6.9E-04	0.66	0.82	-0.16	7	55805069	cg14537194			chr7:55808712-55809001	N_Shelf
hypo94	FGR_insufficient_3	1.5E-03	0.67	0.95	-0.28	8	1460569	cg15938529	DLGAP2	5'UTR		
hypo95	FGR_insufficient_3	3.0E-02	0.77	0.91	-0.15	8	52432288	cg09723536	PXDNL	Body		
hypo96	FGR_insufficient_3	2.2E-06	0.55	0.87	-0.32	10	130855111	cg13822329				
hypo97	FGR_insufficient_3	1.2E-09	0.59	0.88	-0.29	11	108377279	cg03096378	EXPH5;EXPH5;EXPH5	3'UTR;3'UTR;3'UTR		
hypo98	FGR_insufficient_3	1.7E-09	0.54	0.88	-0.34	11	126098838	cg25857886	FAM118B	5'UTR		
hypo99	FGR_insufficient_3	1.4E-05	0.09	0.54	-0.44	13	23500367	cg06743703			chr13:23500589-23501398	N_Shore
hypo100	FGR_insufficient_3	1.4E-05	0.38	0.81	-0.43	13	44452398	cg27311397	CCDC122;C13orf31	5'UTR;TSS1500	chr13:44453207-44454295	N_Shore
hypo101	FGR_insufficient_3	8.5E-02	0.21	0.37	-0.16	14	104928289	cg07483462			chr14:104927441-104928653	Island
hypo102	FGR_insufficient_3	7.5E-15	0.49	0.89	-0.40	14	105778041	cg19689151			chr14:105780339-105782027	N_Shelf
hypo103	FGR_insufficient_3	1.6E-03	0.70	0.87	-0.17	15	25415389	cg20460234	SNORD115-1	TSS1500		
hypo104	FGR_insufficient_3	8.7E-11	0.60	0.89	-0.30	15	75146573	cg26451198	SCAMP2	Body		
hypo105	FGR_insufficient_3	2.0E-12	0.60	0.91	-0.31	17	76854641	cg18621277	TIMP2	Body	chr17:76851759-76851973	S_Shelf
hypo106	FGR_insufficient_3	1.2E-04	0.58	0.79	-0.20	19	2200504	cg08527435	DOT1L	Body	chr19:2199921-2200126	S_Shore
hypo107	FGR_insufficient_4	2.9E-15	0.46	0.91	-0.45	1	27874258	cg00178877	AHDC1	Body	chr1:27874417-27874638	N_Shore
hypo108	FGR_insufficient_4	5.4E-04	0.42	0.74	-0.32	1	46910885	cg12858469			chr1:46913786-46914343	N_Shelf
hypo109	FGR_insufficient_4	2.3E-05	0.45	0.79	-0.34	1	167598560	cg04275362	RCSD1	TSS1500	chr1:167599464-167599839	N_Shore
hypo110	FGR_insufficient_4	2.8E-07	0.45	0.78	-0.33	1	226887044	cg02896872	ITPKB	Body	chr1:226891032-226891381	N_Shelf
hypo111	FGR_insufficient_4	8.1E-02	0.38	0.85	-0.47	2	23918773	cg09990539	KLHL29	Body		
hypo112	FGR_insufficient_4	7.4E-02	0.69	0.90	-0.21	2	151564111	cg15770150				
hypo113	FGR_insufficient_4	3.7E-09	0.56	0.88	-0.32	3	48600317	cg17226356	UCN2	Body		
hypo114	FGR_insufficient_4	7.5E-05	0.55	0.85	-0.30	3	112935090	cg02858625	BOC	5'UTR	chr3:112930437-112931506	S_Shelf
hypo115	FGR_insufficient_4	1.8E-08	0.53	0.87	-0.35	4	159033548	cg25513740				
hypo116	FGR_insufficient_4	1.7E-12	0.48	0.86	-0.38	6	31640160	cg18683309	LY6G5B	3'UTR		
hypo117	FGR_insufficient_4	1.6E-12	0.53	0.92	-0.39	6	160206205	cg26296969	TCP1;TCP1	Body;Body	chr6:160209941-160212015	N_Shelf
hypo118	FGR_insufficient_4	5.0E-08	0.30	0.60	-0.30	7	39051529	cg26154546	POU6F2;POU6F2	Body;Body		
hypo119	FGR_insufficient_4	5.0E-03	0.78	0.92	-0.14	7	154685308	cg04923394	DPP6;DPP6;DPP6	3'UTR;3'UTR;3'UTR	chr7:154684402-154684630	S_Shore
hypo120	FGR_insufficient_4	1.0E-11	0.48	0.84	-0.35	8	142296023	cg12993642			chr8:142295218-142295879	S_Shore
hypo121	FGR_insufficient_4	6.0E-11	0.64	0.88	-0.24	10	30071374	cg15574316				
hypo122	FGR_insufficient_4	1.8E-06	0.42	0.72	-0.30	10	134222445	cg15995714	PWWP2B;PWWP2B	3'UTR;Body	chr10:134218268-134219615	S_Shelf
hypo123	FGR_insufficient_4	2.0E-12	0.07	0.56	-0.49	11	2721428	cg20699737	KCNQ1OT1;KCNQ1;KCNQ1	TSS1500;Body;Body	chr11:2720410-2722087	Island
hypo124	FGR_insufficient_4	1.5E-05	0.40	0.53	-0.13	11	2721437	cg26547719	KCNQ1OT1;KCNQ1;KCNQ1	TSS1500;Body;Body	chr11:2720410-2722087	Island

hypo125	FGR_insufficient_4	7.3E-13	0.52	0.90	-0.37	11	35644854	cg13916633			chr11:35639629-35641485	S_Shelf
hypo126	FGR_insufficient_4	4.3E-04	0.59	0.88	-0.30	11	129683974	cg08524015			chr11:129685737-129686211	N_Shore
hypo127	FGR_insufficient_4	7.4E-09	0.57	0.89	-0.31	15	33188759	cg27158011	FMN1	Body		
hypo128	FGR_insufficient_4	2.9E-13	0.53	0.90	-0.38	16	2218945	cg27166767	TRAF7	Body	chr16:2214222-2215518	S_Shelf
hypo129	FGR_insufficient_4	1.4E-02	0.94	0.98	-0.04	16	57416074	cg05410102	CX3CL1	Body	chr16:57415974-57416338	Island
hypo130	FGR_insufficient_4	8.0E-13	0.55	0.89	-0.34	17	56069567	cg24008395			chr17:56064830-56066051	S_Shelf
hypo131	FGR_insufficient_4	3.6E-03	0.84	0.94	-0.10	19	11754404	cg15555622			chr19:11750529-11751006	S_Shelf
hypo132	FGR_insufficient_4	8.2E-04	0.55	0.82	-0.27	19	47158242	cg06454084	DACT3	Body		
hypo133	FGR_insufficient_4	2.7E-02	0.47	0.78	-0.31	20	23470274	cg11879776	CST8	TSS1500		
hypo134	FGR_insufficient_4	2.1E-06	0.41	0.70	-0.29	20	36975083	cg08351331	LBP	Body		
hypo135	FGR_insufficient_4	3.4E-08	0.43	0.78	-0.35	21	47803078	cg03165002	PCNT	Body	chr21:47802465-47804760	Island
hypo136	FGR_insufficient_5	2.8E-10	0.43	0.80	-0.37	1	1597086	cg06848468	CDK11B;CDK11B;CDK11B;CDK1	Body;Body;Body;Body;Body;3'UT	chr1:1600981-1601231	N_Shelf
hypo137	FGR_insufficient_5	4.8E-04	0.56	0.82	-0.26	1	21552839	cg07114751	ECE1;ECE1;ECE1;ECE1	Body;Body;Body;Body	chr1:21551574-21551931	S_Shore
hypo138	FGR_insufficient_5	1.0E-04	0.50	0.71	-0.21	1	44686794	cg23599716	ERI3	3'UTR		
hypo139	FGR_insufficient_5	3.3E-05	0.47	0.70	-0.23	2	25146822	cg20336204			chr2:25142472-25143689	S_Shelf
hypo140	FGR_insufficient_5	1.6E-04	0.61	0.88	-0.27	2	26098817	cg00844078	ASXL2	Body	chr2:26100513-26101653	N_Shore
hypo141	FGR_insufficient_5	2.1E-08	0.51	0.84	-0.32	2	99081350	cg11555067	INPP4A;INPP4A;INPP4A;INPP4A	5'UTR;5'UTR;5'UTR;5'UTR		
hypo142	FGR_insufficient_5	3.0E-02	0.55	0.80	-0.24	2	223765655	cg22909085	ACSL3;ACSL3	5'UTR;5'UTR		
hypo143	FGR_insufficient_5	4.3E-13	0.17	0.83	-0.66	2	242580471	cg11574665	ATG4B;ATG4B	Body;Body	chr2:242575706-242577492	S_Shelf
hypo144	FGR_insufficient_5	2.6E-10	0.60	0.90	-0.30	4	75020915	cg12718874			chr4:75023577-75024037	N_Shelf
hypo145	FGR_insufficient_5	3.3E-03	0.60	0.86	-0.26	4	109115092	cg15508749				
hypo146	FGR_insufficient_5	7.4E-03	0.47	0.74	-0.27	6	144329887	cg23460430	PLAGL1;PLAGL1;PLAGL1;PLAGL	TSS1500;TSS1500;5'UTR;TSS15	chr6:144328916-144329847	S_Shore
hypo147	FGR_insufficient_5	6.3E-08	0.49	0.79	-0.30	7	1575737	cg24647108	MAFK	5'UTR	chr7:1575736-1575993	Island
hypo148	FGR_insufficient_5	2.5E-05	0.48	0.77	-0.29	7	133056105	cg21826833	EXOC4;EXOC4	Body;Body		
hypo149	FGR_insufficient_5	4.1E-02	0.50	0.80	-0.29	8	102221396	cg22009239			chr8:102217317-102218461	S_Shelf
hypo150	FGR_insufficient_5	3.2E-03	0.61	0.83	-0.22	10	71876389	cg13908507	AIFM2	Body		
hypo151	FGR_insufficient_5	3.4E-04	0.16	0.74	-0.58	11	65685316	cg16649728	DRAP1;C11orf68;C11orf68	TSS1500;Body;Body	chr11:65685100-65685364	Island
hypo152	FGR_insufficient_5	5.0E-02	0.81	0.90	-0.09	11	75066381	cg07225458			chr11:75062224-75062977	S_Shelf
hypo153	FGR_insufficient_5	1.2E-06	0.61	0.89	-0.28	11	94467343	cg06103613				
hypo154	FGR_insufficient_5	6.4E-06	0.58	0.88	-0.30	12	341431	cg04647176	SLC6A13	Body		
hypo155	FGR_insufficient_5	3.3E-06	0.48	0.72	-0.24	12	132516068	cg00089214	EP400	Body	chr12:132514283-132514525	S_Shore
hypo156	FGR_insufficient_5	7.1E-03	0.20	0.60	-0.40	13	51417469	cg17241776	DLEU7	1stExon	chr13:51417371-51418149	Island
hypo157	FGR_insufficient_5	1.1E-06	0.51	0.79	-0.27	17	75312494	cg16722931	SEPT9;SEPT9	5'UTR;Body		
hypo158	FGR_insufficient_5	3.1E-16	0.29	0.87	-0.58	17	79792936	cg01847062	DYSFIP1	TSS200	chr17:79790530-79791553	S_Shore
hypo159	FGR_insufficient_5	5.4E-02	0.55	0.83	-0.29	19	3355151	cg25919177			chr19:3358495-3361248	N_Shelf
hypo160	FGR_insufficient_5	5.6E-04	0.57	0.81	-0.24	19	54327780	cg25866075	NLRP12	TSS200		
hypo161	FGR_insufficient_5	6.7E-02	0.13	0.45	-0.33	20	44880000	cg07676002	CDH22;CDH22	1stExon;5'UTR	chr20:44879699-44880288	Island
hypo162	FGR_insufficient_5	8.8E-03	0.15	0.38	-0.23	20	52789646	cg24582168	CYP24A1;CYP24A1	Body;Body	chr20:52789252-52790986	Island
hypo163	FGR_insufficient_5	3.8E-07	0.26	0.57	-0.31	20	52790063	cg23685712	CYP24A1;CYP24A1	1stExon;1stExon	chr20:52789252-52790986	Island
hypo164	FGR_adequate_1	4.4E-08	0.37	0.81	-0.44	1	1159106	cg09819798	SDF4;SDF4	Body;Body	chr1:1159168-1159528	N_Shore
hypo165	FGR_adequate_1	2.2E-04	0.58	0.82	-0.24	3	30733753	cg05074709	TGFBR2;TGFBR2	3'UTR;3'UTR		
hypo166	FGR_adequate_1	4.9E-03	0.65	0.86	-0.21	3	31614334	cg03923676	STT3B	Body		
hypo167	FGR_adequate_1	2.6E-05	0.61	0.86	-0.26	3	190043045	cg27504745			chr3:190039817-190040465	S_Shelf
hypo168	FGR_adequate_1	4.1E-08	0.70	0.87	-0.17	4	730453	cg20823529	PCGF3	Body	chr4:730282-731005	Island
hypo169	FGR_adequate_1	2.0E-02	0.38	0.81	-0.44	5	33794720	cg07784793	ADAMTS12	Body		
hypo170	FGR_adequate_1	2.3E-02	0.59	0.82	-0.23	5	66123702	cg17606548	MAST4;MAST4;MAST4	TSS1500;Body;Body		
hypo171	FGR_adequate_1	2.5E-10	0.68	0.93	-0.25	5	130497746	cg01175053	HINT1;HINT1;HINT1	Body;Body;Body	chr5:130500226-130501095	N_Shelf
hypo172	FGR_adequate_1	7.6E-09	0.34	0.85	-0.51	6	4923095	cg24598993	CDYL;CDYL;CDYL;CDYL	Body;Body;Body;Body		
hypo173	FGR_adequate_1	3.5E-12	0.63	0.92	-0.29	6	31854545	cg05425464	EHMT2;EHMT2	Body;Body	chr6:31856896-31857104	N_Shelf
hypo174	FGR_adequate_1	2.7E-09	0.71	0.94	-0.23	6	149367279	cg06827883	UST	Body		
hypo175	FGR_adequate_1	7.3E-10	0.61	0.92	-0.31	8	124031034	cg11604216	DERL1;DERL1	Body;Body		
hypo176	FGR_adequate_1	4.7E-04	0.64	0.86	-0.23	11	63990575	cg20160996	FERMT3;FERMT3	Body;Body	chr11:63993589-63993834	N_Shelf
hypo177	FGR_adequate_1	1.0E-06	0.64	0.91	-0.27	11	63990595	cg06219103	FERMT3;FERMT3	Body;Body	chr11:63993589-63993834	N_Shelf
hypo178	FGR_adequate_1	3.3E-10	0.70	0.97	-0.27	11	63990629	cg06243084	FERMT3;FERMT3	Body;Body	chr11:63993589-63993834	N_Shelf
hypo179	FGR_adequate_1	4.7E-03	0.62	0.85	-0.23	11	71931577	cg16682721	FOLR2;FOLR2;FOLR2;FOLR2	Body;Body;Body;Body	chr11:71934247-71936321	N_Shelf
hypo180	FGR_adequate_1	1.5E-02	0.56	0.81	-0.25	11	118657363	cg05587627	DDX6	5'UTR	chr11:118661305-118663026	N_Shelf
hypo181	FGR_adequate_1	9.2E-04	0.61	0.83	-0.21	12	110838588	cg13557301	ANAPC7;ANAPC7	Body;Body	chr12:110841021-110841548	N_Shelf
hypo182	FGR_adequate_1	5.9E-09	0.66	0.91	-0.24	14	62021555	cg18703243				
hypo183	FGR_adequate_1	3.2E-09	0.57	0.81	-0.24	14	103556656	cg03007309			chr14:103557606-103558235	N_Shore
hypo184	FGR_adequate_1	4.8E-11	0.65	0.92	-0.27	16	57484979	cg06578063	COQ9	Body	chr16:57480995-57481666	S_Shelf
hypo185	FGR_adequate_1	2.8E-11	0.57	0.89	-0.32	19	46198630	cg08182826	QPCTL;QPCTL	Body;Body	chr19:46195482-46196292	S_Shelf
hypo186	FGR_adequate_1	8.6E-04	0.80	0.94	-0.14	19	49611733	cg19281603	SNRNP70	3'UTR	chr19:49610928-49611410	S_Shore
hypo187	FGR_adequate_2	6.2E-04	0.51	0.73	-0.22	1	1234367	cg22675767	ACAP3	Body		
hypo188	FGR_adequate_2	2.7E-10	0.61	0.89	-0.28	1	6267579	cg19694465	RNF207	Body	chr1:6268820-6269588	N_Shore
hypo189	FGR_adequate_2	4.7E-06	0.60	0.84	-0.24	1	15764869	cg03096785	CTRC	TSS200		
hypo190	FGR_adequate_2	8.9E-11	0.61	0.89	-0.28	1	119715385	cg18451982				
hypo191	FGR_adequate_2	9.9E-05	0.63	0.84	-0.21	1	231838098	cg10116456	TSNAX-DISC1;DISC1;DISC1;DIS	Body;Body;Body;Body;Body;Body;Body;Body;Body;Body;Body;Body;Body;Body;Body;Body		
hypo192	FGR_adequate_2	1.1E-05	0.57	0.82	-0.26	2	47305388	cg09439348				
hypo193	FGR_adequate_2	1.0E-03	0.70	0.87	-0.17	2	84105004	cg03437666				
hypo194	FGR_adequate_2	2.7E-03	0.66	0.88	-0.22	2	237468416	cg23138373				
hypo195	FGR_adequate_2	1.1E-02	0.88	0.96	-0.08	4	3435449	cg06919392	RGS12;RGS12	Body;Body		
hypo196	FGR_adequate_2	6.7E-04	0.74	0.90	-0.16	4	3949662	cg03309545	LOC348926	Body		
hypo197	FGR_adequate_2	6.3E-04	0.64	0.84	-0.21	4	77480401	cg19600368	SHROOM3	Body		
hypo198	FGR_adequate_2	1.6E-02	0.74	0.89	-0.15	6	169622298	cg27264172	THBS2	Body	chr6:169622297-169622520	Island
hypo199	FGR_adequate_2	1.1E-03	0.73	0.90	-0.17	7	2133441	cg08024992	MAD1L1;MAD1L1;MAD1L1	Body;Body;Body		
hypo200	FGR_adequate_2	3.2E-09	0.69	0.93	-0.23	7	138602271	cg21133261	KIAA1549;KIAA1549	Body;Body		
hypo201	FGR_adequate_2	6.1E-08	0.65	0.91	-0.25	14	102223459	cg23290739			chr14:102226751-102227050	N_Shelf
hypo202	FGR_adequate_2	5.3E-03	0.68	0.87	-0.18	15	40265105	cg09964084	EIF2AK4	Body	chr15:40268581-40269061	N_Shelf
hypo203	FGR_adequate_2	1.1E-02	0.90	0.97	-0.07	16	969918	cg01277300	LMF1	Body	chr16:971429-971825	N_Shore
hypo204	FGR_adequate_2	4.0E-04	0.56	0.82	-0.26	17	33927921	cg22193691	AP2B1;AP2B1	Body;Body	chr17:33923588-33924144	S_Shelf
hypo205	FGR_adequate_2	4.3E-02	0.77	0.94	-0.17	17	48263204	cg25735490	COL1A1	Body		
hypo206	FGR_adequate_2	1.5E-08	0.76	0.94	-0.18	17	76802223	cg21840211	USP36	Body	chr17:76798154-76798461	S_Shelf
hypo207	FGR_adequate_2	9.4E-05	0.76	0.87	-0.11	22	44889756	cg04154583	LDOC1L	3'UTR	chr22:44892393-44894049	N_Shelf
hypo208	FGR_adequate_3	1.1E-02	0.90	0.98	-0.07	1	981392	cg07501049	AGRN	Body	chr1:981068-981400	Island
hypo209	FGR_adequate_3	1.6E-12	0.55	0.92	-0.37	1	1023163	cg04315086	C1orf159	Body	chr1:1023143-1023347	Island
hypo210	FGR_adequate_3	6.3E-02	0.58	0.82	-0.24	2	104996109	cg04058305				
hypo211	FGR_adequate_3	3.9E-02	0.71	0.90	-0.18	2	134254352	cg26479279	NCKAP5;NCKAP5	Body;Body		
hypo212	FGR_adequate_3	6.9E-05	0.55	0.87	-0.32	2	162278173	cg08314300	TBR1	Body	chr2:162279835-162280709	N_Shore
hypo213	FGR_adequate_3	1.1E-07	0.56	0.89	-0.33	3	101284875	cg10507762	RG9MTD1	3'UTR	chr3:101280545-101280952	S_Shelf
hypo214	FGR_adequate_3	4.4E-12	0.51	0.88	-0.37	5	131604900	cg21743925	PDLIM4;PDLIM4	Body;Body	chr5:131607018-131607889	N_Shelf
hypo215	FGR_adequate_3	2.9E-03	0.23	0.53	-0.29	5	176056682	cg07003542	SNCB;EIF4E1B;SNCB	5'UTR;TSS1500;5'UTR	chr5:176056520-176057494	Island
hypo216	FGR_adequate_3	1.8E-02	0.57	0.78	-0.22	6	12279872	cg17617182				
hypo217	FGR_adequate_3	2.6E-11	0.56	0.91	-0.35	6	15509388	cg04738028	JARID2	Body		
hypo218	FGR_adequate_3	5.6E-08	0.60	0.90	-0.30	6	28599165	cg05409752			chr6:28602853-28603295	N_Shelf
hypo219	FGR_adequate_3	6.7E-03	0.46	0.74	-0.28	6	28983079	cg16568131			chr6:28984418-28984686	N_Shore
hypo220	FGR_adequate_3	7.4E-03	0.48	0.84	-0.36	6	28983178	cg03656877			chr6:28984418-28984686	N_Shore
hypo221	FGR_adequate_3	6.0E-04	0.44	0.83	-0.39	6	43047193	cg23816594	PTK7;PTK7;PTK7;PTK7	Body;Body;Body;Body	chr6:43044044-43045057	S_Shelf
hypo222	FGR_adequate_3	3.9E-02	0.84	0.93	-0.08	6	64734868	cg23339291	EYS	Body		
hypo223	FGR_adequate_3	2.7E-13	0.56	0.93	-0.37	6	165751905	cg10855945	PDE10A;PDE10A	Body;Body	chr6:165747763-165747970	S_Shelf
hypo224	FGR_adequate_3	1.2E-11	0.48	0.87	-0.39	7	16890973	cg09185587			chr7:16890768-16891087	Island
hypo225	FGR_adequate_3	3.1E-02	0.48	0.72	-0.24	7	24762562	cg04317854	DFNA5;DFNA5;DFNA5	5'UTR;Body;Body		
hypo226	FGR_adequate_3	8.2E-02	0.22	0.60	-0.38	7	63497644	cg24729221			chr7:63498112-63498314	N_Shore
hypo227	FGR_adequate_3	1.4E-04	0.72	0.90	-0.17	8	23964970	cg11890659				
hypo228	FGR_adequate_3	3.6E-03	0.62	0.90	-0.28	8	23984071	cg10220494				
hypo229	FGR_adequate_3	4.7E-03	0.71	0.90	-0.18	8	93979580	cg02009601	C8orf83	TSS1500	chr8:93977793-93978469	S_Shore
hypo230	FGR_adequate_3	2.0E-06	0.58	0.81	-0.23	10	92153495	cg17905462				
hypo231	FGR_adequate_3	1.4E-04	0.90	0.97	-0.07	10	129145677	cg10214447	DOCK1	Body		
hypo232	FGR_adequate_3	5.0E-02	0.53	0.78	-0.25	10	134590772	cg12673559	INPP5A	Body	chr10:134593235-134593577	N_Shelf
hypo233	FGR_adequate_3	2.7E-02	0.64	0.89	-0.25	12	97956741	cg15028128	MIR135A2	TSS1500		
hypo234	FGR_adequate_3	6.0E-02	0.56	0.85	-0.29	14	86089857	cg13555362	FLRT2	3'UTR		
hypo235	FGR_adequate_3	7.9E-04	0.88	0.97	-0.09	14	101489721	cg02135464	MIR299;MIR411	TSS1500;Body		
hypo236	FGR_adequate_3	4.7E-04	0.41	0.85	-0.45	15	27418698	cg18181624	GABRG3	Body		
hypo237	FGR_adequate_3	2.3E-04	0.63	0.90	-0.27	15	102511865	cg21582633	WASH3P	Body		
hypo238	FGR_adequate_3	2.3E-13	0.49	0.86	-0.37	16	787071	cg17710440	NARFL	Body	chr16:787070-787327	Island
hypo239	FGR_adequate_3	1.3E-09	0.49	0.89	-0.41	16	47216988	cg16543943	ITFG1	Body		
hypo240	FGR_adequate_3	2.4E-03	0.51	0.89	-0.37	16	65850868	cg03515735				
hypo241	FGR_adequate_3	4.1E-09	0.37	0.88	-0.51	16	68560845	cg05798111			chr16:68563759-68564281	N_Shelf
hypo242	FGR_adequate_3	2.9E-02	0.42	0.65	-0.23	17	80621636	cg02531787	RAB40B	Body	chr17:80621841-80622060	N_Shore
hypo243	FGR_adequate_3	9.3E-11	0.50	0.87	-0.37	17	80986949	cg07092293	B3GNTL1	Body	chr17:80989059-80989392	N_Shelf
hypo244	FGR_adequate_3	8.3E-04	0.09	0.63	-0.53	18	55019843	cg13096208	ST8SIA3;ST8SIA3	1stExon;5'UTR	chr18:55019707-55021605	Island
hypo245	FGR_adequate_3	2.0E-02	0.50	0.89	-0.39	19	46112743	cg19874095	EML2	3'UTR		
hypo246	FGR_adequate_3	9.1E-04	0.30	0.51	-0.20	21	42218932	cg09880551	DSCAM;DSCAM	1stExon;5'UTR	chr21:42218489-42219222	Island
hypo247	FGR_adequate_3	3.0E-02	0.14	0.51	-0.37	21	42219193	cg07962392	DSCAM	TSS200	chr21:42218489-42219222	Island
hypo248	FGR_adequate_4	8.4E-08	0.65	0.90	-0.25	4	119696553	cg06645772	SEC24D	Body		
hypo249	FGR_adequate_4	1.8E-03	0.65	0.81	-0.16	4	184320560	cg07824907			chr4:184319564-184320198	S_Shore
hypo250	FGR_adequate_4	3.3E-02	0.80	0.94	-0.15	4	184320640	cg02831900			chr4:184319564-184320198	S_Shore
hypo251	FGR_adequate_4	3.3E-10	0.61	0.88	-0.27	4	186666487	cg11207127	SORBS2;SORBS2;SORBS2;SOR	5'UTR;5'UTR;5'UTR;5'UTR;5'UTR;5'UTR		
hypo252	FGR_adequate_4	6.5E-08	0.62	0.89	-0.27	5	134642951	cg06593237				
hypo253	FGR_adequate_4	1.1E-05	0.69	0.90	-0.21	5	159691948	cg22005656	CCNJL	Body		

hypo254	FGR_adequate_4	6.4E-10	0.72	0.94	-0.22	6	146051934	cg00077566	EPM2A;EPM2A	Body;Body	chr6:146055896-146056981	N_Shelf
hypo255	FGR_adequate_4	2.1E-10	0.75	0.97	-0.22	10	99278449	cg10989317	UBTD1	Body		
hypo256	FGR_adequate_4	2.1E-09	0.22	0.56	-0.34	11	2019732	cg11735853	H19	TSS1500	chr11:2019565-2019863	Island
hypo257	FGR_adequate_4	3.1E-06	0.60	0.83	-0.22	12	64360878	cg26987521	SRGAP1	Body		
hypo258	FGR_adequate_4	5.5E-04	0.84	0.97	-0.12	12	124798985	cg18124134	FAM101A	Body	chr12:124798796-124799224	Island
hypo259	FGR_adequate_4	6.3E-07	0.62	0.87	-0.25	16	2794914	cg16392797	LOC100128788;LOC100128788	TSS200;Body		
hypo260	FGR_adequate_4	4.4E-06	0.59	0.85	-0.26	16	89620074	cg16677432	SPG7	Body		
hypo261	FGR_adequate_4	3.3E-09	0.65	0.92	-0.27	17	80902556	cg07431961	B3GNTL1	3'UTR	chr17:80898985-80899313	S_Shelf
hypo262	FGR_adequate_5	8.2E-05	0.54	0.79	-0.25	1	17329620	cg05064739	ATP13A2;ATP13A2;ATP13A2	Body;Body;Body	chr1:17331885-17332093	N_Shelf
hypo263	FGR_adequate_5	7.3E-11	0.56	0.84	-0.28	1	19209709	cg07785791	ALDH4A1;ALDH4A1;ALDH4A1	Body;Body;Body	chr1:19209626-19209914	Island
hypo264	FGR_adequate_5	2.2E-08	0.60	0.87	-0.26	1	36931973	cg11577454	CSF3R;CSF3R;CSF3R;CSF3R	Body;3'UTR;Body;Body	chr1:36929414-36930131	S_Shore
hypo265	FGR_adequate_5	5.2E-02	0.63	0.84	-0.22	6	119252835	cg26771384	MCM9	Body	chr6:119255738-119256331	N_Shelf
hypo266	FGR_adequate_5	1.8E-06	0.68	0.91	-0.23	6	143891455	cg20298778	LOC285740	TSS1500		
hypo267	FGR_adequate_5	7.3E-02	0.61	0.82	-0.22	10	127779842	cg24194674	ADAM12;ADAM12	Body;Body		
hypo268	FGR_adequate_5	3.3E-08	0.65	0.88	-0.23	11	977931	cg27445792	AP2A2	Body	chr11:976949-977193	S_Shore
hypo269	FGR_adequate_5	1.9E-05	0.53	0.77	-0.24	11	2423571	cg27135510	TSSC4;TSSC4	5'UTR;1stExon	chr11:2421040-2422474	S_Shore
hypo270	FGR_adequate_5	4.5E-09	0.64	0.91	-0.27	11	92577553	cg24168798	FAT3	Body		
hypo271	FGR_adequate_5	3.0E-10	0.68	0.93	-0.25	12	3950698	cg07023594	PARP11	Body	chr12:3947922-3948620	S_Shelf
hypo272	FGR_adequate_5	3.9E-07	0.57	0.79	-0.23	14	24032626	cg04882359	AP1G2	Body	chr14:24036399-24037076	N_Shelf
hypo273	FGR_adequate_5	7.0E-04	0.69	0.91	-0.22	14	71250264	cg01022840	MAP3K9	Body		
hypo274	FGR_adequate_5	4.4E-03	0.81	0.90	-0.08	15	25301930	cg20568038	SNORD116-3;SNORD116-9	TSS200;TSS200		
hypo275	FGR_adequate_5	2.4E-04	0.88	0.96	-0.09	16	1706201	cg04908586	CRAMP1L	Body	chr16:1705866-1706244	Island
hypo276	FGR_adequate_5	9.6E-03	0.48	0.73	-0.25	16	3613509	cg02719221	NLRC3	Body	chr16:3613100-3613546	Island
hypo277	FGR_adequate_5	3.8E-04	0.69	0.89	-0.21	17	78748471	cg00523683	RPTOR;RPTOR	Body;Body		
hypo278	FGR_adequate_5	1.0E-06	0.54	0.91	-0.37	19	53269486	cg05619639	ZNF600	Body		
hypo279	FGR_adequate_5	9.7E-04	0.69	0.91	-0.22	22	39080524	cg05816914	TOMM22	3'UTR	chr22:39077816-39078400	S_Shelf
hypo280	FGR_excessive_1	1.3E-02	0.71	0.89	-0.18	1	6339954	cg11690194	ACOT7;ACOT7;ACOT7;ACOT7	Body;Body;Body;Body	chr1:6339948-6340268	Island
hypo281	FGR_excessive_1	2.1E-11	0.52	0.87	-0.35	1	9005474	cg23738308	CA6	TSS1500		
hypo282	FGR_excessive_1	1.7E-02	0.62	0.85	-0.24	2	183123847	cg07496624	PDE1A;PDE1A	Body;Body		
hypo283	FGR_excessive_1	4.3E-03	0.73	0.89	-0.15	2	183498282	cg26192279				
hypo284	FGR_excessive_1	5.3E-03	0.60	0.79	-0.19	2	192891657	cg22742965	TMEFF2	Body		
hypo285	FGR_excessive_1	1.1E-11	0.25	0.77	-0.52	2	237028869	cg13018278	AGAP1;AGAP1	Body;Body	chr2:237028848-237029121	Island
hypo286	FGR_excessive_1	5.6E-07	0.57	0.84	-0.27	3	126744035	cg13631725	PLXNA1	Body		
hypo287	FGR_excessive_1	2.5E-02	0.37	0.65	-0.29	3	196908930	cg12594803	DLG1;DLG1	Body;Body		
hypo288	FGR_excessive_1	7.2E-02	0.83	0.92	-0.08	4	295862	cg20241375			chr4:298803-299312	N_Shelf
hypo289	FGR_excessive_1	1.1E-04	0.49	0.81	-0.32	4	119826303	cg08774280	SYNPO2;SYNPO2;SYNPO2	Body;Body;Body		
hypo290	FGR_excessive_1	9.6E-04	0.63	0.88	-0.25	4	138468381	cg14017444				
hypo291	FGR_excessive_1	8.1E-02	0.63	0.87	-0.24	4	138535339	cg11920765				
hypo292	FGR_excessive_1	8.5E-03	0.79	0.92	-0.13	4	140619779	cg21634993	MGST2	Body	chr4:140619547-140619780	Island
hypo293	FGR_excessive_1	1.4E-02	0.72	0.88	-0.16	5	41071990	cg01900397	HEATR7B2	TSS1500		
hypo294	FGR_excessive_1	2.3E-04	0.65	0.89	-0.24	5	150054944	cg15699693	MYOZ3;MYOZ3	Body;Body	chr5:150051116-150052107	S_Shelf
hypo295	FGR_excessive_1	1.6E-11	0.57	0.87	-0.31	6	36462140	cg13528479	STK38	3'UTR		
hypo296	FGR_excessive_1	1.9E-02	0.19	0.60	-0.41	6	37617124	cg00807871	MDGA1	Body	chr6:37616722-37617179	Island
hypo297	FGR_excessive_1	1.3E-14	0.52	0.92	-0.39	6	107021926	cg25216662	RTN4IP1	Body		
hypo298	FGR_excessive_1	4.5E-05	0.44	0.68	-0.24	7	2261337	cg12577003	MAD1L1;MAD1L1;MAD1L1	Body;Body;Body		
hypo299	FGR_excessive_1	2.8E-11	0.54	0.90	-0.36	7	108055705	cg06002804	NRCAM;NRCAM	5'UTR;5'UTR		
hypo300	FGR_excessive_1	3.7E-05	0.57	0.83	-0.26	7	142659378	cg26806603	KEL;KEL	1stExon;5'UTR		
hypo301	FGR_excessive_1	1.4E-02	0.76	0.94	-0.17	7	158883968	cg23540020	VIPR2	Body	chr7:158886367-158886595	N_Shelf
hypo302	FGR_excessive_1	2.2E-02	0.64	0.83	-0.20	8	61227705	cg10796022				
hypo303	FGR_excessive_1	6.0E-05	0.50	0.80	-0.30	10	3280494	cg04678570				
hypo304	FGR_excessive_1	2.6E-05	0.51	0.85	-0.34	10	87426906	cg15209053	GRID1	Body		
hypo305	FGR_excessive_1	1.7E-11	0.52	0.84	-0.32	10	126685689	cg07341129	CTBP2;CTBP2;CTBP2	Body;Body;Body	chr10:126686551-126686892	N_Shore
hypo306	FGR_excessive_1	9.4E-10	0.60	0.92	-0.32	10	128932004	cg22909333	DOCK1	Body		
hypo307	FGR_excessive_1	2.1E-05	0.77	0.90	-0.13	11	78380599	cg12051260	ODZ4	Body		
hypo308	FGR_excessive_1	2.4E-02	0.73	0.84	-0.11	11	83372714	cg27373604	DLG2;DLG2;DLG2;DLG2	5'UTR;Body;Body;Body		
hypo309	FGR_excessive_1	1.3E-11	0.52	0.87	-0.35	11	105010531	cg20120939	CARD18	TSS200		
hypo310	FGR_excessive_1	1.6E-04	0.49	0.84	-0.34	11	117063002	cg27227371	SIDT2	Body		
hypo311	FGR_excessive_1	4.3E-02	0.74	0.89	-0.15	11	130536640	cg08193293				
hypo312	FGR_excessive_1	1.2E-05	0.53	0.81	-0.27	12	30845811	cg19250083	IPO8	Body	chr12:30848264-30849016	N_Shelf
hypo313	FGR_excessive_1	3.2E-02	0.78	0.88	-0.10	12	89118099	cg09154414				
hypo314	FGR_excessive_1	4.2E-08	0.81	0.95	-0.14	12	96389483	cg09636302	HAL	Body	chr12:96389405-96389675	Island
hypo315	FGR_excessive_1	3.7E-09	0.57	0.87	-0.31	12	109046825	cg06251181	CORO1C	Body		
hypo316	FGR_excessive_1	5.9E-03	0.50	0.82	-0.32	14	51137626	cg18406010			chr14:51134480-51135304	S_Shelf
hypo317	FGR_excessive_1	2.1E-14	0.65	0.94	-0.29	14	105836263	cg18853010	PACS2;PACS2	Body;Body	chr14:105836031-105836386	Island
hypo318	FGR_excessive_1	8.6E-06	0.47	0.74	-0.27	15	101645225	cg02346706				
hypo319	FGR_excessive_1	9.6E-07	0.65	0.87	-0.21	16	88032157	cg10637565	BANP;BANP	Body;Body	chr16:88032156-88034968	Island
hypo320	FGR_excessive_1	2.3E-02	0.91	0.98	-0.07	16	88550450	cg02071579	ZFPM1	Body	chr16:88554354-88554847	N_Shelf
hypo321	FGR_excessive_1	3.4E-03	0.92	0.98	-0.06	16	89177789	cg04388767	ACSF3;ACSF3;ACSF3	Body;Body;Body	chr16:89173704-89173905	S_Shelf
hypo322	FGR_excessive_1	4.5E-03	0.80	0.96	-0.16	16	89187106	cg26565884	ACSF3;ACSF3;ACSF3	Body;Body;Body	chr16:89186198-89186551	S_Shore
hypo323	FGR_excessive_1	2.3E-03	0.97	0.99	-0.02	17	27903940	cg06013127	GIT1;GIT1	Body;Body	chr17:27899511-27900067	S_Shelf
hypo324	FGR_excessive_1	1.4E-03	0.61	0.83	-0.22	17	55980107	cg15979932	CUEDC1	5'UTR		
hypo325	FGR_excessive_1	2.9E-06	0.70	0.90	-0.19	17	80540666	cg15014069	FOXK2	Body	chr17:80541548-80541754	N_Shore
hypo326	FGR_excessive_1	7.0E-02	0.57	0.79	-0.22	17	80540696	cg09697578	FOXK2	Body	chr17:80541548-80541754	N_Shore
hypo327	FGR_excessive_1	2.2E-05	0.78	0.90	-0.13	17	80542006	cg26986438	FOXK2	Body	chr17:80541912-80542119	Island
hypo328	FGR_excessive_1	1.2E-02	0.74	0.89	-0.14	17	80559449	cg13360263	FOXK2	3'UTR	chr17:80559025-80559450	Island
hypo329	FGR_excessive_1	6.2E-02	0.37	0.62	-0.25	18	32957127	cg00647528	ZNF396	5'UTR	chr18:32956764-32957406	Island
hypo330	FGR_excessive_1	5.1E-03	0.87	0.97	-0.09	20	61472147	cg06598544	COL9A3	3'UTR	chr20:61471903-61472256	Island
hypo331	FGR_excessive_1	6.3E-03	0.61	0.89	-0.28	21	34957259	cg08911536	DONSON	Body	chr21:34960388-34961252	N_Shelf
hypo332	FGR_excessive_2	1.1E-04	0.82	0.97	-0.15	1	5943388	cg26791536	NPHP4	Body		
hypo333	FGR_excessive_2	3.0E-05	0.76	0.95	-0.20	1	5950833	cg21451253	NPHP4	Body	chr1:5950824-5951029	Island
hypo334	FGR_excessive_2	2.2E-05	0.82	0.94	-0.12	1	5951028	cg05463119	NPHP4	Body	chr1:5950824-5951029	Island
hypo335	FGR_excessive_2	7.6E-02	0.66	0.91	-0.24	1	5955929	cg15598789	NPHP4	Body		
hypo336	FGR_excessive_2	7.2E-10	0.47	0.80	-0.33	1	27185941	cg10817250			chr1:27189912-27190128	N_Shelf
hypo337	FGR_excessive_2	1.8E-08	0.52	0.85	-0.33	1	160769081	cg07106927	LY9;LY9	Body;Body	chr1:160771683-160771953	N_Shelf
hypo338	FGR_excessive_2	5.4E-05	0.68	0.92	-0.24	2	219754840	cg26449951	WNT10A	Body	chr2:219757458-219758001	N_Shelf
hypo339	FGR_excessive_2	9.1E-12	0.53	0.90	-0.37	2	231584368	cg20724690	CAB39;CAB39;CAB39	5'UTR;5'UTR;5'UTR		
hypo340	FGR_excessive_2	4.5E-02	0.75	0.96	-0.21	4	3735986	cg03469578				
hypo341	FGR_excessive_2	7.2E-13	0.56	0.92	-0.36	6	32934046	cg00326788			chr6:32935896-32936792	N_Shore
hypo342	FGR_excessive_2	3.9E-14	0.56	0.91	-0.34	6	41913381	cg16323359	CCND3;CCND3	5'UTR;5'UTR	chr6:41908745-41909711	S_Shelf
hypo343	FGR_excessive_2	8.4E-02	0.37	0.59	-0.21	6	43230751	cg25020820	TTBK1	Body	chr6:43230610-43231064	Island
hypo344	FGR_excessive_2	3.1E-03	0.55	0.82	-0.27	6	129768405	cg23563733	LAMA2;LAMA2	Body;Body		
hypo345	FGR_excessive_2	4.3E-04	0.56	0.82	-0.26	7	23627109	cg23711139	CLK2P	TSS1500	chr7:23625611-23626161	S_Shore
hypo346	FGR_excessive_2	5.9E-06	0.08	0.55	-0.48	7	71801793	cg05223720	CALN1;CALN1;CALN1	5'UTR;1stExon;Body	chr7:71800757-71802768	Island
hypo347	FGR_excessive_2	5.2E-03	0.14	0.72	-0.59	7	139989127	cg14876463			chr7:139992321-139992574	N_Shelf
hypo348	FGR_excessive_2	1.1E-02	0.45	0.68	-0.23	9	136655966	cg14415079	VAV2;VAV2	Body;Body	chr9:136659111-136659329	N_Shelf
hypo349	FGR_excessive_2	4.3E-13	0.54	0.90	-0.36	10	61596106	cg09082518	CCDC6	Body		
hypo350	FGR_excessive_2	2.3E-11	0.55	0.89	-0.35	10	118647000	cg19007019	KIAA1598;KIAA1598	Body;Body		
hypo351	FGR_excessive_2	1.4E-04	0.56	0.88	-0.31	11	10469317	cg09403480			chr11:10472000-10472857	N_Shelf
hypo352	FGR_excessive_2	5.6E-09	0.46	0.78	-0.32	11	69462783	cg27021553	CCND1	Body	chr11:69461060-69461378	S_Shore
hypo353	FGR_excessive_2	6.5E-10	0.56	0.88	-0.32	12	93131197	cg15092825	PLEKHG7	5'UTR		
hypo354	FGR_excessive_2	6.7E-13	0.54	0.88	-0.35	12	123351126	cg25957092	VPS37B	3'UTR	chr12:123351641-123351918	N_Shore
hypo355	FGR_excessive_2	1.4E-02	0.43	0.72	-0.30	13	52598798	cg25548582	UTP14C;ALG11	TSS200;Body		
hypo356	FGR_excessive_2	2.1E-02	0.34	0.49	-0.15	15	91423337	cg17396638	FURIN	Body	chr15:91423329-91423583	Island
hypo357	FGR_excessive_2	1.7E-06	0.74	0.89	-0.15	16	3515991	cg06434344	NAT15;NAT15;NAT15	5'UTR;5'UTR;5'UTR		
hypo358	FGR_excessive_2	4.1E-05	0.63	0.83	-0.20	16	3516099	cg01055099	NAT15;NAT15;NAT15	5'UTR;5'UTR;5'UTR		
hypo359	FGR_excessive_2	1.2E-03	0.42	0.74	-0.32	16	73126120	cg00293191	HTA	TSS200		
hypo360	FGR_excessive_2	1.3E-08	0.50	0.86	-0.35	16	88558237	cg05958985	ZFPM1	Body	chr16:88558051-88558329	Island
hypo361	FGR_excessive_2	1.8E-15	0.62	0.96	-0.34	16	89926104	cg27461200	SPIRE2	Body		
hypo362	FGR_excessive_2	4.1E-10	0.82	0.97	-0.15	17	4792905	cg20330086	MINK1;MINK1;MINK1;MINK1	Body;Body;Body;Body		
hypo363	FGR_excessive_2	2.3E-07	0.70	0.94	-0.24	20	25174319	cg20840926			chr20:25176027-25176620	N_Shore
hypo364	FGR_excessive_2	2.2E-05	0.75	0.93	-0.19	20	62317640	cg27508960	RTEL1;RTEL1	Body;Body	chr20:62317505-62317775	Island
hypo365	FGR_excessive_2	3.1E-03	0.72	0.86	-0.14	22	39778604	cg17537935	SYNGR1	3'UTR	chr22:39777665-39777887	S_Shore
hypo366	FGR_excessive_3	1.0E-09	0.53	0.81	-0.28	1	54620626	cg01060188	CDCP2	TSS1500		
hypo367	FGR_excessive_3	9.8E-05	0.55	0.77	-0.22	1	153522407	cg17107867	S100A3	TSS1500		
hypo368	FGR_excessive_3	4.6E-05	0.60	0.87	-0.27	1	226733079	cg13400284			chr1:226736354-226737412	N_Shelf
hypo369	FGR_excessive_3	5.7E-06	0.52	0.83	-0.31	2	69304921	cg05997278	ANTXR1;ANTXR1;ANTXR1	Body;Body;Body		
hypo370	FGR_excessive_3	3.2E-06	0.47	0.80	-0.33	2	128430937	cg04947052	LIMS2;LIMS2;LIMS2	Body;Body;5'UTR	chr2:128432194-128432743	N_Shore
hypo371	FGR_excessive_3	5.3E-07	0.58	0.89	-0.31	2	206139894	cg16851221	PARD3B;PARD3B;PARD3B	Body;Body;Body		
hypo372	FGR_excessive_3	2.5E-04	0.49	0.84	-0.35	2	242664024	cg22077686	ING5	Body	chr2:242664207-242665154	N_Shore
hypo373	FGR_excessive_3	1.0E-02	0.48	0.76	-0.28	3	12184833	cg16052972	SYN2;SYN2	Body;Body		
hypo374	FGR_excessive_3	2.1E-10	0.54	0.89	-0.35	3	50267581	cg06157116			chr3:50263685-50263908	S_Shelf
hypo375	FGR_excessive_3	3.6E-07	0.25	0.56	-0.31	3	182816738	cg25441771	MCCC1	Body	chr3:182816771-182817455	N_Shore
hypo376	FGR_excessive_3	3.8E-06	0.38	0.64	-0.26	3	182817190	cg02616966	MCCC1	1stExon	chr3:182816771-182817455	Island
hypo377	FGR_excessive_3	6.7E-02	0.21	0.39	-0.18	3	182817479	cg08395365	MCCC1	TSS200	chr3:182816771-182817455	S_Shore
hypo378	FGR_excessive_3	1.6E-04	0.22	0.50	-0.27	3	182817578	cg23166970	MCCC1	TSS1500	chr3:182816771-182817455	S_Shore
hypo379	FGR_excessive_3	2.2E-04	0.20	0.44	-0.24	3	182817584	cg22211233	MCCC1	TSS1500	chr3:182816771-182817455	S_Shore
hypo380	FGR_excessive_3	3.8E-04	0.31	0.58	-0.27	3	182817626	cg03344955	MCCC1	TSS1500	chr3:182816771-182817455	S_Shore
hypo381	FGR_excessive_3	1.3E-12	0.57	0.90	-0.33	5	39398185	cg21886364	DAB2	5'UTR		
hypo382	FGR_excessive_3	4.7E-02	0.47	0.75	-0.29	6	52012390	cg14487577	MIR133B	TSS1500		

hypo383	FGR_excessive_3	7.1E-09	0.04	0.48	-0.44	7	151328924	cg18334211	PRKAG2;PRKAG2;PRKAG2	Body;Body;Body		
hypo384	FGR_excessive_3	6.6E-03	0.32	0.56	-0.24	7	151330008	cg01108370	PRKAG2;PRKAG2;PRKAG2	Body;TSS1500;Body		
hypo385	FGR_excessive_3	9.2E-09	0.57	0.89	-0.31	10	72113560	cg09504384	LRRC20;LRRC20;LRRC20	Body;Body;Body		
hypo386	FGR_excessive_3	5.5E-08	0.65	0.90	-0.25	11	35817123	cg07271405	TRIM44	Body		
hypo387	FGR_excessive_3	4.3E-07	0.70	0.82	-0.12	16	84693437	cg09145053	KLHL36	Body	chr16:84695169-84695681	N_Shore
hypo388	FGR_excessive_3	1.3E-06	0.47	0.75	-0.28	17	58165865	cg03120558	LOC645638	TSS200		
hypo389	FGR_excessive_4	1.2E-02	0.52	0.79	-0.27	1	167902728	cg19777644	BRP44;BRP44;BRP44	Body;Body;Body	chr1:167905027-167906801	N_Shelf
hypo390	FGR_excessive_4	1.4E-05	0.36	0.67	-0.31	1	203605590	cg13808058	ATP2B4;ATP2B4	5'UTR;5'UTR		
hypo391	FGR_excessive_4	2.4E-15	0.55	0.91	-0.35	3	39429575	cg14528273	SLC25A38	Body	chr3:39424696-39426058	S_Shelf
hypo392	FGR_excessive_4	3.3E-03	0.62	0.90	-0.28	3	48705266	cg13239227			chr3:48698335-48701667	S_Shelf
hypo393	FGR_excessive_4	6.6E-12	0.56	0.92	-0.36	3	64080488	cg05799088	PRICKLE2	3'UTR		
hypo394	FGR_excessive_4	7.7E-13	0.25	0.81	-0.56	3	119420208	cg25188166			chr3:119421855-119422334	N_Shore
hypo395	FGR_excessive_4	1.4E-06	0.64	0.90	-0.26	4	74854214	cg21398186	PPBP	TSS1500		
hypo396	FGR_excessive_4	1.2E-09	0.78	0.96	-0.18	5	1325969	cg26947723	CLPTM1L	Body		
hypo397	FGR_excessive_4	4.5E-10	0.86	0.97	-0.11	5	138737437	cg11570383	SPATA24	Body	chr5:138739492-138739912	N_Shelf
hypo398	FGR_excessive_4	4.8E-02	0.79	0.89	-0.10	6	13365510	cg14949271	GFOD1	Body	chr6:13364999-13365949	Island
hypo399	FGR_excessive_4	3.3E-04	0.75	0.90	-0.15	6	13365826	cg26738980	GFOD1	Body	chr6:13364999-13365949	Island
hypo400	FGR_excessive_4	1.9E-14	0.53	0.90	-0.37	6	101306732	cg09392911	ASCC3;ASCC3	3'UTR;Body		
hypo401	FGR_excessive_4	1.5E-02	0.85	0.93	-0.08	8	104315598	cg14645926	FZD6;FZD6;FZD6	Body;Body;Body	chr8:104310809-104311620	S_Shelf
hypo402	FGR_excessive_4	4.1E-08	0.43	0.78	-0.35	8	140999001	cg25699417	TRAPPC9;TRAPPC9	Body;Body		
hypo403	FGR_excessive_4	6.6E-16	0.13	0.84	-0.71	9	99264127	cg14155501	CDC14B;CDC14B;CDC14B	3'UTR;3'UTR;3'UTR		
hypo404	FGR_excessive_4	7.6E-02	0.74	0.92	-0.18	10	81444403	cg10452333	LOC650623	Body	chr10:81444163-81444474	Island
hypo405	FGR_excessive_4	2.8E-11	0.49	0.86	-0.37	10	125624685	cg01512466	CPXM2	Body		
hypo406	FGR_excessive_4	2.3E-02	0.57	0.84	-0.27	11	69949353	cg15767218	ANO1;ANO1	Body;Body		
hypo407	FGR_excessive_4	2.0E-11	0.51	0.88	-0.37	12	76277069	cg15007809				
hypo408	FGR_excessive_4	4.7E-06	0.71	0.92	-0.21	13	31732963	cg02309157	HSPH1	Body	chr13:31735109-31736670	N_Shelf
hypo409	FGR_excessive_4	3.2E-08	0.72	0.91	-0.19	15	25415399	cg22241833	SNORD115-1	TSS1500		
hypo410	FGR_excessive_4	4.5E-10	0.57	0.89	-0.32	17	15415649	cg15374027	FAM18B2	Body		
hypo411	FGR_excessive_4	1.3E-02	0.43	0.66	-0.23	17	16381219	cg11624993	C17orf76;C17orf76	Body;Body		
hypo412	FGR_excessive_4	8.0E-05	0.32	0.92	-0.61	17	78093132	cg11469818	GAA;GAA;GAA	3'UTR;3'UTR;3'UTR		
hypo413	FGR_excessive_4	7.1E-04	0.54	0.81	-0.27	18	60260226	cg07429775			chr18:60263380-60264248	N_Shelf
hypo414	FGR_excessive_4	2.1E-02	0.69	0.88	-0.19	19	2586206	cg04696980	GNG7	5'UTR	chr19:2588375-2588590	N_Shelf
hypo415	FGR_excessive_4	8.7E-02	0.86	0.97	-0.11	19	4956786	cg00033371	UHRF1;UHRF1	Body;Body	chr19:4954453-4954870	S_Shore
hypo416	FGR_excessive_4	5.5E-05	0.48	0.72	-0.25	19	10222409	cg18910313	PPAN-P2RY11;P2RY11	Body;1stExon	chr19:10220612-10221147	S_Shore
hypo417	FGR_excessive_4	8.7E-02	0.27	0.49	-0.22	22	46367703	cg13458651	WNT7B	Body	chr22:46366726-46368726	Island
hypo418	FGR_excessive_4	2.9E-04	0.43	0.75	-0.33	22	50990668	cg19800640	C22orf41	Body	chr22:50984972-50988141	S_Shelf
hypo419	N_insufficient_1	2.9E-13	0.57	0.93	-0.36	1	246942431	cg25350722				
hypo420	N_insufficient_1	1.3E-06	0.47	0.80	-0.33	2	98349537	cg27280787	ZAP70;ZAP70	Body;TSS1500	chr2:98350984-98351257	N_Shore
hypo421	N_insufficient_1	3.8E-06	0.44	0.75	-0.31	2	164471195	cg14448393	FIGN	Body		
hypo422	N_insufficient_1	1.5E-03	0.50	0.85	-0.35	4	6665095	cg09867452			chr4:6665671-6666089	N_Shore
hypo423	N_insufficient_1	4.3E-10	0.55	0.92	-0.37	4	56231727	cg08891829	SRD5A3	Body		
hypo424	N_insufficient_1	1.6E-05	0.59	0.88	-0.30	5	77917917	cg07228402	LHFPL2	5'UTR		
hypo425	N_insufficient_1	1.2E-11	0.51	0.86	-0.35	5	149115085	cg26876242	PPARGC1B	Body	chr5:149109570-149111750	S_Shelf
hypo426	N_insufficient_1	2.3E-04	0.12	0.78	-0.66	6	13560875	cg12487153				
hypo427	N_insufficient_1	7.3E-13	0.44	0.78	-0.34	6	30848807	cg06642647			chr6:30852102-30852676	N_Shelf
hypo428	N_insufficient_1	1.3E-02	0.50	0.72	-0.22	6	36492993	cg07986413	STK38	Body		
hypo429	N_insufficient_1	4.8E-10	0.62	0.91	-0.29	7	120455697	cg16969077	TSPAN12	Body		
hypo430	N_insufficient_1	1.1E-07	0.63	0.89	-0.27	7	150079014	cg15362480	ZNF775	5'UTR	chr7:150075939-150076856	S_Shelf
hypo431	N_insufficient_1	2.5E-05	0.50	0.84	-0.34	10	3600710	cg14380840			chr10:3598475-3598930	S_Shore
hypo432	N_insufficient_1	1.5E-05	0.09	0.89	-0.80	11	43391688	cg07515919	TTC17	Body		
hypo433	N_insufficient_1	3.3E-04	0.41	0.78	-0.37	15	65656635	cg13614617	IGDCC3	Body	chr15:65654077-65654334	S_Shelf
hypo434	N_insufficient_1	2.9E-05	0.77	0.93	-0.16	16	89161567	cg06419212	ACSF3;ACSF3;ACSF3	Body;5'UTR;5'UTR	chr16:89160018-89160706	S_Shore
hypo435	N_insufficient_1	8.0E-02	0.62	0.85	-0.23	16	89162196	cg01427313	ACSF3;ACSF3;ACSF3	Body;5'UTR;5'UTR	chr16:89160018-89160706	S_Shore
hypo436	N_insufficient_1	4.8E-02	0.65	0.89	-0.23	16	89165765	cg08316009	ACSF3;ACSF3;ACSF3	Body;5'UTR;5'UTR	chr16:89167116-89167482	N_Shore
hypo437	N_insufficient_1	5.2E-05	0.58	0.79	-0.20	16	89167154	cg27424853	ACSF3;ACSF3;ACSF3	Body;Body;Body	chr16:89167116-89167482	Island
hypo438	N_insufficient_1	3.7E-04	0.71	0.95	-0.24	16	89168371	cg01083549	ACSF3;ACSF3;ACSF3	Body;Body;Body	chr16:89167116-89167482	S_Shore
hypo439	N_insufficient_1	4.0E-06	0.66	0.92	-0.26	16	89168508	cg02098565	ACSF3;ACSF3;ACSF3	Body;Body;Body	chr16:89167116-89167482	S_Shore
hypo440	N_insufficient_1	2.8E-02	0.59	0.83	-0.24	16	89168599	cg04252928	ACSF3;ACSF3;ACSF3	Body;Body;Body	chr16:89167116-89167482	S_Shore
hypo441	N_insufficient_1	5.7E-05	0.64	0.86	-0.22	16	89168963	cg04069539	ACSF3;ACSF3;ACSF3	Body;Body;Body	chr16:89167116-89167482	S_Shore
hypo442	N_insufficient_1	1.2E-04	0.65	0.93	-0.28	16	89169744	cg01942849	ACSF3;ACSF3;ACSF3	Body;Body;Body	chr16:89167763-89167991	S_Shore
hypo443	N_insufficient_1	3.2E-03	0.88	0.98	-0.11	16	89173855	cg07116312	ACSF3;ACSF3;ACSF3	Body;Body;Body	chr16:89173704-89173905	Island
hypo444	N_insufficient_1	8.5E-06	0.69	0.91	-0.22	16	89174012	cg02204831	ACSF3;ACSF3;ACSF3	Body;Body;Body	chr16:89173704-89173905	S_Shore
hypo445	N_insufficient_1	3.7E-06	0.68	0.97	-0.29	16	89177293	cg00623015	ACSF3;ACSF3;ACSF3	Body;Body;Body	chr16:89173704-89173905	S_Shelf
hypo446	N_insufficient_1	7.3E-02	0.65	0.95	-0.30	16	89180742	cg06934654	ACSF3;ACSF3;ACSF3	Body;Body;Body		
hypo447	N_insufficient_1	6.4E-03	0.69	0.90	-0.21	16	89183453	cg03573961	ACSF3;ACSF3;ACSF3	Body;Body;Body	chr16:89186198-89186551	N_Shelf
hypo448	N_insufficient_1	8.3E-03	0.64	0.86	-0.21	16	89186454	cg00759803	ACSF3;ACSF3;ACSF3	Body;Body;Body	chr16:89186198-89186551	Island
hypo449	N_insufficient_1	4.3E-02	0.66	0.84	-0.19	16	89187002	cg07235885	ACSF3;ACSF3;ACSF3	Body;Body;Body	chr16:89186198-89186551	S_Shore
hypo450	N_insufficient_1	2.5E-03	0.68	0.88	-0.20	16	89189383	cg04033022	ACSF3;ACSF3;ACSF3	Body;Body;Body	chr16:89186198-89186551	S_Shelf
hypo451	N_insufficient_1	3.8E-02	0.82	0.92	-0.10	16	89202817	cg02747280	ACSF3;ACSF3;ACSF3	Body;Body;Body		
hypo452	N_insufficient_1	1.5E-06	0.75	0.94	-0.19	16	89203153	cg00144414	ACSF3;ACSF3;ACSF3	Body;Body;Body		
hypo453	N_insufficient_1	8.1E-04	0.70	0.94	-0.24	16	89205832	cg00739608	ACSF3;ACSF3;ACSF3	Body;Body;Body		
hypo454	N_insufficient_1	1.8E-03	0.67	0.91	-0.23	16	89217849	cg07882243	ACSF3;ACSF3;ACSF3	Body;Body;Body	chr16:89219333-89219651	N_Shore
hypo455	N_insufficient_1	1.6E-13	0.64	0.94	-0.30	17	80880952	cg02870816	TBCD	Body		
hypo456	N_insufficient_1	7.7E-13	0.52	0.89	-0.37	20	23584367	cg07473985	CST9	Body		
hypo457	N_insufficient_1	7.7E-12	0.60	0.91	-0.31	20	62492694	cg27272402	C20orf135;C20orf135	5'UTR;1stExon	chr20:62492849-62494249	N_Shore
hypo458	N_insufficient_2	1.9E-04	0.82	0.93	-0.12	1	5931389	cg26661089	NPHP4	Body		
hypo459	N_insufficient_2	1.8E-02	0.85	0.98	-0.13	1	5947386	cg16430402	NPHP4	Body	chr1:5950824-5951029	N_Shelf
hypo460	N_insufficient_2	1.1E-04	0.43	0.75	-0.32	1	12241073	cg05599723	TNFRSF1B	Body		
hypo461	N_insufficient_2	3.6E-04	0.47	0.73	-0.26	1	47688728	cg16006738	TAL1	Body	chr1:47690980-47691727	N_Shelf
hypo462	N_insufficient_2	9.4E-06	0.76	0.92	-0.16	2	54855588	cg01502208	SPTBN1;SPTBN1	Body;Body	chr2:54856098-54856487	N_Shore
hypo463	N_insufficient_2	1.3E-03	0.40	0.68	-0.28	3	32934782	cg22985281				
hypo464	N_insufficient_2	2.8E-10	0.50	0.81	-0.31	4	89299173	cg08684066	HERC6;HERC6	TSS1500;TSS1500	chr4:89299990-89300429	N_Shore
hypo465	N_insufficient_2	3.0E-07	0.90	0.97	-0.07	4	188924752	cg14725631	ZFP42	Body		
hypo466	N_insufficient_2	1.7E-10	0.60	0.91	-0.30	5	54749496	cg00235313	PPAP2A;PPAP2A	Body;Body		
hypo467	N_insufficient_2	7.5E-03	0.48	0.81	-0.33	5	110088130	cg02061660	SLC25A46	Body		
hypo468	N_insufficient_2	2.0E-07	0.61	0.90	-0.28	5	157115228	cg15122603			chr5:157117149-157118379	N_Shore
hypo469	N_insufficient_2	7.7E-11	0.51	0.88	-0.37	6	170593078	cg27614489	DLL1	Body	chr6:170592384-170593079	Island
hypo470	N_insufficient_2	2.0E-08	0.79	0.94	-0.14	7	76604421	cg22730072				
hypo471	N_insufficient_2	1.4E-04	0.44	0.73	-0.29	8	141559946	cg07701049	EIF2C2;EIF2C2	Body;Body	chr8:141559164-141559368	S_Shore
hypo472	N_insufficient_2	3.9E-05	0.59	0.89	-0.30	12	72094869	cg09055659	TMEM19	3'UTR		
hypo473	N_insufficient_2	5.1E-03	0.11	0.87	-0.76	12	133378609	cg18873965	GOLGA3	Body	chr12:133378384-133378638	Island
hypo474	N_insufficient_2	4.1E-07	0.09	0.56	-0.46	15	48819726	cg21011649	FBN1	Body		
hypo475	N_insufficient_2	3.2E-03	0.46	0.77	-0.31	15	94841112	cg02700491	MCTP2;MCTP2	TSS1500;TSS1500		
hypo476	N_insufficient_2	1.1E-14	0.54	0.88	-0.34	17	2266489	cg11881861	SGSM2;SGSM2	Body;Body	chr17:2265492-2265876	S_Shore
hypo477	N_insufficient_2	4.4E-07	0.52	0.82	-0.30	17	5982877	cg03747884	WSCD1	5'UTR		
hypo478	N_insufficient_2	5.6E-02	0.53	0.74	-0.20	19	56612425	cg01037768	ZNF787	Body	chr19:56612299-56612743	Island
hypo479	N_insufficient_2	7.2E-12	0.60	0.94	-0.34	20	18470310	cg02877738	RBBP9	3'UTR		
hypo480	N_insufficient_2	1.3E-02	0.49	0.69	-0.21	20	37055221	cg05082083	LOC388796;SNORA71B;LOC388	Body;TSS1500;Body		
hypo481	N_insufficient_2	6.1E-10	0.46	0.83	-0.37	22	46323087	cg07422735	WNT7B	Body	chr22:46326912-46327254	N_Shelf
hypo482	N_insufficient_3	7.0E-06	0.70	0.85	-0.16	1	899182	cg01930745	KLHL17	Body	chr1:894313-902654	Island
hypo483	N_insufficient_3	2.7E-05	0.44	0.73	-0.29	1	2198760	cg01101178	SKI	Body		
hypo484	N_insufficient_3	7.4E-11	0.56	0.87	-0.30	2	224896241	cg14256643	SERPINE2;SERPINE2;SERPINE2	TSS200;5'UTR;Body;5'UTR		
hypo485	N_insufficient_3	1.4E-15	0.13	0.91	-0.79	2	230928682	cg00483994	SLC16A14	5'UTR	chr2:230932468-230933550	N_Shelf
hypo486	N_insufficient_3	5.3E-04	0.72	0.90	-0.18	2	242691497	cg27590905	D2HGDH	Body		
hypo487	N_insufficient_3	4.7E-05	0.48	0.73	-0.25	3	135774360	cg15210622	PPP2R3A;PPP2R3A	Body;Body		
hypo488	N_insufficient_3	7.6E-02	0.85	0.94	-0.08	4	3233786	cg08072487	HTT	Body		
hypo489	N_insufficient_3	2.9E-09	0.63	0.93	-0.31	6	3800707	cg08433051				
hypo490	N_insufficient_3	7.9E-07	0.54	0.85	-0.30	6	7401622	cg06821182	RIOK1;RIOK1	TSS1500;Body		
hypo491	N_insufficient_3	2.0E-13	0.54	0.90	-0.36	6	31760345	cg15864153	VARS	Body	chr6:31763240-31763905	N_Shelf
hypo492	N_insufficient_3	4.8E-06	0.47	0.85	-0.38	7	70096029	cg04601228	AUTS2;AUTS2	Body;Body	chr7:70096263-70096501	N_Shore
hypo493	N_insufficient_3	2.9E-07	0.59	0.87	-0.28	7	129988860	cg15649111	CPA5;CPA5;CPA5	Body;Body;Body		
hypo494	N_insufficient_3	7.5E-04	0.15	0.54	-0.39	10	43186868	cg17575113				
hypo495	N_insufficient_3	4.4E-08	0.51	0.85	-0.34	10	69872166	cg07824800	MYPN	5'UTR		
hypo496	N_insufficient_3	1.4E-10	0.79	0.97	-0.18	10	135102440	cg12010544	TUBGCP2	Body	chr10:135103295-135103554	N_Shore
hypo497	N_insufficient_3	4.1E-02	0.33	0.50	-0.17	14	69203665	cg23399279				
hypo498	N_insufficient_3	5.3E-12	0.49	0.86	-0.37	16	3543548	cg06592080	C16orf90	3'UTR	chr16:3546366-3546769	N_Shelf
hypo499	N_insufficient_3	8.1E-09	0.58	0.92	-0.34	16	77397865	cg01737667	ADAMTS18	Body		
hypo500	N_insufficient_3	4.8E-05	0.62	0.86	-0.24	17	35685086	cg12184450	ACACA;ACACA;ACACA	Body;Body;Body		
hypo501	N_insufficient_3	2.2E-02	0.15	0.87	-0.72	19	19306927	cg17726666	RFXANK;RFXANK	Body;Body	chr19:19302854-19303148	S_Shelf
hypo502	N_insufficient_3	3.7E-13	0.50	0.88	-0.38	19	20608532	cg07744432	ZNF826	TSS1500		
hypo503	N_insufficient_3	2.2E-10	0.60	0.86	-0.27	20	61455419	cg07400741	COL9A3	Body	chr20:61455418-61455747	Island
hypo504	N_insufficient_4	1.5E-08	0.46	0.79	-0.33	1	1600982	cg25283841	LOC728661;CDK11B;CDK11B;CI	Body;Body;Body;Body;Body;Body;	chr1:1600981-1601231	Island
hypo505	N_insufficient_4	7.1E-12	0.59	0.92	-0.33	1	27142951	cg11712962			chr1:27143740-27143953	N_Shore
hypo506	N_insufficient_4	2.0E-05	0.42	0.71	-0.29	1	37952273	cg24428600				
hypo507	N_insufficient_4	1.2E-04	0.59	0.91	-0.32	1	83452405	cg18411040				
hypo508	N_insufficient_4	2.7E-09	0.49	0.85	-0.36	1	85755348	cg16596317				
hypo509	N_insufficient_4	2.4E-12	0.51	0.88	-0.37	2	97365990	cg12909445	FER1L5;FER1L5	Body;Body		
hypo510	N_insufficient_4	4.9E-13	0.60	0.92	-0.31	3	133751875	cg05046771			chr3:133747920-133748266	S_Shelf
hypo511	N_insufficient_4	7.8E-02	0.40	0.66	-0.27	5	116113889	cg07837187				

hypo512	N_insufficient_4	3.3E-06	0.87	0.97	-0.10	6	30153882	cg16012536	TRIM26	Body		
hypo513	N_insufficient_4	2.7E-04	0.79	0.90	-0.11	6	30157507	cg24443629	TRIM26	Body		
hypo514	N_insufficient_4	2.6E-03	0.83	0.94	-0.11	6	30158249	cg04110164	TRIM26	Body		
hypo515	N_insufficient_4	6.9E-02	0.78	0.87	-0.10	6	30164320	cg01782500	TRIM26	Body		
hypo516	N_insufficient_4	5.3E-02	0.79	0.90	-0.11	6	30166517	cg18901633	TRIM26	Body		
hypo517	N_insufficient_4	1.4E-02	0.78	0.87	-0.09	6	30172433	cg22272129	TRIM26	5'UTR		
hypo518	N_insufficient_4	1.2E-06	0.64	0.93	-0.28	6	74126975	cg24886843	DDX43	3'UTR		
hypo519	N_insufficient_4	3.8E-02	0.89	0.97	-0.07	7	4245514	cg26734522	SDK1;SDK1	Body;Body		
hypo520	N_insufficient_4	1.6E-05	0.10	0.47	-0.37	7	43153223	cg17045801	HECW1	5'UTR	chr7:43152020-43153340	Island
hypo521	N_insufficient_4	8.4E-02	0.82	0.97	-0.15	8	39380341	cg01911981	ADAM3A;ADAM3A;ADAM3A	Body;Body;Body	chr8:39380225-39380533	Island
hypo522	N_insufficient_4	1.4E-08	0.58	0.86	-0.28	9	94178868	cg14193653	NFIL3	5'UTR		
hypo523	N_insufficient_4	6.0E-03	0.51	0.81	-0.30	10	4822998	cg23445945				
hypo524	N_insufficient_4	8.0E-10	0.52	0.83	-0.31	10	89116779	cg18516898	FAM22D	TSS1500	chr10:89114863-89115182	S_Shore
hypo525	N_insufficient_4	3.8E-06	0.42	0.70	-0.28	10	105215053	cg09083688	CALHM1	Body	chr10:105215052-105215460	Island
hypo526	N_insufficient_4	4.3E-04	0.87	0.97	-0.10	11	72465959	cg12598767	STARD10	Body	chr11:72465884-72466209	Island
hypo527	N_insufficient_4	7.4E-02	0.89	0.97	-0.08	11	72466028	cg03697509	STARD10	Body	chr11:72465884-72466209	Island
hypo528	N_insufficient_4	2.3E-06	0.45	0.77	-0.32	12	68720057	cg24599943	MDM1;MDM1	Body;3'UTR		
hypo529	N_insufficient_4	1.8E-08	0.60	0.90	-0.31	12	95048009	cg24950506			chr12:95043551-95044312	S_Shelf
hypo530	N_insufficient_4	1.8E-08	0.66	0.89	-0.22	13	100776866	cg11183144	PCCA;PCCA	Body;Body		
hypo531	N_insufficient_4	3.7E-02	0.93	0.97	-0.04	13	100803190	cg14630369	PCCA;PCCA	Body;Body		
hypo532	N_insufficient_4	2.1E-06	0.72	0.91	-0.19	13	100927476	cg26574777	PCCA;PCCA	Body;Body		
hypo533	N_insufficient_4	8.7E-02	0.53	0.76	-0.23	13	100944053	cg11312087	PCCA;PCCA	Body;Body		
hypo534	N_insufficient_4	8.7E-09	0.76	0.93	-0.17	13	101010186	cg23228451	PCCA;PCCA	Body;Body		
hypo535	N_insufficient_4	5.3E-04	0.77	0.87	-0.10	13	101166039	cg04154116	PCCA;PCCA	Body;Body		
hypo536	N_insufficient_4	1.6E-08	0.78	0.92	-0.15	13	101171765	cg15559940	PCCA;PCCA	Body;Body		
hypo537	N_insufficient_4	2.8E-04	0.71	0.88	-0.17	13	101174096	cg08985968	PCCA;PCCA	Body;Body		
hypo538	N_insufficient_4	3.7E-02	0.86	0.94	-0.08	13	101175894	cg05327509	PCCA;PCCA	Body;Body		
hypo539	N_insufficient_4	8.1E-03	0.85	0.93	-0.08	13	101179286	cg10894626	PCCA;PCCA	Body;Body		
hypo540	N_insufficient_4	1.3E-07	0.52	0.83	-0.32	14	65104174	cg13142949				
hypo541	N_insufficient_4	3.2E-02	0.54	0.81	-0.26	14	67828531	cg11076181	EIF2S1	5'UTR	chr14:67826139-67827526	S_Shore
hypo542	N_insufficient_4	1.8E-06	0.20	0.69	-0.49	15	30861172	cg23804921			chr15:30864782-30865586	N_Shelf
hypo543	N_insufficient_4	7.4E-02	0.08	0.35	-0.27	15	79383852	cg25673084	RASGRF1;RASGRF1	TSS1500;TSS1500	chr15:79381804-79384209	Island
hypo544	N_insufficient_4	2.1E-02	0.08	0.40	-0.31	15	79383858	cg11806630	RASGRF1;RASGRF1	TSS1500;TSS1500	chr15:79381804-79384209	Island
hypo545	N_insufficient_4	3.4E-04	0.08	0.40	-0.32	15	79383980	cg13571707	RASGRF1;RASGRF1	TSS1500;TSS1500	chr15:79381804-79384209	Island
hypo546	N_insufficient_4	4.1E-04	0.42	0.70	-0.27	16	48468609	cg03977385				
hypo547	N_insufficient_4	4.2E-11	0.57	0.89	-0.32	17	53470397	cg24943736	MMD	3'UTR		
hypo548	N_insufficient_4	9.3E-15	0.47	0.88	-0.41	17	80006003	cg27206903	RFNG	3'UTR	chr17:80008514-80010567	N_Shelf
hypo549	N_insufficient_4	1.9E-05	0.49	0.80	-0.30	19	49115456	cg10736770	FAM83E	Body	chr19:49116245-49116641	N_Shore
hypo550	N_insufficient_4	2.8E-08	0.49	0.81	-0.31	19	58372914	cg11949533	ZNF587	3'UTR		
hypo551	N_insufficient_5	9.3E-03	0.57	0.79	-0.22	2	175189039	cg00130528			chr2:175190866-175192123	N_Shore
hypo552	N_insufficient_5	6.1E-03	0.09	0.50	-0.40	3	11680536	cg22079057	VGLL4;VGLL4	Body;Body	chr3:11684192-11684930	N_Shelf
hypo553	N_insufficient_5	2.2E-06	0.37	0.74	-0.36	3	194119885	cg13185177	GP5	5'UTR	chr3:194117601-194118988	S_Shore
hypo554	N_insufficient_5	6.3E-03	0.41	0.73	-0.32	4	8277560	cg19303305	HTRA3	Body		
hypo555	N_insufficient_5	8.7E-04	0.20	0.45	-0.25	5	180486604	cg22418829	BTNL9	Body	chr5:180486154-180486892	Island
hypo556	N_insufficient_5	8.8E-02	0.34	0.59	-0.25	5	180486679	cg16049690	BTNL9	Body	chr5:180486154-180486892	Island
hypo557	N_insufficient_5	3.7E-05	0.71	0.89	-0.19	6	21078941	cg19130313	CDKAL1	Body		
hypo558	N_insufficient_5	6.8E-03	0.47	0.76	-0.29	6	97325863	cg27617159				
hypo559	N_insufficient_5	1.9E-06	0.10	0.86	-0.75	6	154821545	cg07895329	CNKSR3	Body		
hypo560	N_insufficient_5	5.1E-05	0.76	0.93	-0.17	7	1970648	cg17593934	MAD1L1;MAD1L1;MAD1L1	Body;Body;Body	chr7:1970627-1970924	Island
hypo561	N_insufficient_5	1.4E-08	0.54	0.91	-0.38	8	71182802	cg14656774	NCOA2	5'UTR		
hypo562	N_insufficient_5	1.2E-05	0.15	0.85	-0.70	9	37780478	cg13441107	EXOSC3;EXOSC3	3'UTR;3'UTR		
hypo563	N_insufficient_5	1.6E-13	0.57	0.93	-0.36	11	2222912	cg07146321				
hypo564	N_insufficient_5	3.9E-04	0.08	0.83	-0.75	11	17825098	cg08849813	SERGEF	Body		
hypo565	N_insufficient_5	1.1E-04	0.59	0.87	-0.28	11	70305696	cg20454213			chr11:70309696-70310121	N_Shelf
hypo566	N_insufficient_5	8.0E-11	0.50	0.86	-0.36	16	67965867	cg04413148	CTRL	TSS200	chr16:67969290-67970418	N_Shelf
hypo567	N_insufficient_5	1.5E-17	0.13	0.83	-0.71	16	69385761	cg08627125	TMED6	TSS200		
hypo568	N_insufficient_5	7.5E-08	0.47	0.80	-0.33	17	43220288	cg19172143	ACBD4;ACBD4;ACBD4;ACBD4;A	Body;Body;Body;3'UTR;Body	chr17:43221927-43222388	N_Shore
hypo569	N_insufficient_5	2.8E-08	0.58	0.88	-0.30	17	48251798	cg10296030	SGCA;SGCA	Body;Body		
hypo570	N_insufficient_5	3.9E-16	0.67	0.96	-0.28	19	869024	cg21306321	MED16	Body	chr19:870774-871318	N_Shore
hypo571	N_insufficient_5	1.4E-13	0.58	0.92	-0.34	19	44040457	cg27337277			chr19:44039232-44039756	S_Shore
hypo572	N_insufficient_5	2.9E-07	0.48	0.81	-0.33	19	48641011	cg15550049	LIG1	Body	chr19:48640718-48641014	Island
hypo573	N_insufficient_5	2.7E-02	0.09	0.38	-0.30	22	50164437	cg14884760			chr22:50164864-50165513	N_Shore
hypo574	N_adequate_1	2.6E-05	0.59	0.86	-0.27	1	3810632	cg09757127	C1orf174	Body	chr1:3808490-3809236	S_Shore
hypo575	N_adequate_1	1.6E-03	0.62	0.86	-0.24	2	16816994	cg10288578	FAM49A	5'UTR		
hypo576	N_adequate_1	8.3E-03	0.51	0.77	-0.25	4	6284031	cg16417416	WFS1;WFS1	Body;Body		
hypo577	N_adequate_1	1.8E-03	0.17	0.40	-0.23	4	187065417	cg10032668	FAM149A	TSS1500	chr4:187066155-187066460	N_Shore
hypo578	N_adequate_1	3.4E-03	0.17	0.39	-0.21	4	187065516	cg05911003	FAM149A	TSS1500	chr4:187066155-187066460	N_Shore
hypo579	N_adequate_1	3.3E-03	0.18	0.43	-0.25	4	187065713	cg04081326	FAM149A	TSS1500	chr4:187066155-187066460	N_Shore
hypo580	N_adequate_1	2.7E-07	0.23	0.61	-0.38	4	187065831	cg13208832	FAM149A	TSS200	chr4:187066155-187066460	N_Shore
hypo581	N_adequate_1	1.8E-07	0.24	0.59	-0.34	4	187065846	cg16289355	FAM149A	TSS200	chr4:187066155-187066460	N_Shore
hypo582	N_adequate_1	4.9E-05	0.26	0.59	-0.33	4	187065940	cg08055910	FAM149A	TSS200	chr4:187066155-187066460	N_Shore
hypo583	N_adequate_1	5.7E-04	0.21	0.57	-0.36	4	187065944	cg13136036	FAM149A	TSS200	chr4:187066155-187066460	N_Shore
hypo584	N_adequate_1	1.7E-07	0.22	0.54	-0.33	4	187065950	cg19873491	FAM149A	TSS200	chr4:187066155-187066460	N_Shore
hypo585	N_adequate_1	1.2E-07	0.27	0.59	-0.32	4	187065955	cg08644841	FAM149A	TSS200	chr4:187066155-187066460	N_Shore
hypo586	N_adequate_1	1.4E-05	0.70	0.91	-0.21	5	10226648	cg09898988	FAM173B	3'UTR		
hypo587	N_adequate_1	1.9E-10	0.50	0.87	-0.37	6	3267990	cg21819211	PSMG4;PSMG4	3'UTR;3'UTR		
hypo588	N_adequate_1	6.2E-04	0.63	0.85	-0.23	6	70507787	cg07062388	LMBRD1	TSS1500	chr6:70506292-70507024	S_Shore
hypo589	N_adequate_1	2.8E-04	0.81	0.91	-0.10	8	18387969	cg24613644	PSD3;PSD3	3'UTR;3'UTR		
hypo590	N_adequate_1	4.4E-05	0.53	0.79	-0.26	8	126438567	cg18989252			chr8:126441471-126443552	N_Shelf
hypo591	N_adequate_1	9.0E-02	0.39	0.67	-0.27	10	75336540	cg15134506	USP54	TSS1500		
hypo592	N_adequate_1	3.2E-09	0.63	0.91	-0.28	10	103813206	cg04945651	C10orf76	5'UTR	chr10:103815502-103816105	N_Shelf
hypo593	N_adequate_1	8.9E-02	0.74	0.88	-0.14	12	22688743	cg21005240	KIAA0528	Body		
hypo594	N_adequate_1	2.9E-07	0.56	0.82	-0.26	12	72246108	cg10833114	MRS2P2;TBC1D15;TBC1D15;TB(	TSS1500;Body;Body;5'UTR;Body		
hypo595	N_adequate_1	2.4E-07	0.57	0.81	-0.24	17	55540412	cg13790810	MSI2;MSI2	Body;Body		
hypo596	N_adequate_1	4.5E-03	0.56	0.80	-0.24	17	78834710	cg05249744	RPTOR;RPTOR	Body;Body	chr17:78831505-78831864	S_Shelf
hypo597	N_adequate_1	5.3E-06	0.59	0.85	-0.26	19	49204782	cg06594736	FUT2;FUT2	5'UTR;5'UTR	chr19:49206443-49206818	N_Shore
hypo598	N_adequate_2	6.5E-04	0.67	0.86	-0.19	1	180069210	cg24865482	CEP350	Body		
hypo599	N_adequate_2	9.8E-08	0.60	0.89	-0.29	1	211666429	cg06753273	RD3;RD3	TSS200;TSS200		
hypo600	N_adequate_2	4.0E-02	0.44	0.61	-0.18	2	44313980	cg01101677			chr2:44314288-44314564	N_Shore
hypo601	N_adequate_2	3.4E-02	0.72	0.92	-0.20	2	179641543	cg12541127	TTN;TTN;TTN;TTN;TTN	Body;Body;Body;Body;Body		
hypo602	N_adequate_2	6.6E-07	0.65	0.90	-0.25	4	1894156	cg09913413	WHSC1;WHSC1;WHSC1;WHSC1	TSS1500;TSS1500;TSS1500;TSS1500;5'UTR		
hypo603	N_adequate_2	8.9E-03	0.61	0.79	-0.17	6	33173218	cg02647520	HSD17B8	Body	chr6:33172289-33172823	S_Shore
hypo604	N_adequate_2	1.8E-02	0.81	0.94	-0.13	6	68596920	cg06060218				
hypo605	N_adequate_2	3.1E-05	0.71	0.91	-0.20	7	92079392	cg12912919	GATAD1	Body	chr7:92076699-92077395	S_Shore
hypo606	N_adequate_2	7.9E-02	0.57	0.80	-0.23	9	140568043	cg13791668	EHMT1;EHMT1	Body;Body		
hypo607	N_adequate_2	6.8E-11	0.68	0.94	-0.27	10	134359751	cg23736728	INPP5A	Body	chr10:134359533-134359780	Island
hypo608	N_adequate_2	1.4E-03	0.47	0.70	-0.23	10	134541472	cg20210689	INPP5A	Body	chr10:134539817-134540026	S_Shore
hypo609	N_adequate_2	1.0E-04	0.44	0.60	-0.17	11	2721866	cg05740879	KCNQ1OT1;KCNQ1;KCNQ1	TSS1500;Body;Body	chr11:2720410-2722087	Island
hypo610	N_adequate_2	4.9E-06	0.86	0.96	-0.11	11	63886204	cg17676132	FLRT1;MACROD1	3'UTR;Body	chr11:63887355-63887600	N_Shore
hypo611	N_adequate_2	1.5E-04	0.66	0.87	-0.21	15	36843680	cg22231259				
hypo612	N_adequate_2	3.5E-03	0.48	0.72	-0.24	15	63353166	cg13107144	TPM1;TPM1;TPM1;TPM1;TPM1;T	Body;Body;Body;Body;Body;Body;Body		
hypo613	N_adequate_2	1.9E-02	0.61	0.84	-0.24	15	102030434	cg03414001	PCSK6;PCSK6;PCSK6;PCSK6;P(	TSS1500;TSS1500;TSS1500;TSS	chr15:102028860-102030608	Island
hypo614	N_adequate_2	9.2E-05	0.76	0.91	-0.15	16	85543610	cg03350154				
hypo615	N_adequate_2	2.0E-02	0.70	0.87	-0.17	16	85543698	cg02431851				
hypo616	N_adequate_2	1.4E-06	0.86	0.98	-0.12	17	952903	cg10958086	ABR;ABR;ABR	Body;Body;Body	chr17:951746-952265	S_Shore
hypo617	N_adequate_2	2.6E-04	0.80	0.93	-0.13	17	59356720	cg08002981	BCAS3;BCAS3	Body;Body		
hypo618	N_adequate_2	4.6E-04	0.83	0.93	-0.10	18	77728011	cg00842670	HSBP1L1	Body	chr18:77724352-77724836	S_Shelf
hypo619	N_adequate_2	6.4E-03	0.93	0.97	-0.05	19	7532157	cg00734895	ARHGEF18;ARHGEF18	Body;Body	chr19:7531810-7532669	Island
hypo620	N_adequate_2	2.4E-03	0.81	0.91	-0.11	19	7532668	cg09436393	ARHGEF18;ARHGEF18	Body;Body	chr19:7531810-7532669	Island
hypo621	N_adequate_2	3.6E-09	0.83	0.98	-0.15	19	7533810	cg00013993	ARHGEF18;ARHGEF18	Body;Body	chr19:7531810-7532669	S_Shore
hypo622	N_adequate_2	3.0E-07	0.91	0.98	-0.07	19	7535526	cg16618417	ARHGEF18;ARHGEF18	3'UTR;3'UTR	chr19:7531810-7532669	S_Shelf
hypo623	N_adequate_2	5.2E-02	0.70	0.92	-0.22	19	45150513	cg07455685	PVR;PVR;PVR;PVR	Body;Body;Body;Body	chr19:45150371-45150834	Island
hypo624	N_adequate_2	7.1E-03	0.58	0.68	-0.11	19	45152536	cg24098859	PVR;PVR;PVR;PVR	Body;Body;Body;Body	chr19:45150371-45150834	S_Shore
hypo625	N_adequate_2	6.8E-04	0.59	0.82	-0.23	22	50174028	cg06865642	BRD1;LOC90834	Body;TSS200	chr22:50177720-50178221	N_Shelf
hypo626	N_adequate_3	3.1E-04	0.82	0.92	-0.10	1	198828005	cg22821358	MIR181B1	Body		
hypo627	N_adequate_3	3.9E-08	0.67	0.92	-0.25	3	9025986	cg08558733	SRGAP3;SRGAP3	3'UTR;3'UTR	chr3:9027156-9027611	N_Shore
hypo628	N_adequate_3	1.7E-07	0.64	0.90	-0.27	4	745235	cg01017228	PCGF3	Body	chr4:738424-741517	S_Shelf
hypo629	N_adequate_3	5.6E-02	0.83	0.93	-0.10	4	187627773	cg11512859	FAT1	Body		
hypo630	N_adequate_3	6.5E-07	0.68	0.93	-0.25	5	36243704	cg10946263	C5orf33	TSS1500	chr5:36241003-36242664	S_Shore
hypo631	N_adequate_3	4.6E-02	0.64	0.84	-0.20	5	69371421	cg22024931	SMN1;SMN2;SMN2;SMN2;SMN1;	Body;Body;Body;Body;Body;Body		
hypo632	N_adequate_3	2.2E-07	0.77	0.96	-0.19	6	16347197	cg19185641	ATXN1;ATXN1	5'UTR;5'UTR		
hypo633	N_adequate_3	3.3E-04	0.78	0.90	-0.12	6	16375082	cg27395391	ATXN1;ATXN1	5'UTR;5'UTR		
hypo634	N_adequate_3	2.1E-02	0.68	0.89	-0.21	6	106777731	cg25511725			chr6:106773187-106773803	S_Shelf
hypo635	N_adequate_3	6.1E-03	0.27	0.50	-0.23	7	24323764	cg24885417	NPY	TSS200	chr7:24323558-24325080	Island
hypo636	N_adequate_3	9.8E-04	0.25	0.62	-0.38	7	24323799	cg16964348	NPY	TSS200	chr7:24323558-24325080	Island
hypo637	N_adequate_3	6.2E-08	0.27	0.57	-0.30	7	24323939	cg21097881	NPY	5'UTR	chr7:24323558-24325080	Island
hypo638	N_adequate_3	8.6E-02	0.35	0.63	-0.28	7	158362729	cg19423622	PTPRN2;PTPRN2;PTPRN2	Body;Body;Body	chr7:158361909-158362970	Island
hypo639	N_adequate_3	2.0E-02	0.63	0.82	-0.19	8	41420945	cg17298818			chr8:41424341-41425300	N_Shelf
hypo640	N_adequate_3	6.0E-07	0.64	0.90	-0.26	10	101180657	cg05068943	GOT1	Body		

hypo641	N_adequate_3	6.1E-03	0.86	0.98	-0.12	10	131567801	cg13742952				
hypo642	N_adequate_3	4.5E-10	0.66	0.93	-0.27	12	112622311	cg14415857	C12orf51	Body	chr12:112622182-112622815	Island
hypo643	N_adequate_3	1.2E-07	0.58	0.86	-0.28	16	672991	cg02311180	RAB40C	Body	chr16:672265-672507	S_Shore
hypo644	N_adequate_3	2.0E-03	0.85	0.92	-0.08	17	68128832	cg10858415	KCNJ16;KCNJ16;KCNJ16	Body;Body;Body		
hypo645	N_adequate_3	1.2E-05	0.53	0.79	-0.26	17	80857351	cg04774110	TBCD	Body	chr17:80856874-80857352	Island
hypo646	N_adequate_3	1.4E-02	0.84	0.93	-0.09	18	72799885	cg24601355			chr18:72799643-72799947	Island
hypo647	N_adequate_3	4.2E-02	0.53	0.68	-0.15	20	57582787	cg17398227	CTSZ	TSS1500	chr20:57581902-57582595	S_Shore
hypo648	N_adequate_3	7.1E-02	0.50	0.76	-0.26	22	50620975	cg03007524			chr22:50623164-50625226	N_Shelf
hypo649	N_adequate_4	6.9E-13	0.66	0.95	-0.30	2	61351337	cg16887385	KIAA1841;KIAA1841	Body;3'UTR		
hypo650	N_adequate_4	1.3E-04	0.56	0.85	-0.29	3	149292366	cg08498836	WWTR1;WWTR1;WWTR1	Body;Body;Body		
hypo651	N_adequate_4	2.4E-07	0.39	0.85	-0.46	3	194786218	cg10348826			chr3:194785977-194786549	Island
hypo652	N_adequate_4	7.2E-06	0.53	0.85	-0.32	4	57186067	cg12159577	KIAA1211	Body	chr4:57180957-57182147	S_Shelf
hypo653	N_adequate_4	4.3E-11	0.60	0.93	-0.34	5	124537511	cg04636251				
hypo654	N_adequate_4	1.9E-03	0.91	0.98	-0.07	5	178209759	cg06651605				
hypo655	N_adequate_4	8.5E-09	0.42	0.85	-0.43	6	31760762	cg02980249	VARS	Body	chr6:31763240-31763905	N_Shelf
hypo656	N_adequate_4	4.7E-14	0.49	0.86	-0.37	6	34194644	cg26395374			chr6:34191907-34192179	S_Shelf
hypo657	N_adequate_4	1.4E-05	0.72	0.93	-0.21	6	107075197	cg01625761	RTN4IP1	Body		
hypo658	N_adequate_4	6.7E-04	0.07	0.38	-0.31	6	150070727	cg08634157	PCMT1	TSS200	chr6:150070513-150071285	Island
hypo659	N_adequate_4	6.1E-02	0.89	0.97	-0.08	6	168122607	cg14368954			chr6:168120634-168120892	S_Shore
hypo660	N_adequate_4	1.9E-11	0.44	0.81	-0.36	7	127756893	cg21127729				
hypo661	N_adequate_4	5.5E-07	0.59	0.89	-0.30	8	38460153	cg25160034	RNF5P1	TSS1500		
hypo662	N_adequate_4	5.1E-03	0.55	0.83	-0.28	10	13714674	cg08557179	FRMD4A	Body		
hypo663	N_adequate_4	1.1E-07	0.49	0.83	-0.34	10	126331425	cg20151107	FAM53B	Body		
hypo664	N_adequate_4	5.0E-07	0.54	0.85	-0.31	13	114127083	cg00781839	DCUN1D2	Body		
hypo665	N_adequate_4	1.4E-06	0.61	0.89	-0.28	14	55037188	cg01030747	SAMD4A;SAMD4A	Body;Body	chr14:55031826-55035030	S_Shelf
hypo666	N_adequate_4	6.1E-13	0.25	0.85	-0.60	14	90991781	cg06759417			chr14:90991767-90991982	Island
hypo667	N_adequate_4	3.7E-07	0.42	0.76	-0.33	14	103393970	cg23897302	AMN	Body	chr14:103394397-103397070	N_Shore
hypo668	N_adequate_4	1.8E-04	0.45	0.74	-0.29	15	23005487	cg17122577	NIPA2;NIPA2;NIPA2;NIPA2	3'UTR;3'UTR;3'UTR;3'UTR		
hypo669	N_adequate_4	2.2E-02	0.42	0.64	-0.22	16	81586800	cg00864293	CMIP;CMIP	Body;Body		
hypo670	N_adequate_4	6.4E-07	0.51	0.81	-0.31	17	65471461	cg15756507	PITPNC1;PITPNC1	Body;Body		
hypo671	N_adequate_4	4.6E-08	0.50	0.84	-0.34	19	54232239	cg26768546	MIR518E	TSS1500		
hypo672	N_adequate_4	6.7E-03	0.57	0.88	-0.31	22	24284359	cg17293426				
hypo673	N_adequate_5	6.2E-04	0.53	0.79	-0.26	1	1251322	cg08939275	CPSF3L	Body	chr1:1254322-1254548	N_Shelf
hypo674	N_adequate_5	1.9E-09	0.71	0.94	-0.23	1	167738686	cg04348561	MPZL1;MPZL1;MPZL1	Body;Body;Body		
hypo675	N_adequate_5	5.7E-10	0.68	0.90	-0.23	3	53843939	cg01980361	CACNA1D;CACNA1D;CACNA1D	Body;Body;Body		
hypo676	N_adequate_5	3.3E-05	0.73	0.93	-0.20	3	187896222	cg21909391	LPP;FLJ42393	5'UTR;TSS200		
hypo677	N_adequate_5	1.7E-07	0.79	0.89	-0.11	7	5413639	cg21364020	TNRC18	Body	chr7:5413745-5414210	N_Shore
hypo678	N_adequate_5	4.1E-02	0.61	0.79	-0.17	9	71627524	cg04563766	PRKACG;PRKACG	3'UTR;1stExon	chr9:71628407-71628907	N_Shore
hypo679	N_adequate_5	1.8E-03	0.66	0.83	-0.17	9	140681131	cg13632520	EHMT1	Body	chr9:140683120-140683774	N_Shore
hypo680	N_adequate_5	3.5E-04	0.62	0.83	-0.20	10	43882723	cg03699765	HNRNPF;HNRNPF;HNRNPF;HNI	Body;Body;Body;Body;Body;Body		
hypo681	N_adequate_5	1.3E-04	0.69	0.89	-0.19	10	127867337	cg25588387	ADAM12;ADAM12	Body;Body		
hypo682	N_adequate_5	5.0E-06	0.62	0.84	-0.22	11	1000632	cg06272648	AP2A2	Body		
hypo683	N_adequate_5	1.5E-04	0.66	0.86	-0.20	11	78954267	cg14098737	ODZ4	5'UTR		
hypo684	N_adequate_5	9.3E-06	0.66	0.86	-0.20	11	95900374	cg04200485	MAML2	Body		
hypo685	N_adequate_5	4.1E-02	0.82	0.93	-0.11	12	57588180	cg03668470	LRP1;MIR1228	Body;TSS200		
hypo686	N_adequate_5	1.7E-03	0.73	0.90	-0.17	12	90106972	cg00363830			chr12:90101436-90104065	S_Shelf
hypo687	N_adequate_5	8.4E-02	0.85	0.93	-0.08	16	84809541	cg08991234	USP10	Body		
hypo688	N_adequate_5	1.9E-02	0.88	0.96	-0.08	16	87448114	cg26936171	ZCCHC14	Body	chr16:87447699-87448131	Island
hypo689	N_adequate_5	6.0E-10	0.70	0.92	-0.22	19	2255496	cg15164761	JSRP1	TSS200	chr19:2250560-2253959	S_Shore
hypo690	N_adequate_5	2.7E-04	0.58	0.81	-0.23	19	35597415	cg06642503	LOC100128675;LOC100128675	TSS1500;TSS1500		
hypo691	N_adequate_6	5.7E-03	0.78	0.95	-0.17	1	2572909	cg04995493			chr1:2574030-2574864	N_Shore
hypo692	N_adequate_6	5.2E-03	0.67	0.92	-0.25	1	5918400	cg13384923				
hypo693	N_adequate_6	4.6E-03	0.51	0.70	-0.19	1	9062931	cg24295324				
hypo694	N_adequate_6	3.4E-02	0.49	0.70	-0.21	1	11847167	cg22877851	MTHFR	3'UTR		
hypo695	N_adequate_6	1.5E-04	0.87	0.97	-0.09	1	16810776	cg12462858	CROCCL2	Body	chr1:16810605-16810957	Island
hypo696	N_adequate_6	1.0E-05	0.68	0.90	-0.22	1	183897368	cg13951632	RGL1	3'UTR		
hypo697	N_adequate_6	4.3E-07	0.64	0.90	-0.25	1	235265522	cg12444298			chr1:235267554-235267829	N_Shelf
hypo698	N_adequate_6	2.8E-03	0.55	0.77	-0.22	2	3464991	cg25113123	TTC15	Body	chr2:3461654-3461863	S_Shelf
hypo699	N_adequate_6	1.5E-08	0.71	0.91	-0.21	2	9573328	cg16710448	CPSF3	Body		
hypo700	N_adequate_6	2.6E-02	0.60	0.79	-0.19	2	228551538	cg04091914	SLC19A3	3'UTR		
hypo701	N_adequate_6	1.3E-09	0.61	0.89	-0.28	2	240090977	cg06583077	HDAC4	Body		
hypo702	N_adequate_6	1.0E-03	0.19	0.56	-0.37	3	36422093	cg24202123	STAC	TSS200	chr3:36421702-36422210	Island
hypo703	N_adequate_6	7.8E-09	0.66	0.91	-0.25	3	37258149	cg21328643				
hypo704	N_adequate_6	1.2E-10	0.64	0.89	-0.25	4	6611128	cg25892537	MAN2B2	Body		
hypo705	N_adequate_6	4.0E-02	0.44	0.57	-0.14	4	89619053	cg01026744	NAP1L5;HERC3	TSS200;Body		
hypo706	N_adequate_6	4.7E-02	0.75	0.91	-0.16	6	6952892	cg14537605				
hypo707	N_adequate_6	9.3E-07	0.69	0.91	-0.21	6	49928115	cg19592003	DEFB114	Body		
hypo708	N_adequate_6	1.9E-10	0.65	0.90	-0.26	7	2120268	cg12495649	MAD1L1;MAD1L1;MAD1L1	Body;Body;Body	chr7:2116367-2116781	S_Shelf
hypo709	N_adequate_6	8.0E-05	0.60	0.84	-0.24	7	4265272	cg14017689	SDK1;SDK1	Body;Body		
hypo710	N_adequate_6	1.0E-07	0.64	0.91	-0.27	7	16127844	cg21888978	ISPD;ISPD	3'UTR;3'UTR		
hypo711	N_adequate_6	1.8E-03	0.67	0.87	-0.20	7	134363869	cg23891049	BPGM;BPGM	3'UTR;3'UTR		
hypo712	N_adequate_6	8.6E-10	0.64	0.89	-0.25	8	144917532	cg02658043	NRBP2	3'UTR	chr8:144917878-144918318	N_Shore
hypo713	N_adequate_6	3.1E-07	0.65	0.92	-0.27	9	37340211	cg13595739	ZCCHC7	Body		
hypo714	N_adequate_6	1.2E-05	0.69	0.91	-0.22	9	37420996	cg01650612			chr9:37422568-37422915	N_Shore
hypo715	N_adequate_6	6.1E-04	0.67	0.88	-0.21	9	110403556	cg14254422			chr9:110399674-110400566	S_Shelf
hypo716	N_adequate_6	1.9E-02	0.50	0.74	-0.24	9	140077638	cg09307883	ANAPC2	Body	chr9:140079329-140079764	N_Shore
hypo717	N_adequate_6	9.8E-07	0.55	0.80	-0.25	11	285037	cg22280068	NLRP6	Body	chr11:288847-289239	N_Shelf
hypo718	N_adequate_6	6.4E-06	0.64	0.88	-0.24	14	93395464	cg18397726	CHGA	Body		
hypo719	N_adequate_6	8.9E-06	0.60	0.83	-0.24	15	96846163	cg06614221				
hypo720	N_adequate_6	1.7E-04	0.51	0.71	-0.20	16	2986893	cg01283685	FLYWCH1;FLYWCH1	Body;Body		
hypo721	N_adequate_6	1.7E-04	0.34	0.82	-0.48	16	20750043	cg26846609	THUMPD1	Body	chr16:20752646-20753280	N_Shelf
hypo722	N_adequate_6	4.9E-02	0.52	0.77	-0.26	16	85555434	cg00782188			chr16:85551382-85551709	S_Shelf
hypo723	N_adequate_6	1.5E-09	0.63	0.91	-0.29	17	80436296	cg21997403	NARF;NARF;NARF;NARF	Body;Body;Body;Body		
hypo724	N_adequate_6	9.6E-04	0.54	0.78	-0.24	19	17834037	cg08692328	MAP1S	Body	chr19:17836755-17838816	N_Shelf
hypo725	N_adequate_6	6.6E-06	0.64	0.85	-0.21	19	41319790	cg01282508			chr19:41317792-41318151	S_Shore
hypo726	N_adequate_6	1.2E-02	0.69	0.85	-0.16	20	60858364	cg14290705	OSBPL2;OSBPL2	Body;Body	chr20:60860732-60860999	N_Shelf
hypo727	N_adequate_6	5.3E-05	0.66	0.85	-0.18	20	62705807	cg14128040	RGS19;RGS19	Body;Body	chr20:62705080-62705943	Island
hypo728	N_adequate_6	6.2E-03	0.73	0.90	-0.17	22	42423479	cg10766792	WBP2NL	3'UTR		
hypo729	N_adequate_7	3.0E-02	0.33	0.87	-0.54	2	11286089	cg09167828	C2orf50	3'UTR		
hypo730	N_adequate_7	3.8E-08	0.68	0.89	-0.21	7	139872929	cg15122117	JHDM1D	Body	chr7:139875411-139877493	N_Shelf
hypo731	N_adequate_7	1.2E-06	0.62	0.88	-0.26	8	29589696	cg21297996	C8orf75	Body		
hypo732	N_adequate_7	4.6E-03	0.68	0.87	-0.18	9	123364569	cg13459910	MEGF9	3'UTR		
hypo733	N_adequate_7	2.0E-10	0.64	0.92	-0.28	9	133572862	cg13771918	EXOSC2	Body	chr9:133569082-133569451	S_Shelf
hypo734	N_adequate_7	6.5E-05	0.60	0.90	-0.30	11	60858720	cg20418308				
hypo735	N_adequate_7	1.7E-07	0.66	0.90	-0.24	11	76731589	cg27121309	ACER3	3'UTR		
hypo736	N_adequate_7	1.8E-02	0.66	0.90	-0.24	14	69381541	cg04252710	ACTN1;ACTN1;ACTN1	Body;Body;Body		
hypo737	N_adequate_7	1.6E-05	0.64	0.84	-0.20	15	50792856	cg22422264	USP50;USP8;USP8;USP8	3'UTR;3'UTR;3'UTR;3'UTR		
hypo738	N_adequate_8	4.9E-09	0.65	0.89	-0.24	1	1888447	cg12665890	KIAA1751	Body	chr1:1886344-1886870	S_Shore
hypo739	N_adequate_8	1.9E-10	0.67	0.93	-0.25	1	2084391	cg24445388	PRKCZ;PRKCZ;PRKCZ	Body;Body;Body	chr1:2082314-2082529	S_Shore
hypo740	N_adequate_8	5.4E-02	0.61	0.86	-0.25	1	3401697	cg13901857				
hypo741	N_adequate_8	1.1E-11	0.66	0.92	-0.26	1	6653941	cg10830310	KLHL21	Body	chr1:6653397-6653674	S_Shore
hypo742	N_adequate_8	2.7E-09	0.60	0.84	-0.24	1	16060610	cg15135166	PLEKHM2	3'UTR	chr1:16058297-16058505	S_Shelf
hypo743	N_adequate_8	2.9E-06	0.65	0.89	-0.25	1	49244849	cg17076795	AGBL4	Body	chr1:49242371-49242810	S_Shelf
hypo744	N_adequate_8	8.3E-08	0.61	0.87	-0.26	2	8686087	cg00534022				
hypo745	N_adequate_8	1.2E-02	0.60	0.81	-0.20	3	121307887	cg25771052	ARGFX	3'UTR		
hypo746	N_adequate_8	1.9E-04	0.67	0.88	-0.22	4	148701131	cg24290320	ARHGAP10	Body		
hypo747	N_adequate_8	6.3E-02	0.85	0.91	-0.05	4	148993643	cg23650353	ARHGAP10	3'UTR		
hypo748	N_adequate_8	7.8E-04	0.59	0.81	-0.22	5	151041431	cg06571753	SPARC	3'UTR		
hypo749	N_adequate_8	5.3E-08	0.57	0.84	-0.27	6	43857911	cg01172073	LOC100132354	TSS1500		
hypo750	N_adequate_8	1.5E-04	0.66	0.86	-0.20	6	112032564	cg01557421	FYN;FYN;FYN	Body;Body;Body		
hypo751	N_adequate_8	4.8E-09	0.70	0.94	-0.24	8	6607096	cg21257675	AGPAT5	Body		
hypo752	N_adequate_8	2.3E-02	0.90	0.96	-0.07	8	144557035	cg00773700	ZC3H3	Body		
hypo753	N_adequate_8	1.0E-08	0.62	0.90	-0.28	9	92291210	cg13655615	LOC100129066	Body	chr9:92291268-92291524	N_Shore
hypo754	N_adequate_8	2.9E-07	0.64	0.88	-0.25	11	102668176	cg25320665	MMP1;MMP1	Body;5'UTR		
hypo755	N_adequate_8	1.7E-04	0.64	0.87	-0.23	14	92938068	cg21881330	SLC24A4;SLC24A4;SLC24A4	Body;Body;Body		
hypo756	N_adequate_8	8.2E-02	0.53	0.74	-0.21	15	91364242	cg04612952			chr15:91362445-91362658	S_Shore
hypo757	N_adequate_8	1.1E-07	0.46	0.79	-0.33	18	45690642	cg23824354			chr18:45687797-45688512	S_Shelf
hypo758	N_adequate_8	6.5E-09	0.58	0.82	-0.24	19	4101514	cg18236745	MAP2K2	Body	chr19:4101029-4101515	Island
hypo759	N_adequate_8	1.1E-06	0.61	0.87	-0.26	19	12371781	cg24307601			chr19:12371052-12371335	S_Shore
hypo760	N_adequate_8	3.2E-05	0.67	0.89	-0.22	19	20187745	cg12007683	ZNF90	TSS1500		
hypo761	N_adequate_9	1.4E-02	0.79	0.88	-0.09	1	230310556	cg11222630	GALNT2	Body		
hypo762	N_adequate_9	1.5E-02	0.78	0.86	-0.09	3	55005522	cg19631651	CACNA2D3	Body		
hypo763	N_adequate_9	1.0E-06	0.53	0.90	-0.37	3	71201264	cg13726313	FOXP1	Body		
hypo764	N_adequate_9	1.6E-13	0.56	0.92	-0.36	5	1325588	cg06550200	CLPTM1L	Body		
hypo765	N_adequate_9	1.3E-04	0.41	0.67	-0.26	5	142514108	cg19729949	ARHGAP26;ARHGAP26	Body;Body		
hypo766	N_adequate_9	2.1E-02	0.35	0.72	-0.37	6	105400993	cg22620090			chr6:105400877-105401149	Island
hypo767	N_adequate_9	1.4E-02	0.91	0.98	-0.07	6	170060988	cg19951766	WDR27	Body	chr6:170062622-170063112	N_Shore
hypo768	N_adequate_9	4.7E-03	0.16	0.63	-0.47	7	533963	cg04453501			chr7:533426-534109	Island
hypo769	N_adequate_9	7.1E-06	0.59	0.81	-0.22	7	2044787	cg07556911	MAD1L1;MAD1L1;MAD1L1	Body;Body;Body	chr7:2044583-2044788	Island

hypo770	N_adequate_9	1.4E-02	0.85	0.94	-0.09	8	47112230	cg11415389				
hypo771	N_adequate_9	3.9E-17	0.10	0.85	-0.75	9	95475787	cg14341177	BICD2;BICD2	Body;3'UTR	chr9:95477296-95477708	N_Shore
hypo772	N_adequate_9	1.9E-04	0.53	0.76	-0.23	10	35676874	cg17262810	CCNY;CCNY	Body;5'UTR		
hypo773	N_adequate_9	8.2E-15	0.53	0.90	-0.37	10	134585121	cg16109778	INPP5A	Body	chr10:134586170-134586471	N_Shore
hypo774	N_adequate_9	1.0E-08	0.51	0.85	-0.34	11	119993914	cg06654510	TRIM29	Body		
hypo775	N_adequate_9	9.1E-05	0.53	0.84	-0.31	12	89748932	cg05403689			chr12:89745168-89748144	S_Shore
hypo776	N_adequate_9	2.3E-03	0.80	0.92	-0.12	12	130623366	cg00019366			chr12:130623142-130623492	Island
hypo777	N_adequate_9	4.9E-03	0.80	0.96	-0.16	12	131496452	cg10443500	GPR133	Body		
hypo778	N_adequate_9	9.7E-06	0.58	0.84	-0.26	13	24408100	cg25909854	MIPEP	Body		
hypo779	N_adequate_9	7.5E-05	0.10	0.50	-0.41	15	93615629	cg21614339	RGMA;RGMA;RGMA;RGMA;RGI	Body;Body;Body;Body;Body;Body	chr15:93615622-93617185	Island
hypo780	N_adequate_9	8.1E-07	0.10	0.57	-0.48	15	93615677	cg23115069	RGMA;RGMA;RGMA;RGMA;RGI	Body;Body;Body;Body;Body;Body	chr15:93615622-93617185	Island
hypo781	N_adequate_9	1.5E-04	0.13	0.47	-0.34	15	93615718	cg16390448	RGMA;RGMA;RGMA;RGMA;RGI	Body;Body;Body;Body;Body;Body	chr15:93615622-93617185	Island
hypo782	N_adequate_9	1.6E-07	0.26	0.55	-0.28	15	93616072	cg19781376	RGMA;RGMA;RGMA;RGMA;RGI	Body;Body;Body;Body;Body;Body	chr15:93615622-93617185	Island
hypo783	N_adequate_9	2.0E-06	0.12	0.56	-0.44	15	93616588	cg20129994	RGMA;RGMA;RGMA;RGMA;RGI	TSS200;Body;5'UTR;5'UTR;5'UTF	chr15:93615622-93617185	Island
hypo784	N_adequate_9	9.9E-11	0.33	0.88	-0.55	17	19248009	cg24547137	MIR1180;B9D1	TSS200;Body	chr17:19247785-19248069	Island
hypo785	N_adequate_9	1.7E-04	0.64	0.86	-0.22	19	36746737	cg00236601			chr19:36746712-36746914	Island
hypo786	N_adequate_9	1.2E-08	0.57	0.91	-0.33	19	57702020	cg18055682	ZNF264	TSS1500	chr19:57702696-57703425	N_Shore
hypo787	N_adequate_9	2.7E-03	0.43	0.68	-0.25	20	37054900	cg13610455	LOC388796;SNORA71B;LOC388	Body;TSS1500;Body		
hypo788	N_adequate_9	8.4E-03	0.48	0.76	-0.28	21	44486205	cg09128751	CBS	Body	chr21:44486140-44486465	Island
hypo789	N_excessive_1	2.1E-05	0.77	0.91	-0.14	1	144489264	cg12410570	LOC728875	Body	chr1:144491618-144492007	N_Shelf
hypo790	N_excessive_1	5.0E-05	0.60	0.88	-0.29	1	147877188	cg24367548			chr1:147874548-147874846	S_Shelf
hypo791	N_excessive_1	5.2E-12	0.60	0.90	-0.30	1	203602040	cg13384512	ATP2B4;ATP2B4	5'UTR;5'UTR	chr1:203598471-203598853	S_Shelf
hypo792	N_excessive_1	1.5E-02	0.62	0.83	-0.21	2	87020937	cg25939861	CD8A	5'UTR	chr2:87015974-87018182	S_Shelf
hypo793	N_excessive_1	2.1E-02	0.50	0.81	-0.31	2	87090181	cg26135863	CD8B;CD8B;CD8B;CD8B;CD8B	TSS1500;TSS1500;TSS1500;TSS	chr2:87088816-87089037	S_Shore
hypo794	N_excessive_1	6.7E-16	0.62	0.96	-0.34	2	87169392	cg03176543	RGPD1;RGPD2	Body;Body	chr2:87169173-87170303	Island
hypo795	N_excessive_1	1.8E-02	0.83	0.92	-0.09	2	103379054	cg01243790	TMEM182	Body		
hypo796	N_excessive_1	8.7E-13	0.59	0.92	-0.33	2	152349105	cg14802971	NEB;NEB;NEB	Body;Body;Body		
hypo797	N_excessive_1	4.3E-06	0.44	0.80	-0.35	3	48265146	cg04523589	CAMP	1stExon		
hypo798	N_excessive_1	6.3E-05	0.47	0.78	-0.31	3	69171735	cg14457610	LMOD3;LMOD3	5'UTR;1stExon		
hypo799	N_excessive_1	3.4E-03	0.47	0.70	-0.24	3	122604848	cg22707878	LOC100129550	TSS1500		
hypo800	N_excessive_1	1.9E-10	0.46	0.79	-0.32	3	188623991	cg27225068				
hypo801	N_excessive_1	1.3E-03	0.79	0.97	-0.17	5	140053068	cg03136712	DND1	Body	chr5:140052059-140053381	Island
hypo802	N_excessive_1	4.8E-02	0.77	0.88	-0.11	5	156691755	cg03398461	CYFIP2;CYFIP2	TSS1500;TSS1500	chr5:156692812-156693749	N_Shore
hypo803	N_excessive_1	4.3E-05	0.37	0.61	-0.24	6	30899649	cg13561028	SFTA2	Body		
hypo804	N_excessive_1	5.1E-02	0.43	0.69	-0.26	6	31820754	cg07883262				
hypo805	N_excessive_1	5.2E-07	0.52	0.83	-0.31	7	45729156	cg14919164	ADCY1	Body		
hypo806	N_excessive_1	1.2E-02	0.26	0.55	-0.29	10	65224441	cg17775079	JMJD1C;LOC84989	Body;TSS1500	chr10:65224870-65225887	N_Shore
hypo807	N_excessive_1	1.9E-11	0.06	0.60	-0.54	10	65225196	cg11467732	JMJD1C;LOC84989	Body;Body	chr10:65224870-65225887	Island
hypo808	N_excessive_1	1.9E-13	0.09	0.54	-0.45	10	65225544	cg10235275	JMJD1C;JMJD1C;LOC84989	5'UTR;1stExon;Body	chr10:65224870-65225887	Island
hypo809	N_excessive_1	8.1E-15	0.09	0.59	-0.50	10	65225552	cg27430682	JMJD1C;JMJD1C;LOC84989	5'UTR;1stExon;Body	chr10:65224870-65225887	Island
hypo810	N_excessive_1	2.3E-03	0.08	0.37	-0.30	10	65225630	cg01667332	JMJD1C;JMJD1C;LOC84989	5'UTR;1stExon;Body	chr10:65224870-65225887	Island
hypo811	N_excessive_1	6.6E-06	0.11	0.50	-0.38	10	65225947	cg08222002	LOC84989;JMJD1C	Body;TSS1500	chr10:65224870-65225887	S_Shore
hypo812	N_excessive_1	1.4E-03	0.05	0.40	-0.35	10	65225999	cg19955118	LOC84989;JMJD1C	Body;TSS1500	chr10:65224870-65225887	S_Shore
hypo813	N_excessive_1	1.1E-10	0.52	0.84	-0.32	10	103341227	cg03173714	POLL	Body		
hypo814	N_excessive_1	1.2E-05	0.43	0.84	-0.41	10	130279707	cg24625984				
hypo815	N_excessive_1	2.7E-04	0.47	0.76	-0.29	10	134584660	cg02240936	INPP5A	Body	chr10:134586170-134586471	N_Shore
hypo816	N_excessive_1	1.1E-02	0.75	0.89	-0.14	11	32854320	cg17556588	PRRG4	Body	chr11:32851160-32851951	S_Shelf
hypo817	N_excessive_1	9.0E-13	0.63	0.94	-0.30	12	15125458	cg03253385	PDE6H	TSS1500		
hypo818	N_excessive_1	2.5E-04	0.39	0.64	-0.24	12	117440120	cg10065736	FBXW8;FBXW8	Body;Body		
hypo819	N_excessive_1	1.0E-03	0.34	0.74	-0.40	13	113422177	cg11702988	ATP11A;ATP11A	Body;Body	chr13:113420152-113420384	S_Shore
hypo820	N_excessive_1	6.7E-03	0.73	0.94	-0.22	13	114193150	cg21561701	TMCO3	Body	chr13:114195709-114196719	N_Shelf
hypo821	N_excessive_1	4.4E-02	0.68	0.95	-0.26	13	114193224	cg08699341	TMCO3	Body	chr13:114195709-114196719	N_Shelf
hypo822	N_excessive_1	7.9E-05	0.76	0.90	-0.14	13	114198455	cg26112996	TMCO3	Body	chr13:114198318-114198552	Island
hypo823	N_excessive_1	1.1E-04	0.44	0.69	-0.24	15	75490372	cg14832223			chr15:75493982-75495331	N_Shelf
hypo824	N_excessive_1	5.7E-04	0.18	0.64	-0.46	16	89323363	cg27382568			chr16:89323280-89323661	Island
hypo825	N_excessive_1	2.4E-02	0.09	0.55	-0.46	16	89323411	cg01197871			chr16:89323280-89323661	Island
hypo826	N_excessive_1	3.3E-13	0.52	0.91	-0.39	19	626119	cg17830140	POLRMT	Body	chr19:615691-623505	S_Shelf
hypo827	N_excessive_1	2.9E-03	0.16	0.50	-0.34	19	13617012	cg17509220	CACNA1A;CACNA1A	1stExon;1stExon	chr19:13616752-13617267	Island
hypo828	N_excessive_1	1.4E-08	0.07	0.58	-0.51	19	13617091	cg22491927	CACNA1A;CACNA1A;CACNA1A;	5'UTR;1stExon;1stExon;5'UTR	chr19:13616752-13617267	Island
hypo829	N_excessive_1	9.5E-15	0.44	0.84	-0.39	19	41860619	cg16883145	B9D2;TGFB1	Body;TSS1500	chr19:41858527-41860100	S_Shore
hypo830	N_excessive_1	1.0E-03	0.76	0.91	-0.15	19	52491114	cg15815730	ZNF350	TSS1500		
hypo831	N_excessive_1	3.5E-03	0.28	0.54	-0.26	22	40058706	cg01257975	CACNA1I;CACNA1I	Body;Body	chr22:40057941-40058844	Island
hypo832	N_excessive_2	1.2E-08	0.54	0.88	-0.34	2	220303766	cg26667692	SPEG	Body	chr2:220306855-220307096	N_Shelf
hypo833	N_excessive_2	3.6E-02	0.55	0.82	-0.26	4	89660237	cg07213482	FAM13A;FAM13A	Body;Body		
hypo834	N_excessive_2	3.0E-04	0.61	0.85	-0.25	4	175446087	cg06937164			chr4:175443406-175443837	S_Shelf
hypo835	N_excessive_2	3.3E-09	0.57	0.89	-0.33	5	109218877	cg00281129	LOC100289673	TSS200	chr5:109220986-109221247	N_Shelf
hypo836	N_excessive_2	9.7E-12	0.64	0.92	-0.28	5	137957283	cg06565615				
hypo837	N_excessive_2	2.0E-02	0.10	0.76	-0.66	6	14524587	cg15042082				
hypo838	N_excessive_2	2.8E-09	0.15	0.92	-0.77	6	131278559	cg11386709	EPB41L2;EPB41L2;EPB41L2	5'UTR;5'UTR;5'UTR		
hypo839	N_excessive_2	2.3E-02	0.51	0.84	-0.33	6	133084898	cg15621874	VNN2	TSS1500		
hypo840	N_excessive_2	3.4E-06	0.89	0.98	-0.09	7	1902804	cg11033688	MAD1L1;MAD1L1;MAD1L1	Body;Body;Body	chr7:1902762-1903059	Island
hypo841	N_excessive_2	1.5E-02	0.79	0.91	-0.11	7	6198703	cg21828663	USP42	Body	chr7:6198495-6198731	Island
hypo842	N_excessive_2	4.4E-06	0.51	0.81	-0.30	8	19243209	cg20220678	SH2D4A	Body		
hypo843	N_excessive_2	6.9E-14	0.49	0.89	-0.40	9	133738155	cg08951958	ABL1;ABL1	Body;Body	chr9:133738154-133738408	Island
hypo844	N_excessive_2	1.2E-13	0.53	0.92	-0.38	10	465366	cg19963795	DIP2C	Body	chr10:465928-466396	N_Shore
hypo845	N_excessive_2	1.9E-05	0.68	0.86	-0.19	10	62150864	cg18175470	ANK3	TSS1500		
hypo846	N_excessive_2	9.4E-11	0.44	0.87	-0.43	10	93665240	cg25579085			chr10:93668002-93668771	N_Shelf
hypo847	N_excessive_2	2.5E-15	0.65	0.94	-0.29	11	1844649	cg07007213			chr11:1847156-1847381	N_Shelf
hypo848	N_excessive_2	1.5E-06	0.58	0.87	-0.29	12	32851191	cg04454629	DNM1L;DNM1L;DNM1L	Body;Body;Body		
hypo849	N_excessive_2	3.0E-02	0.82	0.91	-0.09	14	91765824	cg08214927	CCDC88C	Body		
hypo850	N_excessive_2	4.9E-05	0.67	0.91	-0.25	15	65593468	cg19985346			chr15:65594415-65594649	N_Shore
hypo851	N_excessive_2	3.9E-04	0.67	0.91	-0.24	15	65593688	cg03633565			chr15:65594415-65594649	N_Shore
hypo852	N_excessive_2	1.9E-02	0.46	0.72	-0.25	15	68667977	cg09430158	ITGA11	Body		
hypo853	N_excessive_2	7.1E-11	0.48	0.87	-0.39	17	688002	cg22288657	RNMTL1	Body	chr17:685150-685988	S_Shelf
hypo854	N_excessive_2	1.3E-10	0.47	0.82	-0.35	17	35684812	cg09855384	ACACA;ACACA;ACACA	Body;Body;Body		
hypo855	N_excessive_2	1.2E-09	0.73	0.94	-0.21	17	41836563	cg15596932	SOST	TSS1500	chr17:41832717-41833195	S_Shelf
hypo856	N_excessive_2	8.9E-02	0.82	0.94	-0.12	19	1255283	cg27172769	MIDN	Body	chr19:1255216-1255594	Island
hypo857	N_excessive_2	7.1E-05	0.50	0.75	-0.25	19	1304930	cg26174454			chr19:1304766-1305062	Island
hypo858	N_excessive_2	9.3E-03	0.49	0.74	-0.25	22	29275887	cg17195635			chr22:29279412-29280287	N_Shelf
hypo859	N_excessive_3	4.9E-02	0.67	0.87	-0.20	1	35222920	cg19165942	GJB5	5'UTR		
hypo860	N_excessive_3	4.6E-02	0.80	0.91	-0.11	1	119860821	cg19275091				
hypo861	N_excessive_3	2.6E-02	0.62	0.85	-0.23	1	147760233	cg02887867				
hypo862	N_excessive_3	1.6E-03	0.92	0.98	-0.06	1	156370496	cg13098071				
hypo863	N_excessive_3	6.9E-02	0.83	0.94	-0.11	1	213562740	cg12334079			chr1:213562739-213562958	Island
hypo864	N_excessive_3	3.2E-15	0.55	0.94	-0.39	2	114733020	cg24861013			chr2:114736853-114737689	N_Shelf
hypo865	N_excessive_3	1.3E-02	0.84	0.93	-0.09	2	172975680	cg24576666			chr2:172972804-172973242	S_Shelf
hypo866	N_excessive_3	5.6E-04	0.85	0.98	-0.12	3	126062601	cg06491573	KLF15	Body	chr3:126062535-126062823	Island
hypo867	N_excessive_3	3.5E-02	0.84	0.95	-0.10	3	138648052	cg12967838			chr3:138647671-138648094	Island
hypo868	N_excessive_3	3.6E-03	0.85	0.97	-0.13	3	182782313	cg23476885	MCCC1	Body	chr3:182782174-182782576	Island
hypo869	N_excessive_3	4.0E-02	0.13	0.60	-0.47	4	5710403	cg06426416	EVC2;EVC2	5'UTR;TSS200	chr4:5709985-5710495	Island
hypo870	N_excessive_3	3.9E-02	0.75	0.90	-0.15	4	184404536	cg25018576			chr4:184404501-184404771	Island
hypo871	N_excessive_3	3.4E-07	0.69	0.91	-0.22	5	16703715	cg25363336	MYO10	Body	chr5:16701071-16701775	S_Shore
hypo872	N_excessive_3	2.1E-03	0.43	0.69	-0.27	6	3895506	cg08454922				
hypo873	N_excessive_3	7.6E-02	0.87	0.97	-0.10	6	28829746	cg08363278			chr6:28831682-28832134	N_Shore
hypo874	N_excessive_3	2.3E-02	0.64	0.83	-0.19	6	40101885	cg16345906				
hypo875	N_excessive_3	3.6E-10	0.48	0.84	-0.35	6	53950838	cg09488352	C6orf142	Body		
hypo876	N_excessive_3	3.5E-02	0.60	0.81	-0.21	6	154699017	cg06139720				
hypo877	N_excessive_3	3.4E-03	0.76	0.92	-0.16	6	159550158	cg16681104			chr6:159550082-159550382	Island
hypo878	N_excessive_3	7.1E-08	0.81	0.96	-0.15	6	159550256	cg17453961			chr6:159550082-159550382	Island
hypo879	N_excessive_3	6.9E-12	0.50	0.88	-0.37	6	168387391	cg08737012			chr6:168390141-168390354	N_Shelf
hypo880	N_excessive_3	5.7E-06	0.24	0.55	-0.31	7	12151356	cg16204818			chr7:12151220-12151559	Island
hypo881	N_excessive_3	8.9E-04	0.26	0.66	-0.40	7	12151638	cg04557057			chr7:12151220-12151559	S_Shore
hypo882	N_excessive_3	1.9E-03	0.89	0.96	-0.07	7	144100630	cg11908531	NOBOX	Body		
hypo883	N_excessive_3	1.7E-02	0.82	0.92	-0.10	7	151504864	cg17192599	PRKAG2;PRKAG2	5'UTR;Body		
hypo884	N_excessive_3	1.8E-02	0.69	0.83	-0.14	7	155018256	cg12598606				
hypo885	N_excessive_3	3.8E-02	0.82	0.94	-0.11	7	155019321	cg02388319				
hypo886	N_excessive_3	4.2E-02	0.85	0.94	-0.09	7	155024732	cg10273890				
hypo887	N_excessive_3	9.2E-08	0.29	0.92	-0.63	8	23423757	cg18339359	SLC25A37	Body		
hypo888	N_excessive_3	1.2E-02	0.93	0.98	-0.05	9	4804648	cg13446043	RCL1	Body	chr9:4804490-4804780	Island
hypo889	N_excessive_3	5.7E-06	0.49	0.82	-0.33	9	100704083	cg14116149	HEMGN	5'UTR		
hypo890	N_excessive_3	8.0E-04	0.93	0.98	-0.05	9	111617595	cg21239572	ACTL7B	1stExon	chr9:111617320-111618144	Island
hypo891	N_excessive_3	5.6E-04	0.80	0.94	-0.14	9	111617933	cg13728285	ACTL7B	1stExon	chr9:111617320-111618144	Island
hypo892	N_excessive_3	5.9E-02	0.88	0.96	-0.08	10	736974	cg04586563	DIP2C	TSS1500	chr10:734707-735606	S_Shore
hypo893	N_excessive_3	8.4E-02	0.81	0.89	-0.08	10	25239568	cg17419399	PRTFDC1	Body	chr10:25241110-25241900	N_Shore
hypo894	N_excessive_3	1.2E-02	0.44	0.68	-0.24	10	91015046	cg06293636			chr10:91011325-91011864	S_Shelf
hypo895	N_excessive_3	7.9E-03	0.70	0.87	-0.17	10	98257362	cg11175977	TLL2	Body		
hypo896	N_excessive_3	5.4E-02	0.91	0.98	-0.07	11	64108790	cg16718999	CCDC88B	Body	chr11:64109822-64110228	N_Shore
hypo897	N_excessive_3	1.0E-11	0.50	0.90	-0.40	11	64242815	cg22824291				
hypo898	N_excessive_3	1.4E-02	0.82	0.93	-0.11	11	102142555	cg02672321			chr11:102139424-102139652	S_Shelf

hypo899	N_excessive_3	4.8E-02	0.66	0.82	-0.16	11	134212891	cg12128119	GLB1L2	Body	chr11:134211759-134212611	S_Shore
hypo900	N_excessive_3	9.2E-02	0.87	0.96	-0.10	13	25740027	cg02592525			chr13:25743998-25746127	N_Shelf
hypo901	N_excessive_3	2.7E-02	0.48	0.78	-0.30	13	95711745	cg14808360	ABCC4	Body		
hypo902	N_excessive_3	6.7E-02	0.91	0.97	-0.07	13	110386152	cg26573274				
hypo903	N_excessive_3	5.1E-02	0.89	0.96	-0.07	13	114023882	cg15660684			chr13:114023638-114023919	Island
hypo904	N_excessive_3	2.6E-08	0.46	0.82	-0.36	15	42162712	cg16590643	SPTBN5	Body		
hypo905	N_excessive_3	7.0E-03	0.78	0.88	-0.10	15	45412590	cg26387667	DUOXA1	Body	chr15:45408573-45409528	S_Shelf
hypo906	N_excessive_3	2.3E-03	0.76	0.92	-0.17	15	57611481	cg03904104				
hypo907	N_excessive_3	1.3E-02	0.75	0.87	-0.12	16	75563114	cg25421069	CHST5	Body	chr16:75563113-75564112	Island
hypo908	N_excessive_3	3.4E-05	0.91	0.98	-0.08	17	8012578	cg11310756	ALOXE3;ALOXE3	Body;Body	chr17:8012474-8012846	Island
hypo909	N_excessive_3	1.6E-06	0.50	0.86	-0.36	17	19617253	cg16569407	SLC47A2;SLC47A2	Body;Body	chr17:19617083-19617424	Island
hypo910	N_excessive_3	8.3E-02	0.72	0.87	-0.15	17	29818175	cg24174232	RAB11FIP4	Body	chr17:29814614-29815662	S_Shelf
hypo911	N_excessive_3	3.8E-04	0.90	0.97	-0.07	17	79792777	cg26066277	DYSFIP1	1stExon	chr17:79790530-79791553	S_Shore
hypo912	N_excessive_3	7.8E-11	0.51	0.86	-0.36	17	80772942	cg06796120	TBCD	Body	chr17:80772792-80773003	Island
hypo913	N_excessive_3	1.4E-09	0.57	0.89	-0.32	18	60245587	cg13863769	ZCCHC2	3'UTR		
hypo914	N_excessive_3	7.6E-08	0.91	0.99	-0.08	19	5455843	cg22694548	ZNRF4	1stExon	chr19:5455481-5456262	Island
hypo915	N_excessive_3	3.1E-02	0.78	0.90	-0.12	19	41630815	cg07750118	CYP2F1	Body	chr19:41630485-41630794	S_Shore
hypo916	N_excessive_3	7.0E-10	0.11	0.68	-0.57	19	47123947	cg19043207	PTGIR	3'UTR	chr19:47126782-47127478	N_Shelf
hypo917	N_excessive_3	1.7E-11	0.45	0.78	-0.33	19	47341466	cg25230305	AP2S1;AP2S1	3'UTR;3'UTR		
hypo918	N_excessive_3	1.1E-05	0.91	0.98	-0.07	19	52216726	cg02261174	HAS1	Body	chr19:52216659-52217389	Island
hypo919	N_excessive_3	1.2E-06	0.45	0.73	-0.28	22	18386798	cg21563049	MICAL3;MICAL3;MICAL3	Body;Body;Body		
hypo920	N_excessive_3	4.9E-10	0.53	0.87	-0.34	22	38155575	cg22615910	TRIOBP;TRIOBP;TRIOBP	Body;3'UTR;Body		
hypo921	N_excessive_3	5.8E-03	0.88	0.97	-0.09	22	46770644	cg24968629	CELSR1	Body	chr22:46770433-46770871	Island
hypo922	N_excessive_4	4.7E-09	0.51	0.87	-0.36	1	26394009	cg04489573	TRIM63;TRIM63	1stExon;5'UTR		
hypo923	N_excessive_4	9.6E-03	0.41	0.68	-0.27	2	70186181	cg20199770			chr2:70188511-70188726	N_Shelf
hypo924	N_excessive_4	1.3E-10	0.55	0.91	-0.35	2	176042976	cg04928692	ATP5G3;ATP5G3	3'UTR;3'UTR	chr2:176045969-176046504	N_Shelf
hypo925	N_excessive_4	1.3E-02	0.79	0.89	-0.09	2	242664612	cg14409746	ING5	3'UTR	chr2:242664207-242665154	Island
hypo926	N_excessive_4	6.2E-02	0.47	0.77	-0.30	3	108191083	cg01135165	MYH15	Body		
hypo927	N_excessive_4	2.9E-10	0.60	0.90	-0.29	3	156546908	cg17778101	LEKR1	5'UTR	chr3:156543891-156544405	S_Shelf
hypo928	N_excessive_4	9.7E-10	0.51	0.85	-0.34	3	182694617	cg13265572	DCUN1D1	Body	chr3:182697749-182698731	N_Shelf
hypo929	N_excessive_4	3.4E-03	0.71	0.88	-0.17	5	20306611	cg22831264			chr5:20305562-20305936	S_Shore
hypo930	N_excessive_4	6.1E-14	0.56	0.93	-0.36	6	30026181	cg06193668	NCRNA00171	Body	chr6:30028936-30029195	N_Shelf
hypo931	N_excessive_4	9.0E-05	0.47	0.77	-0.30	6	31761062	cg10807309	VARS	Body	chr6:31763240-31763905	N_Shelf
hypo932	N_excessive_4	5.0E-05	0.59	0.93	-0.34	6	68535378	cg12086421				
hypo933	N_excessive_4	7.2E-02	0.70	0.83	-0.13	8	21937728	cg16782396	EPB49;EPB49;EPB49;EPB49;EP	Body;Body;Body;Body;Body;Body		
hypo934	N_excessive_4	1.5E-14	0.51	0.90	-0.39	8	145085954	cg03722185	SPATC1;SPATC1	TSS1500;TSS1500		
hypo935	N_excessive_4	3.3E-08	0.55	0.87	-0.32	9	95993065	cg14655590	WNK2	Body		
hypo936	N_excessive_4	2.8E-04	0.60	0.85	-0.24	9	104147168	cg05770171	BAAT;BAAT;BAAT	TSS1500;1stExon;5'UTR		
hypo937	N_excessive_4	2.0E-02	0.54	0.88	-0.34	10	10202925	cg05201317				
hypo938	N_excessive_4	3.7E-02	0.12	0.84	-0.73	10	111912029	cg24051234				
hypo939	N_excessive_4	6.9E-09	0.53	0.83	-0.30	11	8761869	cg08592104	ST5;ST5;ST5	Body;Body;Body		
hypo940	N_excessive_4	1.4E-04	0.51	0.80	-0.29	12	14765994	cg27486692	GUCY2C	3'UTR		
hypo941	N_excessive_4	7.4E-12	0.55	0.91	-0.36	14	24683542	cg26407029	MDP1;CHMP4A	Body;TSS1500	chr14:24682402-24682837	S_Shore
hypo942	N_excessive_4	5.4E-07	0.53	0.85	-0.32	14	73073497	cg18263767				
hypo943	N_excessive_4	1.6E-08	0.61	0.88	-0.26	16	31515409	cg27592424	C16orf58	Body	chr16:31519381-31519940	N_Shelf
hypo944	N_excessive_4	1.3E-07	0.69	0.93	-0.24	17	1839591	cg18059383	RTN4RL1	3'UTR	chr17:1839989-1841209	N_Shore
hypo945	N_excessive_4	5.7E-15	0.46	0.92	-0.46	19	3201528	cg13689497	NCLN	Body	chr19:3201455-3201681	Island
hypo946	N_excessive_4	1.4E-09	0.52	0.83	-0.31	20	2847327	cg13630574	VPS16;PTPRA;VPS16	3'UTR;5'UTR;3'UTR		
hypo947	N_excessive_5	1.1E-05	0.45	0.86	-0.41	1	9308197	cg11150353	H6PD	Body		
hypo948	N_excessive_5	4.6E-07	0.59	0.89	-0.30	2	101635691	cg07297467	RPL31;TBC1D8	3'UTR;Body		
hypo949	N_excessive_5	2.8E-06	0.52	0.82	-0.31	3	155458975	cg04078644			chr3:155461917-155462561	N_Shelf
hypo950	N_excessive_5	1.6E-10	0.54	0.87	-0.33	4	24567220	cg04436083	DHX15	Body		
hypo951	N_excessive_5	1.0E-03	0.75	0.90	-0.15	4	187524686	cg24997677	FAT1	Body		
hypo952	N_excessive_5	6.3E-03	0.72	0.89	-0.17	4	187527265	cg11972666	FAT1	Body		
hypo953	N_excessive_5	1.3E-07	0.49	0.77	-0.28	5	131599890	cg02484352	PDLIM4;PDLIM4	Body;Body		
hypo954	N_excessive_5	7.4E-05	0.55	0.82	-0.27	6	89340909	cg15692086	RNGTT	Body		
hypo955	N_excessive_5	5.5E-06	0.57	0.87	-0.31	6	161432631	cg25138713	MAP3K4;MAP3K4	Body;Body		
hypo956	N_excessive_5	1.2E-02	0.60	0.88	-0.28	7	39298343	cg27443265	POU6F2;POU6F2	Body;Body		
hypo957	N_excessive_5	1.6E-04	0.66	0.86	-0.20	7	39305238	cg10874881	POU6F2;POU6F2	Body;Body		
hypo958	N_excessive_5	1.9E-12	0.14	0.56	-0.42	7	94286267	cg27001184	SGCE;PEG10;SGCE;PEG10;SG	TSS1500;5'UTR;TSS1500;5'UTR;	chr7:94284858-94286527	Island
hypo959	N_excessive_5	3.7E-07	0.68	0.87	-0.19	7	149557121	cg01844514	ZNF862	Body	chr7:149558420-149558665	N_Shore
hypo960	N_excessive_5	2.4E-14	0.57	0.91	-0.35	8	39950015	cg10139476				
hypo961	N_excessive_5	5.5E-05	0.14	0.44	-0.30	9	86153558	cg21197678	FRMD3	TSS1500	chr9:86152353-86153777	Island
hypo962	N_excessive_5	1.3E-11	0.55	0.85	-0.30	10	71570779	cg23386779	COL13A1;COL13A1;COL13A1;C	Body;Body;Body;Body;Body;Body;Body;Body;Body;Body;Body;Body;Body;Body;Body;Body		
hypo963	N_excessive_5	2.0E-02	0.60	0.83	-0.24	10	98982314	cg17107501	ARHGAP19	3'UTR		
hypo964	N_excessive_5	5.2E-11	0.55	0.91	-0.36	10	133747849	cg07454788	PPP2R2D;PPP2R2D;PPP2R2D	TSS200;TSS200;TSS200	chr10:133751399-133751634	N_Shelf
hypo965	N_excessive_5	7.1E-05	0.40	0.76	-0.36	11	2397685	cg03848831	CD81	TSS1500	chr11:2398223-2399598	N_Shore
hypo966	N_excessive_5	4.8E-04	0.44	0.70	-0.26	11	66457621	cg23149790	SPTBN2	Body	chr11:66458783-66459123	N_Shore
hypo967	N_excessive_5	7.9E-10	0.64	0.89	-0.25	16	1369367	cg08969217	UBE2I;UBE2I;UBE2I;UBE2I	Body;Body;Body;Body	chr16:1369053-1369525	Island
hypo968	N_excessive_5	1.5E-11	0.52	0.90	-0.37	16	81517665	cg05499338	CMIP	Body		
hypo969	N_excessive_5	2.5E-04	0.51	0.80	-0.29	17	9871831	cg16983282	GAS7;GAS7;GAS7	Body;Body;Body		
hypo970	N_excessive_5	2.7E-06	0.37	0.89	-0.52	17	46608329	cg24900666	HOXB1	TSS200	chr17:46607804-46608390	Island
hypo971	N_excessive_5	6.6E-02	0.23	0.46	-0.23	19	10304766	cg19803081	DNMT1;DNMT1	Body;Body	chr19:10304966-10305864	N_Shore
hypo972	N_excessive_5	3.3E-03	0.39	0.58	-0.19	19	10305305	cg17445987	DNMT1;DNMT1	Body;Body	chr19:10304966-10305864	Island
hypo973	N_excessive_5	5.4E-06	0.42	0.58	-0.16	19	10305755	cg23401624	DNMT1;DNMT1	TSS200;TSS200	chr19:10304966-10305864	Island
hypo974	N_excessive_5	4.2E-03	0.37	0.53	-0.16	19	10305780	cg24477899	DNMT1;DNMT1	TSS200;TSS200	chr19:10304966-10305864	Island
hypo975	N_excessive_5	3.7E-02	0.25	0.49	-0.24	19	10305863	cg10635912	DNMT1;DNMT1	TSS200;TSS200	chr19:10304966-10305864	Island
hypo976	N_excessive_5	1.8E-02	0.23	0.41	-0.18	19	10305911	cg15043801	DNMT1;DNMT1	TSS200;TSS200	chr19:10304966-10305864	S_Shore
hypo977	N_excessive_5	2.3E-04	0.47	0.82	-0.35	19	53403009	cg06917858			chr19:53400460-53401205	S_Shore

Supplementary Table3: Full results of Gene ontlogy term analysis for the full and partial lists of hyper- and hypo-methylated outliers using DAVID

Hypermethylated_all (2521-outliers in Table S1)_1001-genes

Category	Term	Count	%	PValue	Genes	List Total	Pop Hits	Pop Total	Fold Enrichment	Bonferroni	Benjamini	FDR
GOTERM_BP_FAT	GO:0006355~regulation of transcription, DNA-dependent	157	15.8	6.7E-12	MEF2C, ZNF582, HMX2, HMGN:	704	1773	13528	1.70	2.0E-08	2.0E-08	1.2E-08
GOTERM_BP_FAT	GO:0030182~neuron differentiation	60	6.0	1.3E-11	FOXA2, TUBB2B, WNT3A, PAX6	704	438	13528	2.63	3.8E-08	1.9E-08	2.3E-08
GOTERM_BP_FAT	GO:0051252~regulation of RNA metabolic process	157	15.8	3.9E-11	MEF2C, ZNF582, HMX2, HMGN:	704	1813	13528	1.66	1.1E-07	3.7E-08	6.9E-08
GOTERM_BP_FAT	GO:0045449~regulation of transcription	199	20.1	2.4E-09	MEF2C, HMGN2, HOXD13, ZNF	704	2601	13528	1.47	7.0E-06	1.7E-06	4.3E-06
GOTERM_BP_FAT	GO:0007409~axonogenesis	31	3.1	6.6E-08	WNT3A, PAX6, PAX2, CXCL12, I	704	193	13528	3.09	1.9E-04	3.9E-05	1.2E-04

Category	Term	Count	%	PValue	Genes	List Total	Pop Hits	Pop Total	Fold Enrichment	Bonferroni	Benjamini	FDR
GOTERM_CC_FAT	GO:0044456~synapse part	27	2.7	1.9E-04	RAB3C, GABRB3, SYT5, GABRE	628	246	12782	2.23	7.5E-02	7.5E-02	2.6E-01
GOTERM_CC_FAT	GO:0045202~synapse	34	3.4	3.2E-04	RAB3C, GABRB3, TLN2, SYT5, (	628	355	12782	1.95	1.2E-01	6.3E-02	4.4E-01
GOTERM_CC_FAT	GO:0031012~extracellular matrix	32	3.2	8.4E-04	ADAMTS17, ADAMTS19, ADAM	628	345	12782	1.89	2.9E-01	1.1E-01	1.2E+00
GOTERM_CC_FAT	GO:0044459~plasma membrane part	137	13.8	1.9E-03	SLC6A1, GPR125, GABRB3, SY	628	2203	12782	1.27	5.4E-01	1.8E-01	2.6E+00
GOTERM_CC_FAT	GO:0005578~proteinaceous extracellular matrix	29	2.9	2.2E-03	ADAMTS17, ADAMTS19, ADAM	628	320	12782	1.84	5.9E-01	1.7E-01	3.0E+00

Category	Term	Count	%	PValue	Genes	List Total	Pop Hits	Pop Total	Fold Enrichment	Bonferroni	Benjamini	FDR
GOTERM_MF_FAT	GO:0003700~transcription factor activity	103	10.4	2.9E-11	MEF2C, HMX2, HOXD13, RORB	699	975	12983	1.96	2.5E-08	2.5E-08	4.5E-08
GOTERM_MF_FAT	GO:0043565~sequence-specific DNA binding	73	7.4	1.4E-10	MEF2C, HNF1B, HMX2, FOSL2,	699	607	12983	2.23	1.2E-07	5.9E-08	2.1E-07
GOTERM_MF_FAT	GO:0030528~transcription regulator activity	129	13.0	7.0E-08	MEF2C, HMX2, HOXD13, RORB	699	1512	12983	1.58	5.9E-05	2.0E-05	1.1E-04
GOTERM_MF_FAT	GO:0003677~DNA binding	175	17.6	1.3E-06	MEF2C, ZNF582, HMX2, HMGN:	699	2331	12983	1.39	1.1E-03	2.8E-04	2.0E-03
GOTERM_MF_FAT	GO:0043167~ion binding	268	27.0	7.9E-04	SYT5, PTGS1, SYT9, ZNF250, F:	699	4241	12983	1.17	4.9E-01	1.3E-01	1.2E+00

Hypermethylated_delta-beta >0.2_945-outliers_409-genes

Category	Term	Count	%	PValue	Genes	List Total	Pop Hits	Pop Total	Fold Enrichment	Bonferroni	Benjamini	FDR
GOTERM_BP_FAT	GO:0006355~regulation of transcription, DNA-dependent	82	20.2	1.3E-11	ZNF19, ZNF582, HNF1B, HMX2,	292	1773	13528	2.14	2.3E-08	2.3E-08	2.2E-08
GOTERM_BP_FAT	GO:0051252~regulation of RNA metabolic process	82	20.2	4.0E-11	ZNF19, ZNF582, HNF1B, HMX2,	292	1813	13528	2.10	7.2E-08	3.6E-08	6.8E-08
GOTERM_BP_FAT	GO:0045449~regulation of transcription	97	24.0	1.3E-08	ZNF582, HMX2, ZNF583, RORA	292	2601	13528	1.73	2.4E-05	7.9E-06	2.2E-05
GOTERM_BP_FAT	GO:0006350~transcription	79	19.5	4.4E-07	ZNF19, ZNF582, HNF1B, HMX2,	292	2101	13528	1.74	8.0E-04	2.0E-04	7.5E-04
GOTERM_BP_FAT	GO:0030182~neuron differentiation	26	6.4	9.0E-06	PAX6, RORA, RTN1, TUBB, S1P	292	438	13528	2.75	1.6E-02	3.3E-03	1.5E-02

Category	Term	Count	%	PValue	Genes	List Total	Pop Hits	Pop Total	Fold Enrichment	Bonferroni	Benjamini	FDR
GOTERM_CC_FAT	GO:0005578~proteinaceous extracellular matrix	16	4.0	7.9E-04	FLRT3, WNT10A, ADAMTS17, A	234	320	12782	2.73	1.9E-01	1.9E-01	1.0E+00
GOTERM_CC_FAT	GO:0031012~extracellular matrix	16	4.0	1.7E-03	FLRT3, WNT10A, ADAMTS17, A	234	345	12782	2.53	3.5E-01	1.9E-01	2.1E+00
GOTERM_CC_FAT	GO:0043204~perikaryon	4	1.0	1.7E-02	MAP1B, ENO2, ESR1, TTLL7	234	30	12782	7.28	9.9E-01	7.7E-01	2.0E+01
GOTERM_CC_FAT	GO:0045121~membrane raft	7	1.7	4.7E-02	RGMB, TUBB, SLC6A2, MDC1, (	234	143	12782	2.67	1	9.6E-01	4.7E+01
GOTERM_CC_FAT	GO:0005901~caveola	4	1.0	6.9E-02	TUBB, MDC1, IRS1, F2R	234	52	12782	4.20	1	9.8E-01	6.1E+01

Category	Term	Count	%	PValue	Genes	List Total	Pop Hits	Pop Total	Fold Enrichment	Bonferroni	Benjamini	FDR
GOTERM_MF_FAT	GO:0003700~transcription factor activity	50	12.3	4.7E-08	ZNF19, HMX2, HNF1B, BACH2,	289	975	12983	2.30	2.3E-05	2.3E-05	6.7E-05
GOTERM_MF_FAT	GO:0043565~sequence-specific DNA binding	35	8.6	6.2E-07	HMX2, HNF1B, FOSL2, BACH2,	289	607	12983	2.59	3.0E-04	1.5E-04	8.9E-04
GOTERM_MF_FAT	GO:0003677~DNA binding	84	20.7	3.3E-06	ZNF19, ZNF582, HNF1B, HMX2,	289	2331	12983	1.62	1.6E-03	5.3E-04	4.7E-03
GOTERM_MF_FAT	GO:0030528~transcription regulator activity	58	14.3	3.7E-05	ZNF19, HMX2, HNF1B, BACH2,	289	1512	12983	1.72	1.8E-02	4.5E-03	5.3E-02
GOTERM_MF_FAT	GO:0008270~zinc ion binding	74	18.3	7.5E-04	ZNF19, ZNF582, NPEPL1, ZNF5	289	2311	12983	1.44	3.0E-01	7.0E-02	1.1E+00

Hypermethylated_pTSS_1631-outliers_790genes

Category	Term	Count	%	PValue	Genes	List Total	Pop Hits	Pop Total	Fold Enrichment	Bonferroni	Benjamini	FDR
GOTERM_BP_FAT	GO:0006355~regulation of transcription, DNA-dependent	126	16.0	1.4E-09	ZNF582, MEF2C, HMX2, ZNF58:	568	1773	13528	1.69	3.9E-06	3.9E-06	2.6E-06
GOTERM_BP_FAT	GO:0051252~regulation of RNA metabolic process	126	16.0	5.7E-09	ZNF582, MEF2C, HMX2, ZNF58:	568	1813	13528	1.66	1.5E-05	7.7E-06	1.0E-05
GOTERM_BP_FAT	GO:0030182~neuron differentiation	47	6.0	8.4E-09	FOXA2, TUBB2B, PAX6, RORB,	568	438	13528	2.56	2.3E-05	7.5E-06	1.5E-05
GOTERM_BP_FAT	GO:0045449~regulation of transcription	161	20.5	7.6E-08	MEF2C, ZNF582, HMX2, ZNF58:	568	2601	13528	1.47	2.1E-04	5.2E-05	1.4E-04
GOTERM_BP_FAT	GO:0006350~transcription	135	17.2	1.7E-07	ZNF582, MEF2C, HMX2, ZNF58:	568	2101	13528	1.53	4.6E-04	9.1E-05	3.0E-04

Category	Term	Count	%	PValue	Genes	List Total	Pop Hits	Pop Total	Fold Enrichment	Bonferroni	Benjamini	FDR
GOTERM_CC_FAT	GO:0044456~synapse part	24	3.1	8.8E-05	GABRD, ARC, GLRB, KCND2, G	497	246	12782	2.51	3.3E-02	3.3E-02	1.2E-01
GOTERM_CC_FAT	GO:0044459~plasma membrane part	117	14.9	2.2E-04	SLC6A1, GPR125, GABRB3, SY	497	2203	12782	1.37	8.2E-02	4.2E-02	3.1E-01
GOTERM_CC_FAT	GO:0031226~intrinsic to plasma membrane	72	9.2	2.8E-04	SLC6A1, GABRB3, GABRB2, SL	497	1215	12782	1.52	1.0E-01	3.5E-02	3.9E-01
GOTERM_CC_FAT	GO:0045202~synapse	29	3.7	3.0E-04	GABRB3, SYT5, GABRB2, GABE	497	355	12782	2.10	1.1E-01	2.8E-02	4.1E-01
GOTERM_CC_FAT	GO:0045211~postsynaptic membrane	15	1.9	7.5E-04	GABRD, GLRB, ARC, GABRB3,	497	135	12782	2.86	2.5E-01	5.6E-02	1.0E+00

Category	Term	Count	%	PValue	Genes	List Total	Pop Hits	Pop Total	Fold Enrichment	Bonferroni	Benjamini	FDR
GOTERM_MF_FAT	GO:0003700~transcription factor activity	83	10.6	4.8E-09	ZNF19, MEF2C, HMX2, FOSL2,	568	975	12983	1.95	3.6E-06	3.6E-06	7.3E-06
GOTERM_MF_FAT	GO:0043565~sequence-specific DNA binding	59	7.5	1.4E-08	MEF2C, HMX2, FOSL2, BACH2,	568	607	12983	2.22	1.0E-05	5.2E-06	2.1E-05
GOTERM_MF_FAT	GO:0030528~transcription regulator activity	102	13.0	6.7E-06	MEF2C, HMX2, HOXD13, RORB	568	1512	12983	1.54	5.0E-03	1.7E-03	1.0E-02
GOTERM_MF_FAT	GO:0003677~DNA binding	142	18.1	1.6E-05	MEF2C, ZNF582, HMX2, ZNF58:	568	2331	12983	1.39	1.2E-02	2.9E-03	2.4E-02
GOTERM_MF_FAT	GO:0043167~ion binding	225	28.6	2.7E-04	SYT5, PTGS1, SYT9, ZNF250, F:	568	4241	12983	1.21	1.9E-01	4.0E-02	4.1E-01

Hypermethylated_clustered_1407-outliers_317-genes

Category	Term	Count	%	PValue	Genes	List Total	Pop Hits	Pop Total	Fold Enrichment	Bonferroni	Benjamini	FDR
GOTERM_BP_FAT	GO:0006355~regulation of transcription, DNA-dependent	52	16.5	5.7E-05	HMX2, ZNF583, PAX6, ZNF250,	227	1773	13528	1.75	8.4E-02	8.4E-02	9.5E-02
GOTERM_BP_FAT	GO:0051252~regulation of RNA metabolic process	52	16.5	1.0E-04	HMX2, ZNF583, PAX6, ZNF250,	227	1813	13528	1.71	1.4E-01	7.5E-02	1.7E-01
GOTERM_BP_FAT	GO:0045449~regulation of transcription	66	21.0	3.1E-04	HMX2, ZNF518B, ZNF583, PAX6	227	2601	13528	1.51	3.8E-01	1.5E-01	5.2E-01
GOTERM_BP_FAT	GO:0030182~neuron differentiation	19	6.0	4.1E-04	DFNA5, TUBB2B, PTPRZ1, MAP	227	438	13528	2.59	4.6E-01	1.4E-01	6.8E-01
GOTERM_BP_FAT	GO:0006350~transcription	55	17.5	6.2E-04	HMX2, ZNF518B, ZNF583, PAX6	227	2101	13528	1.56	6.1E-01	1.7E-01	1.0E+00

Category	Term	Count	%	PValue	Genes	List Total	Pop Hits	Pop Total	Fold Enrichment	Bonferroni	Benjamini	FDR
GOTERM_CC_FAT	GO:0005875~microtubule associated complex	6	1.9	1.9E-02	DNAH11, DNAH10, PEA15, MAF	193	102	12782	3.90	9.9E-01	9.9E-01	2.2E+01
GOTERM_CC_FAT	GO:0044463~cell projection part	9	2.9	2.5E-02	DNAH11, DNAH10, NPHP4, SPA	193	234	12782	2.55	1	9.5E-01	2.8E+01
GOTERM_CC_FAT	GO:0031980~mitochondrial lumen	8	2.5	5.5E-02	MCCC2, NAGS, SUCLG2, HSPE	193	227	12782	2.33	1	9.9E-01	5.2E+01
GOTERM_CC_FAT	GO:0005759~mitochondrial matrix	8	2.5	5.5E-02	MCCC2, NAGS, SUCLG2, HSPE	193	227	12782	2.33	1	9.9E-01	5.2E+01
GOTERM_CC_FAT	GO:0031012~extracellular matrix	10	3.2	7.6E-02	FLRT3, COL4A3, ADAMTS19, P'	193	345	12782	1.92	1	9.9E-01	6.4E+01

Category	Term	Count	%	PValue	Genes	List Total	Pop Hits	Pop Total	Fold Enrichment	Bonferroni	Benjamini	FDR
GOTERM_MF_FAT	GO:0003700~transcription factor activity	34	10.8	4.4E-05	HMX2, ZNF132, PAX6, MYEF2, (	212	975	12983	2.14	1.6E-02	1.6E-02	6.0E-02
GOTERM_MF_FAT	GO:0043565~sequence-specific DNA binding	23	7.3	3.6E-04	FOXL2, HMX2, ESR1, PAX6, ZH	212	607	12983	2.32	1.3E-01	6.6E-02	5.0E-01
GOTERM_MF_FAT	GO:0003677~DNA binding	59	18.7	3.7E-04	HMX2, ZNF518B, ZNF583, PAX6	212	2331	12983	1.55	1.3E-01	4.6E-02	5.1E-01
GOTERM_MF_FAT	GO:0030528~transcription regulator activity	40	12.7	2.2E-03	HMX2, ZNF132, PAX6, MYEF2, (	212	1512	12983	1.62	5.7E-01	1.9E-01	3.1E+00
GOTERM_MF_FAT	GO:0005158~insulin receptor binding	4	1.3	1.0E-02	IRS2, DOK6, DOK7, IRS1	212	28	12983	8.75	9.8E-01	5.4E-01	1.3E+01

Hypermethylated_delta-beta >0.2&pTSS&clustered_419-outliers_163-genes

Category	Term	Count	%	PValue	Genes	List Total	Pop Hits	Pop Total	Fold Enrichment	Bonferroni	Benjamini	FDR
GOTERM_BP_FAT	GO:0006355~regulation of transcription, DNA-dependent	36	22.1	3.3E-06	HMX2, ZNF583, ZNF805, PAX6,	122	1773	13528	2.25	3.8E-03	3.8E-03	5.2E-03
GOTERM_BP_FAT	GO:0051252~regulation of RNA metabolic process	36	22.1	5.4E-06	HMX2, ZNF583, ZNF805, PAX6,	122	1813	13528	2.20	6.3E-03	3.2E-03	8.7E-03
GOTERM_BP_FAT	GO:0045449~regulation of transcription	44	27.0	1.7E-05	HMX2, ZNF583, ZNF518B, ZNF	122	2601	13528	1.88	2.0E-02	6.8E-03	2.8E-02
GOTERM_BP_FAT	GO:0006350~transcription	38	23.3	2.2E-05	HMX2, ZNF518B, ZNF583, ZNF	122	2101	13528	2.01	2.6E-02	6.5E-03	3.6E-02
GOTERM_BP_FAT	GO:0051789~response to protein stimulus	5	3.1	1.6E-02	HSPA2, MAP1B, HSPE1, HSPD1	122	107	13528	5.18	1	9.7E-01	2.2E+01

Category	Term	Count	%	PValue	Genes	List Total	Pop Hits	Pop Total	Fold Enrichment	Bonferroni	Benjamini	FDR
GOTERM_CC_FAT	GO:0005759~mitochondrial matrix	6	3.7	2.3E-02	SUCLG2, HSPE1, HSPD1, HADI	92	227	12782	3.67	9.8E-01	9.8E-01	2.5E+01
GOTERM_CC_FAT	GO:0031980~mitochondrial lumen	6	3.7	2.3E-02	SUCLG2, HSPE1, HSPD1, HADI	92	227	12782	3.67	9.8E-01	9.8E-01	2.5E+01
GOTERM_CC_FAT	GO:0044429~mitochondrial part	10	6.1	2.6E-02	GPD2, SUCLG2, HSPE1, HSPD	92	595	12782	2.34	9.9E-01	8.8E-01	2.7E+01
GOTERM_CC_FAT	GO:0005578~proteinaceous extracellular matrix	6	3.7	7.8E-02	ADAMTS19, PTPRZ1, NTN4, NT	92	320	12782	2.61	1	9.9E-01	6.3E+01
GOTERM_CC_FAT	GO:0044463~cell projection part	5	3.1	8.6E-02	DNAH10, SPAG6, PDE4A, ESR1	92	234	12782	2.97	1	9.8E-01	6.6E+01

Category	Term	Count	%	PValue	Genes	List Total	Pop Hits	Pop Total	Fold Enrichment	Bonferroni	Benjamini	FDR
GOTERM_MF_FAT	GO:0003677~DNA binding	39	23.9	2.2E-04	HMX2, ZNF518B, ZNF583, ZNF	122	2331	12983	1.78	5.9E-02	5.9E-02	2.9E-01
GOTERM_MF_FAT	GO:0003700~transcription factor activity	21	12.9	6.5E-04	FOXL2, HMX2, ESR1, PAX6, ZH	122	975	12983	2.29	1.6E-01	8.5E-02	8.6E-01
GOTERM_MF_FAT	GO:0043565~sequence-specific DNA binding	14	8.6	4.3E-03	HMX2, FOXL2, ESR1, PAX6, ZH	122	607	12983	2.45	6.9E-01	3.2E-01	5.5E+00
GOTERM_MF_FAT	GO:0003705~RNA polymerase II transcription factor activity, enhanc	4	2.5	5.7E-03	MYEF2, KCNH8, FOXC1, PROX	122	39	12983	10.91	7.9E-01	3.2E-01	7.2E+00
GOTERM_MF_FAT	GO:0030528~transcription regulator activity	23	14.1	2.2E-02	FOXL2, HMX2, ESR1, PAX6, ZH	122	1512	12983	1.62	1	7.0E-01	2.6E+01

Hypomethylated_all (977-outliers in Table S2)_606-genes

Category	Term	Count	%	PValue	Genes	List Total	Pop Hits	Pop Total	Fold Enrichment	Bonferroni	Benjamini	FDR
GOTERM_BP_FAT	GO:0050690~regulation of defense response to virus by virus	4	0.66	1.4E-03	AP2B1, AP2A2, CD8B, AP2S1	414	8	13528	16.34	9.5E-01	9.5E-01	2.4E+00
GOTERM_BP_FAT	GO:0030036~actin cytoskeleton organization	17	2.82	1.6E-03	TRIOBP, PRKCZ, SHROOM3, TI	414	226	13528	2.46	9.7E-01	8.1E-01	2.7E+00
GOTERM_BP_FAT	GO:0030029~actin filament-based process	17	2.82	3.0E-03	TRIOBP, PRKCZ, SHROOM3, TI	414	241	13528	2.30	1.0E+00	8.8E-01	5.1E+00
GOTERM_BP_FAT	GO:0031333~negative regulation of protein complex assembly	6	1.00	3.5E-03	TRIOBP, PRKCZ, SPTBN5, SPTI	414	34	13528	5.77	1.0E+00	8.4E-01	5.8E+00
GOTERM_BP_FAT	GO:0051693~actin filament capping	5	0.83	4.0E-03	TRIOBP, SPTBN5, SPTBN2, SPT	414	22	13528	7.43	1.0E+00	8.2E-01	6.8E+00

Category	Term	Count	%	PValue	Genes	List Total	Pop Hits	Pop Total	Fold Enrichment	Bonferroni	Benjamini	FDR
GOTERM_CC_FAT	GO:0015629~actin cytoskeleton	21	3.48	1.5E-04	TRIOBP, PRKCZ, MYH15, SPTB	379	269	12782	2.63	5.5E-02	5.5E-02	2.1E-01
GOTERM_CC_FAT	GO:0005856~cytoskeleton	63	10.45	5.1E-04	MAD1L1, MYH15, HINT1, FERM	379	1381	12782	1.54	1.7E-01	9.1E-02	7.0E-01
GOTERM_CC_FAT	GO:0044433~cytoplasmic vesicle part	15	2.49	1.3E-03	AP1G2, AP2S1, ACTN1, SPARC	379	187	12782	2.71	4.0E-01	1.5E-01	1.8E+00
GOTERM_CC_FAT	GO:0008091~spectrin	4	0.66	1.9E-03	EPB41L2, SPTBN5, SPTBN2, SF	379	9	12782	14.99	5.1E-01	1.6E-01	2.6E+00
GOTERM_CC_FAT	GO:0043292~contractile fiber	11	1.82	3.1E-03	HDAC4, MYH15, NEB, SORBS2,	379	121	12782	3.07	6.9E-01	2.1E-01	4.2E+00

Category	Term	Count	%	PValue	Genes	List Total	Pop Hits	Pop Total	Fold Enrichment	Bonferroni	Benjamini	FDR
GOTERM_MF_FAT	GO:0008092~cytoskeletal protein binding	37	6.14	4.1E-06	TRIOBP, MYH15, SHROOM3, AE	411	504	12983	2.32	2.5E-03	2.5E-03	6.1E-03
GOTERM_MF_FAT	GO:0003779~actin binding	23	3.81	6.9E-04	TRIOBP, SHROOM3, MYH15, SI	411	326	12983	2.23	3.4E-01	1.9E-01	1.0E+00
GOTERM_MF_FAT	GO:0030695~GTPase regulator activity	24	3.98	4.7E-03	GIT1, SERGEF, ABR, ARHGEF1	411	404	12983	1.88	9.5E-01	6.2E-01	6.8E+00
GOTERM_MF_FAT	GO:0060589~nucleoside-triphosphatase regulator activity	24	3.98	6.1E-03	GIT1, SERGEF, ABR, ARHGEF1	411	413	12983	1.84	9.8E-01	6.1E-01	8.7E+00
GOTERM_MF_FAT	GO:0005083~small GTPase regulator activity	18	2.99	6.3E-03	GIT1, SERGEF, ABR, ARHGEF1	411	274	12983	2.08	9.8E-01	5.4E-01	8.9E+00

Hypomethylated_delta-beta<-0.2_729-outliers_483-genes

Category	Term	Count	%	PValue	Genes	List Total	Pop Hits	Pop Total	Fold Enrichment	Bonferroni	Benjamini	FDR
GOTERM_BP_FAT	GO:0030036~actin cytoskeleton organization	16	3.33	4.1E-04	PRKCZ, TRIOBP, SHROOM3, TI	329	226	13528	2.91	5.4E-01	5.4E-01	7.0E-01
GOTERM_BP_FAT	GO:0050690~regulation of defense response to virus by virus	4	0.83	7.2E-04	AP2B1, AP2A2, CD8B, AP2S1	329	8	13528	20.56	7.4E-01	4.9E-01	1.2E+00
GOTERM_BP_FAT	GO:0030029~actin filament-based process	16	3.33	8.1E-04	PRKCZ, TRIOBP, SHROOM3, TI	329	241	13528	2.73	7.8E-01	4.0E-01	1.4E+00
GOTERM_BP_FAT	GO:0032989~cellular component morphogenesis	20	4.16	3.9E-03	DNM1L, SHROOM3, RTN4RL1,	329	397	13528	2.07	1	8.4E-01	6.4E+00
GOTERM_BP_FAT	GO:0002831~regulation of response to biotic stimulus	5	1.04	5.0E-03	PVR, AP2B1, AP2A2, CD8B, AP2	329	29	13528	7.09	1	8.5E-01	8.2E+00

Category	Term	Count	%	PValue	Genes	List Total	Pop Hits	Pop Total	Fold Enrichment	Bonferroni	Benjamini	FDR
GOTERM_CC_FAT	GO:0015629~actin cytoskeleton	18	3.74	2.0E-04	TRIOBP, PRKCZ, MYH15, SPTB	300	269	12782	2.85	7.0E-02	7.0E-02	2.8E-01
GOTERM_CC_FAT	GO:0044433~cytoplasmic vesicle part	13	2.70	1.5E-03	AP1G2, AP2S1, ACTN1, SPARC	300	187	12782	2.96	4.2E-01	2.4E-01	2.0E+00
GOTERM_CC_FAT	GO:0005856~cytoskeleton	50	10.40	1.9E-03	MAD1L1, MYH15, HINT1, FERM	300	1381	12782	1.54	5.0E-01	2.0E-01	2.6E+00
GOTERM_CC_FAT	GO:0043292~contractile fiber	10	2.08	2.1E-03	HDAC4, MYH15, NEB, SORBS2,	300	121	12782	3.52	5.3E-01	1.7E-01	2.9E+00
GOTERM_CC_FAT	GO:0030016~myofibril	9	1.87	4.5E-03	HDAC4, MYH15, NEB, SORBS2,	300	111	12782	3.45	8.0E-01	2.8E-01	6.0E+00

Category	Term	Count	%	PValue	Genes	List Total	Pop Hits	Pop Total	Fold Enrichment	Bonferroni	Benjamini	FDR
GOTERM_MF_FAT	GO:0008092~cytoskeletal protein binding	30	6.24	3.3E-05	TRIOBP, MYH15, SHROOM3, AE	330	504	12983	2.34	1.9E-02	1.9E-02	4.9E-02
GOTERM_MF_FAT	GO:0032559~adenyl ribonucleotide binding	59	12.27	6.1E-04	ADCY1, SPG7, MYH15, STK38, I	330	1497	12983	1.55	2.9E-01	1.6E-01	8.9E-01
GOTERM_MF_FAT	GO:0003779~actin binding	20	4.16	6.6E-04	TRIOBP, SHROOM3, MYH15, SI	330	326	12983	2.41	3.1E-01	1.2E-01	9.6E-01
GOTERM_MF_FAT	GO:0001883~purine nucleoside binding	62	12.89	6.6E-04	ADCY1, SPG7, MYH15, STK38, I	330	1601	12983	1.52	3.1E-01	8.9E-02	9.6E-01
GOTERM_MF_FAT	GO:0005524~ATP binding	58	12.06	7.6E-04	ADCY1, SPG7, MYH15, STK38, I	330	1477	12983	1.54	3.5E-01	8.2E-02	1.1E+00

Hypomethylated_pTSS_188-outliers_155-genes

Category	Term	Count	%	PValue	Genes	List Total	Pop Hits	Pop Total	Fold Enrichment	Bonferroni	Benjamini	FDR
GOTERM_BP_FAT	GO:0030510~regulation of BMP signaling pathway	3	1.94	1.6E-02	RGMA, SOST, PCSK6	89	30	13528	15.20	1	1	2.2E+01
GOTERM_BP_FAT	GO:0022037~metencephalon development	3	1.94	2.2E-02	HOXB1, NCOA2, CACNA1A	89	35	13528	13.03	1	1	2.9E+01
GOTERM_BP_FAT	GO:0007610~behavior	8	5.16	3.3E-02	ATXN1, SAA2, NPY, PPBP, RAS	89	469	13528	2.59	1	1	4.1E+01
GOTERM_BP_FAT	GO:0007626~locomotory behavior	6	3.87	3.3E-02	ATXN1, SAA2, PPBP, CACNA1A	89	274	13528	3.33	1	1	4.1E+01
GOTERM_BP_FAT	GO:0021548~pons development	2	1.29	3.8E-02	HOXB1, CACNA1A	89	6	13528	50.67	1	1	4.6E+01

Category	Term	Count	%	PValue	Genes	List Total	Pop Hits	Pop Total	Fold Enrichment	Bonferroni	Benjamini	FDR
GOTERM_CC_FAT	GO:0043005~neuron projection	6	3.87	7.4E-02	NRCAM, SNCB, ANK3, RASGRF	85	342	12782	2.64	1	1	6.2E+01
GOTERM_CC_FAT	GO:0042101~T cell receptor complex	2	1.29	7.6E-02	CD8A, CD8B	85	12	12782	25.06	1	1	6.2E+01

Category	Term	Count	%	PValue	Genes	List Total	Pop Hits	Pop Total	Fold Enrichment	Bonferroni	Benjamini	FDR
GOTERM_MF_FAT	GO:0004857~enzyme inhibitor activity	7	4.52	1.2E-02	CST8, SERPINE2, SNCB, CARD	93	270	12983	3.62	9.6E-01	9.6E-01	1.5E+01
GOTERM_MF_FAT	GO:0008092~cytoskeletal protein binding	8	5.16	6.6E-02	FRMD3, NRCAM, EPB41L2, SOI	93	504	12983	2.22	1	1	5.9E+01

Hypomethylated_clustered_106-outliers_28-genes

Category	Term	Count	%	PValue	Genes	List Total	Pop Hits	Pop Total	Fold Enrichment	Bonferroni	Benjamini	FDR
GOTERM_BP_FAT	GO:0008219~cell death	4	14.29	5.0E-02	NLRC4, RASGRF1, ARHGEF18,	17	719	13528	4.43	1	1	5.2E+01

Category	Term	Count	%	PValue	Genes	List Total	Pop Hits	Pop Total	Fold Enrichment	Bonferroni	Benjamini	FDR
GOTERM_CC_FAT	no terms detected											

Category	Term	Count	%	PValue	Genes	List Total	Pop Hits	Pop Total	Fold Enrichment	Bonferroni	Benjamini	FDR
GOTERM_MF_FAT	GO:0009374~biotin binding	2	7.14	6.5E-03	MCCC1, PCCA	18	5	12983	288.51	4.8E-01	4.8E-01	7.0E+00
GOTERM_MF_FAT	GO:0016885~ligase activity, forming carbon-carbon bonds	2	7.14	9.1E-03	MCCC1, PCCA	18	7	12983	206.08	6.0E-01	3.7E-01	9.7E+00
GOTERM_MF_FAT	GO:0033293~monocarboxylic acid binding	2	7.14	6.8E-02	MCCC1, PCCA	18	54	12983	26.71	1	9.1E-01	5.5E+01
GOTERM_MF_FAT	GO:0005089~Rho guanyl-nucleotide exchange factor activity	2	7.14	9.3E-02	RASGRF1, ARHGEF18	18	74	12983	19.49	1	9.1E-01	6.6E+01

Hypomethylated_delta-beta<-0.2&pTSS&clustered_30-outliers_13-genes

Category	Term	Count	%	PValue	Genes	List Total	Pop Hits	Pop Total	Fold Enrichment	Bonferroni	Benjamini	FDR
GOTERM_BP_FAT	GO:0007610~behavior	3	23.08	2.9E-02	NPY, RASGRF1, CACNA1A	9	469	13528	9.61	1	1	3.4E+01
GOTERM_BP_FAT	GO:0006766~vitamin metabolic process	2	15.38	4.4E-02	CYP24A1, MCCC1	9	75	13528	40.08	1	1	4.6E+01
GOTERM_BP_FAT	GO:0030534~adult behavior	2	15.38	5.0E-02	NPY, CACNA1A	9	86	13528	34.96	1	1	5.1E+01
GOTERM_BP_FAT	GO:0008219~cell death	3	23.08	6.4E-02	NLRC4, RASGRF1, CACNA1A	9	719	13528	6.27	1	1	6.0E+01
GOTERM_BP_FAT	GO:0016265~death	3	23.08	6.5E-02	NLRC4, RASGRF1, CACNA1A	9	724	13528	6.23	1	1	6.0E+01

Category	Term	Count	%	PValue	Genes	List Total	Pop Hits	Pop Total	Fold Enrichment	Bonferroni	Benjamini	FDR
GOTERM_CC_FAT	no terms detected											

Category	Term	Count	%	PValue	Genes	List Total	Pop Hits	Pop Total	Fold Enrichment	Bonferroni	Benjamini	FDR
GOTERM_MF_FAT	no terms detected											

A gene ontology term and associated information were shown in black when its Benjamini's corrected p-value is < 0.05, and otherwise shown in grey.

Supplementary Table 4: additional clinical informaton of the subjects

Subject [a]	BMI		Body weight (kg)		Gestational weght gain (kg)	Maternal complication [b]	Gestational week [c]	Caesarean section [d]		Newborn's gender	# of methylated outliers	
	pre-pregnancy	at delivery	pre-pregnancy	at delivery				Yes/No	Reason		hyper	hypo
FGR_insufficient_1	8.0	18.3	43.0	45.1	2.1		37	N		female	53	44
FGR_insufficient_2	20.0	21.1	57.0	60.4	3.4		35	Y	growth arrest	male	106	32
FGR_insufficient_3	17.6	19.6	44.0	49.0	5.0		37	N		male	105	30
FGR_insufficient_4	19.5	21.3	53.0	58.1	5.1		34	Y	growth arrest	female	60	29
FGR_insufficient_5	19.1	21.7	50.0	56.9	6.9		36	N		female	27	28
FGR_adequate_1	20.0	23.3	45.0	52.5	7.5		37	Y	due to previous caesarean delivery	female	44	23
FGR_adequate_2	17.9	21.0	43.0	50.5	7.5		37	Y	due to previous caesarean delivery	female	36	21
FGR_adequate_3	18.7	21.6	51.0	58.7	7.7		35	N		male	137	40
FGR_adequate_4	22.6	25.8	56.5	64.5	8.0		37	N		male	88	14
FGR_adequate_5	22.0	26.6	49.6	59.8	10.2		39	Y	request without clinical indication	female	34	18
FGR_excesive_1	18.4	23.5	46.0	58.8	12.8		32	Y	subamniotic hematoma	female	154	52
FGR_excesive_2	19.0	24.4	45.0	57.8	12.8		37	N		female	59	34
FGR_excesive_3	20.1	25.8	49.0	62.8	13.8		37	Y	breech presentation	female	21	23
FGR_excesive_4	20.7	27.3	46.0	60.5	14.5		39	N		female	41	30
Normal_insufficient_1	20.8	23.2	48.0	53.6	5.6	depression	41	N		female	382	39
Normal_insufficient_2	23.0	25.3	59.0	64.7	5.7		41	Y	arrest of labor	female	78	24
Normal_insufficient_3	19.1	21.6	49.0	55.2	6.2		37	N		female	146	22
Normal_insufficient_4	17.5	20.5	41.0	47.9	6.9		39	N		male	84	47
Normal_insufficient_5	18.1	21.1	43.0	50.0	7.0		40	N		female	55	23
Normal_adequate_1	22.6	25.5	56.5	63.6	7.1		40	N		male	40	24
Normal_adequate_2	20.1	23.0	51.5	59.0	7.5		38	N		female	40	28
Normal_adequate_3	22.9	26.2	55.0	63.0	8.0		35	N		male	23	23
Normal_adequate_4	20.7	24.1	53.0	61.6	8.6		38	N		male	43	24
Normal_adequate_5	18.0	21.0	52.0	60.7	8.7		39	N		male	42	18
Normal_adequate_6	20.1	23.3	54.0	62.8	8.8		37	Y	congenital dislocation of the hip	female	18	38
Normal_adequate_7	17.3	21.5	39.0	48.4	9.4		40	N		female	54	9
Normal_adequate_8	19.4	23.7	46.0	56.2	10.2		39	N		male	33	23
Normal_adequate_9	19.6	23.4	52.0	62.2	10.2		36	Y	due to previous caesarean delivery	male	46	28
Normal_excesive_1	20.5	26.1	50.0	63.5	13.5		40	N		male	54	43
Normal_excesive_2	19.2	24.8	48.0	61.8	13.8		41	N		male	80	27
Normal_excesive_3	20.7	26.1	57.0	71.9	14.9	autoimmune hepatitis	38	N		male	62	63
Normal_excesive_4	20.4	26.7	49.0	64.2	15.2		40	N		female	54	25
Normal_excesive_5	18.0	23.8	49.0	64.9	15.9	pituitary adenoma	39	Y	request without clinical indication	female	222	31

[a] All subjects did not smoke or drink alcohol during pregnancy.

[b] Women with pregnancy complications of gestational diabetes, pre-eclampsia, and pregnancy-induced hypertension were not enrolled in this study.
Maternal complications other than GDM, PE, and PIH are listed.

[c] No statistically significant correlation was detected between gestational weeks and the numbers of hyper- and hypomethylated outliers.
The preason's correlation coefficient (and p-value) were 0.094 (0.602) for hypermethylated outliers and 0.038 (0.832) for hypomethylated outliers.

[d] No statistically dignificant difference was detected for the numbers of hyper- and hypomethylated outliers between the C-section group (n=11) and vaginal delivery group (n=22).
The Wilcoxon rank sum test p-values were 0.479 for hypermethylated outliers and 0.632 for hypomethyltaed outliers.
